# Identification and validation of prognostic genes associated with clear cell renal cell carcinoma: based on public whole transcriptome sequencing datasets

**DOI:** 10.3389/fonc.2026.1857894

**Published:** 2026-07-08

**Authors:** Pengcheng Chang, Zitong Qin, Runzhang Liu, Binxian Wang, Huaiquan Lu, Suoshi Jing, Chenhao Guo, Weiping Li

**Affiliations:** 1 Department of Urology, The First Hospital of Lanzhou University, Lanzhou, Gansu, China; 2 The First Clinical Medical College of Lanzhou University, Lanzhou, Gansu, China

**Keywords:** clear cell renal cell carcinoma, prognostic genes, regulatory network, risk model, whole transcriptome sequencing

## Abstract

**Background:**

Clear cell renal cell carcinoma (ccRCC) is a prevalent malignancy, representing 80−90% of kidney cancer cases. This study aimed to identify potential prognostic genes to improve patient survival prediction and provide new insights into the pathogenesis and treatment of ccRCC through comprehensive whole transcriptome sequencing analysis.

**Methods:**

The analysis utilized whole transcriptomic data from publicly available datasets, including TCGA-KIRC and GSE96574. Methods involved identifying differentially expressed mRNAs and long non-coding RNAs, prognostic gene screening via MCODE plugin and risk model construction, followed by functional enrichment, molecular network construction, drug prediction, and molecular docking. The expression levels of key genes were subsequently validated using reverse transcription quantitative PCR (RT−qPCR) on clinical samples.

**Results:**

Eight prognostic genes (IFNG, CXCL13, KLRK1, LAG3, ITGAX, TNFRSF9, CD2, CD8B) were identified and formed a risk stratification model. These genes were primarily enriched in immune-related pathways such as antigen processing and presentation. A regulatory network involving transcription factors, miRNAs, lncRNA PVT1, and circRNAs was constructed. Drug prediction and molecular docking suggested potential targeted drugs, including amitriptyline hydrochloride for CD8B and IFNG, and rituximab for CXCL13 and IFNG, with strong binding affinities noted for ITGAX and KLRK1. RT−qPCR validation confirmed significantly elevated expression of IFNG, LAG3, TNFRSF9, and CD8B in ccRCC patients compared to controls (p < 0.05).

**Conclusions:**

This study identifies an eight-gene signature as a promising prognostic biomarker for ccRCC, deeply involved in the tumor immune microenvironment. The findings offer novel insights into ccRCC pathogenesis and highlight potential therapeutic targets and agents, paving the way for improved prognostic strategies and immunotherapeutic approaches.

## 1 Introduction

Renal cell carcinoma (RCC), a malignancy arising from the renal epithelium, has shown a persistent increase in incidence over the past decade, imposing significant strain on public health systems (1). Patients are typically diagnosed at a median age of ~60 years, with males outnumbering females 2:1 (2). Accounting for 80%-90% of renal malignancies, ccRCC represents the most prevalent histologic subtype of renal cell carcinoma (3, 4). The treatment of ccRCC primarily involves curative-intent surgical resection and systemic therapies including targeted therapy and immunotherapy (5). Although ccRCC is a disease detectable at early stages and amenable to successful treatment through surgical intervention or ablative strategies, 20-30% of patients experience metastatic recurrence postoperatively, while some develop primary or secondary resistance during systemic treatments (6, 7). These issues represent critical challenges in current clinical practice and the molecular mechanisms underlying ccRCC tumorigenesis and progression have garnered significant scientific attention. The pathogenesis of ccRCC is initiated by landmark genetic events including 3p deletion and VHL mutation, while its malignant progression is mediated through DNA repair deficiency and mitotic errors. These molecular aberrations drive genomic instability and promote aneuploidy formation (8). During disease evolution, synergistic interactions between key chromosomal gains/losses, PI3K pathway component mutations, and secondary hits in other oncogenic drivers progressively enhance tumor cell invasiveness and metastatic potential, ultimately culminating in adverse clinical outcomes (8). Consequently, uncovering and establishing innovative prognostic biomarkers/models for ccRCC proves essential to deciphering the molecular pathogenesis of this malignancy and accelerating patient-tailored therapeutic paradigms (9).

Transcriptome sequencing refers to the targeted sequencing of a defined gene set or transcriptomic subset, enabling investigation of specific gene expression patterns through systematic analysis of alterations in mRNA abundance (10). Whole transcriptome sequencing encompasses comprehensive profiling of the entire transcriptome, including all protein-coding mRNAs, non-coding RNAs, splice variants, and post-transcriptional modifications (11). Whole transcriptome sequencing has emerged as a pivotal tool for disease mechanism investigation, precision diagnostics, and targeted therapy development by systematically elucidating gene expression networks to generate multidimensional biological insights. With the widespread adoption of single-cell and spatial transcriptomic technologies, its applications are poised to enhance the depth of disease research and further advance clinical translation. Through whole transcriptome sequencing, disease-specific expression signatures can be systematically characterized to stratify and predict pathological progression, ultimately informing precision therapeutic strategy development (12).

This study integrated mRNA and lncRNA expression profiles of clear cell renal cell carcinoma patients and normal controls from public databases. Through differential expression analysis, we identified critical prognostic genes (e.g., IFNG, CXCL13, PVT1) and elucidated pivotal genes exerting essential roles in ccRCC pathogenesis and progression. These findings provide an integrated technological platform and theoretical framework for precision diagnosis, prognostic evaluation, and targeted therapy of ccRCC, thereby propelling personalized diagnostic and therapeutic paradigms.

## 2 Materials and methods

### 2.1 Data source

The cancer genome atlas kidney renal clear cell carcinoma (TCGA-KIRC) was retrieved from The Cancer Genome Atlas (TCGA) database, accessible at (https://portal.gdc.cancer.gov/) and was utilized as both mRNAs and lncRNAs training sets. This dataset consists of 535 ccRCC patient tumor tissue samples as the ccRCC group and 72 adjacent non-cancerous tissue samples as the control group. Notably, a total of 530 tumor samples had survival information available within them. In addition, GSE96574 (platform: GPL1961) was taken from Gene Expression Omnibus (GEO) database, available at (https://www.ncbi.nlm.nih.gov/geo/) as another training set of lncRNAs and included 5 ccRCC patient tumor tissue samples and 5 adjacent non-cancerous tissue samples. Meanwhile, E-MTAB-1980 was acquired from the ArrayExpress database (https://www./arrayexpress/ArrayExpress) as a validation set of mRNAs, including 101 ccRCC patient tumor tissue samples. The survival endpoint for this dataset is overall survival (OS), defined in accordance with the TCGA-KIRC protocol. The dataset employs the same gene expression quantification methods and standardization procedures as TCGA, enabling direct external validation of the model. The download time for all data was August 15th, 2024.

### 2.2 Differential expression analysis

To detect differentially expressed mRNAs (DE-mRNAs) from TCGA-KIRC dataset, DESeq2 package (v 1.42.0) was employed with thresholds of adjusted p < 0.05 and |log2 Fold Change (FC)| > 3 (13). This stringent fold-change cutoff was applied to prioritize genes with substantial expression alterations, reducing false positives from marginal changes in transcriptomic data. A volcano map was generated by ggplot2 package (v 3.4.2), which marked the top 10 DE-mRNAs with the highest |log2FC| in both up and downregulated (14). A heatmap illustrating profiles for the ten most significantly up- and downregulated genes was generated utilizing the ComplexHeatmap package (v 2.16.0), ranked by |log2FC| values (highest to lowest) (15). At the same time, the differentially expressed TCGA lncRNAs (DE-TCGA-lncRNAs) were identified with DESeq2, using p < 0.05 and |log2 FC| > 1 as cutoffs. The ggplot2 and ComplexHeatmap packages were employed to generate a volcano map and heatmap, which marked and visualized the top 10 DE-TCGA-lncRNAs with the highest |log2FC| in both up-regulated and down-regulated. The differentially expressed GEO lncRNAs (DE-GEO-lncRNAs) between ccRCC and normal samples in GSE96574 were identified using the limma package (v 3.56.2), with the screening criteria was p < 0.05 and |log2 FC| > 1 (16). The ggplot2 and ComplexHeatmap packages were employed to generate a volcano map and heatmap, showcasing all the DE-GEO-lncRNAs. Finally, to select key differentially expressed lncRNAs (DE-lncRNAs), we intersected the up-regulated and down-regulated lncRNAs from both the DE-TCGA-lncRNAs and DE-GEO-lncRNAs.

### 2.3 Functional enrichment and protein-protein interaction network analyses

To functionally characterize the roles and associated pathways of DE-mRNAs, Gene Ontology (GO) analysis and Kyoto Encyclopedia of Genes and Genomes (KEGG) of DE-mRNAs were screened by clusterProfiler package (v 4.8.2) with a significance level of p < 0.05 (17). GO analysis involved examining three aspects: biological processes (BP), molecular function (MF), and cellular component (CC), and displaying the top five most significantly enriched terms for each category. In addition, the KEGG pathways were presented in a ranked order, starting with the lowest p-values (p < 0.05), highlighting the most significant 10 pathways. Furthermore, PPI network with a confidence level of 0.4 was constructed involving the DE-mRNAs, by drawing upon the Search Tool for the Retrieval of Interacting Genes (STRING) database (https://cn.string-db.org/) to delve deeper into protein-protein relationships, and were visualized in Cytoscape software (v 3.10.2) (18). Afterwards, the MCODE plugin was utilized to screen for high-correlation gene clusters, treating these genes as candidate genes for subsequent examination.

### 2.4 Identification of prognostic genes and risk model construction and validation

Utilizing the caret package (v 6.0.94), a training and testing dataset was divided from the 530 primary tumor samples from TCGA-KIRC with complete survival data, maintaining a 7:3 ratio (19). The dataset comprised 371 samples for training and 159 samples for testing. The survival package (v 3.7.0) performed univariate Cox regression to detect overall survival (OS)-associated genes, with significance thresholds of p < 0.05 and HR ≠ 1 (20). The glmnet package (v 4.1.8) was employed, set the parameter family to Cox to achieve least absolute shrinkage and selection operator (LASSO) regression (21). Ten-fold cross-validation was implemented with 5 repeats (random seed = 68) to select the optimal lambda, defined as the lambda value minimizing the partial likelihood deviance. Genes having non-zero coefficients were subjected to the proportional hazard (PH) assumption check, where p > 0.05 indicates no evidence of violation included as prognostic genes. Finally, prognostic genes were used for constructing the risk model with following formula: risk score = $\sum_{\text{i}=1}^{\text{n}}(\text{expi}\times {\text{β}}_{\text{i}}\;)$, ${\text{β}}_{\text{i}}$ was the regression coefficient for prognostic genes, and expi denotes their expression levels. Applying the validated risk threshold (optimal specificity risk score: training set: 1.1723, testing set: 0.9338, validation set: 9.8361), ccRCC samples were bifurcated into high-risk and low-risk prognostic categories. Subsequently, the risk scores, OS times, and expression patterns of prognostic genes were worked out for every ccRCC patient. The Kaplan-Meier (K-M) survival curves of disease-free survival (DFS) were generated by survminer package (v 0.4.9) to contrast the survival outcomes between two risk groups (p < 0.05) (22). To further assess the validity of the risk models from training set, the survivalROC package (v 1.0.3.1) was plotted the receiver operating characteristic (ROC) with 1-, 3-, 5-year as the survival time nodes, calculating the area under the curve (AUC) (23). AUC values exceeding 0.6 signified informative predictive capacity. It should be noted that to further validate the results, the above analyses were also conducted on the testing set and validation set E-MTAB-1980. To evaluate the independent predictive capacity of risk scores and clinical characteristics (age, gender, stage, T stage, N stage, M stage) for overall survival (OS) in the TCGA-KIRC training cohort, multivariate Cox regression analyses were performed using the survival package (v 3.5-7). Variables with p < 0.1 in univariate analysis were included in the multivariate Cox model to calculate hazard ratios (HR) and 95% confidence intervals (CI). The proportional hazards (PH) assumption was tested via Schoenfeld residuals, with p > 0.05 indicating no violation. Based on the multivariate Cox results, a nomogram was constructed using the rms (v 8.1-1) to predict 1/3/5-year OS, and the time-dependent concordance index (C-index) was calculated. Calibration curves were plotted with rms to assess the consistency between predicted and actual probabilities, time-dependent receiver operating characteristic (ROC) curves (AUC) were drawn using the survivalROC(v 1.0.3.1), and decision curve analysis (DCA) was conducted to evaluate clinical net benefit.

### 2.5 Clinical characteristic analysis

To investigate correlations linking clinical characteristics to the risk model, we first excluded samples with missing clinical-pathological data (including age, gender, TNM stage). Finally, 174 samples with complete profiles were selected from the original 371 training samples for analysis. Missing data were not imputed to avoid bias from artificial filling. We classified samples into subtypes using clinicopathological parameters: age (> 60 and ≤ 60 years), gender, M stage, T stage, N stage, and Stage (I, II, III, IV). Box plots and heatmaps were generated using the ggplot2 and ComplexHeatmap packages, respectively. The ccRCC samples were then stratified into high and low-risk groups by their optimal risk scores. The Wilcoxon test was conducted to compare the risk scores across different clinical characteristics (age, stage, T stage, N stage, and M stage) with a significance level of p < 0.05 to indicate significant differences. Categorical variables stratified by high- versus low-risk status were subjected to chi-square testing, assessing associations linking risk scores with demographic and tumor characteristics.

### 2.6 Immune microenvironment and checkpoint analysis

To evaluate differences in immune infiltration between high- and low-risk groups, based on tumor samples with survival information in the TCGA-KIRC training cohort, enrichment scores of 28 immune cells were calculated using the ssGSEA algorithm in the R package GSVA (v 1.46.0) (24), inter-group differences were tested by Wilcoxon tests (R package stats); correlations were analyzed by Spearman method using the R package psych (v 2.2.8); plots were generated with the R package ggplot2 (v 3.5.2). Immune checkpoint analysis followed the same methods; inflammatory signatures used the MSigDB HALLMARK INFLAMMATORY RESPONSE gene set.

### 2.7 Gene set enrichment analysis and associated pathway analyses

To characterize the biological functions and pathways underlying ccRCC pathogenesis mediated by prognostic genes, based on the training set with 371 samples, a reference gene set “c2.cp.kegg_medicus.v2023.2.Hs.symbols.gmt” from the Molecular Signatures Database (MSigBD) (https://www.gsea-msigdb.org/gsea/msigdb/) was chosen. Subsequently, GSEA was executed with the clusterProfiler package to identify significantly enriched pathways with a significance level of p < 0.05. Plots were generated based on the top 5 significantly enriched pathways for each prognostic gene. A ridge plot depicted the expression patterns of core genes from the enriched pathways, where positive and negative values signified upregulation and downregulation, respectively. Following that, calculating the single sample gene set enrichment analysis (ssGSEA) scores of enriched pathways in the training set with 371 samples, using the ssGSEA algorithm from the GSVA package (v 1.49.4) (24). The differential analysis was then conducted using the limma package, comparing the differences in pathway scores between two risk categories. Significant differential pathways between risk groups were identified with |t| > 2 and p < 0.05.

### 2.8 Chromosome localization and GeneMANIA analyses of prognostic genes

To delve into the genomic context of the prognostic genes, we accessed chromosome localization data from the ENSEMBL database (https://www.ensembl.org/). The RCircos package (v 1.2.2) was used to draw the location map of prognostic genes on chromosomes in the training set (25). Aiming to characterize prognostic genes’ interplay and biological roles, a gene-gene interaction network was constructed using prognostic genes and other functionally similar genes through the GeneMANIA online platform (http://genemania.org/) to analyze their functional relevance, with an FDR (false discovery rate) threshold of 0.05 to ensure a reliable network analysis and functional coherence.

### 2.9 Regulatory network analysis of prognostic genes

To investigate transcription factors (TFs) that target the prognosis genes, NetworkAnalyst database (https://www.networkanalyst.ca/) was employed to identify TFs that regulate these genes. Subsequently, using Cytoscape software to show this regulatory network. Next, to delve deeper into the molecular regulation, miRTarBase v9.0 and TarBase v9.0 databases from NetworkAnalyst (https://www.networkanalyst.ca/) were used to predict microRNAs (miRNAs) that could interact with the prognosis genes. Then selected from the intersection to obtain the targeted miRNAs. MiRNet (https://www.mirnet.ca/miRNet/home.xhtml) was then employed to predict lncRNAs that targeted these miRNAs, which were intersected with the DE-lncRNAs identified previously. The overlapping lncRNAs, showing consistent regulatory trends with the prognosis genes, were selected as the final predicted intersected lncRNAs. Finally, utilizing the ENCORI database (https://rnasysu.com/encori/index.php), circular RNAs (circRNAs) that were predicted (TDMDScore ≥ 2) for miRNA targeting were identified. All these findings were used to construct a circRNAs/lncRNAs-miRNAs-mRNAs network based on Cytoscape software.

### 2.10 Drug prediction and molecular docking

The Drug-Gene Interaction Database (DGIdb v4.0) (https://www.dgidb.org/) was employed to determine potential drugs linked to prognosis genes. The visualization of the regulatory network was constructed utilizing Cytoscape software. Additionally, the molecular structures of predicted drugs were accessed through the public chemical database (Pubchem) (https://pubchem.ncbi.nlm.nih.gov). Later on, to investigate the proteins at play, Protein Data Bank (RCSB PDB, https://www.rcsb.org/) was accessed for the 3D crystal structures of the prognosis genes. Molecular docking studies were performed with the target proteins and active molecules through CB-Dock2 (https://cadd.labshare.cn/cb-dock2/index.php). Typically, a binding affinity is considered strong when the docking score (binding energy) falls below -5 kcal/mol, indicating a robust compound-target interaction.

### 2.11 Reverse transcription quantitative polymerase chain reaction

The samples of 5 ccRCC tumor tissue samples and 5 matched adjacent non-cancerous tissue samples were taken at The First hospital of Lanzhou University. This study received approval from Ethics Committee of The First hospital of Lanzhou University (LDYYLL2025-896), and informed consent was obtained from all participants. Given the limited sample size and the priority of core immune-related genes identified in bioinformatic analyses, expression levels of five representative prognostic genes (IFNG, LAG3, ITGAX, TNFRSF9, and CD8B) were selected for RT-qPCR validation. Expression analysis of five key prognostic genes was performed using the following reagents and instruments: Total RNA was extracted using TRizol reagent (Ambion, Austin, USA; 15596-018CN) according to the manufacturer’s protocol. RNA concentration and purity were measured using a NanoPhotometer N50 spectrophotometer (Implen, Munich, Germany), with A260/A280 ratios of 1.8–2.0 and A260/A230 ratios > 0.5 indicating high-quality RNA. Reverse transcription was performed using Hifair^®^ III 1st Strand cDNA Synthesis SuperMix for qPCR (gDNA digester plus) (Yeasen Biotechnology, Shanghai, China; 11141ES60) in a 20 μL reaction system containing 2 μg total RNA, incubated at 25 °C for 5 min, 50 °C for 15 min, and 85 °C for 5 s. Quantitative PCR was conducted using 2×Universal Blue SYBR Green qPCR Master Mix (Servicebio, Wuhan, China; G3326) on a CFX Connect Real-Time PCR System (Bio-Rad, Hercules, USA; XLFZ006) with the following cycling conditions: pre-denaturation at 95 °C for 1 min, followed by 40 cycles of denaturation at 95 °C for 20 s, annealing at 55 °C for 20 s, and extension at 72 °C for 30 s. Melting curve analysis (60–95 °C, increment 0.5 °C/5 s) was performed to confirm single-product specificity (**Supplementary Figure 4**). GAPDH served as the endogenous control gene for normalization. Gene expression levels were calculated through 2−ΔΔCt method (26). Finally, Graphpad Prism (v 5.0.0) was employed to plot and calculate the P value (27).

### 2.12 Statistical analysis

All analyses were conducted using the R software (v 4.3.3). Statistical significance (p < 0.05) was assessed using the Wilcoxon test for specific comparisons, while between-group differences in PCR measurements were evaluated using t-tests. ns: no significant, *p < 0.05, **p < 0.01, ***p < 0.001, ****p < 0.0001.

## 3 Results

### 3.1 Identification of DE-mRNAs and key DE-lncRNA in ccRCC

**Figure 1:**
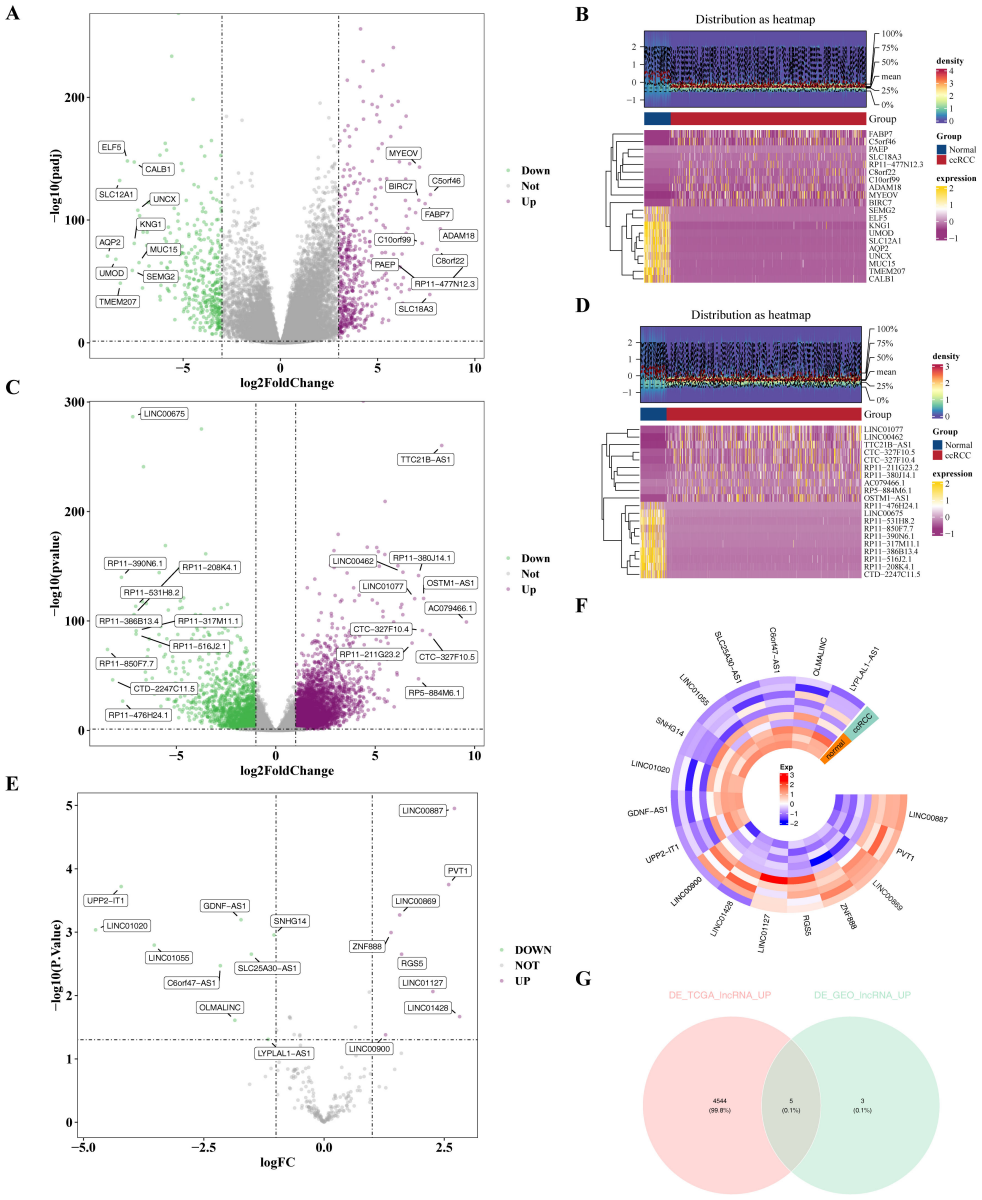
Plots of differential gene screening and key lncRNA Identification in ccRCC. **(A)** Volcano plot of mRNAs in ccRCC tumor tissues (n = 535) vs. adjacent normal tissues (n = 72) from TCGA-KIRC. Purple dots indicate upregulated mRNAs, green dots indicate downregulated mRNAs, and the top 10 most significantly differentially expressed mRNAs are labeled. **(B)** Heatmap of the top 10 most significantly upregulated and downregulated mRNAs in ccRCC vs. normal tissues, with columns representing genes and rows representing samples. **(C)** Volcano plot of lncRNAs in ccRCC vs. normal tissues from TCGA-KIRC. Purple dots indicate upregulated lncRNAs, green dots indicate downregulated lncRNAs, and the top 10 most significantly differentially expressed mRNAs are labeled. **(D)** Heatmap of the top 10 most significantly differentially expressed lncRNAs in TCGA-KIRC. **(E)** Volcano plot of lncRNAs in ccRCC umor tissues (n = 85) vs. normal tissues (n = 5) from GSE96574. **(F)** Circular heatmap of differentially expressed lncRNAs in GSE96574 (85 tumor, 5 normal). Red: high expression; blue: low. Tumors cluster distinctly. **(G)** Venn diagram showing overlapping upregulated lncRNAs between TCGA-KIRC and GSE96574, with the final 9 key DE-lncRNAs highlighted.

After differential expression analysis in TCGA-KIRC, 824 DE-mRNAs were obtained between ccRCC and normal tissues, with 461 upregulated and 363 downregulated. (**Figures 1A, B**). Within TCGA-KIRC, 5,907 DE-TCGA-lncRNAs were distinguished, consisting of 4,549 up-regulated and 1,358 down-regulated (**Figures 1C, D**). Within GSE96574 dataset, 17 DE-GEO-lncRNAs were discerned, featuring 8 upregulated and 9 downregulated in ccRCC samples. (**Figures 1E, F**). An intersection analysis of the upregulated and downregulated DE-lncRNAs (DE-TCGA-lncRNA and DE-GEO-lncRNA), respectively. This resulted in 5 upregulated and 4 downregulated DE-lncRNAs. By merging the overlapping genes, we ultimately identified 9 key DE-lncRNAs (**Figure 1G**).

### 3.2 Functional analysis and the PPI network of DE-mRNAs

**Figure 2:**
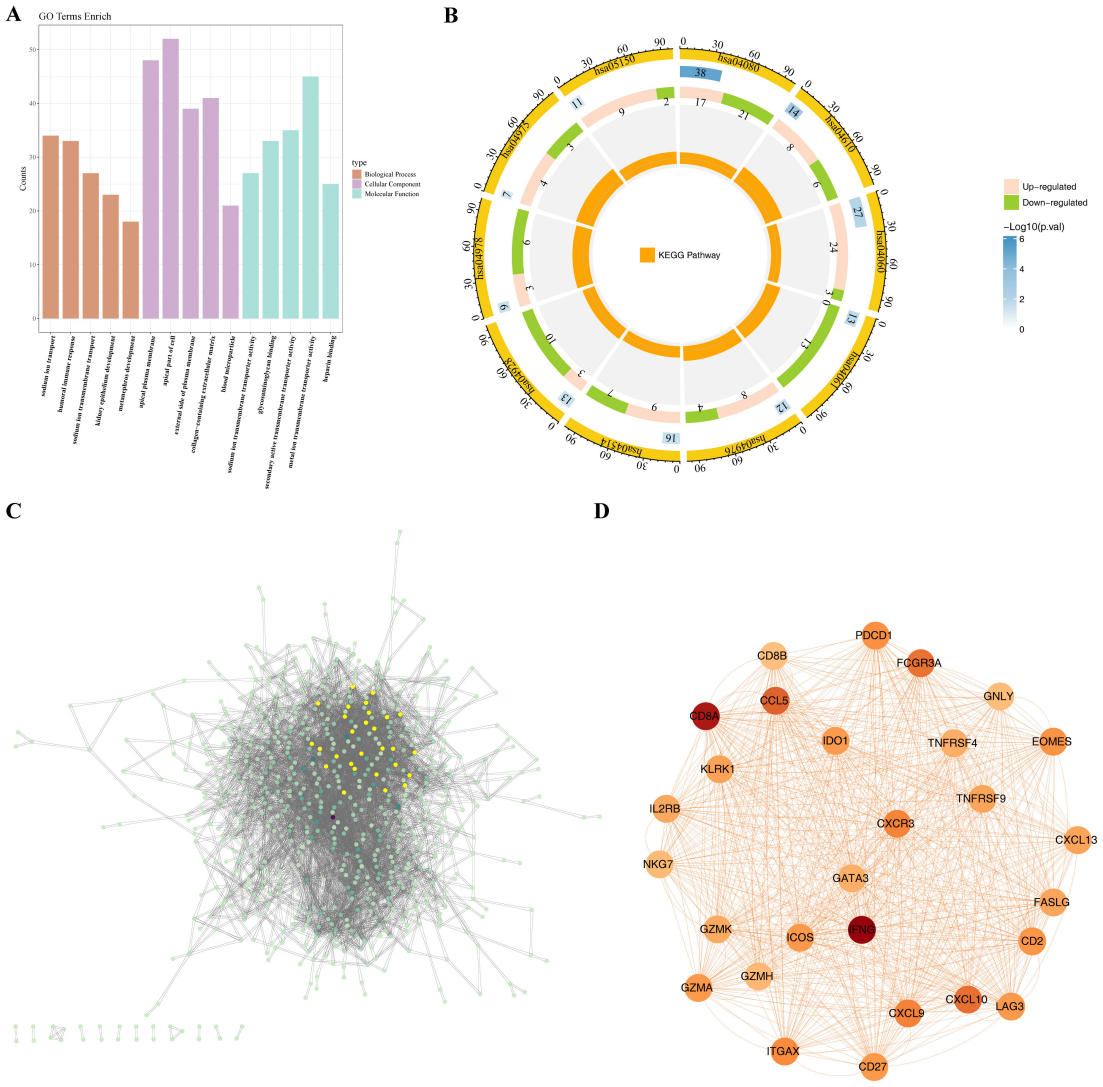
Functional analysis and the PPI network of DE-mRNAs. **(A)** GO enrichment analysis of differentially expressed mRNA genes. **(B)** Top KEGG pathways enriched by differentially expressed mRNAs. **(C)** Protein-protein interaction (PPI) network of differentially expressed mRNAs. **(D)** Network of high-correlation genes identified from PPI analysis; GO, gene ontology; BP, biological process; CC, cellular component; MF, molecular function; KEGG, kyoto encyclopedia of genes and genomes; PPI, protein-protein interaction.

The DE-mRNAs were significantly enriched in 1,091 GO terms (p < 0.05), of which 871 BPs, such as sodium ion transport and humoral immune response, and 76 CCs, such as apical cellular domains (plasma membrane and apical region) and 144 MFs, such as sodium ion transmembrane transporter activity and glycosaminoglycan binding (**Supplementary Table 2**). The top 5 significant terms for each category were shown in **Figure 2A**. Similarly, 34 KEGG pathways were enriched, with a p-value cutoff of p < 0.05, which primarily encompassing neurotransmitter-receptor signaling, complement activation, and coagulation cascades (**Supplementary Table 3**). The top 10 KEGG pathways were presented in **Figure 2B**. The PPI network analysis identified 696 proteins of DE-mRNAs that exhibited strong interactions with other proteins (**Figure 2C**), and selected 28 high-correlation genes as candidate genes including GNLY, CXCR3, FCGR3A, CD8A, TNFRSF4, TNFRSF9, ICOS, CD2, GZMK, EOMES, CXCL9, GZMH, NKG7, CXCL13, PDCD1, KLRK1, IDO1, LAG3, CD27, FASLG, IL2RB, ITGAX, CD8B, CXCL10, CCL5, GATA3, GZMA, and IFNG (**Figure 2D**).

### 3.3 Identification of 8 prognostic genes and construction of a risk model

**Figure 3:**
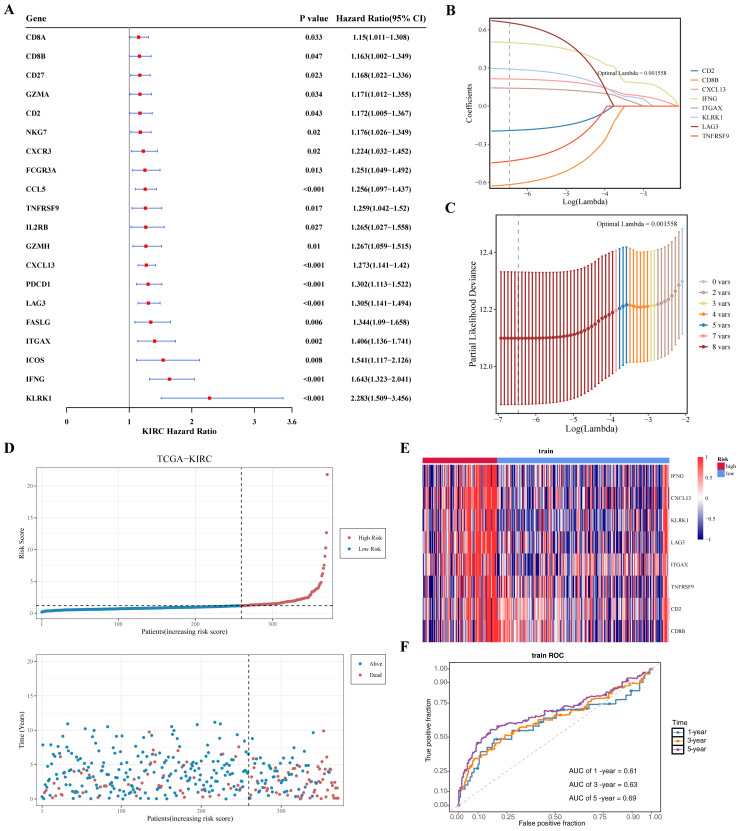
Prognostic gene identification and risk model construction in ccRCC. **(A)** Univariate cox analysis of candidate prognostic genes. **(B)** LASSO regression analysis for gene selection. **(C)** Partial likelihood deviance plot for optimal lambda selection. **(D)** Risk score distribution and prognostic stratification. **(E)** Kaplan-Meier survival analysis for high-risk and low-risk groups. **(F)** ROC curves for 1-, 3-, and 5-year survival prediction.

Through univariate Cox analysis 20 genes of candidate genes with significant effects on patient survival status were acquired based on the training set, which were IFNG, CXCL13, KLRK1, LAG3, CCL5, PDCD1, ITGAX, FASLG, ICOS, GZMH, FCGR3A, TNFRSF9, CXCR3, NKG7, CD27, IL2RB, CD8A, GZMA, CD2, and CD8B (HR > 1, p < 0.05) (**Figure 3A**). LASSO regression analysis then narrowed down the list to 8 genes demonstrating survival links (optimal lambda = 0.001558), which were IFNG, CXCL13, KLRK1, LAG3, ITGAX, TNFRSF9, CD2, and CD8B (**Figures 3B, C**). Subsequent PH assumption test was confirmed that all 8 prognostic genes associated with survival were included in the construction for risk model (p > 0.05) (**Supplementary Figure 1**). Risk scores were calculated based on 8 prognostic genes, and the formula for calculating the model: risk score = 0.4996 × IFNG + 0.2130 × CXCL13 + 0.2912 × KLRK1 + 0.6558 × LAG3 + 0.1411 × ITGAX + -0.4331 × TNFRSF9 + -0.1925 × CD2 + -0.6178 × CD8B. Then, based on a risk score threshold of 1.1723 (determined to optimally discriminate prognosis), ccRCC patients in the training cohort (n=371) were stratified as high-risk (n = 112) or low-risk (n = 259). Shorter survival times and heightened mortality correlated directly with augmented risk scores, as revealed by prognostic stratification patterns (**Figure 3D**). The K-M survival curves revealed significantly lower OS for the high-risk group (p < 0.0001) (**Figure 3E**). Subsequent ROC analyses at 1, 3, and 5 years resulted AUCs of 0.61, 0.63, and 0.69, respectively. These values all greater than 0.6, indicated good moderate predictive capacity of the risk model (**Figure 3F**).

**Figure 4:**
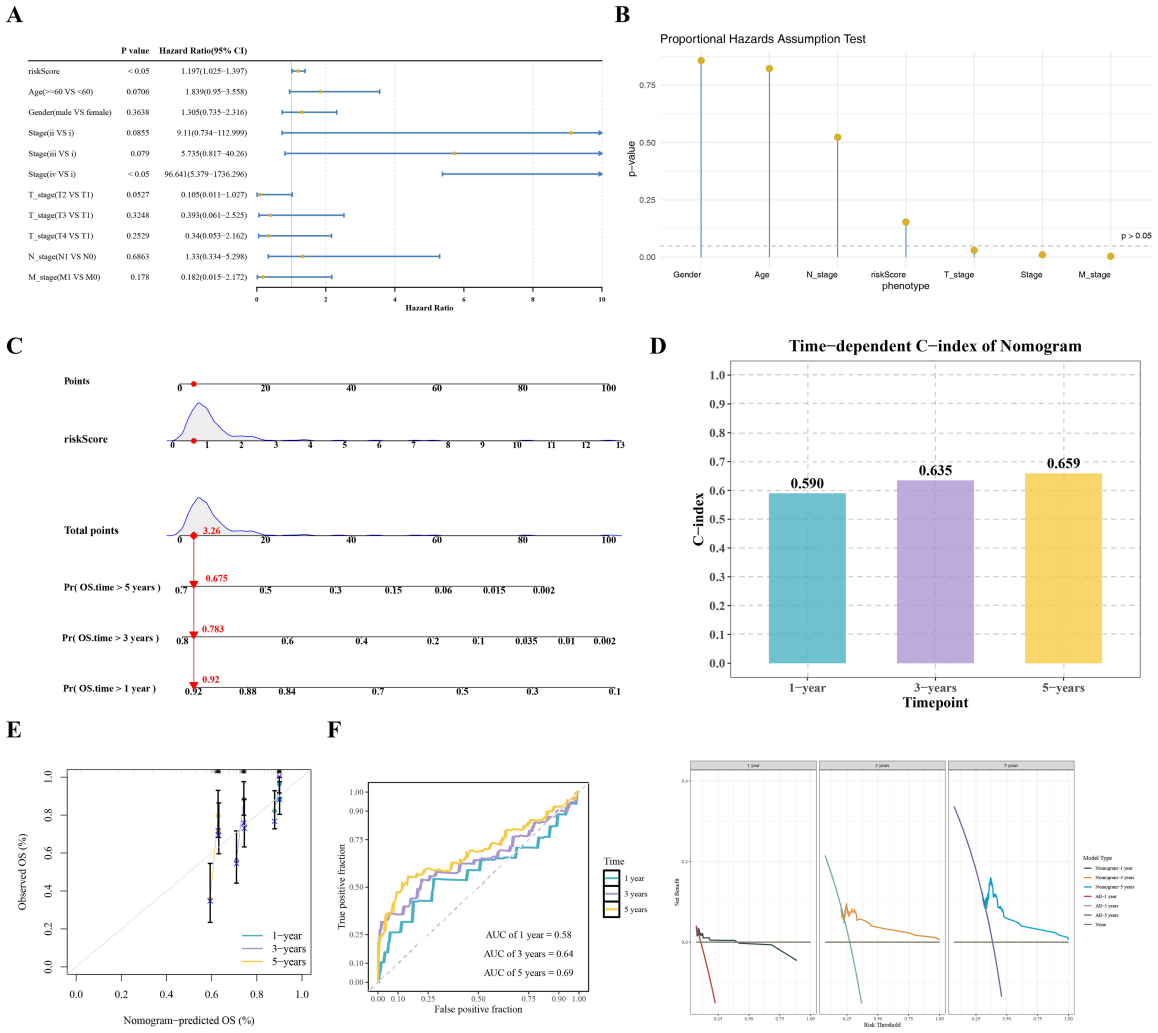
Multivariate cox regression analysis and prognostic model validation. **(A)** Forest plot showing riskScore as an independent prognostic factor for OS after adjusting for clinical variables (HR = 1.197, 95% CI = 1.025–1.397, p < 0.05). **(B)** PH assumption test indicating time-varying hazard ratios for riskScore, T stage, M stage, and overall stage (p < 0.05), while age, gender, and N stage satisfied the assumption (p > 0.05). **(C)** Nomogram integrating riskScore and clinical factors to predict 1-, 3-, and 5-year OS probabilities. **(D)** Time-dependent C-index analysis demonstrating improved discrimination over time (1-, 3-, and 5-year predictions, respectively). **(E)** Calibration curves showing high consistency between predicted and observed survival rates for 3- and 5-year OS (slopes ≈ 1), with suboptimal 1-year calibration (AUC = 0.58). **(F)** ROC curves (left) and decision curve analysis (DCA, right) indicating moderate predictive efficiency (3- and 5-year AUC = 0.64 and 0.69) and clinical utility outperforming the “no intervention” strategy across most risk thresholds.

Multivariate Cox regression analysis (**Figure 4**) showed that after adjusting for age, gender, TNM stage, and other variables, riskScore remained an independent prognostic factor for OS (HR = 1.197, 95%CI=1.025-1.397, p < 0.05) (**Figure 4A**). The PH assumption test (**Figure 4B**) revealed that riskScore (p < 0.05), T stage, M stage, and overall stage violated the PH assumption (p < 0.05), indicating that the hazard ratio varied over time; age, gender, and N stage satisfied the PH assumption (p > 0.05). This suggests that time-dependent effects should be considered in model prediction. A nomogram was constructed based on multivariate Cox results (**Figure 4C**), predicting 1/3/5-year OS probabilities by summing single scores of risk score, age, and other factors. Time-dependent C-index analysis (**Figure 4D**) showed C-index values of 0.590, 0.635, and 0.659 for 1/3/5 years, respectively, with slightly improved discrimination over follow-up time. Calibration curves (**Figure 4E**) showed high consistency between predicted and observed survival rates for 3/5 years (slopes close to 1), while 1-year calibration was suboptimal (AUC = 0.58). ROC curves (upper right) showed 3/5-year AUC values of 0.64 and 0.69 (>0.6), indicating moderate predictive efficiency. DCA demonstrated that the model outperformed the ‘no intervention’ strategy at most risk thresholds (**Figure 4F**).

### 3.4 Risk prognostic model as effective tools for survival prediction

Furthermore, the risk model was validated in the testing cohort. The optimal risk score threshold, determined as 0.9338, was applied for this validation. Risk stratification of the 159 testing samples identified 88 high-risk and 71 low-risk subgroups, with significantly distinct survival trajectories between cohorts (**Supplementary Figure 2A**). The K-M survival curves for two risk groups illustrated a significantly lower OS in the high-risk group (p < 0.0001) (**Supplementary Figure 2B**). Subsequent ROC analyses at 1, 3 and 5 years had an AUC greater than 0.6 (**Supplementary Figure 2C**). Finally, the risk model was validated within E-MTAB-1980 dataset with the best threshold of risk score was obtained as 9.8361. In this validation set of 101 samples, patients were categorized into high-risk (n = 31) and low-risk groups, revealing a worse prognosis for survival in the high-risk group (**Supplementary Figure 2D**). The K-M survival curves for two risk groups illustrated lower OS in the high-risk group (p = 0.0170) (**Supplementary Figure 2E**). Subsequent ROC analyses at 1, 3 and 5 years displayed AUC values above 0.6, confirming the model’s potential predictive utility (**Supplementary Figure 2F**).

### 3.5 Analysis of correlations between risk model and clinical characteristics

**Figure 5:**
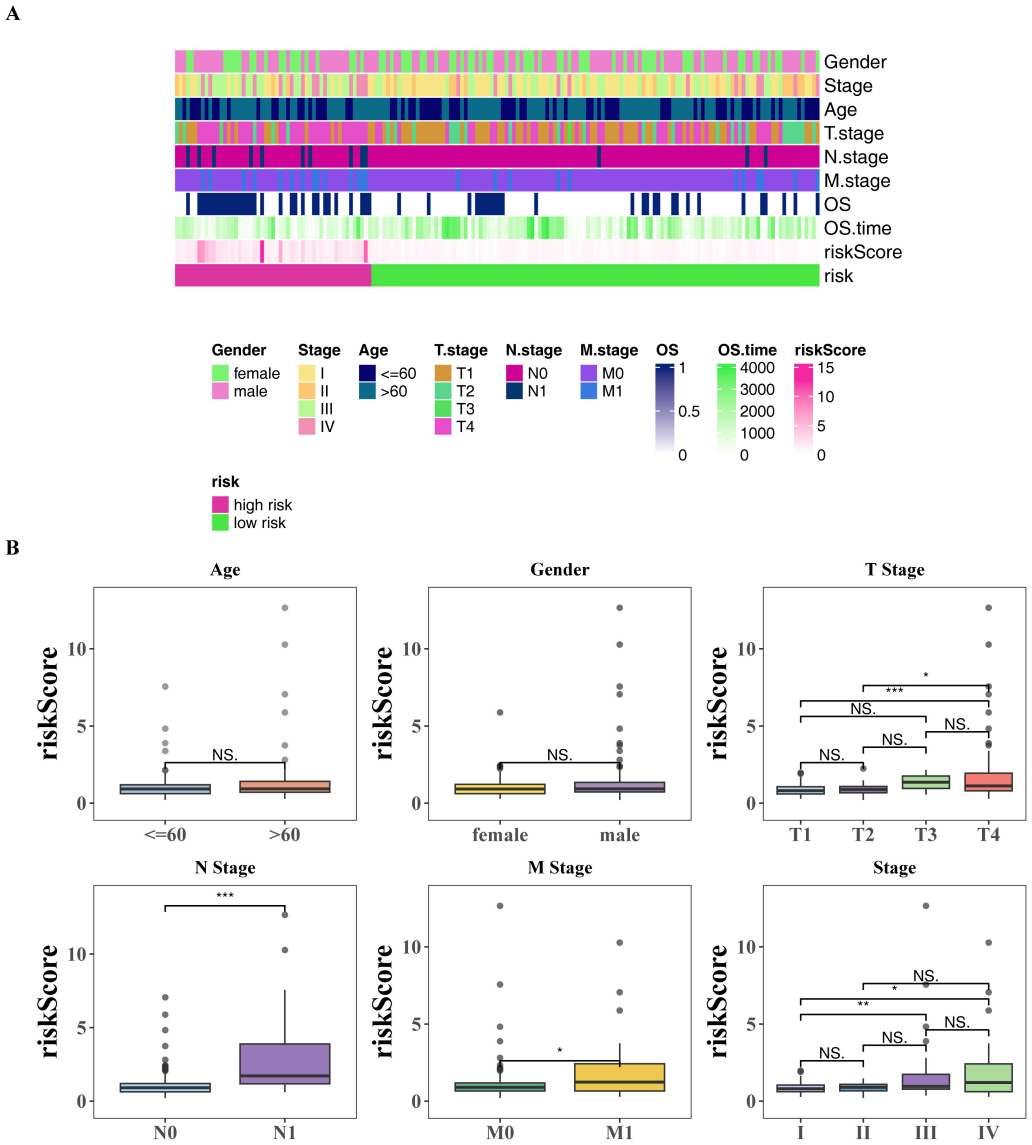
Risk score distribution and clinical correlation analysis in ccRCC. **(A)** Risk score distribution in the external validation set, with the optimal cutoff value determined by the Youden index. **(B)** Kaplan-Meier survival curves for the risk model in the external validation set (high-risk n=53, low-risk n=121). **(B)** Kaplan-Meier survival curves for the risk model in the external validation set (high-risk n=53, low-risk n=121).

In the training set of 371 samples, the risk score distribution across various clinical parameters was visualized in a heatmap (**Figure 5A**). The Wilcoxon test did not reveal significant differences in risk scores based on age or gender (p > 0.05). However, statistically significant differences were found in risk scores when comparing T1 to T4 stages, T2 to T4 stages, N0 to N1 stages, M0 to M1 stages, and stages I to III, as well as between stages I and IV (p < 0.05) (**Figure 5B**). Significant stratification (p<0.05) was observed for tumor staging parameters (T stage, N stage, M stage) and survival metrics – including OS status and survival time – when contrasting risk-stratified subpopulations in the initial clinical correlation analysis (**Table 1**). This analysis highlights the relevance of the risk model in stratifying patients and aids in tailoring treatment plans for more precise risk management in ccRCC.

**Table 1:** Clinical correlation analysis of risk-stratified subpopulations.

Characteristic	Subgroup	High-risk	Low-risk	P‑value	Test
n		53	121		
Gender (%)	female	20 (37.7)	51 (42.1)	0.706	
	male	33 (62.3)	70 (57.9)		
Stage (%)	I	12 (22.6)	57 (47.1)	0.001	
	II	4 (7.5)	19 (15.7)		
	III	23 (43.4)	33 (27.3)		
	IV	14 (26.4)	12 (9.9)		
Age (%)	<=60	20 (37.7)	51 (42.1)	0.706	
	>60	33 (62.3)	70 (57.9)		
T.stage (%)	T1	13 (24.5)	58 (47.9)	0.001	
	T2	5 (9.4)	24 (19.8)		
	T3	1 (1.9)	1 (0.8)		
	T4	34 (64.2)	38 (31.4)		
N.stage (%)	N0	43 (81.1)	118 (97.5)	0.001	
	N1	10 (18.9)	3 (2.5)		
M.stage (%)	M0	39 (73.6)	110 (90.9)	0.006	
OS (%)	M1	14 (26.4)	11 (9.1)		
	0	22 (41.5)	94 (77.7)	<0.001	
	1	31 (58.5)	27 (22.3)		
OS.time (median [IQR])		927.00 [374.00, 1491.00]	1315.00 [603.00, 2150.00]	0.017	nonnorm

### 3.6 Association between risk score and immune microenvironment

**Figure 6:**
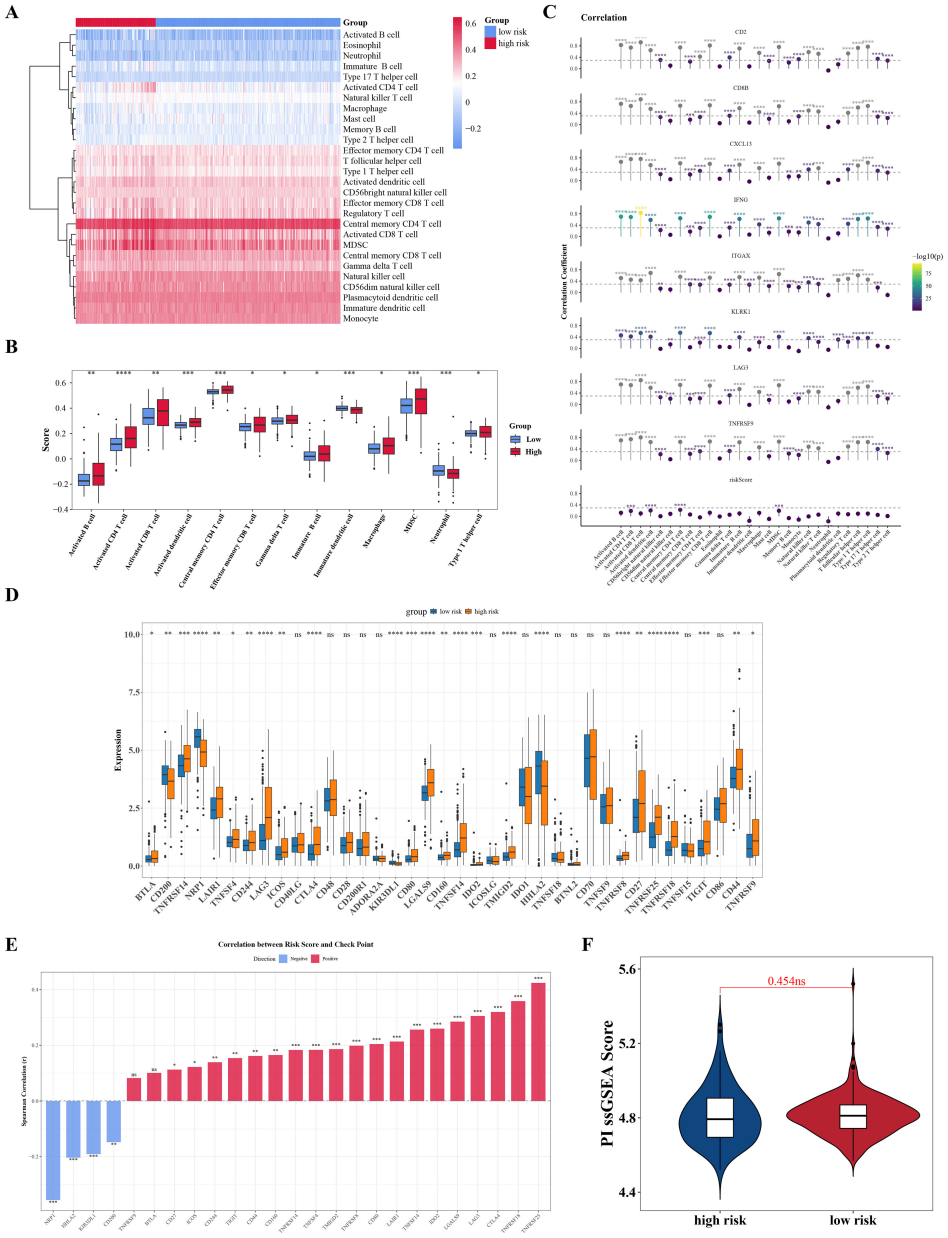
Immune microenvironment profiling of the risk model in the TCGA-KIRC training cohort. **(A)** Heatmap showing enrichment scores of 28 immune cell types. The high-risk group (red) exhibited higher enrichment of cells such as central memory CD4 T cells compared to the low-risk group (blue). **(B)** Boxplots of 13 immune cells with significant intergroup differences (p < 0.05; e.g., activated B cells, activated CD8 T cells). **(C)** Correlation analysis between risk score and differential immune cells (|cor| < 0.3, p > 0.05, not significant). **(D)** Correlation network between prognostic genes and immune cells (e.g., positive correlation between IFNG and activated CD8 T cells). **(E, F)** Differential expression of 25 immune checkpoints (p < 0.05); the high-risk group overexpressed suppressive checkpoints (e.g., NRP1, LAIR1, HHLA2), while the low-risk group overexpressed activating checkpoints (e.g., CD86, CD200). **(G)** GSVA inflammatory scores based on the MSigDB HALLMARK INFLAMMATORY RESPONSE gene set, showing no significant intergroup differences or correlation with risk score (p > 0.05). Statistical significance is indicated as: * p < 0.05; ** p < 0.01; *** p < 0.001; **** p < 0.0001; ns, not significant (p > 0.05).

Enrichment scores of 28 immune cells in the training cohort TCGA-KIRC were calculated (**Figure 6A**). The heatmap showed that the high-risk group (red) had significantly higher enrichment scores of immune cells such as Central memory CD4 T cell than the low-risk group (blue). Thirteen immune cells with significant differences between groups (p < 0.05) were defined as differential immune cells (e.g., Activated B cell, Activated CD4 T cell) (**Figure 6B**). No significant correlation was found between the risk score and differential immune cells (|cor| < 0.3, p > 0.05), but prognostic genes were correlated with immune cells (e.g., positive correlation between IFNG and Activated CD8 T cell, **Figure 6C**). Twenty-five immune checkpoints differed significantly between groups (p < 0.05) (**Figure 6D**): the high-risk group overexpressed suppressive checkpoints (e.g., NRP1, LAIR1, HHLA2), while the low-risk group overexpressed activating checkpoints (e.g., CD86, CD200), indicating stronger immunosuppression in the high-risk group. The strongest negative correlation was found between NRP1 and the risk score, and the strongest positive correlation with TNFRSF25 (**Figure 6E**). GSVA inflammatory scores based on the MSigDB HALLMARK INFLAMMATORY RESPONSE gene set showed no significant differences between groups (p > 0.05) or correlation with the risk score (**Figure 6F**).

### 3.7 Functional enrichment results of prognostic genes

**Figure 7:**
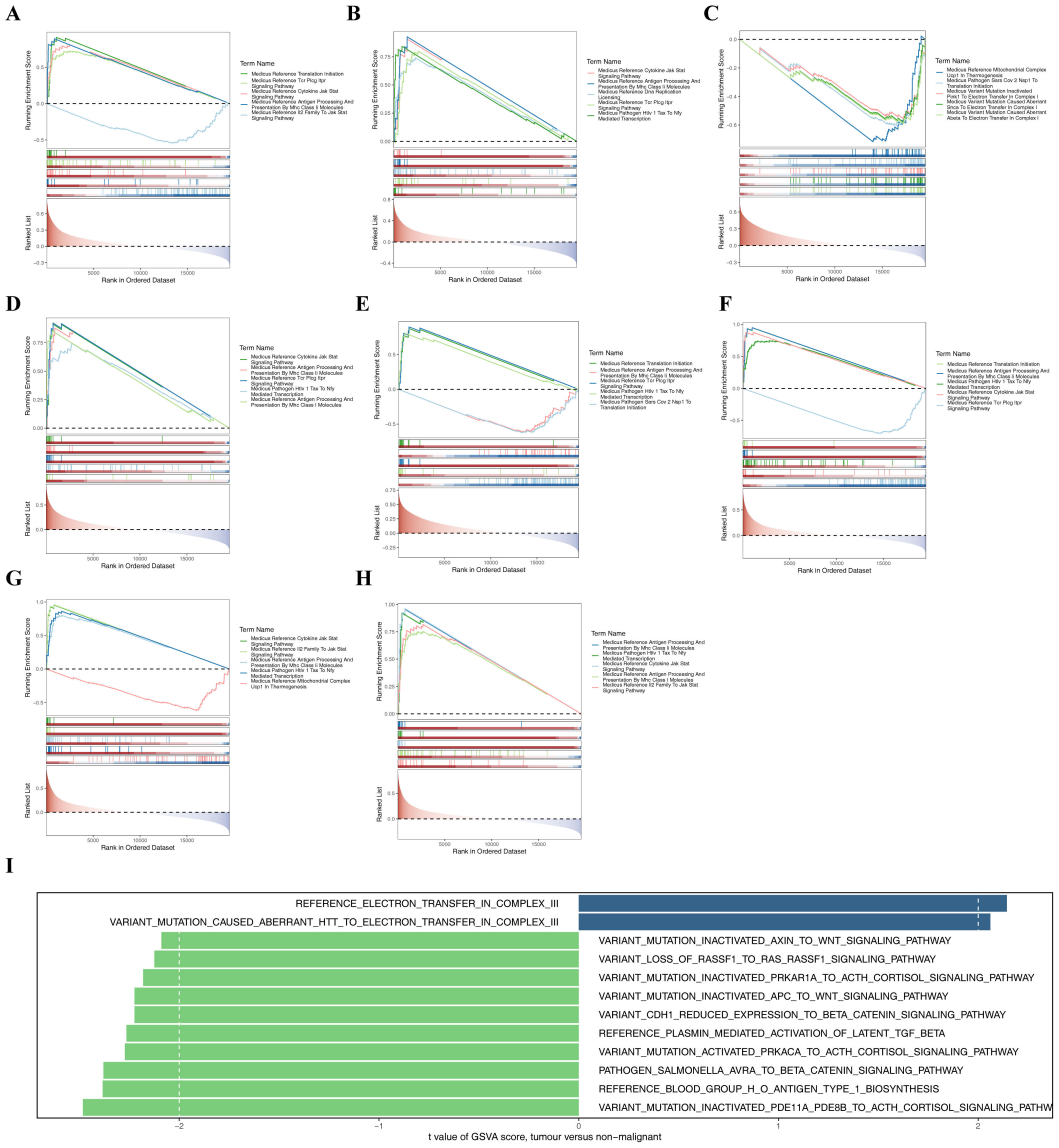
Functional enrichment and pathway analysis of prognostic genes in ccRCC. GSEA analysis of **(A)** IFNG pathways, **(B)** CXCL13 pathways, **(C)** KLRK1 pathways, **(D)** LAG3 pathways, **(E)** ITGAX pathways, **(F)** TNFRSF9 pathways, **(G)** CD2 pathways, **(H)** CD8B pathways, **(I)** pathway activation in high-risk and low-risk groups.

GSEA analysis investigated the potential functional involvement and pathways of eight prognostic genes in ccRCC of the training set (371 samples). Pathways were deemed significantly enriched at p < 0.05 (**Supplementary Table 4**). We discovered 88 distinct pathways significantly associated with IFNG, including involvement in translation initiation, and TCR PLCG ITPR signaling pathway (**Figure 7A**). A total of 56 pathways that were significantly enriched for CXCL13, like cytokine JAK-STAT signaling pathway (**Figure 7B**). Similarly, 44 pathways were implicated for KLRK1, such as mitochondrial complex UCP1 in thermogenesis, and SARS Cov 2 Nsp1 to translation initiation (**Figure 7C**). And identified 48 pathways that were significantly enriched for LAG3, such as cytokine JAK-STAT signaling pathway (**Figure 7D**). And for ITGAX, 17 pathways were highlighted, featuring translation initiation (**Figure 7E**). A total of 76 pathways were identified as significantly enriched for TNFRSF9, involving processes like translation initiation as well as MHC-II-mediated exogenous antigen handling and immune recognition (**Figure 7F**). Similarly, 59 pathways were associated with CD2, which included the cytokine JAK-STAT signaling pathway (**Figure 7G**) And for CD8B, 40 pathways stood out, like HTLV 1 Tax to NFY mediated transcription (**Figure 7H**). Notably, MHC-II antigen processing/presentation ranked among the top five significantly enriched pathways across the seven prognosis-associated genes, with the exception of KLRK1. Subsequently, the expression distribution of the core enriched genes in the significantly enriched pathways for the eight prognosis-related genes was depicted through ridge plots (**Supplementary Figure 3**). Finally, the GSVA indicated that in high-risk group, two pathways-electron transfer in complex III, and variant mutation-induced abnormal HTT to electron transfer contribution in complex III were upregulated. In contrast, in the low-risk group, ten pathways were prominently active, such as the blood group H-O antigen type 1 biosynthesis (**Figure 7I**). The GSVA highlighted the differential activation: the high-risk group involved in electron transfer in complex III and aberrant HTT contribution signaled possible metabolic or energy dysregulation linked to ccRCC progression. Conversely, the low-risk group showed enrichment of pathways like PDE11A-PDE8B inactivation and blood group H-O antigen synthesis, hinting at potential compensatory mechanisms and regulation of hormonal signaling. Targeting these pathways offers significant potential for the development of targeted therapies.

### 3.8 GeneMANIA analysis and chromosome localization results of prognostic genes

**Figure 8:**
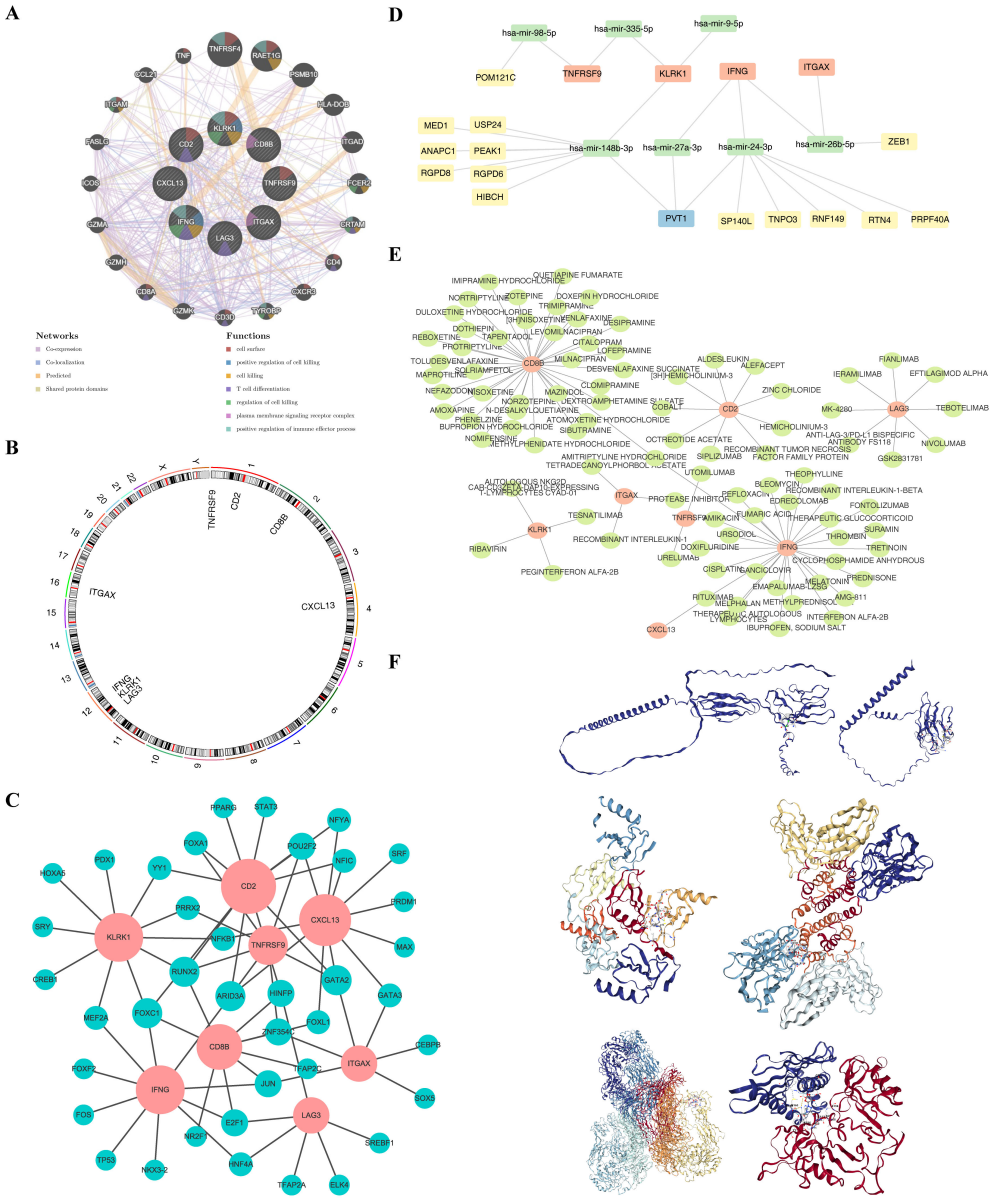
CcRCC prognostic gene interaction networks and molecular docking analysis. **(A)** GeneMANIA network analysis of prognostic genes. **(B)** Chromosomal localization of prognostic genes. **(C)** Prognostic gene transcription factor interaction network. **(D)** Comprehensive prognostic gene regulatory network. **(E)** Drug prediction and molecular docking of prognostic genes. **(F)** Molecular docking of prognostic genes with their targeted compounds.

The GeneMANIA analysis results showed TNFRSF4, RAET1G, PSMB10, HLA-DOB, ITGAD, FCER2, CRTAM, CD4, CXCR3, TYROBP, CD3D, GZMK, CD8A, GZMH, GZMA, ICOS, FASLG, ITGAM, CCL21, and TNF genes associated with prognostic genes, which were primarily involved in functions such as cell surface, positive regulation of cell killing, cell killing, and plasma membrane signaling receptor complex (**Figure 8A**). In chromosomal localization, the genomic locations of the prognosis-associated genes displayed that TNFRSF9 and CD2 were mapped to chromosome 1, CD8B to chromosome 2, CXCL13 to chromosome 4, IFNG, KLRK1, and LAG3 were located on chromosome 12, and ITGAX was positioned on chromosome 16 (**Figure 8B**). It suggests a potential role in distinct genomic regions related to immune regulation, which might underpin specific immune responses in ccRCC. Understanding the genomic context of these genes could help uncover epigenetic or structural variations that contribute to ccRCC susceptibility and response to therapy.

### 3.9 Establishment of prognostic genes interaction networks

Network analyst network predictions illustrated that CD2 predicted 12 TFs, CD8B predicted 9 TFs, CXCL13 predicted 11 TFs, IFNG predicted 10 TFs, ITGAX predicted 6 TFs, KLRK1 predicted 10 TFs, LAG3 predicted 6 TFs, and TNFRSF9 predicted 7 TFs. Among them, the transcription factor FOXC1, RUNX2, ARID3A, andGATA2 regulates four prognostic genes (**Figure 8C**). Furthermore, by taking the intersection of the findings from both databases, it was discovered that miRNAs targeting IFNG, KLRK1, ITGAX, and TNFRSF9 were identified (**Supplementary Table 5**). The miRNet database was employed to predict lncRNAs as potential targets of miRNAs. An overlap analysis was then conducted between the lncRNAs derived from this previous step and the key DE-LncRNAs. This analysis led to the identification of common lncRNA, PVT1, specifically in the miRNAs targeting IFNG and KLRK1 (**Supplementary Table 6**). Lastly, the ENCORI database study had demonstrated that a total of eight circRNAs were predicted to interact with miRNAs that were specifically connected to IFNG activity, while six circRNAs were implicated in the regulation of KLRK1 via miRNA interactions. Notably, a single circRNA was found to be associated with each miRNA in the cases of ITGAX and TNFRSF9, elucidating distinct regulatory patterns. (**Supplementary Table 7**). In light of these findings, a hypothetical prognostic gene regulatory network was constructed incorporating circRNAs, lncRNAs, miRNAs, and their associated mRNAs, which provides potential directions for future experimental validation of molecular interactions related to these gene targets (**Figure 8D**).

### 3.10 Drug prediction and molecular docking of prognostic genes

A potential drug prediction was conducted using the DGIdb database for prognosis-related genes in ccRCC treatment, where CD2 predicted nine targeted drugs, CXCL13 was linked to one. Notably, IFNG showed 28 targeted drug candidates, CD8B had 37, while KLRK1 suggested four potential targets. ITGAX was associated with three drugs, LAG3 had eight, and TNFRSF9 linked to only two targeted therapies (**Figure 8E**). Bioinformatic predictions from DGIdb suggested potential overlaps in linked compounds for some prognostic genes, focusing on repurposed drug candidate, already-approved medications being investigated for new therapeutic indications. Examples included: Amitriptyline hydrochloride (originally approved for depression) (28) linked to CD8B and IFNG; Rituximab (originally approved for lymphoma) (29) linked to CXCL13 and IFNG; Recombinant interleukin-1 (approved for inflammatory diseases) (30)and ribavirin (approved for viral infections) (31) as additional repurposed candidates. The overlapping drug targets between prognostic genes point to shared mechanisms that could be exploited for combined therapies or drug repurposing. Molecular docking was performed for prognostic genes and their active compound targets. The results showed that, CXCL13 interacted with rituximab yielded -5.5 kcal/mol, while CD8B interacted with n-desalkylqetiapine at -5.4 kcal/mol. The predicted binding affinity between IFNG and fumaric acid was -5.1 kcal/mol. ITGAX had a score of -7.2 kcal/mol when combined with recombinant interleukin-1. KLRK1 displayed a score of -6.8 kcal/mol with ribavirin (**Figure 8F**). Unfortunately, the three-dimensional structures of predicted drugs for LAG3 and TNFRSF9 were unavailable. Molecular docking results revealed the varying binding affinities between these genes and their predicted targets, providing insights into the molecular interactions that could be involved in disease mechanism and response.

### 3.11 Expression analysis of prognostic genes

**Figure 9:**
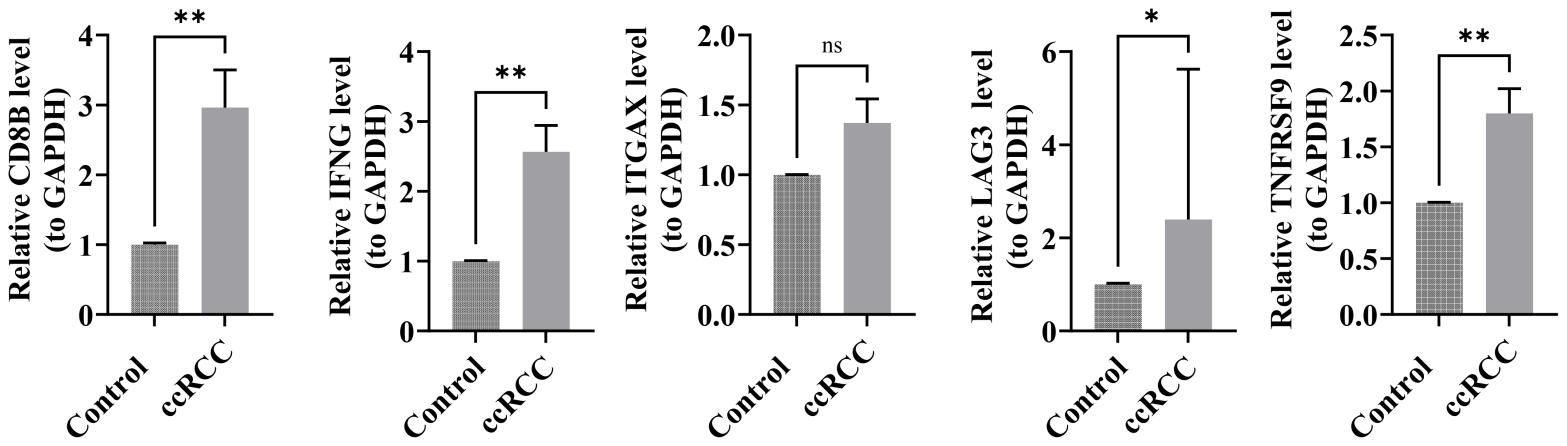
Analysis of prognostic gene expression in ccRCC **(A-E)**. ns: no significant; *p < 0,05; **p < 0.01.

The RT-qPCR results revealed that IFNG, TNFRSF9 and CD8B showed significantly elevated expression in ccRCC tumor tissue samples (p *<* 0.005). Unfortunately, the expression level of ITGAX and LAG3 were not significant difference between ccRCC tumor tissue samples and control paraneoplastic tissue samples (**Figure 9**).

## 4 Discussion

ccRCC represents a complex malignancy characterized by escalating genomic variability and tumor heterogeneity, coupled with pronounced immune infiltration and prognostic heterogeneity (3, 32). Reliable molecular biomarkers are essential for refining patient selection and predicting therapeutic response in ccRCC, as demonstrated by studies linking SPOP mutations to PD-L1 expression and CD8/TIL presence in prostate cancer (33), and PSMA PET/CT to accurate lymph node staging (34). The need for sophisticated risk stratification models is underscored by recent insights into ccRCC genomic heterogeneity (e.g., 3p loss, VHL/SETD2/PBRM1/BAP1 mutations) and its impact on immune microenvironment remodeling (8, 35). This investigation focuses on delineating prognostic gene risk models and exploring molecular regulatory mechanisms in ccRCC. Through bioinformatics analyses of whole-transcriptome data, we identified eight pivotal prognostic genes (IFNG, CXCL13, KLRK1, LAG3, ITGAX, TNFRSF9, CD2, and CD8B), subsequently selected five representative prognostic genes—IFNG, LAG3, ITGAX, TNFRSF9, and CD8B—for validation in a clinical cohort. Comprehensive functional analyses were systematically conducted on the eight prognostic genes to elucidate their mechanistic roles in ccRCC tumorigenesis and progression, thereby establishing a theoretical framework for advancing diagnostic biomarkers and therapeutic strategies in ccRCC management (12).

Building upon existing literature and our analytical findings, this study comprehensively discusses eight prognostic genes and their risk signature model (IFNG, CXCL13, KLRK1, LAG3, ITGAX, TNFRSF9, CD2, and CD8B). Notably, IFNG and KLRK1 demonstrated the most extensive pathway enrichment profiles and functional diversity, encompassing critical biological processes including positive regulation of cellular cytotoxicity, immune effector functions, and T cell differentiation. Significantly, IFNG and KLRK1 exhibited the most extensive interactome within molecular regulatory networks, demonstrating the highest degree of connectivity with TFs and non-coding RNAs. The IFNG gene product is a soluble signaling molecule of the type II interferon group. Secreted by both innate and adaptive immune cells, it plays critical roles in immune regulation (36). Regarding PD-L1 expression on tumor cells, two key evasion strategies are employed: innate immune resistance and adaptive immune resistance. The PD-1/PD-L1 checkpoint functions as a central mediator of immune suppression in the tumor microenvironment, predominantly linked to IFN-γ signaling pathways (37, 38). The findings of Chen et al. demonstrated that constitutive PD-L1 expression in RCC, synergistically regulated by cytokines such as IFN-γ and IL-1α through the STAT1 and NF-κB signaling pathways, significantly reinforces the tumor immunosuppressive microenvironment. These findings suggest that multi-pathway targeting may potentiate anti-PD-1 therapeutic efficacy (39). Emerging studies have revealed that in RCC, the FGFR signaling pathway attenuates antitumor immunity by suppressing IFNγ-mediated JAK/STAT signaling. Pharmacological targeting of FGFR (e.g., Lenvatinib) may restore IFNγ pathway activity, thereby synergizing with anti-PD-1 antibodies to amplify therapeutic efficacy (40). However, KLRK1 is a prognostically significant gene identified in this study. The functional variant rs1049174 SNP in the KLRK1 gene (encoding NKG2D) regulates NKG2D expression and modulates the immune surveillance pathway of NK cells, potentially influencing lymphoma susceptibility (41); however, its role remains undercharacterized in RCC research. Its precise mechanistic roles, regulatory networks, and clinical translatability have yet to be fully elucidated, positioning this receptor as a promising frontier for future RCC investigations.

The remaining six prognostic genes in our analysis also demonstrate significant research potential. Classified under the CXC chemokine family, CXCL13 functions as an antimicrobial peptide and chemoattractant. According to Dai et al., intratumoral infiltration of CXCL13+CD8+ T cells correlates with adverse clinical outcomes and immune evasion in ccRCC patients (42). Studies have further demonstrated that M2 macrophage-derived CXCL13 activates the Akt signaling pathway, driving key oncogenic processes: proliferation, motility, invasion, and epithelial-mesenchymal transition (EMT) in ccRCC. Elevated expression levels of CXCL13 are significantly correlated with diminished OS and DFS in clinical settings (43, 44). Within ccRCC tumors, DNA methylation of the lymphocyte-activation gene 3 (LAG3) shows significant associations with several key factors: spatial heterogeneity of LAG3 in malignant and immunocyte lineages, cytotoxic infiltrate densities within the tumor microenvironment, and ultimately, patient OS (45). And LAG3 may influence the effector function of CD4 T cells in cancer through the ATM-AMPKα regulatory axis (46).Notably, the observation of lower LAG3 levels in metastatic sites versus primary tumors indicates that site-specific expression may hold prognostic significance; however, its functional role in ccRCC pathogenesis remains poorly characterized (47, 48). Research indicates that within ccRCC tumor specimens, the promoters of ITGAX, LAPTM5, and SERPINE1 exhibit significantly reduced methylation relative to normal tissues (49). In the advanced ccRCC tumor immune microenvironment (TIME), the co-occurrence of CD8+ T cell exhaustion and M2 macrophage polarization has been well documented. This spatial association enables ligand-receptor-mediated cross-talk that drives both immunosuppressive phenotypes. This immune dysregulation pathway correlates with poorer prognosis in external datasets, suggesting potential immunosuppressive targets in ccRCC (35, 50). TNFRSF9+ CD8+ T cells are also enriched in ccRCC and serve as an adverse prognostic factor (51). TNFRSF9 may contribute to enhancing traditional T-cell and NK-cell immunity, thereby preventing tumor growth and viral infection (52). In contrast, CD8b serves as a hallmark marker of immune exhaustion, with its expression closely correlating with immune exhaustion (53). Existing literature recognizes CD2AP as a prognostic indicator in ccRCC, where its low expression and hypermethylation closely correlate with tumor progression and adverse outcomes; however, the mechanistic basis demands deeper investigation (54). Furthermore, the inactivation of CD2AP promotes the differentiation of CD4 T cells, thereby participating in functional regulation (55), highlighting its role in immune function.

Immunotherapy has markedly evolved in the treatment of advanced ccRCC, transitioning from interferon-α and high-dose IL-2 to targeted therapies against VEGF/VEGFR and mTOR pathways, and more recently to immune checkpoint inhibitors (ICIs) that restore antitumor immunity and improve outcomes even after prior targeted therapy failures (3, 56). It should be noted that the eight selected prognostic genes (IFNG, CXCL13, KLRK1, LAG3, ITGAX, TNFRSF9, CD2, CD8B) are all strongly immune-related, suggesting the risk model may primarily reflect tumor immune microenvironment infiltration rather than pure tumor-intrinsic biology. This aligns with the nature of ccRCC as an ‘immune-hot’ tumor, where progression highly depends on immune escape and microenvironment remodeling. Subsequent analyses showed that the high-risk group was enriched with more suppressive immune cells (e.g., M2 macrophages) and immune checkpoints (e.g., NRP1, LAIR1), while the low-risk group expressed higher levels of activating immune checkpoints (e.g., CD86), indicating the model captures a biologically meaningful immune contexture: the immunosuppressive microenvironment in the high-risk group promotes tumor progression, whereas the activated state in the low-risk group improves prognosis. This is not a limitation but highlights the model’s value in immune microenvironment stratification, offering a new perspective for predicting immunotherapy response in ccRCC. Our identification of CXCL13+CD8+ T cells as poor prognostic indicators—with elevated exhaustion markers, reduced effector molecules, and association with an immune-evasive microenvironment and TNFRSF9+CD8+ T cells as both exhausted/effector phenotypes linked to better immunotherapy response (42, 57), extends current understanding of T cell dysfunction in ccRCC.

In addition, whole-transcriptome analysis was employed in our study and appears to be a reliable approach. This method can reveal unique transcriptomic patterns in ccRCC cells, which aids in understanding the biological basis of cellular differentiation, development, and disease pathogenesis. Numerous RNAs with poorly characterized biological functions, including non-coding RNAs such as miRNAs, lncRNAs, and circRNAs, were identified through whole-transcriptome analysis as pivotal regulators steering gene expression programs, thereby governing cellular destiny (58).

In summary, we identified eight prognosis-associated genes in ccRCC through integrated bioinformatics analysis and developed an exploratory risk model that shows potential to predict patient survival outcomes, which still requires further validation in larger-scale cohorts. Our study further proposed hypothetical molecular interaction networks targeting these genes, including potential regulatory relationships with TFs, miRNAs, lncRNAs, and circRNAs, which require experimental validation. Bioinformatic analyses also suggested several compounds as potential candidates for further investigation, though their clinical relevance remains to be confirmed. However, a limitation is the use of cohort-specific optimal cutoffs for risk stratification rather than a unified predefined cutoff. This approach may inflate apparent model performance and reduce clinical generalizability, necessitating validation with a fixed cutoff in future large-scale studies. Necessitating validation in larger cohorts with additional clinical experiments and case studies to confirm the diagnostic performance of these prognostic genes. Additionally, the molecular mechanisms and potential therapeutic targets in ccRCC are multifaceted, and its progression constitutes a complex systemic process, warranting further investigation.

## Data Availability

The datasets presented in this study can be found in online repositories. The names of the repository/repositories and accession number(s) can be found in the article/**Supplementary Material**.

## Supplementary material

Supplementary Figure 1 Validation Plot of Proportional Hazards Assumption Test for the Prognostic Gene Risk Model.

Supplementary Figure 2 Results Plots of Risk Model Validation.

Supplementary Figure 3 GSEA Ridge Plots of Enriched Pathways for Prognostic Genes.

Supplementary Figure 4 The melt curve analysis figure for the experimental section.

Supplementary Table 1 RT-qPCR Primer Sequence Table.

Supplementary Table 2 GO Enrichment Analysis of Differentially Expressed mRNAs.

Supplementary Table 3 Results of KEGG Enrichment Analysis for Differentially Expressed mRNAs.

Supplementary Table 4 Results of Gene Set Enrichment Analysis for Prognostic Genes.

Supplementary Table 5 Database Intersection Analysis Table of miRNAs Targeting Key Prognostic Genes.

Supplementary Table 6 Intersection Analysis Table of lncRNAs in miRNA - Targeted Key Gene Pathways.

Supplementary Table 7 Prediction Analysis Table of circRNA - miRNA Interactions Associated with Prognostic Genes.
