# Supplementary figures and images for: Identification and validation of prognostic genes associated with clear cell renal cell carcinoma: based on public whole transcriptome sequencing datasets
Source: Front Oncol. 2026 Jul 8;16:1857894. doi: 10.3389/fonc.2026.1857894 (PMC13388226; doi:10.3389/fonc.2026.1857894)

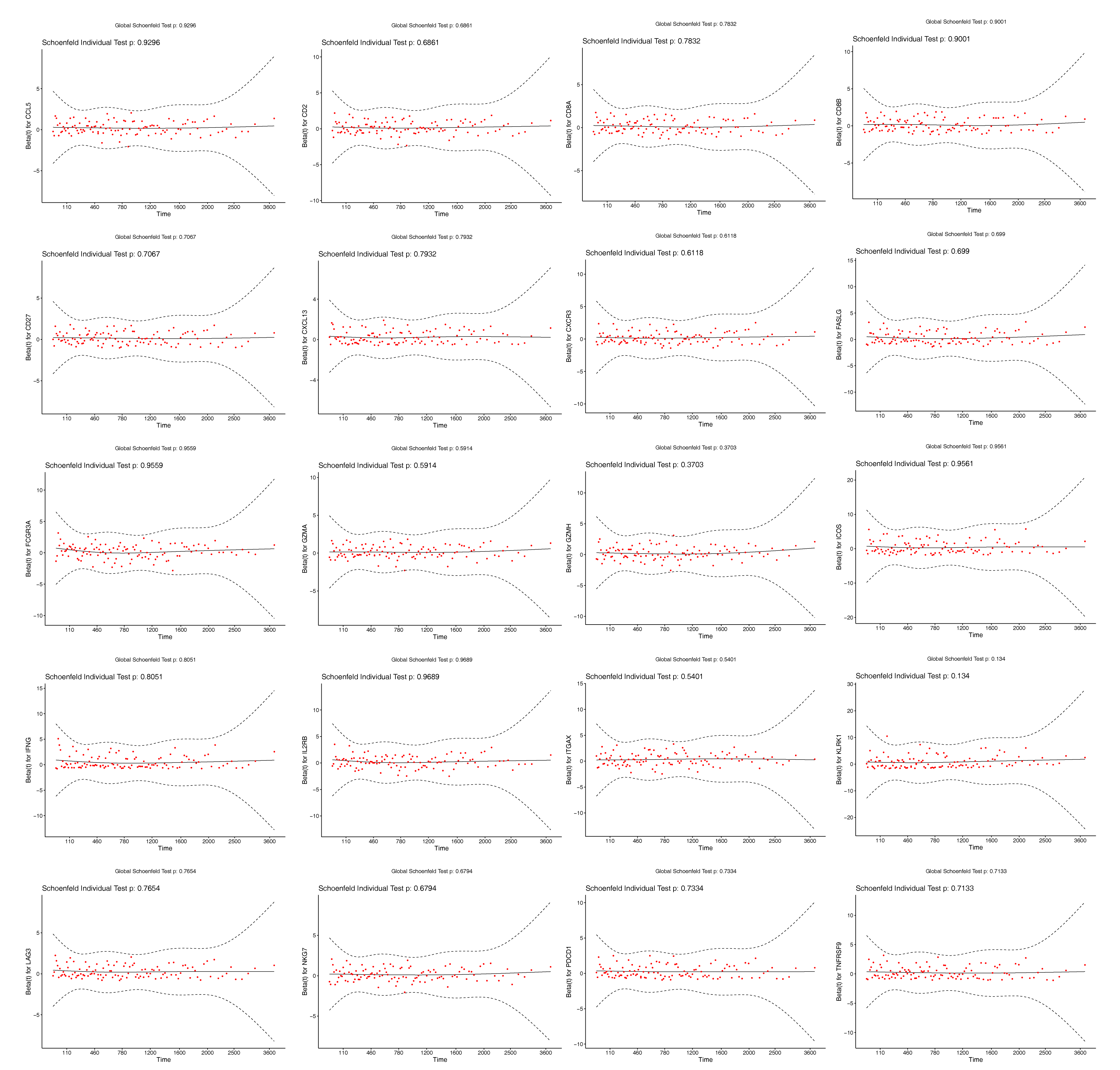

Supplement: Supplementary Figure 1 — Validation Plot of Proportional Hazards Assumption Test for the Prognostic Gene Risk Model. [file Image1.tif]

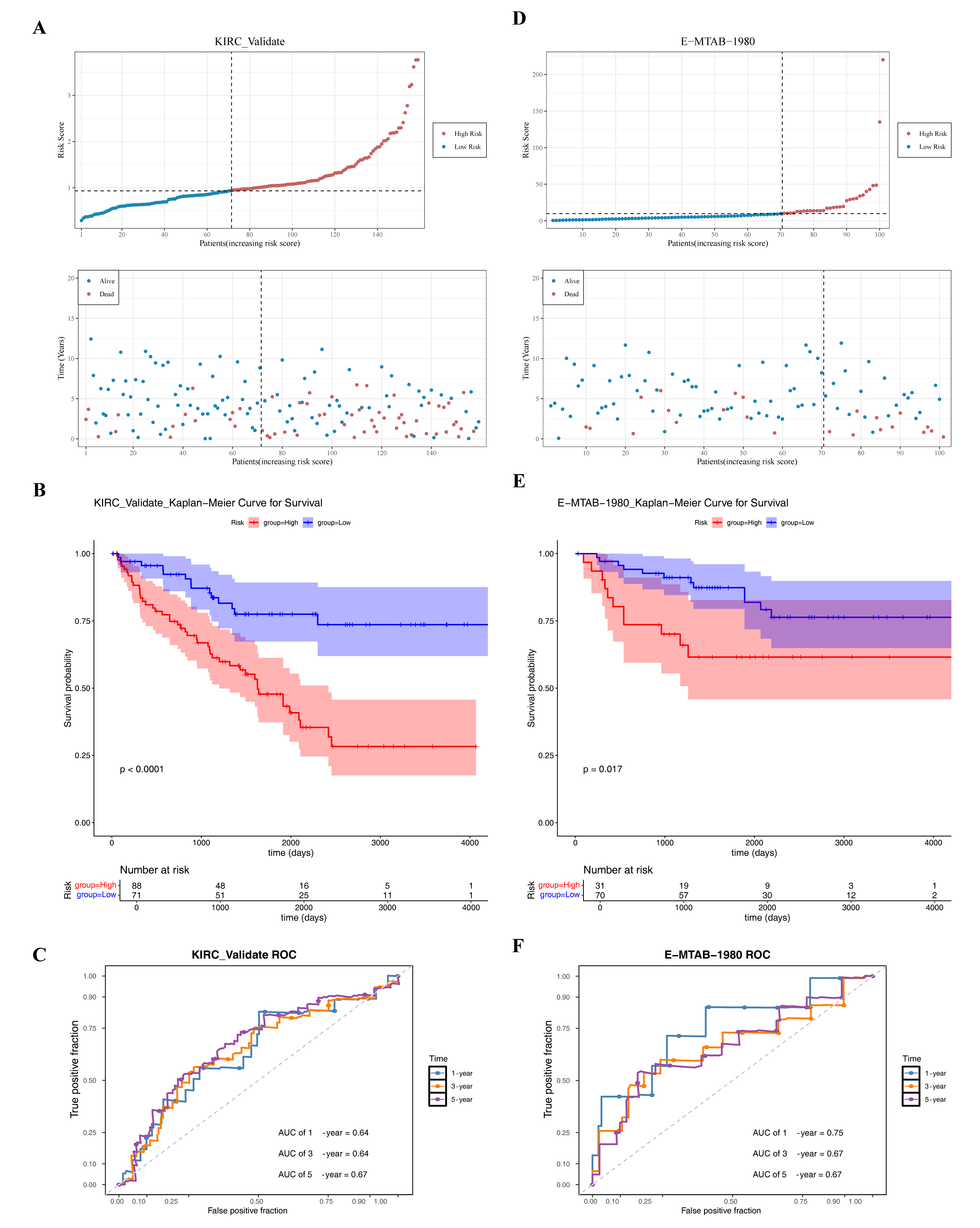

Supplement: Supplementary Figure 2 — Results Plots of Risk Model Validation. [file Image2.tif]

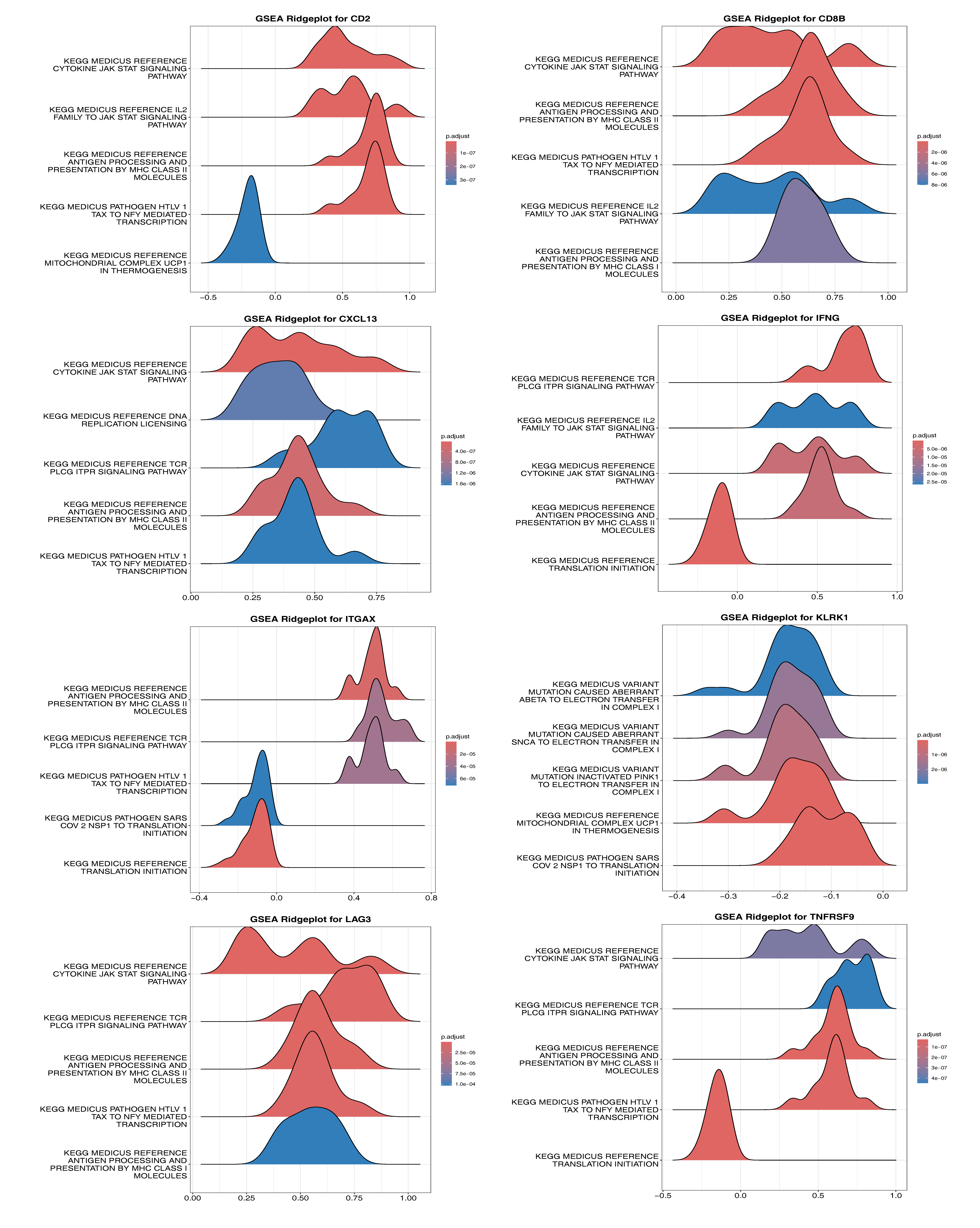

Supplement: Supplementary Figure 3 — GSEA Ridge Plots of Enriched Pathways for Prognostic Genes. [file Image3.tif]

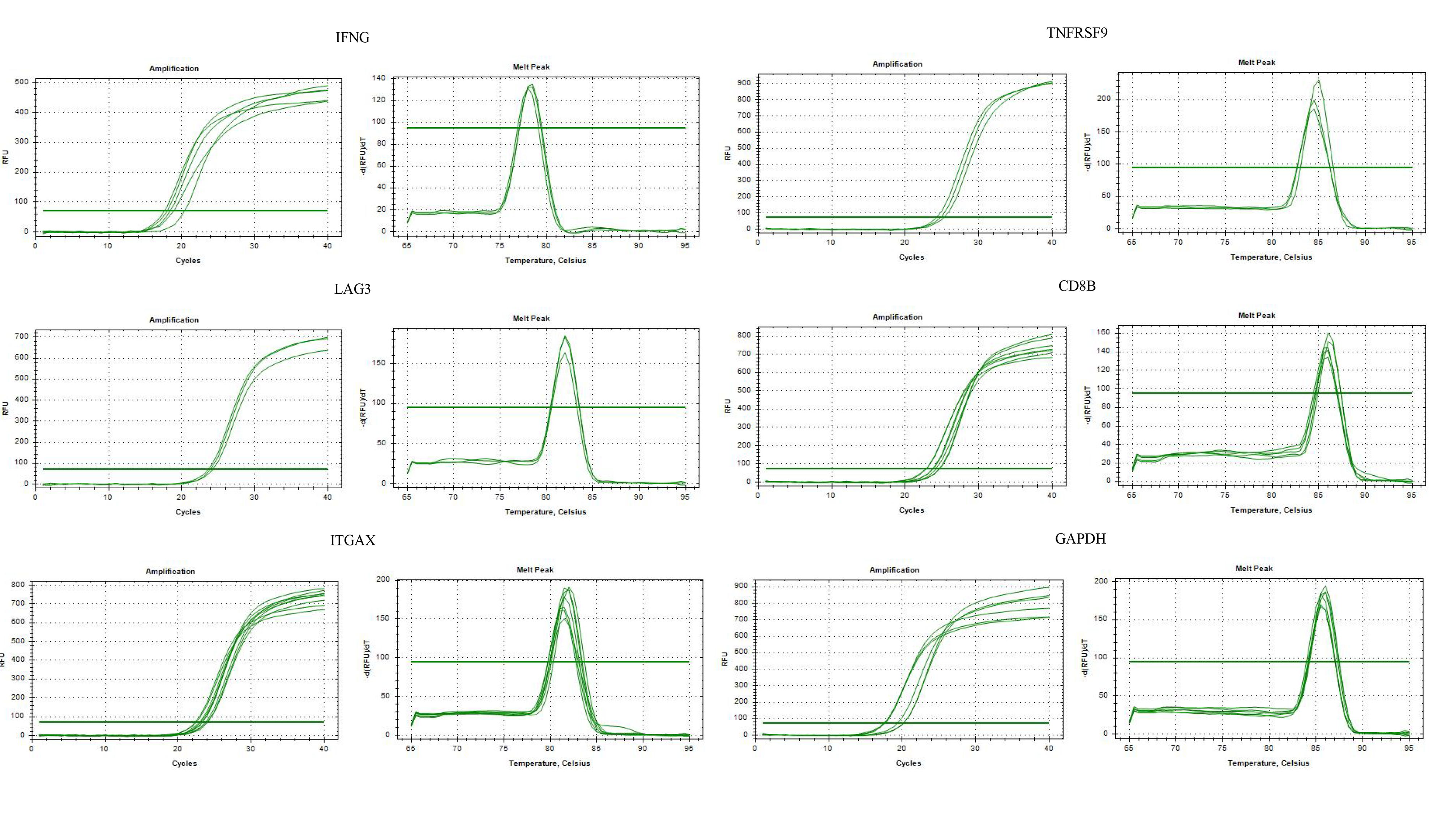

Supplement: Supplementary Figure 4 — The melt curve analysis figure for the experimental section. [file Image4.tif]
