# Supplementary material for: Identification and validation of prognostic genes associated with clear cell renal cell carcinoma: based on public whole transcriptome sequencing datasets
Source: Front Oncol. 2026 Jul 8;16:1857894. doi: 10.3389/fonc.2026.1857894 (PMC13388226; doi:10.3389/fonc.2026.1857894)
Supplement: Supplementary Table 1 — RT-qPCR Primer Sequence Table. [file Table1.docx]

Table S1. RT-qPCR Primer Sequence Table.

|  |  |
| --- | --- |
| **Primer** | **Sequence** |
| IFNG F | TGCAATCTGAGCCAGTGCTT |
| IFNG R | GCACCAGGCATGAAATCTCC |
| LAG3 F | TGACTGGAGACAATGGCGAC |
| LAG3 R | CGTTGGGCACCTGTGATGAT |
| ITGAX F | CACTCCTCCTGTTCACAGCC |
| ITGAX R | AGTTGGCATACTGGACCACG |
| TNFRSF9 F | AATGGGACGAAGGAGAGGGA |
| TNFRSF9 R | GAGAAACGGAGCGTGAGGAA |
| CD8B F | CAAGAAGAGAGTGTGCCGGT |
| CD8B R | CCTTCCCCTTGAGGCTGTTT |
| GAPDH F | ATGGGCAGCCGTTAGGAAAG |
| GAPDH R | AGGAAAAGCATCACCCGGAG |
