# Supplementary material for: Identification and validation of prognostic genes associated with clear cell renal cell carcinoma: based on public whole transcriptome sequencing datasets
Source: Front Oncol. 2026 Jul 8;16:1857894. doi: 10.3389/fonc.2026.1857894 (PMC13388226; doi:10.3389/fonc.2026.1857894)
Supplement: Supplementary Table 2 — GO Enrichment Analysis of Differentially Expressed mRNAs. [file Table2.docx]

Table S2. GO Enrichment Analysis of Differentially Expressed mRNAs.

|  | ONTOLOGY | ID | Description | GeneRatio | BgRatio | pvalue | p.adjust | qvalue | geneID | Count |
| --- | --- | --- | --- | --- | --- | --- | --- | --- | --- | --- |
| GO:0016324 | CC | GO:0016324 | apical plasma membrane | 48/743 | 403/19886 | 1.28E-12 | 5.36E-10 | 4.69E-10 | SLC16A3/ENPP3/SLC12A1/SLC4A11/ATP12A/SLC14A2/CLIC5/PTPRO/SLC7A8/SLC15A2/CASR/GP2/SLC5A2/TRPV5/AQP2/PTH1R/UMOD/SLC47A2/SLC22A8/ATP2B2/DPEP1/SLC26A4/SPTBN2/SLC17A4/SLC9A3/SLC9A4/SLC17A2/SLC5A11/MUC17/P2RX2/SLC34A3/CYP4F2/SLC34A1/SCNN1B/SLC13A2/SLC12A3/TF/MAL/PROM2/SCNN1G/PAPPA2/SLC7A13/SLC22A7/LHFPL5/ADCY8/S100G/SLC9A2/CEACAM20 | 48 |
| GO:0045177 | CC | GO:0045177 | apical part of cell | 52/743 | 469/19886 | 2.38E-12 | 5.36E-10 | 4.69E-10 | SLC16A3/ENPP3/SLC12A1/SLC4A11/ATP12A/SLC14A2/CLIC5/PTPRO/SLC7A8/SLC15A2/CASR/GP2/SLC5A2/TRPV5/AQP2/PTH1R/UMOD/SLC47A2/SLC22A8/ATP2B2/DPEP1/SLC26A4/SPTBN2/SLC17A4/SLC9A3/SLC9A4/SLC17A2/SLC5A11/CHL1/MUC17/P2RX2/SLC34A3/CYP4F2/SLC34A1/FABP1/SCNN1B/SLC13A2/SLC12A3/TF/MAL/EPB41L4B/PROM2/SCNN1G/PAPPA2/SLC7A13/SLC22A7/LHFPL5/ADCY8/S100G/SLC9A2/SLC4A9/CEACAM20 | 52 |
| GO:0006814 | BP | GO:0006814 | sodium ion transport | 34/695 | 248/18870 | 3.88E-11 | 1.78E-07 | 1.51E-07 | SLC6A3/SLC12A1/SLC4A11/WNK4/ATP12A/NOS1/CHP2/SLC5A7/NKAIN1/SLC5A2/UMOD/HECW1/SLC17A4/PKD2L1/SLC13A3/SLC9A3/SLC9A4/SLC17A2/SLC5A11/SLC34A3/SCN7A/SLC34A1/FXYD4/SCNN1B/SLC13A2/SLC12A3/CNTN1/SCN2A/SCNN1G/SCN10A/NKX2-5/SLC9A2/SLC4A9/CLCNKB | 34 |
| GO:0015081 | MF | GO:0015081 | sodium ion transmembrane transporter activity | 27/709 | 157/18496 | 5.27E-11 | 3.98E-08 | 3.39E-08 | GRIK3/SLC6A3/SLC12A1/SLC4A11/ATP12A/SLC5A7/SLC28A2/SLC5A2/SLC17A4/PKD2L1/SLC13A3/SLC9A3/SLC9A4/SLC17A2/SLC5A11/SLC34A3/SCN7A/SLC34A1/SCNN1B/SLC13A2/SLC12A3/SCN2A/SCNN1G/GRIK5/SCN10A/SLC9A2/SLC4A9 | 27 |
| GO:0005539 | MF | GO:0005539 | glycosaminoglycan binding | 33/709 | 240/18496 | 1.95E-10 | 7.37E-08 | 6.28E-08 | COL23A1/TREM2/VEGFA/TNFAIP6/COL5A3/FGF1/PGF/SFRP1/HRG/SOST/KNG1/CXCL10/CXCL11/HAPLN1/SERPINA5/CEL/F2/F11/CXCL13/APOB/LIPH/NELL1/ITIH1/PLA2G2D/PRG2/SAA1/PGLYRP2/FGF10/EPYC/REG1B/BMP7/REG3A/FGFBP1 | 33 |
| GO:0006959 | BP | GO:0006959 | humoral immune response | 33/695 | 258/18870 | 4.80E-10 | 8.11E-07 | 6.91E-07 | TREM2/GNLY/C3/HRG/CXCL9/CXCL10/CXCL11/IFNG/SUSD4/SEMG2/CST9/PDCD1/GATA3/F2/CXCL5/CXCL13/CD5L/KLK7/C7/TF/SEMG1/CR2/FOXJ1/LGALS4/REG1B/WFDC10B/KRT6A/FGA/IFNA14/KLK5/FGB/REG3A/MBL2 | 33 |
| GO:0035725 | BP | GO:0035725 | sodium ion transmembrane transport | 27/695 | 180/18870 | 5.32E-10 | 8.11E-07 | 6.91E-07 | SLC6A3/SLC12A1/SLC4A11/WNK4/ATP12A/NOS1/CHP2/SLC5A2/HECW1/SLC17A4/PKD2L1/SLC13A3/SLC9A3/SLC9A4/SLC17A2/SLC34A3/SCN7A/SLC34A1/FXYD4/SCNN1B/SLC13A2/SLC12A3/SCN2A/SCNN1G/SCN10A/SLC9A2/SLC4A9 | 27 |
| GO:0015291 | MF | GO:0015291 | secondary active transmembrane transporter activity | 35/709 | 286/18496 | 1.37E-09 | 3.45E-07 | 2.94E-07 | SLC16A3/SLC6A3/SLC12A1/SLC4A11/SLC16A5/SLC7A8/SLC15A2/SLC5A7/SLC28A2/SLC5A2/SLC17A9/SLC47A2/SLC22A8/SLC26A4/SLC17A4/SLC2A12/SLC13A3/SLC9A3/SLC9A4/SLC17A2/SLC5A11/SLC34A3/SLC18A3/SLC34A1/SLC13A2/SLC12A3/SLC36A2/SLC30A2/SLC7A13/SLC30A8/SLC22A7/SLC9A2/SLC4A9/SLC4A1/SLC30A10 | 35 |
| GO:0046873 | MF | GO:0046873 | metal ion transmembrane transporter activity | 45/709 | 441/18496 | 2.37E-09 | 4.48E-07 | 3.81E-07 | GRIK3/KCNJ10/SLC6A3/SLC12A1/SLC4A11/CLDN16/TRPV6/ATP12A/KCNJ1/TRPA1/KCNK9/KCNN1/SLC5A7/SLC28A2/SLC5A2/TRPV5/KCNK10/NIPAL1/ATP2B2/SLC17A4/PKD2L1/SLC13A3/SLC9A3/SLC9A4/SLC17A2/SLC5A11/SLC34A3/SCN7A/SLC34A1/FXYD4/SCNN1B/SLC13A2/KCNK17/SLC12A3/SCN2A/SCNN1G/GRIK5/SLC30A2/SLC30A8/SCN10A/SLC9A2/SLC4A9/SLC30A10/CACNG2/KCNU1 | 45 |
| GO:0072073 | BP | GO:0072073 | kidney epithelium development | 23/695 | 150/18870 | 6.59E-09 | 7.31E-06 | 6.22E-06 | VEGFA/CALB1/IRX2/FGF1/WNK4/SFRP1/TCF21/WNT9B/PTPRO/NPHS1/IRX1/UMOD/GPC3/TFAP2B/HES5/GATA3/NPHS2/TACSTD2/WNT7B/FOXJ1/SIM1/LHX1/BMP7 | 23 |
| GO:0001656 | BP | GO:0001656 | metanephros development | 18/695 | 94/18870 | 8.19E-09 | 7.31E-06 | 6.22E-06 | CALB1/IRX2/TCF21/WNT9B/IRX1/AQP2/GDF6/UMOD/GPC3/TFAP2B/HES5/GATA3/NPHS2/WNT7B/FOXJ1/FGF10/LHX1/BMP7 | 18 |
| GO:0002526 | BP | GO:0002526 | acute inflammatory response | 19/695 | 107/18870 | 1.17E-08 | 7.31E-06 | 6.22E-06 | MYLK3/FCGR3A/IL20RB/FCGR1A/C3/TFR2/F2/HP/EPO/PLA2G2D/CRP/SAA1/SAA2/ORM2/LBP/ORM1/SAA4/REG3A/MBL2 | 19 |
| GO:0006953 | BP | GO:0006953 | acute-phase response | 13/695 | 48/18870 | 1.20E-08 | 7.31E-06 | 6.22E-06 | TFR2/F2/HP/EPO/CRP/SAA1/SAA2/ORM2/LBP/ORM1/SAA4/REG3A/MBL2 | 13 |
| GO:0008201 | MF | GO:0008201 | heparin binding | 25/709 | 174/18496 | 1.27E-08 | 1.92E-06 | 1.63E-06 | COL23A1/VEGFA/COL5A3/FGF1/PGF/SFRP1/HRG/SOST/KNG1/CXCL10/CXCL11/SERPINA5/CEL/F2/F11/CXCL13/APOB/LIPH/NELL1/PLA2G2D/PRG2/SAA1/FGF10/BMP7/FGFBP1 | 25 |
| GO:0072006 | BP | GO:0072006 | nephron development | 23/695 | 156/18870 | 1.42E-08 | 7.31E-06 | 6.22E-06 | VEGFA/CALB1/IRX2/FGF1/WNK4/ANGPT2/TCF21/WNT9B/PTPRO/NPHS1/IRX1/UMOD/GPC3/TFAP2B/HES5/GATA3/NPHS2/TACSTD2/ERBB4/WNT7B/FOXJ1/LHX1/BMP7 | 23 |
| GO:0072009 | BP | GO:0072009 | nephron epithelium development | 20/695 | 120/18870 | 1.49E-08 | 7.31E-06 | 6.22E-06 | VEGFA/CALB1/IRX2/FGF1/WNK4/TCF21/WNT9B/PTPRO/NPHS1/IRX1/UMOD/GPC3/TFAP2B/HES5/GATA3/NPHS2/TACSTD2/WNT7B/FOXJ1/LHX1 | 20 |
| GO:0060993 | BP | GO:0060993 | kidney morphogenesis | 18/695 | 98/18870 | 1.63E-08 | 7.31E-06 | 6.22E-06 | VEGFA/CALB1/IRX2/FGF1/WNK4/TCF21/WNT9B/IRX1/GPC3/HES5/GATA3/TACSTD2/ERBB4/WNT7B/FOXJ1/FGF10/LHX1/BMP7 | 18 |
| GO:0019730 | BP | GO:0019730 | antimicrobial humoral response | 21/695 | 133/18870 | 1.76E-08 | 7.31E-06 | 6.22E-06 | GNLY/HRG/CXCL9/CXCL10/CXCL11/SEMG2/CST9/F2/CXCL5/CXCL13/KLK7/TF/SEMG1/LGALS4/REG1B/WFDC10B/KRT6A/FGA/KLK5/FGB/REG3A | 21 |
| GO:0001822 | BP | GO:0001822 | kidney development | 34/695 | 314/18870 | 2.00E-08 | 7.61E-06 | 6.49E-06 | VEGFA/CALB1/IRX2/SOX11/FGF1/WNK4/ANGPT2/SFRP1/TCF21/WNT9B/PROX1/PTPRO/NPHS1/IRX1/AQP2/GDF6/UMOD/TP73/GPC3/TFAP2B/STRA6/MMP9/HES5/GATA3/NPHS2/TACSTD2/ERBB4/SLC34A1/WNT7B/FOXJ1/FGF10/SIM1/LHX1/BMP7 | 34 |
| GO:0009897 | CC | GO:0009897 | external side of plasma membrane | 39/743 | 387/19886 | 2.05E-08 | 3.08E-06 | 2.69E-06 | ENPP3/ITGAX/TNFRSF4/FCGR3A/ITGAD/IL2RB/FCGR1A/FASLG/TNFRSF9/GP2/HLA-G/CD8A/CD2/CXCL9/CXCL10/CXCR3/SERPINA5/LAG3/CD27/TFR2/KLRK1/PDCD1/F2/CLEC12B/FGG/HHLA2/MUC17/MUC16/SCNN1B/PRND/SCNN1G/VTCN1/FOLR3/FGA/PLG/CHRNA4/FGB/MOG/MBL2 | 39 |
| GO:0062023 | CC | GO:0062023 | collagen-containing extracellular matrix | 41/743 | 429/19886 | 3.98E-08 | 4.48E-06 | 3.92E-06 | COL23A1/ANGPTL4/COL5A3/GPC5/ANGPT2/VWF/SFRP1/HRG/SOST/LOX/KNG1/COL4A6/ADAMDEC1/HAPLN1/SERPINA5/GPC3/TGFBI/COL26A1/MMP9/F2/CTHRC1/ZP1/FIBCD1/FGG/ADAMTS20/MUC17/ITIH1/INHBE/FREM1/PRG2/FGF10/L1CAM/LGALS4/ORM2/FGA/ORM1/BMP7/AMELY/PLG/FGB/APOA4 | 41 |
| GO:0072001 | BP | GO:0072001 | renal system development | 34/695 | 324/18870 | 4.35E-08 | 1.53E-05 | 1.30E-05 | VEGFA/CALB1/IRX2/SOX11/FGF1/WNK4/ANGPT2/SFRP1/TCF21/WNT9B/PROX1/PTPRO/NPHS1/IRX1/AQP2/GDF6/UMOD/TP73/GPC3/TFAP2B/STRA6/MMP9/HES5/GATA3/NPHS2/TACSTD2/ERBB4/SLC34A1/WNT7B/FOXJ1/FGF10/SIM1/LHX1/BMP7 | 34 |
| GO:0001906 | BP | GO:0001906 | cell killing | 27/695 | 223/18870 | 5.87E-08 | 1.92E-05 | 1.63E-05 | FCGR3A/PIK3R6/NKG7/FCGR1A/GNLY/C3/HRG/GZMH/GZMA/GBP5/HLA-G/CD2/CRTAM/IFNG/LAG3/KLRK1/HAMP/F2/CD5L/CLEC12B/C7/SCNN1B/SEMG1/KRT6A/KLRF2/DEFB132/MBL2 | 27 |
| GO:0022853 | MF | GO:0022853 | active monoatomic ion transmembrane transporter activity | 29/709 | 244/18496 | 6.78E-08 | 8.54E-06 | 7.27E-06 | SLC16A3/SLC6A3/SLC12A1/ATP12A/SLC15A2/SLC5A7/SLC28A2/SLC5A2/SLC47A2/ATP2B2/SLC26A4/SLC17A4/SLC13A3/SLC9A3/SLC9A4/SLC17A2/SLC5A11/SLC34A3/SLC18A3/SLC34A1/SLC13A2/SLC12A3/SLC36A2/SLC30A2/SLC30A8/SLC9A2/SLC4A9/SLC4A1/SLC30A10 | 29 |
| GO:0072562 | CC | GO:0072562 | blood microparticle | 21/743 | 144/19886 | 9.37E-08 | 8.43E-06 | 7.38E-06 | ANGPTL4/HSPA2/C3/HRG/CP/KNG1/ALB/AFM/F2/CD5L/HP/FGG/ITIH1/TF/ORM2/FGA/ORM1/PLG/SLC4A1/FGB/APOA4 | 21 |
| GO:0141060 | BP | GO:0141060 | disruption of anatomical structure in another organism | 17/695 | 99/18870 | 1.14E-07 | 3.48E-05 | 2.97E-05 | NKG7/GNLY/HRG/GZMH/GZMA/GBP5/IFNG/HAMP/F2/C7/SCNN1B/SEMG1/REG1B/KRT6A/REG3A/DEFB132/MBL2 | 17 |
| GO:0045165 | BP | GO:0045165 | cell fate commitment | 30/695 | 286/18870 | 2.73E-07 | 7.80E-05 | 6.65E-05 | WNT8B/POU5F1/SFRP1/WNT9B/PROX1/EOMES/BATF/DLX1/TBX15/RAG2/HES5/GATA3/DLX2/TBX5/ERBB4/NKX6-2/NR2E1/WNT7B/SPDEF/FGF10/PITX1/ISL2/POU4F1/NKX2-5/ARX/DMRT3/ESRP1/MYOD1/SOX1/GATA4 | 30 |
| GO:0061844 | BP | GO:0061844 | antimicrobial humoral immune response mediated by antimicrobial peptide | 15/695 | 83/18870 | 3.18E-07 | 8.55E-05 | 7.28E-05 | GNLY/HRG/CXCL9/CXCL10/CXCL11/F2/CXCL5/CXCL13/KLK7/SEMG1/LGALS4/REG1B/KRT6A/KLK5/REG3A | 15 |
| GO:0005125 | MF | GO:0005125 | cytokine activity | 27/709 | 237/18496 | 4.51E-07 | 4.88E-05 | 4.15E-05 | VEGFA/CD70/INHBB/WNT8B/TNFSF9/WNT9B/CCL18/FASLG/CCL5/TNFSF14/CXCL9/CXCL10/CXCL11/GDF6/IFNG/SCG2/FAM3B/CXCL5/CXCL13/CCL20/EPO/INHBE/WNT7B/IL11/CCL25/BMP7/IFNA14 | 27 |
| GO:0042742 | BP | GO:0042742 | defense response to bacterium | 32/695 | 330/18870 | 6.49E-07 | 0.000153631 | 0.000130895 | TREM2/FCGR1A/GNLY/GBP5/AICDA/UMOD/KLRK1/HAMP/RAG2/SEMG2/F2/CXCL13/HP/CCL20/KLK7/SCNN1B/TF/SEMG1/CRP/PRG2/PGLYRP2/GSDMC/LGALS4/WFDC10B/KRT6A/LBP/FGA/KLK5/FGB/DEFB132/MBL2/DEFB125 | 32 |
| GO:0098659 | BP | GO:0098659 | inorganic cation import across plasma membrane | 19/695 | 137/18870 | 6.72E-07 | 0.000153631 | 0.000130895 | KCNJ10/SLC12A1/TRPV6/ATP12A/KCNJ1/KCNK9/SLC5A2/TRPV5/IFNG/SLC9A3/SLC9A4/SCN7A/SLC34A1/SCNN1B/SLC12A3/SCN2A/SCNN1G/SCN10A/SLC9A2 | 19 |
| GO:0099587 | BP | GO:0099587 | inorganic ion import across plasma membrane | 19/695 | 137/18870 | 6.72E-07 | 0.000153631 | 0.000130895 | KCNJ10/SLC12A1/TRPV6/ATP12A/KCNJ1/KCNK9/SLC5A2/TRPV5/IFNG/SLC9A3/SLC9A4/SCN7A/SLC34A1/SCNN1B/SLC12A3/SCN2A/SCNN1G/SCN10A/SLC9A2 | 19 |
| GO:0072080 | BP | GO:0072080 | nephron tubule development | 16/695 | 100/18870 | 7.28E-07 | 0.000158451 | 0.000135002 | VEGFA/CALB1/IRX2/FGF1/WNK4/TCF21/WNT9B/IRX1/UMOD/GPC3/TFAP2B/HES5/GATA3/TACSTD2/WNT7B/LHX1 | 16 |
| GO:1901681 | MF | GO:1901681 | sulfur compound binding | 29/709 | 275/18496 | 8.66E-07 | 7.58E-05 | 6.46E-05 | COL23A1/VEGFA/COL5A3/FGF1/PGF/SFRP1/HRG/SOST/KNG1/CXCL10/CXCL11/SERPINA5/CEL/F2/F11/CXCL13/APOB/LIPH/NELL1/GAL3ST3/TF/PLA2G2D/PRG2/SAA1/FGF10/HS3ST5/BMP7/FGFBP1/SULT2A1 | 29 |
| GO:0004252 | MF | GO:0004252 | serine-type endopeptidase activity | 22/709 | 174/18496 | 9.02E-07 | 7.58E-05 | 6.46E-05 | HTRA4/GZMH/GZMA/PRSS53/GZMK/MMP9/F2/PROZ/F11/CD5L/HP/OVCH2/KLK7/KLK6/TMPRSS4/TMPRSS2/MMP13/PRSS22/TMPRSS11A/PLG/KLK5/KLK4 | 22 |
| GO:0015293 | MF | GO:0015293 | symporter activity | 20/709 | 149/18496 | 1.09E-06 | 8.22E-05 | 7.00E-05 | SLC16A3/SLC6A3/SLC12A1/SLC4A11/SLC16A5/SLC15A2/SLC5A7/SLC28A2/SLC5A2/SLC17A4/SLC2A12/SLC13A3/SLC17A2/SLC5A11/SLC34A3/SLC34A1/SLC13A2/SLC12A3/SLC36A2/SLC4A9 | 20 |
| GO:0061326 | BP | GO:0061326 | renal tubule development | 16/695 | 104/18870 | 1.25E-06 | 0.000239839 | 0.000204345 | VEGFA/CALB1/IRX2/FGF1/WNK4/TCF21/WNT9B/IRX1/UMOD/GPC3/TFAP2B/HES5/GATA3/TACSTD2/WNT7B/LHX1 | 16 |
| GO:0031640 | BP | GO:0031640 | killing of cells of another organism | 15/695 | 92/18870 | 1.26E-06 | 0.000239839 | 0.000204345 | NKG7/GNLY/HRG/GZMH/GZMA/GBP5/IFNG/HAMP/F2/C7/SCNN1B/SEMG1/KRT6A/DEFB132/MBL2 | 15 |
| GO:0141061 | BP | GO:0141061 | disruption of cell in another organism | 15/695 | 92/18870 | 1.26E-06 | 0.000239839 | 0.000204345 | NKG7/GNLY/HRG/GZMH/GZMA/GBP5/IFNG/HAMP/F2/C7/SCNN1B/SEMG1/KRT6A/DEFB132/MBL2 | 15 |
| GO:0008236 | MF | GO:0008236 | serine-type peptidase activity | 23/709 | 192/18496 | 1.33E-06 | 9.16E-05 | 7.81E-05 | HTRA4/GZMH/GZMA/PRSS53/GZMK/MMP9/F2/PROZ/F11/CD5L/HP/OVCH2/KLK7/KLK6/TMPRSS4/DPP6/TMPRSS2/MMP13/PRSS22/TMPRSS11A/PLG/KLK5/KLK4 | 23 |
| GO:0072028 | BP | GO:0072028 | nephron morphogenesis | 14/695 | 81/18870 | 1.38E-06 | 0.000251758 | 0.0002145 | VEGFA/IRX2/FGF1/WNK4/TCF21/WNT9B/IRX1/GPC3/HES5/GATA3/TACSTD2/ERBB4/LHX1/BMP7 | 14 |
| GO:0010038 | BP | GO:0010038 | response to metal ion | 33/695 | 359/18870 | 1.44E-06 | 0.000253542 | 0.00021602 | KCNJ10/SLC6A3/INHBB/TRPV6/CPNE6/ABAT/CRHBP/CHP2/CASR/AQP2/TNNT2/TFR2/HAMP/DPEP1/MMP9/ITPKA/MT1G/TNNC1/FGG/TERT/SYT7/SLC34A1/SLC13A2/TF/MT1H/SLC30A2/MT3/SLC30A8/ADCY8/FGA/FGB/SLC30A10/CACNG2 | 33 |
| GO:0015370 | MF | GO:0015370 | solute:sodium symporter activity | 14/709 | 80/18496 | 1.88E-06 | 0.000111025 | 9.46E-05 | SLC6A3/SLC12A1/SLC5A7/SLC28A2/SLC5A2/SLC17A4/SLC13A3/SLC17A2/SLC5A11/SLC34A3/SLC34A1/SLC13A2/SLC12A3/SLC4A9 | 14 |
| GO:0017171 | MF | GO:0017171 | serine hydrolase activity | 23/709 | 196/18496 | 1.91E-06 | 0.000111025 | 9.46E-05 | HTRA4/GZMH/GZMA/PRSS53/GZMK/MMP9/F2/PROZ/F11/CD5L/HP/OVCH2/KLK7/KLK6/TMPRSS4/DPP6/TMPRSS2/MMP13/PRSS22/TMPRSS11A/PLG/KLK5/KLK4 | 23 |
| GO:0015294 | MF | GO:0015294 | solute:monoatomic cation symporter activity | 17/709 | 117/18496 | 2.29E-06 | 0.000119639 | 0.000101948 | SLC16A3/SLC6A3/SLC12A1/SLC15A2/SLC5A7/SLC28A2/SLC5A2/SLC17A4/SLC13A3/SLC17A2/SLC5A11/SLC34A3/SLC34A1/SLC13A2/SLC12A3/SLC36A2/SLC4A9 | 17 |
| GO:0022804 | MF | GO:0022804 | active transmembrane transporter activity | 39/709 | 454/18496 | 2.37E-06 | 0.000119639 | 0.000101948 | SLC16A3/SLC6A3/SLC12A1/SLC4A11/ATP12A/SLC16A5/ABCA4/SLC7A8/SLC15A2/SLC5A7/SLC28A2/SLC5A2/SLC17A9/SLC47A2/SLC22A8/ATP2B2/SLC26A4/SLC17A4/SLC2A12/SLC13A3/SLC9A3/SLC9A4/SLC17A2/SLC5A11/SLC34A3/SLC18A3/SLC34A1/ABCA13/SLC13A2/SLC12A3/SLC36A2/SLC30A2/SLC7A13/SLC30A8/SLC22A7/SLC9A2/SLC4A9/SLC4A1/SLC30A10 | 39 |
| GO:0016323 | CC | GO:0016323 | basolateral plasma membrane | 26/743 | 249/19886 | 2.43E-06 | 0.000174329 | 0.000152512 | CA9/SLC16A3/KCNJ10/SLC4A11/ATP12A/VSIG1/CLDN19/SLC7A8/CASR/AQP2/PTH1R/UMOD/SLC22A8/ATP2B2/SLC13A3/SLC9A4/ERBB4/AQP9/PROM2/SLC22A7/ADCY8/S100G/SLC4A9/SLC4A1/CLDN8/BSND | 26 |
| GO:0009925 | CC | GO:0009925 | basal plasma membrane | 28/743 | 282/19886 | 2.71E-06 | 0.000174329 | 0.000152512 | CA9/SLC16A3/KCNJ10/SLC4A11/ATP12A/VSIG1/CLDN19/SLC7A8/CASR/AQP2/PTH1R/UMOD/SLC22A8/ATP2B2/SLC13A3/SLC9A4/TACSTD2/ERBB4/AQP9/TF/PROM2/SLC22A7/ADCY8/S100G/SLC4A9/SLC4A1/CLDN8/BSND | 28 |
| GO:0004175 | MF | GO:0004175 | endopeptidase activity | 35/709 | 389/18496 | 2.79E-06 | 0.000131988 | 0.000112471 | HTRA4/SFRP1/ADAM18/GZMH/GZMA/CAPN12/CTSW/PRSS53/CAPN11/ADAMDEC1/PAPPA/CASP5/GZMK/ADAMTS14/MMP9/F2/PROZ/F11/CD5L/HP/OVCH2/KLK7/ADAMTS20/MEP1A/KLK6/TMPRSS4/ADAMTS19/PAPPA2/TMPRSS2/MMP13/PRSS22/TMPRSS11A/PLG/KLK5/KLK4 | 35 |
| GO:1905330 | BP | GO:1905330 | regulation of morphogenesis of an epithelium | 12/695 | 65/18870 | 3.81E-06 | 0.000644367 | 0.000549007 | VEGFA/FGF1/ITGAX/EGF/SFRP1/CXCL10/GATA3/HOXD13/TACSTD2/FGF10/LHX1/BMP7 | 12 |
| GO:0015698 | BP | GO:0015698 | inorganic anion transport | 20/695 | 169/18870 | 4.31E-06 | 0.00070317 | 0.000599108 | GABRD/SLC12A1/SLC4A11/WNK4/CLIC5/CASR/ANO4/SLC22A8/SLC26A4/SLC17A4/CLCNKA/SLC34A3/SLC34A1/SLC12A3/NMUR2/GABRA2/SLC4A9/CLCNKB/SLC4A1/BSND | 20 |
| GO:0031093 | CC | GO:0031093 | platelet alpha granule lumen | 12/743 | 67/19886 | 6.15E-06 | 0.000339665 | 0.000297158 | VEGFA/EGF/VWF/HRG/KNG1/ALB/FGG/ORM2/FGA/ORM1/PLG/FGB | 12 |
| GO:0031091 | CC | GO:0031091 | platelet alpha granule | 14/743 | 91/19886 | 6.79E-06 | 0.000339665 | 0.000297158 | VEGFA/EGF/VWF/HRG/KNG1/TREML1/ALB/SERPINA5/FGG/ORM2/FGA/ORM1/PLG/FGB | 14 |
| GO:0072044 | BP | GO:0072044 | collecting duct development | 6/695 | 15/18870 | 9.20E-06 | 0.001449256 | 0.00123478 | CALB1/WNT9B/AQP2/UMOD/TFAP2B/WNT7B | 6 |
| GO:0045178 | CC | GO:0045178 | basal part of cell | 28/743 | 301/19886 | 9.43E-06 | 0.000424294 | 0.000371195 | CA9/SLC16A3/KCNJ10/SLC4A11/ATP12A/VSIG1/CLDN19/SLC7A8/CASR/AQP2/PTH1R/UMOD/SLC22A8/ATP2B2/SLC13A3/SLC9A4/TACSTD2/ERBB4/AQP9/TF/PROM2/SLC22A7/ADCY8/S100G/SLC4A9/SLC4A1/CLDN8/BSND | 28 |
| GO:0003208 | BP | GO:0003208 | cardiac ventricle morphogenesis | 12/695 | 71/18870 | 9.89E-06 | 0.001506353 | 0.001283427 | PROX1/TNNT2/TNNI1/GATA3/TNNC1/TBX5/MYH7/GRHL2/NPY2R/POU4F1/NKX2-5/GATA4 | 12 |
| GO:0098739 | BP | GO:0098739 | import across plasma membrane | 22/695 | 212/18870 | 1.25E-05 | 0.001837875 | 0.001565888 | KCNJ10/SLC12A1/TRPV6/ATP12A/KCNJ1/KCNK9/SLC7A8/SLC15A2/SLC5A2/TRPV5/IFNG/SLC9A3/SLC9A4/SCN7A/SLC34A1/GRM1/SCNN1B/SLC12A3/SCN2A/SCNN1G/SCN10A/SLC9A2 | 22 |
| GO:0048880 | BP | GO:0048880 | sensory system development | 33/695 | 401/18870 | 1.52E-05 | 0.002169811 | 0.0018487 | HSF4/VEGFA/CALB1/SOX11/SLC6A3/BIRC7/C3/WNT9B/PROX1/FASLG/DLX1/ATP2B2/TFAP2B/STRA6/OPN4/HES5/GATA3/DLX2/OLFM3/NR2E1/VSX1/WNT7B/GJA8/LHX2/GRHL2/FGF10/LIM2/POU4F1/CRYAA/LHX1/BMP7/SOX1/KERA | 33 |
| GO:0050819 | BP | GO:0050819 | negative regulation of coagulation | 10/695 | 52/18870 | 1.68E-05 | 0.002324659 | 0.001980632 | HRG/KNG1/F2/F11/FGG/TSPAN8/HS3ST5/FGA/PLG/FGB | 10 |
| GO:0003206 | BP | GO:0003206 | cardiac chamber morphogenesis | 16/695 | 127/18870 | 1.76E-05 | 0.002361933 | 0.002012389 | SOX11/DNAH11/PROX1/TNNT2/TNNI1/GATA3/TNNC1/TBX5/MYH7/GRHL2/OVOL2/NPY2R/POU4F1/NKX2-5/BMP7/GATA4 | 16 |
| GO:0030856 | BP | GO:0030856 | regulation of epithelial cell differentiation | 19/695 | 171/18870 | 1.85E-05 | 0.002413278 | 0.002056136 | VEGFA/WNT9B/ALOX15B/CYP27B1/IFNG/TP73/MMP9/HES5/GATA3/KRT36/SULT2B1/FOXJ1/GRHL2/FGF10/OVOL2/LHX1/ESRP1/BMP7/REG3A | 19 |
| GO:0034706 | CC | GO:0034706 | sodium channel complex | 7/743 | 24/19886 | 1.96E-05 | 0.000801898 | 0.000701543 | GRIK3/SCN7A/SCNN1B/SCN2A/SCNN1G/GRIK5/SCN10A | 7 |
| GO:0072078 | BP | GO:0072078 | nephron tubule morphogenesis | 12/695 | 77/18870 | 2.32E-05 | 0.002945285 | 0.002509411 | VEGFA/IRX2/FGF1/WNK4/TCF21/WNT9B/IRX1/GPC3/HES5/GATA3/TACSTD2/LHX1 | 12 |
| GO:0010721 | BP | GO:0010721 | negative regulation of cell development | 26/695 | 287/18870 | 2.39E-05 | 0.002953511 | 0.00251642 | TREM2/VEGFA/SEMA5B/TNFAIP6/SOX11/PTHLH/LILRB4/SFRP1/PROX1/TMEM178A/HLA-G/DLX1/LAG3/RAG2/HES5/F2/DLX2/PAEP/NKX6-2/NR2E1/TTPA/MT3/FOXJ1/LHX2/PGLYRP2/BMP7 | 26 |
| GO:0001823 | BP | GO:0001823 | mesonephros development | 14/695 | 103/18870 | 2.46E-05 | 0.002957222 | 0.002519582 | VEGFA/CALB1/FGF1/SFRP1/TCF21/WNT9B/GPC3/GATA3/TACSTD2/FOXJ1/FGF10/SIM1/LHX1/BMP7 | 14 |
| GO:0009954 | BP | GO:0009954 | proximal/distal pattern formation | 8/695 | 34/18870 | 2.52E-05 | 0.002957222 | 0.002519582 | IRX2/IRX1/DLX1/HES5/DLX2/HOXB9/EN1/FGF10 | 8 |
| GO:0098661 | BP | GO:0098661 | inorganic anion transmembrane transport | 16/695 | 131/18870 | 2.60E-05 | 0.002968923 | 0.002529551 | GABRD/SLC12A1/SLC4A11/CLIC5/CASR/ANO4/SLC26A4/CLCNKA/SLC34A1/SLC12A3/NMUR2/GABRA2/SLC4A9/CLCNKB/SLC4A1/BSND | 16 |
| GO:0150063 | BP | GO:0150063 | visual system development | 32/695 | 395/18870 | 2.78E-05 | 0.003098511 | 0.002639961 | HSF4/VEGFA/CALB1/SOX11/SLC6A3/BIRC7/C3/WNT9B/PROX1/FASLG/DLX1/ATP2B2/TFAP2B/STRA6/OPN4/HES5/GATA3/DLX2/OLFM3/NR2E1/VSX1/WNT7B/GJA8/LHX2/GRHL2/FGF10/LIM2/CRYAA/LHX1/BMP7/SOX1/KERA | 32 |
| GO:0005272 | MF | GO:0005272 | sodium channel activity | 9/709 | 43/18496 | 2.97E-05 | 0.001321211 | 0.001125844 | GRIK3/SLC4A11/PKD2L1/SCN7A/SCNN1B/SCN2A/SCNN1G/GRIK5/SCN10A | 9 |
| GO:0072088 | BP | GO:0072088 | nephron epithelium morphogenesis | 12/695 | 79/18870 | 3.02E-05 | 0.003232645 | 0.002754245 | VEGFA/IRX2/FGF1/WNK4/TCF21/WNT9B/IRX1/GPC3/HES5/GATA3/TACSTD2/LHX1 | 12 |
| GO:0021772 | BP | GO:0021772 | olfactory bulb development | 8/695 | 35/18870 | 3.17E-05 | 0.003232645 | 0.002754245 | UNCX/DLX5/EOMES/DLX2/ERBB4/NR2E1/LHX2/ARX | 8 |
| GO:0098719 | BP | GO:0098719 | sodium ion import across plasma membrane | 7/695 | 26/18870 | 3.18E-05 | 0.003232645 | 0.002754245 | SLC5A2/SLC9A3/SLC9A4/SLC34A1/SCNN1B/SCNN1G/SLC9A2 | 7 |
| GO:0043010 | BP | GO:0043010 | camera-type eye development | 29/695 | 344/18870 | 3.18E-05 | 0.003232645 | 0.002754245 | HSF4/VEGFA/CALB1/SOX11/SLC6A3/BIRC7/WNT9B/PROX1/DLX1/ATP2B2/TFAP2B/STRA6/OPN4/HES5/GATA3/DLX2/NR2E1/VSX1/WNT7B/GJA8/LHX2/GRHL2/FGF10/LIM2/CRYAA/LHX1/BMP7/SOX1/KERA | 29 |
| GO:0042475 | BP | GO:0042475 | odontogenesis of dentin-containing tooth | 13/695 | 93/18870 | 3.53E-05 | 0.0035096 | 0.002990213 | DLX1/DLX2/SLC34A1/LHX8/DMP1/FGF10/SCN10A/DMRT3/BMP7/AMELY/KLK5/KLK4/NKX2-3 | 13 |
| GO:0050878 | BP | GO:0050878 | regulation of body fluid levels | 30/695 | 365/18870 | 3.76E-05 | 0.003656937 | 0.003115746 | VEGFA/HK2/SLC6A3/WNK4/VWF/HRG/PTPRO/KNG1/TREML1/TRPV5/AQP2/AVPR2/UMOD/CEL/F2/PROZ/F11/FGG/CYP4F2/ERBB4/TSPAN8/SCNN1B/SCNN1G/SAA1/FGF10/FGA/SLC4A9/PLG/SLC4A1/FGB | 30 |
| GO:0061333 | BP | GO:0061333 | renal tubule morphogenesis | 12/695 | 81/18870 | 3.90E-05 | 0.003717068 | 0.003166978 | VEGFA/IRX2/FGF1/WNK4/TCF21/WNT9B/IRX1/GPC3/HES5/GATA3/TACSTD2/LHX1 | 12 |
| GO:0042730 | BP | GO:0042730 | fibrinolysis | 7/695 | 27/18870 | 4.16E-05 | 0.003877759 | 0.003303888 | HRG/F2/F11/FGG/FGA/PLG/FGB | 7 |
| GO:0072070 | BP | GO:0072070 | loop of Henle development | 5/695 | 12/18870 | 4.26E-05 | 0.003898067 | 0.003321191 | IRX2/IRX1/UMOD/HES5/WNT7B | 5 |
| GO:0005216 | MF | GO:0005216 | monoatomic ion channel activity | 35/709 | 443/18496 | 4.55E-05 | 0.001910516 | 0.001628008 | GABRD/GRIK3/KCNJ10/SLC4A11/TRPV6/CLIC5/KCNJ1/TRPA1/KCNK9/KCNN1/CHRNA1/TRPV5/KCNK10/ANO4/PKD2L1/CLCNKA/P2RX2/CHRNA6/SCN7A/FXYD4/SCNN1B/KCNK17/SCN2A/GRIA4/SCNN1G/GRIK5/CHRND/NMUR2/GABRA2/SCN10A/CHRNA4/CLCNKB/CACNG2/KCNU1/BSND | 35 |
| GO:0002920 | BP | GO:0002920 | regulation of humoral immune response | 9/695 | 47/18870 | 4.60E-05 | 0.004120008 | 0.003510287 | TREM2/C3/SUSD4/CXCL13/CD5L/KLK7/CR2/FOXJ1/KLK5 | 9 |
| GO:0015711 | BP | GO:0015711 | organic anion transport | 34/695 | 443/18870 | 4.73E-05 | 0.004159979 | 0.003544343 | FABP6/SLC16A3/KCNJ10/SLC4A11/PLA2R1/FABP7/RBP2/ABAT/SLC16A5/SLC7A8/CASR/AVPR1B/SLC17A9/UMOD/SLC22A8/AGXT/SLC26A4/SLC17A4/SLC13A3/UGT1A3/SLC17A2/CYP4F2/AQP9/FABP1/GRM1/SLC13A2/SLC36A2/PLA2G2D/NMUR2/FOLR3/SLC7A13/SLC22A7/SLC4A9/SLC4A1 | 34 |
| GO:0021988 | BP | GO:0021988 | olfactory lobe development | 8/695 | 37/18870 | 4.87E-05 | 0.004196811 | 0.003575724 | UNCX/DLX5/EOMES/DLX2/ERBB4/NR2E1/LHX2/ARX | 8 |
| GO:1902476 | BP | GO:1902476 | chloride transmembrane transport | 14/695 | 110/18870 | 5.19E-05 | 0.004393179 | 0.003743031 | GABRD/SLC12A1/CLIC5/CASR/ANO4/SLC26A4/CLCNKA/SLC12A3/NMUR2/GABRA2/SLC4A9/CLCNKB/SLC4A1/BSND | 14 |
| GO:0045686 | BP | GO:0045686 | negative regulation of glial cell differentiation | 7/695 | 28/18870 | 5.37E-05 | 0.00444978 | 0.003791256 | TREM2/DLX1/HES5/F2/DLX2/NKX6-2/NR2E1 | 7 |
| GO:0030195 | BP | GO:0030195 | negative regulation of blood coagulation | 9/695 | 48/18870 | 5.48E-05 | 0.00444978 | 0.003791256 | HRG/KNG1/F2/F11/FGG/TSPAN8/FGA/PLG/FGB | 9 |
| GO:0001654 | BP | GO:0001654 | eye development | 31/695 | 391/18870 | 5.55E-05 | 0.00444978 | 0.003791256 | HSF4/VEGFA/CALB1/SOX11/SLC6A3/BIRC7/WNT9B/PROX1/FASLG/DLX1/ATP2B2/TFAP2B/STRA6/OPN4/HES5/GATA3/DLX2/OLFM3/NR2E1/VSX1/WNT7B/GJA8/LHX2/GRHL2/FGF10/LIM2/CRYAA/LHX1/BMP7/SOX1/KERA | 31 |
| GO:0006821 | BP | GO:0006821 | chloride transport | 15/695 | 125/18870 | 5.67E-05 | 0.004468857 | 0.003807509 | GABRD/SLC12A1/WNK4/CLIC5/CASR/ANO4/SLC26A4/CLCNKA/SLC12A3/NMUR2/GABRA2/SLC4A9/CLCNKB/SLC4A1/BSND | 15 |
| GO:0022839 | MF | GO:0022839 | monoatomic ion gated channel activity | 28/709 | 325/18496 | 5.90E-05 | 0.002348631 | 0.00200134 | GABRD/GRIK3/KCNJ10/CLIC5/KCNJ1/TRPA1/KCNK9/KCNN1/CHRNA1/KCNK10/PKD2L1/CLCNKA/P2RX2/CHRNA6/SCN7A/SCNN1B/KCNK17/SCN2A/GRIA4/SCNN1G/GRIK5/CHRND/GABRA2/SCN10A/CHRNA4/CLCNKB/CACNG2/KCNU1 | 28 |
| GO:0001657 | BP | GO:0001657 | ureteric bud development | 13/695 | 98/18870 | 6.18E-05 | 0.0047401 | 0.004038611 | VEGFA/CALB1/FGF1/SFRP1/TCF21/WNT9B/GPC3/GATA3/TACSTD2/FOXJ1/SIM1/LHX1/BMP7 | 13 |
| GO:0003014 | BP | GO:0003014 | renal system process | 15/695 | 126/18870 | 6.22E-05 | 0.0047401 | 0.004038611 | WNK4/PTPRO/SLC15A2/SLC5A2/TRPV5/AQP2/AVPR2/UMOD/CLCNKA/NPHS2/CYP4F2/SCNN1B/SLC12A3/CLCNKB/SLC4A1 | 15 |
| GO:0022836 | MF | GO:0022836 | gated channel activity | 28/709 | 326/18496 | 6.23E-05 | 0.002354348 | 0.002006212 | GABRD/GRIK3/KCNJ10/CLIC5/KCNJ1/TRPA1/KCNK9/KCNN1/CHRNA1/KCNK10/PKD2L1/CLCNKA/P2RX2/CHRNA6/SCN7A/SCNN1B/KCNK17/SCN2A/GRIA4/SCNN1G/GRIK5/CHRND/GABRA2/SCN10A/CHRNA4/CLCNKB/CACNG2/KCNU1 | 28 |
| GO:0048546 | BP | GO:0048546 | digestive tract morphogenesis | 9/695 | 49/18870 | 6.49E-05 | 0.004784696 | 0.004076607 | SOX11/SFRP1/TCF21/STRA6/HOXD13/FGF10/OVOL2/GATA4/NKX2-3 | 9 |
| GO:1900047 | BP | GO:1900047 | negative regulation of hemostasis | 9/695 | 49/18870 | 6.49E-05 | 0.004784696 | 0.004076607 | HRG/KNG1/F2/F11/FGG/TSPAN8/FGA/PLG/FGB | 9 |
| GO:0006820 | BP | GO:0006820 | monoatomic anion transport | 18/695 | 172/18870 | 6.75E-05 | 0.004836151 | 0.004120448 | GABRD/SLC12A1/SLC4A11/WNK4/CLIC5/CASR/ANO4/SLC26A4/SLC17A4/CLCNKA/SLC17A2/SLC12A3/NMUR2/GABRA2/SLC4A9/CLCNKB/SLC4A1/BSND | 18 |
| GO:0072163 | BP | GO:0072163 | mesonephric epithelium development | 13/695 | 99/18870 | 6.88E-05 | 0.004836151 | 0.004120448 | VEGFA/CALB1/FGF1/SFRP1/TCF21/WNT9B/GPC3/GATA3/TACSTD2/FOXJ1/SIM1/LHX1/BMP7 | 13 |
| GO:0072164 | BP | GO:0072164 | mesonephric tubule development | 13/695 | 99/18870 | 6.88E-05 | 0.004836151 | 0.004120448 | VEGFA/CALB1/FGF1/SFRP1/TCF21/WNT9B/GPC3/GATA3/TACSTD2/FOXJ1/SIM1/LHX1/BMP7 | 13 |
| GO:1990266 | BP | GO:1990266 | neutrophil migration | 15/695 | 129/18870 | 8.17E-05 | 0.005657341 | 0.004820109 | TNFAIP6/CCL18/CCL5/GP2/CXCL9/CXCL10/CXCL11/UMOD/DPEP1/CXCL5/CXCL13/CCL20/SAA1/CCL25/LBP | 15 |
| GO:0007389 | BP | GO:0007389 | pattern specification process | 35/695 | 475/18870 | 8.36E-05 | 0.005704259 | 0.004860084 | SIM2/IRX2/FGF1/WNT8B/UNCX/DNAH11/SFRP1/C3/RFX8/EOMES/IRX1/DLX1/GPC3/HELT/HES5/DLX2/HOXB9/HOXD13/EN1/TBX5/ERBB4/WNT7B/GBX2/LMX1B/FOXJ1/LHX2/FGF10/OVOL2/BARX1/LHX1/NKX2-5/DMRT3/BMP7/SOX1/GATA4 | 35 |
| GO:0008009 | MF | GO:0008009 | chemokine activity | 9/709 | 49/18496 | 8.81E-05 | 0.00317313 | 0.002703921 | CCL18/CCL5/CXCL9/CXCL10/CXCL11/CXCL5/CXCL13/CCL20/CCL25 | 9 |
| GO:0097529 | BP | GO:0097529 | myeloid leukocyte migration | 22/695 | 242/18870 | 9.42E-05 | 0.006333967 | 0.005396601 | TREM2/VEGFA/TNFAIP6/PGF/PLA2G7/PTPRO/CCL18/CCL5/GP2/CXCL9/CXCL10/CXCL11/UMOD/SCG2/DPEP1/CXCL5/CXCL13/CCL20/SAA1/CCL25/LBP/CALCA | 22 |
| GO:1902074 | BP | GO:1902074 | response to salt | 29/695 | 366/18870 | 9.66E-05 | 0.006400295 | 0.005453113 | KCNJ10/SLC6A3/INHBB/TRPV6/CPNE6/ABAT/CRHBP/CHP2/CASR/AQP2/TNNT2/UMOD/DPEP1/ITPKA/PKD2L1/FGG/LY6H/P2RX2/EN1/SYT7/SLC34A1/SLC13A2/PPP1R1B/CHRND/TACR3/ADCY8/FGA/FGB/CACNG2 | 29 |
| GO:0005179 | MF | GO:0005179 | hormone activity | 15/709 | 126/18496 | 9.71E-05 | 0.003336555 | 0.00284318 | STC2/INHBB/PTHLH/KNG1/HAMP/PMCH/EPO/INHBE/CHGB/SST/CCL25/APELA/FSHB/REG3A/CALCA | 15 |
| GO:0055074 | BP | GO:0055074 | calcium ion homeostasis | 27/695 | 331/18870 | 0.000103015 | 0.00672544 | 0.00573014 | NOL3/STC2/CALB1/TRPV6/FATE1/WNK4/NOS1/TRPA1/FASLG/CCL5/HCRTR2/TMEM178A/CASR/CXCL9/CXCL10/CYP27B1/TRPV5/SCGN/CXCL11/PTH1R/UMOD/ATP2B2/F2/GRM1/GPR12/ADCY8/CALCA | 27 |
| GO:0072210 | BP | GO:0072210 | metanephric nephron development | 8/695 | 41/18870 | 0.000105773 | 0.006779442 | 0.00577615 | IRX2/TCF21/WNT9B/IRX1/TFAP2B/HES5/NPHS2/LHX1 | 8 |
| GO:0048247 | BP | GO:0048247 | lymphocyte chemotaxis | 10/695 | 64/18870 | 0.000107995 | 0.006779442 | 0.00577615 | CCL18/CCL5/TNFSF14/CXCL10/CXCL11/KLRK1/CXCL13/CCL20/SAA1/CCL25 | 10 |
| GO:0003338 | BP | GO:0003338 | metanephros morphogenesis | 7/695 | 31/18870 | 0.000108293 | 0.006779442 | 0.00577615 | CALB1/WNT9B/HES5/WNT7B/FOXJ1/FGF10/LHX1 | 7 |
| GO:0015079 | MF | GO:0015079 | potassium ion transmembrane transporter activity | 17/709 | 157/18496 | 0.000112951 | 0.003712642 | 0.003163655 | GRIK3/KCNJ10/SLC12A1/ATP12A/KCNJ1/KCNK9/KCNN1/KCNK10/PKD2L1/SLC9A3/SLC9A4/FXYD4/KCNK17/SLC12A3/GRIK5/SLC9A2/KCNU1 | 17 |
| GO:0046942 | BP | GO:0046942 | carboxylic acid transport | 28/695 | 351/18870 | 0.000113069 | 0.00698279 | 0.005949405 | FABP6/SLC16A3/KCNJ10/PLA2R1/FABP7/RBP2/ABAT/SLC16A5/SLC7A8/CASR/AVPR1B/UMOD/SLC22A8/AGXT/SLC26A4/SLC13A3/UGT1A3/CYP4F2/AQP9/FABP1/GRM1/SLC13A2/SLC36A2/PLA2G2D/NMUR2/FOLR3/SLC7A13/SLC22A7 | 28 |
| GO:0001666 | BP | GO:0001666 | response to hypoxia | 25/695 | 298/18870 | 0.000118494 | 0.007103161 | 0.006051962 | EGLN3/HILPDA/NOL3/CA9/STC2/ANGPTL4/TREM2/VEGFA/HK2/PGF/ANGPT2/SFRP1/NOS1/ABAT/CASR/TERT/P2RX2/EPO/FMN2/FABP1/SCN2A/MGARP/MT3/BMP7/CHRNA4 | 25 |
| GO:0015849 | BP | GO:0015849 | organic acid transport | 28/695 | 352/18870 | 0.000118663 | 0.007103161 | 0.006051962 | FABP6/SLC16A3/KCNJ10/PLA2R1/FABP7/RBP2/ABAT/SLC16A5/SLC7A8/CASR/AVPR1B/UMOD/SLC22A8/AGXT/SLC26A4/SLC13A3/UGT1A3/CYP4F2/AQP9/FABP1/GRM1/SLC13A2/SLC36A2/PLA2G2D/NMUR2/FOLR3/SLC7A13/SLC22A7 | 28 |
| GO:0060562 | BP | GO:0060562 | epithelial tube morphogenesis | 27/695 | 334/18870 | 0.000119681 | 0.007103161 | 0.006051962 | VEGFA/IRX2/FGF1/ITGAX/WNK4/EGF/SFRP1/TCF21/WNT9B/PROX1/CASR/IRX1/CXCL10/GPC3/HES5/GATA3/CTHRC1/TACSTD2/GBX2/LHX2/GRHL2/FGF10/OVOL2/LHX1/NKX2-5/BMP7/GATA4 | 27 |
| GO:0007586 | BP | GO:0007586 | digestion | 15/695 | 134/18870 | 0.000126074 | 0.007386629 | 0.006293479 | SCARB1/WNK4/VSIG1/HRH2/CHIT1/HAMP/CEL/SLC9A4/FABP1/MOGAT2/FGF10/SST/ARX/SLC4A9/APOA4 | 15 |
| GO:0042886 | BP | GO:0042886 | amide transport | 28/695 | 354/18870 | 0.0001306 | 0.007554951 | 0.006436892 | KCNJ10/INHBB/SFRP1/SLC14A2/PTPRN/ABAT/CCL5/CRHBP/SLC15A2/CASR/UMOD/IFNG/TFR2/TFAP2B/FAM3B/F2/FGG/AQP9/SYT7/GRM1/ABCA13/SSTR5/FOLR3/SLC7A13/SLC30A8/ADCY8/FGA/FGB | 28 |
| GO:0045907 | BP | GO:0045907 | positive regulation of vasoconstriction | 7/695 | 32/18870 | 0.000134246 | 0.007668792 | 0.006533885 | CASR/AVPR1B/HRH2/AVPR2/FGG/FGA/FGB | 7 |
| GO:0009914 | BP | GO:0009914 | hormone transport | 26/695 | 319/18870 | 0.000139364 | 0.00786291 | 0.006699275 | SOX11/INHBB/TRPV6/WNK4/SFRP1/PTPRN/ABAT/CCL5/CRHBP/SLC7A8/CASR/IFNG/TFR2/TFAP2B/GATA3/FAM3B/F2/SLC17A4/FGG/SYT7/SSTR5/IL11/SLC30A8/ADCY8/FGA/FGB | 26 |
| GO:0042330 | BP | GO:0042330 | taxis | 34/695 | 470/18870 | 0.000149557 | 0.008335049 | 0.007101542 | TREM2/VEGFA/SEMA5B/TNFAIP6/FGF1/PGF/ANGPT2/PLA2G7/HRG/LOX/PTPRO/CCL18/CCL5/CASR/TNFSF14/CXCL9/CXCL10/CXCL11/CXCR3/SCG2/KLRK1/DPEP1/OPN4/CXCL5/CXCL13/CCL20/HOXB9/PIK3C2G/SAA1/FGF10/L1CAM/CCL25/LBP/CALCA | 34 |
| GO:0030593 | BP | GO:0030593 | neutrophil chemotaxis | 13/695 | 107/18870 | 0.000153896 | 0.008473525 | 0.007219525 | TNFAIP6/CCL18/CCL5/CXCL9/CXCL10/CXCL11/DPEP1/CXCL5/CXCL13/CCL20/SAA1/CCL25/LBP | 13 |
| GO:0015276 | MF | GO:0015276 | ligand-gated monoatomic ion channel activity | 16/709 | 146/18496 | 0.000154651 | 0.004871507 | 0.004151159 | GABRD/GRIK3/KCNJ10/KCNJ1/TRPA1/KCNN1/CHRNA1/P2RX2/CHRNA6/SCNN1B/GRIA4/SCNN1G/GRIK5/CHRND/GABRA2/CHRNA4 | 16 |
| GO:0003205 | BP | GO:0003205 | cardiac chamber development | 17/695 | 168/18870 | 0.000160945 | 0.008756176 | 0.007460347 | SOX11/DNAH11/PROX1/TNNT2/TNNI1/STRA6/GATA3/TNNC1/TBX5/MYH7/GRHL2/OVOL2/NPY2R/POU4F1/NKX2-5/BMP7/GATA4 | 17 |
| GO:0006869 | BP | GO:0006869 | lipid transport | 33/695 | 453/18870 | 0.000163746 | 0.008803759 | 0.007500888 | SCARB1/FABP6/TREM2/APOC1/PLA2R1/WNK4/FABP7/EGF/RBP2/ATP8B3/ABCA4/FASLG/AVPR1B/ANO4/SERPINA5/SLC22A8/CEL/STRA6/APOL5/SLC13A3/APOB/CYP4F2/AQP9/SYT7/FABP1/ABCA13/PLA2G2D/NMUR2/TTPA/SLC22A7/LBP/SLC4A1/APOA4 | 33 |
| GO:0034702 | CC | GO:0034702 | monoatomic ion channel complex | 25/743 | 300/19886 | 0.000165447 | 0.006204275 | 0.005427834 | GABRD/GRIK3/HSPA2/TRPV6/CLIC5/KCNN1/CHRNA1/PKD2L1/CLCNKA/OLFM3/CHRNA6/SCN7A/SCNN1B/SCN2A/GRIA4/SCNN1G/GRIK5/CHRND/DPP6/GABRA2/SCN10A/CHRNA4/CATSPERD/CLCNKB/CACNG2 | 25 |
| GO:0022834 | MF | GO:0022834 | ligand-gated channel activity | 16/709 | 147/18496 | 0.000167507 | 0.005065408 | 0.004316388 | GABRD/GRIK3/KCNJ10/KCNJ1/TRPA1/KCNN1/CHRNA1/P2RX2/CHRNA6/SCNN1B/GRIA4/SCNN1G/GRIK5/CHRND/GABRA2/CHRNA4 | 16 |
| GO:0034483 | MF | GO:0034483 | heparan sulfate sulfotransferase activity | 5/709 | 15/18496 | 0.000177849 | 0.005171311 | 0.004406631 | HS6ST2/HS3ST2/NDST3/HS3ST6/HS3ST5 | 5 |
| GO:0060326 | BP | GO:0060326 | cell chemotaxis | 26/695 | 325/18870 | 0.000187594 | 0.009968674 | 0.008493406 | VEGFA/TNFAIP6/FGF1/PGF/PLA2G7/HRG/LOX/PTPRO/CCL18/CCL5/TNFSF14/CXCL9/CXCL10/CXCL11/CXCR3/SCG2/KLRK1/DPEP1/CXCL5/CXCL13/CCL20/HOXB9/SAA1/CCL25/LBP/CALCA | 26 |
| GO:0050997 | MF | GO:0050997 | quaternary ammonium group binding | 9/709 | 54/18496 | 0.00019234 | 0.005385534 | 0.004589177 | APOC1/SLC5A7/CHRNA1/SERPINA5/CRP/CHRND/GPR12/CHRNA4/APOA4 | 9 |
| GO:0050863 | BP | GO:0050863 | regulation of T cell activation | 29/695 | 381/18870 | 0.000192966 | 0.01013626 | 0.008636192 | CD70/IDO1/IL20RB/PIK3R6/LILRB4/TNFSF9/CCL5/TNFRSF9/HLA-G/CD2/ZNF683/TNFSF14/BATF/CRTAM/ICOS/LAT/SIRPG/IFNG/LAG3/CD27/KLRK1/RAG2/GATA3/HHLA2/EPO/PLA2G2D/FOXJ1/VTCN1/ACTL6B | 29 |
| GO:0043025 | CC | GO:0043025 | neuronal cell body | 35/743 | 489/19886 | 0.000196182 | 0.006790906 | 0.00594105 | GABRD/GRIK3/CALB1/DDN/ENO2/SLC6A3/KLHL14/CPNE6/PTPRN/CRHBP/KCNN1/CASR/SLC5A7/FLRT1/ATP2B2/OPN4/SPTBN2/UNC5A/APOB/P2RX2/PPP1R1B/GRIA4/GABRA2/TACR3/L1CAM/STMN2/SST/ADCY8/BRINP3/SNCB/CPLX2/CHRNA4/FRMD7/KLHL1/CALCA | 35 |
| GO:0070293 | BP | GO:0070293 | renal absorption | 7/695 | 34/18870 | 0.000201261 | 0.010451874 | 0.008905097 | WNK4/SLC15A2/SLC5A2/UMOD/CLCNKA/SLC12A3/CLCNKB | 7 |
| GO:0015108 | MF | GO:0015108 | chloride transmembrane transporter activity | 14/709 | 120/18496 | 0.000203792 | 0.005502375 | 0.004688741 | GABRD/SLC6A3/SLC12A1/CLIC5/ANO4/SLC26A4/CLCNKA/SLC12A3/NMUR2/GABRA2/SLC4A9/CLCNKB/SLC4A1/BSND | 14 |
| GO:0014014 | BP | GO:0014014 | negative regulation of gliogenesis | 8/695 | 45/18870 | 0.000209584 | 0.010761806 | 0.009169162 | TREM2/SOX11/DLX1/HES5/F2/DLX2/NKX6-2/NR2E1 | 8 |
| GO:0015297 | MF | GO:0015297 | antiporter activity | 15/709 | 135/18496 | 0.000211261 | 0.005507358 | 0.004692987 | SLC4A11/SLC7A8/SLC47A2/SLC22A8/SLC26A4/SLC9A3/SLC9A4/SLC18A3/SLC30A2/SLC7A13/SLC30A8/SLC9A2/SLC4A9/SLC4A1/SLC30A10 | 15 |
| GO:0097530 | BP | GO:0097530 | granulocyte migration | 16/695 | 156/18870 | 0.000212804 | 0.010805704 | 0.009206565 | TNFAIP6/CCL18/CCL5/GP2/CXCL9/CXCL10/CXCL11/UMOD/SCG2/DPEP1/CXCL5/CXCL13/CCL20/SAA1/CCL25/LBP | 16 |
| GO:0061005 | BP | GO:0061005 | cell differentiation involved in kidney development | 9/695 | 57/18870 | 0.000218669 | 0.010981491 | 0.009356336 | TCF21/WNT9B/PTPRO/NPHS1/MMP9/GATA3/NPHS2/FOXJ1/LHX1 | 9 |
| GO:1904315 | MF | GO:1904315 | transmitter-gated monoatomic ion channel activity involved in regulation of postsynaptic membrane potential | 9/709 | 55/18496 | 0.00022226 | 0.005600959 | 0.004772747 | GABRD/GRIK3/CHRNA1/CHRNA6/GRIA4/GRIK5/CHRND/GABRA2/CHRNA4 | 9 |
| GO:0030595 | BP | GO:0030595 | leukocyte chemotaxis | 21/695 | 240/18870 | 0.0002292 | 0.011370413 | 0.009687702 | VEGFA/TNFAIP6/PGF/PLA2G7/PTPRO/CCL18/CCL5/TNFSF14/CXCL9/CXCL10/CXCL11/SCG2/KLRK1/DPEP1/CXCL5/CXCL13/CCL20/SAA1/CCL25/LBP/CALCA | 21 |
| GO:0050953 | BP | GO:0050953 | sensory perception of light stimulus | 20/695 | 223/18870 | 0.000231389 | 0.011370413 | 0.009687702 | SEMA5B/KCNJ10/MYO3A/CLIC5/CLDN19/ABCA4/EYA4/TGFBI/OPN4/MYO3B/TACSTD2/GJA3/NR2E1/VSX1/PCDH15/CRYAA/RDH8/ZIC2/GUCA1C/KERA | 20 |
| GO:0043501 | BP | GO:0043501 | skeletal muscle adaptation | 6/695 | 25/18870 | 0.000237435 | 0.01154339 | 0.00983508 | MYOZ2/TNNI1/TNNC1/TNNT1/MYH7/MYOD1 | 6 |
| GO:2000241 | BP | GO:2000241 | regulation of reproductive process | 19/695 | 207/18870 | 0.000244788 | 0.01161448 | 0.009895649 | VEGFA/INHBB/SFRP1/TPPP2/STRA8/TEX11/SPINK13/SEMG2/RBM46/ZP1/PAEP/HOXD13/SEMG1/HORMAD1/TACR3/BMP7/APELA/DMRT1/NANOS2 | 19 |
| GO:0055078 | BP | GO:0055078 | sodium ion homeostasis | 8/695 | 46/18870 | 0.00024562 | 0.01161448 | 0.009895649 | SLC12A1/ATP12A/UMOD/SCN7A/CYP4F2/SCNN1B/SLC12A3/SCNN1G | 8 |
| GO:0003231 | BP | GO:0003231 | cardiac ventricle development | 14/695 | 127/18870 | 0.000246522 | 0.01161448 | 0.009895649 | SOX11/PROX1/TNNT2/TNNI1/STRA6/GATA3/TNNC1/TBX5/MYH7/GRHL2/NPY2R/POU4F1/NKX2-5/GATA4 | 14 |
| GO:0048562 | BP | GO:0048562 | embryonic organ morphogenesis | 24/695 | 295/18870 | 0.000255194 | 0.011900354 | 0.010139217 | SOX11/DLX5/MYO3A/TCF21/WNT9B/PROX1/STRA6/TBX15/GATA3/CTHRC1/DLX2/MYO3B/HOXB9/GBX2/ALX1/GRHL2/FGF10/OVOL2/LHFPL5/LHX1/HMX2/NKX2-5/BMP7/GATA4 | 24 |
| GO:0015103 | MF | GO:0015103 | inorganic anion transmembrane transporter activity | 16/709 | 153/18496 | 0.000265962 | 0.006486036 | 0.005526948 | GABRD/SLC6A3/SLC12A1/SLC4A11/CLIC5/ANO4/SLC22A8/SLC26A4/CLCNKA/SLC12A3/NMUR2/GABRA2/SLC4A9/CLCNKB/SLC4A1/BSND | 16 |
| GO:1990868 | BP | GO:1990868 | response to chemokine | 12/695 | 99/18870 | 0.000279336 | 0.01256735 | 0.010707504 | TREM2/LOX/CCL18/CCL5/CXCL9/CXCL10/CXCL11/CXCR3/CXCL5/CXCL13/CCL20/CCL25 | 12 |
| GO:1990869 | BP | GO:1990869 | cellular response to chemokine | 12/695 | 99/18870 | 0.000279336 | 0.01256735 | 0.010707504 | TREM2/LOX/CCL18/CCL5/CXCL9/CXCL10/CXCL11/CXCR3/CXCL5/CXCL13/CCL20/CCL25 | 12 |
| GO:0036293 | BP | GO:0036293 | response to decreased oxygen levels | 25/695 | 315/18870 | 0.000279494 | 0.01256735 | 0.010707504 | EGLN3/HILPDA/NOL3/CA9/STC2/ANGPTL4/TREM2/VEGFA/HK2/PGF/ANGPT2/SFRP1/NOS1/ABAT/CASR/TERT/P2RX2/EPO/FMN2/FABP1/SCN2A/MGARP/MT3/BMP7/CHRNA4 | 25 |
| GO:0051592 | BP | GO:0051592 | response to calcium ion | 15/695 | 144/18870 | 0.000280497 | 0.01256735 | 0.010707504 | INHBB/TRPV6/CPNE6/CRHBP/CHP2/CASR/TNNT2/DPEP1/ITPKA/FGG/SYT7/ADCY8/FGA/FGB/CACNG2 | 15 |
| GO:0051480 | BP | GO:0051480 | regulation of cytosolic calcium ion concentration | 9/695 | 59/18870 | 0.000286073 | 0.012592633 | 0.010729045 | CALB1/HCRTR2/TMEM178A/SCGN/ATP2B2/F2/GRM1/ADCY8/CALCA | 9 |
| GO:0035850 | BP | GO:0035850 | epithelial cell differentiation involved in kidney development | 8/695 | 47/18870 | 0.000286572 | 0.012592633 | 0.010729045 | WNT9B/PTPRO/NPHS1/MMP9/GATA3/NPHS2/FOXJ1/LHX1 | 8 |
| GO:2000352 | BP | GO:2000352 | negative regulation of endothelial cell apoptotic process | 7/695 | 36/18870 | 0.000292962 | 0.012750828 | 0.010863829 | ANGPTL4/SCG2/GATA3/FGG/TERT/FGA/FGB | 7 |
| GO:0099529 | MF | GO:0099529 | neurotransmitter receptor activity involved in regulation of postsynaptic membrane potential | 9/709 | 57/18496 | 0.000293749 | 0.006860922 | 0.0058464 | GABRD/GRIK3/CHRNA1/CHRNA6/GRIA4/GRIK5/CHRND/GABRA2/CHRNA4 | 9 |
| GO:0006935 | BP | GO:0006935 | chemotaxis | 33/695 | 468/18870 | 0.000296572 | 0.012775697 | 0.010885018 | TREM2/VEGFA/SEMA5B/TNFAIP6/FGF1/PGF/ANGPT2/PLA2G7/HRG/LOX/PTPRO/CCL18/CCL5/CASR/TNFSF14/CXCL9/CXCL10/CXCL11/CXCR3/SCG2/KLRK1/DPEP1/CXCL5/CXCL13/CCL20/HOXB9/PIK3C2G/SAA1/FGF10/L1CAM/CCL25/LBP/CALCA | 33 |
| GO:0050926 | BP | GO:0050926 | regulation of positive chemotaxis | 6/695 | 26/18870 | 0.000299125 | 0.012775697 | 0.010885018 | VEGFA/PGF/ANGPT2/CASR/SCG2/FGF10 | 6 |
| GO:0099094 | MF | GO:0099094 | ligand-gated monoatomic cation channel activity | 13/709 | 110/18496 | 0.000299485 | 0.006860922 | 0.0058464 | GRIK3/KCNJ10/KCNJ1/TRPA1/KCNN1/CHRNA1/P2RX2/CHRNA6/SCNN1B/SCNN1G/GRIK5/CHRND/CHRNA4 | 13 |
| GO:0071621 | BP | GO:0071621 | granulocyte chemotaxis | 14/695 | 130/18870 | 0.000314513 | 0.013308558 | 0.011339021 | TNFAIP6/CCL18/CCL5/CXCL9/CXCL10/CXCL11/SCG2/DPEP1/CXCL5/CXCL13/CCL20/SAA1/CCL25/LBP | 14 |
| GO:0034358 | CC | GO:0034358 | plasma lipoprotein particle | 7/743 | 36/19886 | 0.000319952 | 0.009094078 | 0.007955988 | APOC1/PLA2G7/APOB/SAA1/SAA2/SAA4/APOA4 | 7 |
| GO:1990777 | CC | GO:1990777 | lipoprotein particle | 7/743 | 36/19886 | 0.000319952 | 0.009094078 | 0.007955988 | APOC1/PLA2G7/APOB/SAA1/SAA2/SAA4/APOA4 | 7 |
| GO:0034364 | CC | GO:0034364 | high-density lipoprotein particle | 6/743 | 26/19886 | 0.000323345 | 0.009094078 | 0.007955988 | APOC1/PLA2G7/SAA1/SAA2/SAA4/APOA4 | 6 |
| GO:0045604 | BP | GO:0045604 | regulation of epidermal cell differentiation | 9/695 | 60/18870 | 0.000325729 | 0.01345177 | 0.011461038 | ALOX15B/CYP27B1/HES5/KRT36/SULT2B1/GRHL2/OVOL2/ESRP1/REG3A | 9 |
| GO:0045109 | BP | GO:0045109 | intermediate filament organization | 10/695 | 73/18870 | 0.0003291 | 0.01345177 | 0.011461038 | KRT81/KRT36/KRT32/KRT72/KRT40/KRT25/KRT6A/KRT78/KRT20/KRT6B | 10 |
| GO:0050818 | BP | GO:0050818 | regulation of coagulation | 10/695 | 73/18870 | 0.0003291 | 0.01345177 | 0.011461038 | HRG/KNG1/F2/F11/FGG/TSPAN8/HS3ST5/FGA/PLG/FGB | 10 |
| GO:0048469 | BP | GO:0048469 | cell maturation | 18/695 | 195/18870 | 0.000329671 | 0.01345177 | 0.011461038 | VEGFA/C3/LGI4/PTH1R/SEMG2/HES5/GATA3/FGG/PAEP/C1QL1/EPO/SEMG1/VSX1/FOXJ1/TFCP2L1/HOXB13/CATSPERD/ACTL6B | 18 |
| GO:0071456 | BP | GO:0071456 | cellular response to hypoxia | 15/695 | 147/18870 | 0.000350862 | 0.01418973 | 0.012089787 | EGLN3/HILPDA/NOL3/STC2/TREM2/VEGFA/SFRP1/CASR/TERT/FMN2/FABP1/SCN2A/MGARP/MT3/BMP7 | 15 |
| GO:0005126 | MF | GO:0005126 | cytokine receptor binding | 23/709 | 273/18496 | 0.000361936 | 0.008047749 | 0.006857731 | NOL3/VEGFA/CD70/CD300LF/PGF/TNFSF9/CCL18/FASLG/CCL5/TNFSF14/CXCL9/CXCL10/RASL11B/CXCL11/IFNG/GATA3/CXCL5/CXCL13/CCL20/EPO/IL11/CCL25/IFNA14 | 23 |
| GO:0050900 | BP | GO:0050900 | leukocyte migration | 29/695 | 396/18870 | 0.000367212 | 0.014696159 | 0.012521269 | TREM2/VEGFA/TNFAIP6/PGF/PLA2G7/PTPRO/CCL18/CCL5/GP2/TNFSF14/CXCL9/CXCL10/CRTAM/CXCL11/CXCR3/UMOD/SCG2/KLRK1/DPEP1/GATA3/CXCL5/CXCL13/CCL20/SAA1/FOXJ1/CCL25/LBP/NKX2-3/CALCA | 29 |
| GO:2000351 | BP | GO:2000351 | regulation of endothelial cell apoptotic process | 9/695 | 61/18870 | 0.000369816 | 0.014696159 | 0.012521269 | ANGPTL4/FASLG/HLA-G/SCG2/GATA3/FGG/TERT/FGA/FGB | 9 |
| GO:0008188 | MF | GO:0008188 | neuropeptide receptor activity | 8/709 | 47/18496 | 0.000374609 | 0.008091552 | 0.006895057 | KISS1R/MCHR1/HCRTR2/SSTR5/NMUR2/TACR3/SORCS3/NPY2R | 8 |
| GO:0030594 | MF | GO:0030594 | neurotransmitter receptor activity | 12/709 | 99/18496 | 0.0004004 | 0.008223341 | 0.007007358 | GABRD/GRIK3/HTR6/CHRNA1/HRH2/CHRNA6/GRM1/GRIA4/GRIK5/CHRND/GABRA2/CHRNA4 | 12 |
| GO:0098960 | MF | GO:0098960 | postsynaptic neurotransmitter receptor activity | 10/709 | 72/18496 | 0.000402465 | 0.008223341 | 0.007007358 | GABRD/GRIK3/CHRNA1/CHRNA6/GRM1/GRIA4/GRIK5/CHRND/GABRA2/CHRNA4 | 10 |
| GO:0022600 | BP | GO:0022600 | digestive system process | 12/695 | 103/18870 | 0.000404337 | 0.015929496 | 0.013572085 | SCARB1/WNK4/VSIG1/HRH2/HAMP/CEL/SLC9A4/FABP1/MOGAT2/FGF10/SLC4A9/APOA4 | 12 |
| GO:0061045 | BP | GO:0061045 | negative regulation of wound healing | 10/695 | 75/18870 | 0.000411058 | 0.016055851 | 0.01367974 | CLDN19/HRG/KNG1/F2/F11/FGG/TSPAN8/FGA/PLG/FGB | 10 |
| GO:0048608 | BP | GO:0048608 | reproductive structure development | 24/695 | 305/18870 | 0.000415843 | 0.016105102 | 0.013721703 | VEGFA/TNFAIP6/INHBB/SFRP1/TCF21/PTPRN/ADAM18/C3/WNT9B/ALOX15B/TEX11/STRA6/GATA3/HSD17B3/MSH4/HOXD13/LHX8/FGF10/HOXB13/LHX1/BMP7/GATA4/FSHB/DMRT1 | 24 |
| GO:0070482 | BP | GO:0070482 | response to oxygen levels | 26/695 | 343/18870 | 0.000432751 | 0.016619076 | 0.014159613 | EGLN3/HILPDA/NOL3/CA9/STC2/ANGPTL4/TREM2/VEGFA/HK2/PGF/ANGPT2/SFRP1/NOS1/ABAT/CASR/TERT/P2RX2/EPO/FMN2/FABP1/SCN2A/MGARP/MT3/BMP7/CHRNA4/MYOD1 | 26 |
| GO:0007548 | BP | GO:0007548 | sex differentiation | 23/695 | 288/18870 | 0.00044241 | 0.016848457 | 0.014355048 | VEGFA/TNFAIP6/INHBB/SFRP1/TCF21/PTPRN/ADAM18/WNT9B/TEX11/STRA6/GATA3/HSD17B3/MSH4/HOXD13/DACH2/LHX8/FGF10/LHX1/DMRT3/GATA4/FSHB/DMRT1/DMRTC2 | 23 |
| GO:0071248 | BP | GO:0071248 | cellular response to metal ion | 18/695 | 200/18870 | 0.000447802 | 0.016912863 | 0.014409923 | KCNJ10/INHBB/CPNE6/CRHBP/CHP2/AQP2/TFR2/DPEP1/MMP9/ITPKA/MT1G/SYT7/SLC34A1/SLC13A2/TF/MT1H/MT3/ADCY8 | 18 |
| GO:0042267 | BP | GO:0042267 | natural killer cell mediated cytotoxicity | 10/695 | 76/18870 | 0.000458002 | 0.017016837 | 0.01449851 | FCGR3A/PIK3R6/NKG7/HLA-G/CD2/CRTAM/LAG3/KLRK1/CLEC12B/KLRF2 | 10 |
| GO:2000242 | BP | GO:2000242 | negative regulation of reproductive process | 10/695 | 76/18870 | 0.000458002 | 0.017016837 | 0.01449851 | TEX11/SPINK13/SEMG2/ZP1/PAEP/SEMG1/HORMAD1/BMP7/DMRT1/NANOS2 | 10 |
| GO:0045236 | MF | GO:0045236 | CXCR chemokine receptor binding | 5/709 | 18/18496 | 0.000461108 | 0.009062183 | 0.007722161 | CXCL9/CXCL10/CXCL11/CXCL5/CXCL13 | 5 |
| GO:0035148 | BP | GO:0035148 | tube formation | 15/695 | 151/18870 | 0.000467888 | 0.017048064 | 0.014525116 | VEGFA/IRX2/EGF/SFRP1/WNT9B/PROX1/IRX1/HES5/GATA3/CTHRC1/LHX2/GRHL2/FGF10/OVOL2/BMP7 | 15 |
| GO:0051346 | BP | GO:0051346 | negative regulation of hydrolase activity | 19/695 | 218/18870 | 0.000470034 | 0.017048064 | 0.014525116 | NOL3/ANGPTL4/VEGFA/APOC1/PLA2R1/NOS1/HRG/GZMA/KNG1/CST7/SERPINA5/KLRK1/DPEP1/MMP9/FABP1/PPP1R1B/SERPINA12/SERPINA9/MAGEA3 | 19 |
| GO:0070374 | BP | GO:0070374 | positive regulation of ERK1 and ERK2 cascade | 19/695 | 218/18870 | 0.000470034 | 0.017048064 | 0.014525116 | TREM2/MTURN/FGF1/CCL18/CCL5/CASR/FGG/CCL20/EPO/ERBB4/MT3/FGF10/CCL25/FGA/APELA/GATA4/FGB/SLC30A10/OR2AT4 | 19 |
| GO:0007626 | BP | GO:0007626 | locomotory behavior | 18/695 | 201/18870 | 0.000475383 | 0.01707347 | 0.014546762 | KCNJ10/CALB1/SLC6A3/IDO1/ABAT/LGI4/CHL1/EN1/C1QL1/GRM1/CNTN1/PPP1R1B/PAK6/NPY2R/ADCY8/DMRT3/DMBX1/KLHL1 | 18 |
| GO:0046879 | BP | GO:0046879 | hormone secretion | 24/695 | 308/18870 | 0.000478818 | 0.01707347 | 0.014546762 | SOX11/INHBB/TRPV6/WNK4/SFRP1/PTPRN/ABAT/CCL5/CRHBP/CASR/IFNG/TFR2/TFAP2B/GATA3/FAM3B/F2/FGG/SYT7/SSTR5/IL11/SLC30A8/ADCY8/FGA/FGB | 24 |
| GO:0002765 | BP | GO:0002765 | immune response-inhibiting signal transduction | 4/695 | 11/18870 | 0.000489415 | 0.01707347 | 0.014546762 | IL20RB/LILRB4/HLA-G/CLEC12B | 4 |
| GO:0021889 | BP | GO:0021889 | olfactory bulb interneuron differentiation | 4/695 | 11/18870 | 0.000489415 | 0.01707347 | 0.014546762 | UNCX/DLX5/ERBB4/ARX | 4 |
| GO:0072178 | BP | GO:0072178 | nephric duct morphogenesis | 4/695 | 11/18870 | 0.000489415 | 0.01707347 | 0.014546762 | WNT9B/GPC3/GATA3/LHX1 | 4 |
| GO:0022824 | MF | GO:0022824 | transmitter-gated monoatomic ion channel activity | 9/709 | 61/18496 | 0.000494148 | 0.009062183 | 0.007722161 | GABRD/GRIK3/CHRNA1/CHRNA6/GRIA4/GRIK5/CHRND/GABRA2/CHRNA4 | 9 |
| GO:0022835 | MF | GO:0022835 | transmitter-gated channel activity | 9/709 | 61/18496 | 0.000494148 | 0.009062183 | 0.007722161 | GABRD/GRIK3/CHRNA1/CHRNA6/GRIA4/GRIK5/CHRND/GABRA2/CHRNA4 | 9 |
| GO:0007601 | BP | GO:0007601 | visual perception | 19/695 | 219/18870 | 0.000497423 | 0.017202546 | 0.014656736 | SEMA5B/KCNJ10/MYO3A/CLIC5/CLDN19/ABCA4/EYA4/TGFBI/OPN4/MYO3B/TACSTD2/GJA3/NR2E1/VSX1/CRYAA/RDH8/ZIC2/GUCA1C/KERA | 19 |
| GO:0061458 | BP | GO:0061458 | reproductive system development | 24/695 | 309/18870 | 0.000501589 | 0.017202546 | 0.014656736 | VEGFA/TNFAIP6/INHBB/SFRP1/TCF21/PTPRN/ADAM18/C3/WNT9B/ALOX15B/TEX11/STRA6/GATA3/HSD17B3/MSH4/HOXD13/LHX8/FGF10/HOXB13/LHX1/BMP7/GATA4/FSHB/DMRT1 | 24 |
| GO:0005261 | MF | GO:0005261 | monoatomic cation channel activity | 26/709 | 333/18496 | 0.000501787 | 0.009062183 | 0.007722161 | GRIK3/KCNJ10/SLC4A11/TRPV6/KCNJ1/TRPA1/KCNK9/KCNN1/CHRNA1/TRPV5/KCNK10/PKD2L1/P2RX2/CHRNA6/SCN7A/FXYD4/SCNN1B/KCNK17/SCN2A/SCNN1G/GRIK5/CHRND/SCN10A/CHRNA4/CACNG2/KCNU1 | 26 |
| GO:0005230 | MF | GO:0005230 | extracellular ligand-gated monoatomic ion channel activity | 10/709 | 74/18496 | 0.000503455 | 0.009062183 | 0.007722161 | GABRD/GRIK3/CHRNA1/P2RX2/CHRNA6/GRIA4/GRIK5/CHRND/GABRA2/CHRNA4 | 10 |
| GO:0055064 | BP | GO:0055064 | chloride ion homeostasis | 5/695 | 19/18870 | 0.000505153 | 0.017202546 | 0.014656736 | SLC12A1/WNK4/FASLG/UMOD/SLC12A3 | 5 |
| GO:0070098 | BP | GO:0070098 | chemokine-mediated signaling pathway | 11/695 | 91/18870 | 0.000508172 | 0.017202546 | 0.014656736 | TREM2/CCL18/CCL5/CXCL9/CXCL10/CXCL11/CXCR3/CXCL5/CXCL13/CCL20/CCL25 | 11 |
| GO:0007631 | BP | GO:0007631 | feeding behavior | 12/695 | 106/18870 | 0.000526534 | 0.017693102 | 0.015074694 | MCHR1/HCRTR2/STRA6/HELT/MRAP2/PMCH/EN1/NMUR2/TACR3/POU4F1/DMBX1/CALCA | 12 |
| GO:0032994 | CC | GO:0032994 | protein-lipid complex | 7/743 | 39/19886 | 0.000534927 | 0.014159838 | 0.012387788 | APOC1/PLA2G7/APOB/SAA1/SAA2/SAA4/APOA4 | 7 |
| GO:0001763 | BP | GO:0001763 | morphogenesis of a branching structure | 18/695 | 203/18870 | 0.000534974 | 0.017819785 | 0.015182629 | VEGFA/FGF1/EGF/SFRP1/TCF21/WNT9B/PROX1/CASR/GPC3/DLX2/HOXD13/TACSTD2/GBX2/GRHL2/FGF10/HOXB13/LHX1/BMP7 | 18 |
| GO:0072175 | BP | GO:0072175 | epithelial tube formation | 14/695 | 137/18870 | 0.000538103 | 0.017819785 | 0.015182629 | IRX2/EGF/SFRP1/WNT9B/PROX1/IRX1/HES5/GATA3/CTHRC1/LHX2/GRHL2/FGF10/OVOL2/BMP7 | 14 |
| GO:0002449 | BP | GO:0002449 | lymphocyte mediated immunity | 27/695 | 368/18870 | 0.000561383 | 0.018207518 | 0.015512982 | TREM2/CD70/FCGR3A/IL2RB/IL20RB/PIK3R6/LILRB4/NKG7/FCGR1A/C3/AICDA/HLA-G/CD8A/CD2/BATF/CRTAM/LAG3/CD27/KLRK1/SUSD4/GATA3/CLEC12B/C7/CR2/FOXJ1/KLRF2/MBL2 | 27 |
| GO:0006910 | BP | GO:0006910 | phagocytosis, recognition | 6/695 | 29/18870 | 0.000561764 | 0.018207518 | 0.015512982 | SCARB1/TREM2/FCGR1A/CRP/LBP/MBL2 | 6 |
| GO:0072207 | BP | GO:0072207 | metanephric epithelium development | 6/695 | 29/18870 | 0.000561764 | 0.018207518 | 0.015512982 | CALB1/WNT9B/UMOD/HES5/NPHS2/WNT7B | 6 |
| GO:0061138 | BP | GO:0061138 | morphogenesis of a branching epithelium | 17/695 | 187/18870 | 0.000569229 | 0.018319557 | 0.01560844 | VEGFA/FGF1/EGF/SFRP1/TCF21/WNT9B/PROX1/CASR/GPC3/HOXD13/TACSTD2/GBX2/GRHL2/FGF10/HOXB13/LHX1/BMP7 | 17 |
| GO:0005796 | CC | GO:0005796 | Golgi lumen | 12/743 | 106/19886 | 0.000599041 | 0.014362055 | 0.012564698 | GPC5/MUC3A/MUC12/UMOD/MUC15/GPC3/F2/PROZ/MUC17/MUC16/WNT7B/KERA | 12 |
| GO:0032809 | CC | GO:0032809 | neuronal cell body membrane | 6/743 | 29/19886 | 0.000606398 | 0.014362055 | 0.012564698 | SLC6A3/FLRT1/ATP2B2/UNC5A/TACR3/ADCY8 | 6 |
| GO:0045807 | BP | GO:0045807 | positive regulation of endocytosis | 15/695 | 155/18870 | 0.00061681 | 0.019712025 | 0.016794836 | TREM2/VEGFA/CD300LF/IL2RB/EGF/FCGR1A/C3/SIRPG/IFNG/GPC3/TFR2/ABCA13/TF/APELA/MBL2 | 15 |
| GO:0002228 | BP | GO:0002228 | natural killer cell mediated immunity | 10/695 | 79/18870 | 0.000626273 | 0.019738398 | 0.016817306 | FCGR3A/PIK3R6/NKG7/HLA-G/CD2/CRTAM/LAG3/KLRK1/CLEC12B/KLRF2 | 10 |
| GO:0045685 | BP | GO:0045685 | regulation of glial cell differentiation | 10/695 | 79/18870 | 0.000626273 | 0.019738398 | 0.016817306 | TREM2/DLX1/TP73/ZNF488/HES5/F2/DLX2/NKX6-2/NR2E1/SOX1 | 10 |
| GO:0044703 | BP | GO:0044703 | multi-organism reproductive process | 18/695 | 206/18870 | 0.000636408 | 0.019920432 | 0.016972401 | STC2/VEGFA/IDO1/PTHLH/ANGPT2/CLIC5/CRHBP/CYP27B1/EDDM3A/PAPPA/MMP9/EPO/SEMG1/EPYC/APELA/FSHB/CALCA/PSG6 | 18 |
| GO:0099634 | CC | GO:0099634 | postsynaptic specialization membrane | 13/743 | 122/19886 | 0.000646397 | 0.014543935 | 0.012723816 | NETO2/GRIK3/PTPRO/CHRNA1/ATP2B2/ERBB4/GRM1/GRIA4/GRIK5/CHRND/GABRA2/SORCS3/CACNG2 | 13 |
| GO:0002643 | BP | GO:0002643 | regulation of tolerance induction | 5/695 | 20/18870 | 0.000653264 | 0.020048379 | 0.017081413 | IDO1/LILRB4/HLA-G/PDCD1/FOXJ1 | 5 |
| GO:0072079 | BP | GO:0072079 | nephron tubule formation | 5/695 | 20/18870 | 0.000653264 | 0.020048379 | 0.017081413 | IRX2/WNT9B/IRX1/HES5/GATA3 | 5 |
| GO:0003002 | BP | GO:0003002 | regionalization | 30/695 | 430/18870 | 0.000653656 | 0.020048379 | 0.017081413 | IRX2/FGF1/WNT8B/DNAH11/SFRP1/C3/RFX8/EOMES/IRX1/DLX1/GPC3/HELT/HES5/DLX2/HOXB9/HOXD13/EN1/WNT7B/GBX2/LMX1B/FOXJ1/LHX2/FGF10/OVOL2/BARX1/LHX1/NKX2-5/DMRT3/SOX1/GATA4 | 30 |
| GO:0019842 | MF | GO:0019842 | vitamin binding | 15/709 | 150/18496 | 0.000657873 | 0.011566321 | 0.009856013 | EGLN3/CALB1/SDS/RBP2/ABAT/ABCA4/ALB/GADL1/AGXT/STRA6/OPN4/AFM/TTPA/FOLR3/S100G | 15 |
| GO:0021872 | BP | GO:0021872 | forebrain generation of neurons | 8/695 | 53/18870 | 0.00066532 | 0.020270072 | 0.017270297 | PROX1/DLX1/HES5/DLX2/LHX8/NR2E1/GBX2/SOX1 | 8 |
| GO:0045682 | BP | GO:0045682 | regulation of epidermis development | 9/695 | 66/18870 | 0.000670447 | 0.020290999 | 0.017288127 | ALOX15B/CYP27B1/HES5/KRT36/SULT2B1/GRHL2/OVOL2/ESRP1/REG3A | 9 |
| GO:0002507 | BP | GO:0002507 | tolerance induction | 6/695 | 30/18870 | 0.000680562 | 0.0203279 | 0.017319568 | IDO1/LILRB4/HLA-G/ICOS/PDCD1/FOXJ1 | 6 |
| GO:0060142 | BP | GO:0060142 | regulation of syncytium formation by plasma membrane fusion | 6/695 | 30/18870 | 0.000680562 | 0.0203279 | 0.017319568 | TREM2/TNFSF14/CXCL9/CXCL10/DCSTAMP/MYOD1 | 6 |
| GO:0002645 | BP | GO:0002645 | positive regulation of tolerance induction | 4/695 | 12/18870 | 0.000712798 | 0.020748313 | 0.017677764 | IDO1/LILRB4/HLA-G/FOXJ1 | 4 |
| GO:0015015 | BP | GO:0015015 | heparan sulfate proteoglycan biosynthetic process, enzymatic modification | 4/695 | 12/18870 | 0.000712798 | 0.020748313 | 0.017677764 | HS6ST2/HS3ST2/HS3ST6/HS3ST5 | 4 |
| GO:0060442 | BP | GO:0060442 | branching involved in prostate gland morphogenesis | 4/695 | 12/18870 | 0.000712798 | 0.020748313 | 0.017677764 | SFRP1/HOXD13/HOXB13/BMP7 | 4 |
| GO:0072017 | BP | GO:0072017 | distal tubule development | 4/695 | 12/18870 | 0.000712798 | 0.020748313 | 0.017677764 | CALB1/WNK4/UMOD/TFAP2B | 4 |
| GO:0071453 | BP | GO:0071453 | cellular response to oxygen levels | 16/695 | 174/18870 | 0.000722398 | 0.020803408 | 0.017724704 | EGLN3/HILPDA/NOL3/STC2/TREM2/VEGFA/SFRP1/CASR/TERT/FMN2/FABP1/SCN2A/MGARP/MT3/BMP7/MYOD1 | 16 |
| GO:0070371 | BP | GO:0070371 | ERK1 and ERK2 cascade | 25/695 | 336/18870 | 0.000724864 | 0.020803408 | 0.017724704 | TREM2/MTURN/FGF1/DUSP9/WNK4/EGF/CCL18/CCL5/CASR/LMO3/FGG/CCL20/EPO/ERBB4/TF/MT3/C1QL4/FGF10/CCL25/FGA/APELA/GATA4/FGB/SLC30A10/OR2AT4 | 25 |
| GO:0060537 | BP | GO:0060537 | muscle tissue development | 30/695 | 433/18870 | 0.000731279 | 0.020803408 | 0.017724704 | VEGFA/MYLK3/SOX11/TCF21/PROX1/LOX/NPHS1/EOMES/MYOZ2/CHRNA1/MSC/TNNT2/TP73/TNNI1/TFAP2B/STRA6/TNNC1/P2RX2/TBX5/ERBB4/MYH7/CHRND/ANKRD33/PITX1/SGCZ/POU4F1/NKX2-5/BMP7/MYOD1/GATA4 | 30 |
| GO:0007218 | BP | GO:0007218 | neuropeptide signaling pathway | 12/695 | 110/18870 | 0.000736526 | 0.020803408 | 0.017724704 | NXPH4/KISS1R/MCHR1/HCRTR2/PMCH/SSTR5/NMUR2/UTS2R/SORCS3/NPY2R/CALCA/NXPH2 | 12 |
| GO:0022407 | BP | GO:0022407 | regulation of cell-cell adhesion | 33/695 | 493/18870 | 0.000737451 | 0.020803408 | 0.017724704 | VEGFA/CD70/IDO1/IL20RB/PIK3R6/LILRB4/TNFSF9/CCL5/HLA-G/TNFSF14/CRTAM/ICOS/SIRPG/IFNG/LAG3/CD27/KLRK1/RAG2/GATA3/CXCL13/FGG/HHLA2/EPO/PLA2G2D/B4GALNT2/FOXJ1/VTCN1/CCL25/FGA/BMP7/PLG/FGB/ACTL6B | 33 |
| GO:0005788 | CC | GO:0005788 | endoplasmic reticulum lumen | 24/743 | 313/19886 | 0.000739997 | 0.015857087 | 0.013872633 | COL23A1/STC2/COL5A3/IGFBP3/RNASET2/C3/CP/KNG1/ALB/COL4A6/GPC3/SCG2/COL26A1/F2/PROZ/FGG/APOB/CHGB/TF/DMP1/WNT7B/IGFBP1/FGA/APOA4 | 24 |
| GO:0072577 | BP | GO:0072577 | endothelial cell apoptotic process | 9/695 | 67/18870 | 0.000749635 | 0.020994978 | 0.017887924 | ANGPTL4/FASLG/HLA-G/SCG2/GATA3/FGG/TERT/FGA/FGB | 9 |
| GO:0036294 | BP | GO:0036294 | cellular response to decreased oxygen levels | 15/695 | 158/18870 | 0.00075343 | 0.020994978 | 0.017887924 | EGLN3/HILPDA/NOL3/STC2/TREM2/VEGFA/SFRP1/CASR/TERT/FMN2/FABP1/SCN2A/MGARP/MT3/BMP7 | 15 |
| GO:0043410 | BP | GO:0043410 | positive regulation of MAPK cascade | 32/695 | 474/18870 | 0.000765277 | 0.021195843 | 0.018059063 | TREM2/VEGFA/MTURN/IGFBP3/FGF1/BIRC7/EGF/PIK3R6/CCL18/CCL5/CASR/AVPR1B/GDF6/EPGN/TP73/CD27/FGG/CCL20/EPO/ERBB4/GRM1/WNT7B/MT3/IL11/FGF10/CCL25/FGA/APELA/GATA4/FGB/SLC30A10/OR2AT4 | 32 |
| GO:0043027 | MF | GO:0043027 | cysteine-type endopeptidase inhibitor activity involved in apoptotic process | 5/709 | 20/18496 | 0.000782925 | 0.013452067 | 0.011462915 | NOL3/BIRC7/TNFSF14/CD27/DPEP1 | 5 |
| GO:0070664 | BP | GO:0070664 | negative regulation of leukocyte proliferation | 11/695 | 96/18870 | 0.000802433 | 0.022091064 | 0.0188218 | ENPP3/SOX11/IDO1/IL20RB/LILRB4/HLA-G/CRTAM/PLA2G2D/CRP/FOXJ1/VTCN1 | 11 |
| GO:0055081 | BP | GO:0055081 | monoatomic anion homeostasis | 5/695 | 21/18870 | 0.000831627 | 0.022748811 | 0.019382207 | SLC12A1/WNK4/FASLG/UMOD/SLC12A3 | 5 |
| GO:0030193 | BP | GO:0030193 | regulation of blood coagulation | 9/695 | 68/18870 | 0.00083628 | 0.022748811 | 0.019382207 | HRG/KNG1/F2/F11/FGG/TSPAN8/FGA/PLG/FGB | 9 |
| GO:0140828 | MF | GO:0140828 | metal cation:monoatomic cation antiporter activity | 7/709 | 41/18496 | 0.000851106 | 0.013814484 | 0.011771741 | SLC9A3/SLC9A4/SLC30A2/SLC30A8/SLC9A2/SLC4A9/SLC30A10 | 7 |
| GO:0071241 | BP | GO:0071241 | cellular response to inorganic substance | 19/695 | 229/18870 | 0.000856588 | 0.02316335 | 0.019735398 | KCNJ10/INHBB/CPNE6/CRHBP/CHP2/AQP2/TFR2/DPEP1/MMP9/ITPKA/MT1G/SYT7/SLC34A1/SLC13A2/SLC12A3/TF/MT1H/MT3/ADCY8 | 19 |
| GO:0008146 | MF | GO:0008146 | sulfotransferase activity | 8/709 | 53/18496 | 0.000862862 | 0.013814484 | 0.011771741 | HS6ST2/HS3ST2/NDST3/GAL3ST3/SULT2B1/HS3ST6/HS3ST5/SULT2A1 | 8 |
| GO:0030246 | MF | GO:0030246 | carbohydrate binding | 22/709 | 273/18496 | 0.000873026 | 0.013814484 | 0.011771741 | EGLN3/SIGLEC8/NPTX2/HK2/PLA2R1/SOST/KLRK1/KLRG2/LGALS12/ITLN1/CLEC12B/ALDOB/GAL3ST3/ITIH1/FREM1/CNTN1/PRG2/LGALS4/REG1B/KLRF2/REG3A/MBL2 | 22 |
| GO:1903035 | BP | GO:1903035 | negative regulation of response to wounding | 11/695 | 97/18870 | 0.000875612 | 0.02353851 | 0.020055038 | CLDN19/HRG/KNG1/F2/F11/FGG/TSPAN8/FGA/PLG/FGB/REG3A | 11 |
| GO:0001664 | MF | GO:0001664 | G protein-coupled receptor binding | 23/709 | 291/18496 | 0.00087711 | 0.013814484 | 0.011771741 | WNT8B/SFRP1/C3/WNT9B/CCL18/CCL5/CXCL9/CXCL10/CXCL11/PTGER1/RTP2/CXCL5/CTHRC1/CXCL13/MRAP2/PMCH/CCL20/RTP5/SAA1/WNT7B/CCL25/APELA/CALCA | 23 |
| GO:0046717 | BP | GO:0046717 | acid secretion | 7/695 | 43/18870 | 0.000904718 | 0.024178732 | 0.020600514 | ABAT/CASR/HRH2/UMOD/AGXT/SLC9A4/UGT1A3 | 7 |
| GO:0090276 | BP | GO:0090276 | regulation of peptide hormone secretion | 17/695 | 195/18870 | 0.000912967 | 0.024257311 | 0.020667463 | INHBB/SFRP1/ABAT/CCL5/CRHBP/CASR/IFNG/TFR2/TFAP2B/F2/FGG/SYT7/SSTR5/SLC30A8/ADCY8/FGA/FGB | 17 |
| GO:1903131 | BP | GO:1903131 | mononuclear cell differentiation | 32/695 | 481/18870 | 0.000975598 | 0.025731554 | 0.021923533 | TREM2/VEGFA/PIK3R6/USP44/LILRB4/TNFSF9/SFRP1/TNFRSF9/EOMES/AICDA/HLA-G/CD8A/CD2/ZNF683/BATF/CRTAM/IFNG/LAG3/CD27/UBD/RAG2/GATA3/MT1G/DCSTAMP/PLA2G2D/CR2/FOXJ1/IL11/PGLYRP2/IFNA14/ACTL6B/NKX2-3 | 32 |
| GO:0015718 | BP | GO:0015718 | monocarboxylic acid transport | 16/695 | 179/18870 | 0.000979713 | 0.025731554 | 0.021923533 | FABP6/SLC16A3/PLA2R1/FABP7/RBP2/SLC16A5/CASR/AVPR1B/SLC22A8/UGT1A3/CYP4F2/AQP9/FABP1/PLA2G2D/NMUR2/SLC22A7 | 16 |
| GO:0060541 | BP | GO:0060541 | respiratory system development | 18/695 | 214/18870 | 0.000991324 | 0.025832416 | 0.022009468 | SIM2/VEGFA/SOX11/FGF1/DLX5/TCF21/PROX1/LOX/MSC/TP73/GPC3/STRA6/TBX5/WNT7B/FOXJ1/SPDEF/GRHL2/FGF10 | 18 |
| GO:0070294 | BP | GO:0070294 | renal sodium ion absorption | 4/695 | 13/18870 | 0.000999728 | 0.025832416 | 0.022009468 | WNK4/UMOD/SLC12A3/CLCNKB | 4 |
| GO:0050920 | BP | GO:0050920 | regulation of chemotaxis | 19/695 | 232/18870 | 0.001000512 | 0.025832416 | 0.022009468 | TREM2/VEGFA/SEMA5B/TNFAIP6/FGF1/PGF/ANGPT2/PLA2G7/HRG/PTPRO/CCL5/CASR/TNFSF14/CXCL10/SCG2/KLRK1/CXCL13/FGF10/LBP | 19 |
| GO:0045785 | BP | GO:0045785 | positive regulation of cell adhesion | 32/695 | 482/18870 | 0.001009438 | 0.025916467 | 0.02208108 | VEGFA/CD70/PIK3R6/LILRB4/TNFSF9/SFRP1/HRG/CCL5/HLA-G/TNFSF14/ICOS/SIRPG/IFNG/CD27/KLRK1/COL26A1/IBSP/GATA3/CXCL13/FGG/HHLA2/EPO/OLFM4/DMP1/EPB41L4B/SAA1/VTCN1/CCL25/FGA/BMP7/FGB/ACTL6B | 32 |
| GO:0019731 | BP | GO:0019731 | antibacterial humoral response | 9/695 | 70/18870 | 0.001034014 | 0.026068751 | 0.022210828 | SEMG2/KLK7/TF/SEMG1/LGALS4/WFDC10B/FGA/KLK5/FGB | 9 |
| GO:1900046 | BP | GO:1900046 | regulation of hemostasis | 9/695 | 70/18870 | 0.001034014 | 0.026068751 | 0.022210828 | HRG/KNG1/F2/F11/FGG/TSPAN8/FGA/PLG/FGB | 9 |
| GO:0021879 | BP | GO:0021879 | forebrain neuron differentiation | 7/695 | 44/18870 | 0.001042205 | 0.026068751 | 0.022210828 | PROX1/DLX1/HES5/DLX2/LHX8/GBX2/SOX1 | 7 |
| GO:0060428 | BP | GO:0060428 | lung epithelium development | 7/695 | 44/18870 | 0.001042205 | 0.026068751 | 0.022210828 | TP73/STRA6/WNT7B/FOXJ1/SPDEF/GRHL2/FGF10 | 7 |
| GO:0030449 | BP | GO:0030449 | regulation of complement activation | 5/695 | 22/18870 | 0.001043891 | 0.026068751 | 0.022210828 | TREM2/C3/SUSD4/CD5L/CR2 | 5 |
| GO:0044298 | CC | GO:0044298 | cell body membrane | 6/743 | 32/19886 | 0.001051726 | 0.0200475 | 0.017538631 | SLC6A3/FLRT1/ATP2B2/UNC5A/TACR3/ADCY8 | 6 |
| GO:0008509 | MF | GO:0008509 | monoatomic anion transmembrane transporter activity | 14/709 | 141/18496 | 0.001053914 | 0.016260383 | 0.013855965 | GABRD/SLC6A3/SLC12A1/CLIC5/ANO4/SLC26A4/CLCNKA/SLC12A3/NMUR2/GABRA2/SLC4A9/CLCNKB/SLC4A1/BSND | 14 |
| GO:0043679 | CC | GO:0043679 | axon terminus | 12/743 | 113/19886 | 0.00106168 | 0.0200475 | 0.017538631 | GRIK3/CALB1/SLC6A3/PTPRN/CRHBP/CASR/CDH8/SCGN/SLC18A3/SNCB/CPLX2/CALCA | 12 |
| GO:0002791 | BP | GO:0002791 | regulation of peptide secretion | 17/695 | 198/18870 | 0.001081131 | 0.026588308 | 0.022653496 | INHBB/SFRP1/ABAT/CCL5/CRHBP/CASR/IFNG/TFR2/TFAP2B/F2/FGG/SYT7/SSTR5/SLC30A8/ADCY8/FGA/FGB | 17 |
| GO:0035150 | BP | GO:0035150 | regulation of tube size | 14/695 | 147/18870 | 0.00108215 | 0.026588308 | 0.022653496 | NOS1/WNT9B/KNG1/CASR/AVPR1B/HRH2/AVPR2/FGG/SCNN1B/CRP/UTS2R/FGA/FGB/CALCA | 14 |
| GO:0098656 | BP | GO:0098656 | monoatomic anion transmembrane transport | 14/695 | 147/18870 | 0.00108215 | 0.026588308 | 0.022653496 | GABRD/SLC12A1/CLIC5/CASR/ANO4/SLC26A4/CLCNKA/SLC12A3/NMUR2/GABRA2/SLC4A9/CLCNKB/SLC4A1/BSND | 14 |
| GO:0044306 | CC | GO:0044306 | neuron projection terminus | 13/743 | 129/19886 | 0.001092413 | 0.0200475 | 0.017538631 | GRIK3/CALB1/SLC6A3/PTPRN/CRHBP/CASR/CDH8/FLRT1/SCGN/SLC18A3/SNCB/CPLX2/CALCA | 13 |
| GO:0044706 | BP | GO:0044706 | multi-multicellular organism process | 18/695 | 216/18870 | 0.001102723 | 0.026929007 | 0.022943775 | STC2/VEGFA/IDO1/PTHLH/ANGPT2/CLIC5/CRHBP/CYP27B1/EDDM3A/PAPPA/MMP9/EPO/SEMG1/EPYC/APELA/FSHB/CALCA/PSG6 | 18 |
| GO:0006874 | BP | GO:0006874 | intracellular calcium ion homeostasis | 23/695 | 308/18870 | 0.001107802 | 0.026929007 | 0.022943775 | NOL3/STC2/CALB1/FATE1/NOS1/TRPA1/FASLG/CCL5/HCRTR2/TMEM178A/CASR/CXCL9/CXCL10/SCGN/CXCL11/PTH1R/UMOD/ATP2B2/F2/GRM1/GPR12/ADCY8/CALCA | 23 |
| GO:0005865 | CC | GO:0005865 | striated muscle thin filament | 5/743 | 22/19886 | 0.00111375 | 0.0200475 | 0.017538631 | TNNT2/TNNI1/TNNC1/TNNT1/ACTL8 | 5 |
| GO:0005436 | MF | GO:0005436 | sodium:phosphate symporter activity | 4/709 | 13/18496 | 0.00116039 | 0.017545097 | 0.014950709 | SLC17A4/SLC17A2/SLC34A3/SLC34A1 | 4 |
| GO:0071805 | BP | GO:0071805 | potassium ion transmembrane transport | 18/695 | 217/18870 | 0.00116231 | 0.028004764 | 0.02386033 | TREM2/KCNJ10/SLC12A1/WNK4/ATP12A/KCNJ1/KCNK9/KCNN1/KCNK10/PKD2L1/SLC9A3/SLC9A4/FXYD4/KCNK17/SLC12A3/DPP6/SLC9A2/KCNU1 | 18 |
| GO:0002443 | BP | GO:0002443 | leukocyte mediated immunity | 31/695 | 466/18870 | 0.001164312 | 0.028004764 | 0.02386033 | TREM2/CD70/FCGR3A/IL2RB/IL20RB/PIK3R6/LILRB4/NKG7/FCGR1A/C3/AICDA/HLA-G/CD8A/CD2/BATF/CRTAM/LAT/LAG3/CD27/KLRK1/SUSD4/GATA3/F2/CLEC12B/C7/SCNN1B/CR2/FOXJ1/KLRF2/CPLX2/MBL2 | 31 |
| GO:0042476 | BP | GO:0042476 | odontogenesis | 13/695 | 132/18870 | 0.00118428 | 0.028309916 | 0.024120322 | DLX1/DLX2/SLC34A1/LHX8/DMP1/FGF10/SCN10A/DMRT3/BMP7/AMELY/KLK5/KLK4/NKX2-3 | 13 |
| GO:0006775 | BP | GO:0006775 | fat-soluble vitamin metabolic process | 7/695 | 45/18870 | 0.001195583 | 0.028309916 | 0.024120322 | RBP2/CYP27B1/IFNG/UGT1A3/CYP4F2/TTPA/UGT1A4 | 7 |
| GO:0048713 | BP | GO:0048713 | regulation of oligodendrocyte differentiation | 7/695 | 45/18870 | 0.001195583 | 0.028309916 | 0.024120322 | DLX1/TP73/ZNF488/HES5/DLX2/NKX6-2/SOX1 | 7 |
| GO:0090087 | BP | GO:0090087 | regulation of peptide transport | 17/695 | 200/18870 | 0.001207291 | 0.028439786 | 0.024230973 | INHBB/SFRP1/ABAT/CCL5/CRHBP/CASR/IFNG/TFR2/TFAP2B/F2/FGG/SYT7/SSTR5/SLC30A8/ADCY8/FGA/FGB | 17 |
| GO:0003229 | BP | GO:0003229 | ventricular cardiac muscle tissue development | 8/695 | 58/18870 | 0.001224657 | 0.028700931 | 0.024453471 | PROX1/TNNT2/TNNI1/TNNC1/TBX5/MYH7/POU4F1/NKX2-5 | 8 |
| GO:0002697 | BP | GO:0002697 | regulation of immune effector process | 27/695 | 389/18870 | 0.001285659 | 0.029933393 | 0.025503541 | TREM2/ENPP3/TNFRSF4/IL20RB/PIK3R6/LILRB4/FCGR1A/C3/HLA-G/ZNF683/BATF/CRTAM/IFNG/LAG3/KLRK1/SUSD4/GATA3/CD5L/CLEC12B/KLK7/CR2/PRG2/FOXJ1/PGLYRP2/LBP/KLK5/MBL2 | 27 |
| GO:0070661 | BP | GO:0070661 | leukocyte proliferation | 25/695 | 350/18870 | 0.001290345 | 0.029933393 | 0.025503541 | TREM2/ENPP3/CD70/SOX11/TNFRSF4/IDO1/FCGR3A/IL20RB/LILRB4/TNFSF9/CCL5/TNFRSF9/HLA-G/TNFSF14/CRTAM/RAG2/HHLA2/EPO/PLA2G2D/CR2/CRP/FOXJ1/VTCN1/FGF10/IFNA14 | 25 |
| GO:0001228 | MF | GO:0001228 | DNA-binding transcription activator activity, RNA polymerase II-specific | 32/709 | 471/18496 | 0.001320027 | 0.019567466 | 0.016674031 | ESRRB/SOX11/DLX5/TCF21/PRDM16/MYBL2/BATF/EHF/TP73/TFAP2B/GATA3/ESRRG/DLX2/HOXD13/TBX5/SPIC/NR2E1/GBX2/FOXJ1/ALX1/LHX2/GRHL2/OVOL2/TFCP2L1/PITX1/POU4F1/NKX2-5/ARX/MYOD1/SOX1/GATA4/DMRT1 | 32 |
| GO:0098862 | CC | GO:0098862 | cluster of actin-based cell projections | 15/743 | 165/19886 | 0.001357804 | 0.022996487 | 0.020118564 | CALB1/IDO1/MYO3A/CLIC5/TRPA1/SLC28A2/SLC26A4/SLC17A4/ITLN1/SLC9A3/MYO3B/SLC34A3/SLC34A1/PCDH15/LHFPL5 | 15 |
| GO:0002524 | BP | GO:0002524 | hypersensitivity | 4/695 | 14/18870 | 0.001359069 | 0.03047491 | 0.025964918 | FCGR3A/IL20RB/FCGR1A/C3 | 4 |
| GO:0014733 | BP | GO:0014733 | regulation of skeletal muscle adaptation | 4/695 | 14/18870 | 0.001359069 | 0.03047491 | 0.025964918 | TNNI1/TNNC1/TNNT1/MYH7 | 4 |
| GO:0034116 | BP | GO:0034116 | positive regulation of heterotypic cell-cell adhesion | 4/695 | 14/18870 | 0.001359069 | 0.03047491 | 0.025964918 | FGG/FGA/BMP7/FGB | 4 |
| GO:0048715 | BP | GO:0048715 | negative regulation of oligodendrocyte differentiation | 4/695 | 14/18870 | 0.001359069 | 0.03047491 | 0.025964918 | DLX1/HES5/DLX2/NKX6-2 | 4 |
| GO:0050930 | BP | GO:0050930 | induction of positive chemotaxis | 4/695 | 14/18870 | 0.001359069 | 0.03047491 | 0.025964918 | VEGFA/PGF/SCG2/FGF10 | 4 |
| GO:0002323 | BP | GO:0002323 | natural killer cell activation involved in immune response | 6/695 | 34/18870 | 0.001360368 | 0.03047491 | 0.025964918 | FCGR3A/NKG7/ZNF683/PGLYRP2/KLRF2/IFNA14 | 6 |
| GO:0055075 | BP | GO:0055075 | potassium ion homeostasis | 6/695 | 34/18870 | 0.001360368 | 0.03047491 | 0.025964918 | KCNJ10/SLC12A1/ATP12A/UMOD/SCNN1B/SLC12A3 | 6 |
| GO:0071682 | CC | GO:0071682 | endocytic vesicle lumen | 5/743 | 23/19886 | 0.001379789 | 0.022996487 | 0.020118564 | GNLY/SCGB3A2/HP/APOB/SAA1 | 5 |
| GO:0048663 | BP | GO:0048663 | neuron fate commitment | 9/695 | 73/18870 | 0.001400029 | 0.031058894 | 0.026462478 | PROX1/DLX1/DLX2/NKX6-2/ISL2/POU4F1/DMRT3/ESRP1/SOX1 | 9 |
| GO:0140962 | BP | GO:0140962 | multicellular organismal-level chemical homeostasis | 9/695 | 73/18870 | 0.001400029 | 0.031058894 | 0.026462478 | VEGFA/AQP2/UMOD/TFR2/HAMP/CYP4F2/SCNN1B/SCNN1G/MBL2 | 9 |
| GO:0032943 | BP | GO:0032943 | mononuclear cell proliferation | 23/695 | 314/18870 | 0.001429001 | 0.031548481 | 0.026879611 | CD70/SOX11/TNFRSF4/IDO1/FCGR3A/IL20RB/LILRB4/TNFSF9/CCL5/TNFRSF9/HLA-G/TNFSF14/CRTAM/RAG2/HHLA2/EPO/PLA2G2D/CR2/CRP/FOXJ1/VTCN1/FGF10/IFNA14 | 23 |
| GO:0007565 | BP | GO:0007565 | female pregnancy | 16/695 | 186/18870 | 0.001467555 | 0.032243876 | 0.027472095 | STC2/VEGFA/IDO1/PTHLH/ANGPT2/CLIC5/CRHBP/CYP27B1/PAPPA/MMP9/EPO/EPYC/APELA/FSHB/CALCA/PSG6 | 16 |
| GO:0090596 | BP | GO:0090596 | sensory organ morphogenesis | 21/695 | 277/18870 | 0.001484292 | 0.032350537 | 0.027562971 | VEGFA/CALB1/SOX11/DLX5/MYO3A/PROX1/FASLG/TFAP2B/STRA6/GATA3/CTHRC1/OLFM3/MYO3B/VSX1/GBX2/FGF10/LHFPL5/LHX1/HMX2/BMP7/SOX1 | 21 |
| GO:0048568 | BP | GO:0048568 | embryonic organ development | 30/695 | 453/18870 | 0.00148766 | 0.032350537 | 0.027562971 | VEGFA/SOX11/DLX5/MYO3A/TCF21/WNT9B/PROX1/EOMES/STRA6/TBX15/GATA3/CTHRC1/DLX2/MYO3B/HOXB9/EN1/WNT7B/GBX2/TTPA/ALX1/GRHL2/FGF10/OVOL2/LHFPL5/LHX1/HMX2/NKX2-5/BMP7/APELA/GATA4 | 30 |
| GO:0016331 | BP | GO:0016331 | morphogenesis of embryonic epithelium | 14/695 | 152/18870 | 0.001493646 | 0.032350537 | 0.027562971 | IRX2/SOX11/SFRP1/WNT9B/IRX1/HES5/GATA3/CTHRC1/WNT7B/LHX2/GRHL2/FGF10/OVOL2/BMP7 | 14 |
| GO:0001216 | MF | GO:0001216 | DNA-binding transcription activator activity | 32/709 | 475/18496 | 0.00151199 | 0.021982003 | 0.018731531 | ESRRB/SOX11/DLX5/TCF21/PRDM16/MYBL2/BATF/EHF/TP73/TFAP2B/GATA3/ESRRG/DLX2/HOXD13/TBX5/SPIC/NR2E1/GBX2/FOXJ1/ALX1/LHX2/GRHL2/OVOL2/TFCP2L1/PITX1/POU4F1/NKX2-5/ARX/MYOD1/SOX1/GATA4/DMRT1 | 32 |
| GO:0009410 | BP | GO:0009410 | response to xenobiotic stimulus | 29/695 | 434/18870 | 0.001548452 | 0.032916375 | 0.02804507 | CA9/GSTM3/SLC6A3/CYP2J2/NNMT/WNK4/SFRP1/NOS1/ABAT/TRPA1/LOX/CRHBP/SLC28A2/UMOD/TP73/TFAP2B/DPEP1/AIM2/UGT1A3/SLITRK5/CYP4F2/SLC34A1/SCNN1B/CYP2B6/SLC22A7/SST/UGT1A10/GATA4/SULT2A1 | 29 |
| GO:0055010 | BP | GO:0055010 | ventricular cardiac muscle tissue morphogenesis | 7/695 | 47/18870 | 0.001555005 | 0.032916375 | 0.02804507 | PROX1/TNNT2/TNNI1/TNNC1/MYH7/POU4F1/NKX2-5 | 7 |
| GO:0002274 | BP | GO:0002274 | myeloid leukocyte activation | 19/695 | 241/18870 | 0.001562677 | 0.032916375 | 0.02804507 | TREM2/ENPP3/FCGR3A/CD300LF/WNK4/LILRB4/CCL5/CST7/CD2/BATF/LAT/IFNG/UBD/HAMP/MT1G/DCSTAMP/SCNN1B/LBP/CPLX2 | 19 |
| GO:0006813 | BP | GO:0006813 | potassium ion transport | 19/695 | 241/18870 | 0.001562677 | 0.032916375 | 0.02804507 | TREM2/KCNJ10/SLC12A1/WNK4/ATP12A/NOS1/KCNJ1/KCNK9/KCNN1/KCNK10/PKD2L1/SLC9A3/SLC9A4/FXYD4/KCNK17/SLC12A3/DPP6/SLC9A2/KCNU1 | 19 |
| GO:0030072 | BP | GO:0030072 | peptide hormone secretion | 19/695 | 241/18870 | 0.001562677 | 0.032916375 | 0.02804507 | INHBB/SFRP1/PTPRN/ABAT/CCL5/CRHBP/CASR/IFNG/TFR2/TFAP2B/FAM3B/F2/FGG/SYT7/SSTR5/SLC30A8/ADCY8/FGA/FGB | 19 |
| GO:0050866 | BP | GO:0050866 | negative regulation of cell activation | 18/695 | 223/18870 | 0.001580447 | 0.032916375 | 0.02804507 | TREM2/ENPP3/SOX11/IDO1/CD300LF/IL20RB/LILRB4/SFRP1/CST7/HLA-G/CRTAM/LAG3/RAG2/F2/PLA2G2D/FOXJ1/PGLYRP2/VTCN1 | 18 |
| GO:0046660 | BP | GO:0046660 | female sex differentiation | 12/695 | 120/18870 | 0.001583227 | 0.032916375 | 0.02804507 | VEGFA/TNFAIP6/INHBB/SFRP1/PTPRN/STRA6/MSH4/DACH2/LHX8/FGF10/LHX1/FSHB | 12 |
| GO:0060143 | BP | GO:0060143 | positive regulation of syncytium formation by plasma membrane fusion | 5/695 | 24/18870 | 0.001585306 | 0.032916375 | 0.02804507 | TREM2/TNFSF14/CXCL9/DCSTAMP/MYOD1 | 5 |
| GO:0002088 | BP | GO:0002088 | lens development in camera-type eye | 10/695 | 89/18870 | 0.001591798 | 0.032916375 | 0.02804507 | HSF4/SOX11/BIRC7/PROX1/GATA3/WNT7B/GJA8/LIM2/CRYAA/SOX1 | 10 |
| GO:0032945 | BP | GO:0032945 | negative regulation of mononuclear cell proliferation | 10/695 | 89/18870 | 0.001591798 | 0.032916375 | 0.02804507 | SOX11/IDO1/IL20RB/LILRB4/HLA-G/CRTAM/PLA2G2D/CRP/FOXJ1/VTCN1 | 10 |
| GO:0032944 | BP | GO:0032944 | regulation of mononuclear cell proliferation | 19/695 | 242/18870 | 0.001639119 | 0.033602244 | 0.028629437 | CD70/SOX11/TNFRSF4/IDO1/FCGR3A/IL20RB/LILRB4/TNFSF9/CCL5/TNFRSF9/HLA-G/CRTAM/HHLA2/EPO/PLA2G2D/CRP/FOXJ1/VTCN1/FGF10 | 19 |
| GO:0030324 | BP | GO:0030324 | lung development | 16/695 | 188/18870 | 0.001639672 | 0.033602244 | 0.028629437 | SIM2/VEGFA/SOX11/FGF1/TCF21/PROX1/LOX/TP73/GPC3/STRA6/TBX5/WNT7B/FOXJ1/SPDEF/GRHL2/FGF10 | 16 |
| GO:0031253 | CC | GO:0031253 | cell projection membrane | 25/743 | 352/19886 | 0.001706125 | 0.027419865 | 0.023988373 | CA9/DDN/MCHR1/AIF1L/SLC7A8/HLA-G/SLC28A2/UMOD/ATP2B2/DPEP1/SLC26A4/CDHR1/UNC5A/SLC17A4/ITLN1/PKD2L1/SLC9A3/SLC34A3/SLC34A1/PROM2/GABRA2/TACR3/EPS8L3/RAB25/CEACAM20 | 25 |
| GO:0015833 | BP | GO:0015833 | peptide transport | 20/695 | 262/18870 | 0.00175386 | 0.035781886 | 0.030486513 | INHBB/SFRP1/PTPRN/ABAT/CCL5/CRHBP/SLC15A2/CASR/IFNG/TFR2/TFAP2B/FAM3B/F2/FGG/SYT7/SSTR5/SLC30A8/ADCY8/FGA/FGB | 20 |
| GO:0014003 | BP | GO:0014003 | oligodendrocyte development | 7/695 | 48/18870 | 0.001763651 | 0.035821705 | 0.030520439 | KCNJ10/SOX11/ZNF488/HES5/MAL/CNTN1/NKX6-2 | 7 |
| GO:0002517 | BP | GO:0002517 | T cell tolerance induction | 4/695 | 15/18870 | 0.001799652 | 0.03623088 | 0.03086906 | IDO1/LILRB4/HLA-G/ICOS | 4 |
| GO:0072176 | BP | GO:0072176 | nephric duct development | 4/695 | 15/18870 | 0.001799652 | 0.03623088 | 0.03086906 | WNT9B/GPC3/GATA3/LHX1 | 4 |
| GO:0030316 | BP | GO:0030316 | osteoclast differentiation | 11/695 | 106/18870 | 0.001817798 | 0.036276587 | 0.030908003 | TREM2/TNFAIP6/LILRB4/SFRP1/TMEM178A/IFNG/GPC3/DCSTAMP/TF/POU4F1/FSHB | 11 |
| GO:0048709 | BP | GO:0048709 | oligodendrocyte differentiation | 11/695 | 106/18870 | 0.001817798 | 0.036276587 | 0.030908003 | KCNJ10/SOX11/DLX1/TP73/ZNF488/HES5/DLX2/MAL/CNTN1/NKX6-2/SOX1 | 11 |
| GO:0097060 | CC | GO:0097060 | synaptic membrane | 27/743 | 393/19886 | 0.001838766 | 0.028532581 | 0.024961837 | GABRD/NETO2/GRIK3/DDN/SLC6A3/PTPRO/SLC5A7/CDH8/CHRNA1/ATP2B2/KCTD8/NTNG1/CHRNA6/ERBB4/SYT7/GRM1/CNTN1/SCN2A/GRIA4/GRIK5/CHRND/GABRA2/SORCS3/SCN10A/ADCY8/CHRNA4/CACNG2 | 27 |
| GO:0005201 | MF | GO:0005201 | extracellular matrix structural constituent | 15/709 | 166/18496 | 0.001844315 | 0.026307594 | 0.022417498 | COL23A1/COL5A3/VWF/MUC3A/COL4A6/HAPLN1/TGFBI/CTHRC1/ZP1/FGG/MUC17/PRG2/FGA/AMELY/FGB | 15 |
| GO:0007159 | BP | GO:0007159 | leukocyte cell-cell adhesion | 28/695 | 419/18870 | 0.00184848 | 0.036621343 | 0.031201738 | CD70/IDO1/IL20RB/PIK3R6/LILRB4/TNFSF9/CCL5/HLA-G/TNFSF14/CRTAM/ICOS/UMOD/SIRPG/IFNG/LAG3/CD27/KLRK1/RAG2/GATA3/HHLA2/EPO/PLA2G2D/FOXJ1/VTCN1/CCL25/BMP7/ACTL6B/APOA4 | 28 |
| GO:0006882 | BP | GO:0006882 | intracellular zinc ion homeostasis | 6/695 | 36/18870 | 0.001851101 | 0.036621343 | 0.031201738 | MT1G/MT1H/SLC30A2/MT3/SLC30A8/SLC30A10 | 6 |
| GO:0019865 | MF | GO:0019865 | immunoglobulin binding | 5/709 | 24/18496 | 0.001890515 | 0.026467211 | 0.022553513 | FCGR3A/VWF/FCGR1A/HRG/UMOD | 5 |
| GO:1902105 | BP | GO:1902105 | regulation of leukocyte differentiation | 23/695 | 321/18870 | 0.001901773 | 0.037382079 | 0.031849893 | TREM2/TNFAIP6/PIK3R6/LILRB4/TNFSF9/SFRP1/TMEM178A/HLA-G/CD2/ZNF683/BATF/CRTAM/IFNG/LAG3/CD27/RAG2/GATA3/DCSTAMP/FOXJ1/PGLYRP2/POU4F1/FSHB/ACTL6B | 23 |
| GO:1902495 | CC | GO:1902495 | transmembrane transporter complex | 27/743 | 394/19886 | 0.001906223 | 0.028593346 | 0.025014997 | GABRD/GRIK3/HSPA2/TRPV6/ATP12A/CLIC5/KCNN1/CHRNA1/PKD2L1/CLCNKA/OLFM3/CHRNA6/SCN7A/FXYD4/SCNN1B/SCN2A/GRIA4/SCNN1G/GRIK5/CHRND/DPP6/GABRA2/SCN10A/CHRNA4/CATSPERD/CLCNKB/CACNG2 | 27 |
| GO:0050927 | BP | GO:0050927 | positive regulation of positive chemotaxis | 5/695 | 25/18870 | 0.001922273 | 0.037382079 | 0.031849893 | VEGFA/PGF/CASR/SCG2/FGF10 | 5 |
| GO:0072170 | BP | GO:0072170 | metanephric tubule development | 5/695 | 25/18870 | 0.001922273 | 0.037382079 | 0.031849893 | CALB1/WNT9B/UMOD/HES5/WNT7B | 5 |
| GO:0072243 | BP | GO:0072243 | metanephric nephron epithelium development | 5/695 | 25/18870 | 0.001922273 | 0.037382079 | 0.031849893 | CALB1/UMOD/HES5/NPHS2/WNT7B | 5 |
| GO:0002790 | BP | GO:0002790 | peptide secretion | 19/695 | 246/18870 | 0.001977376 | 0.038290719 | 0.032624063 | INHBB/SFRP1/PTPRN/ABAT/CCL5/CRHBP/CASR/IFNG/TFR2/TFAP2B/FAM3B/F2/FGG/SYT7/SSTR5/SLC30A8/ADCY8/FGA/FGB | 19 |
| GO:0046850 | BP | GO:0046850 | regulation of bone remodeling | 7/695 | 49/18870 | 0.001993387 | 0.038437889 | 0.032749453 | SFRP1/HAMP/DCSTAMP/SYT7/TF/FSHB/CALCA | 7 |
| GO:0001909 | BP | GO:0001909 | leukocyte mediated cytotoxicity | 13/695 | 140/18870 | 0.002020044 | 0.038788231 | 0.033047948 | FCGR3A/PIK3R6/NKG7/FCGR1A/HLA-G/CD2/CRTAM/LAG3/KLRK1/F2/CLEC12B/SCNN1B/KLRF2 | 13 |
| GO:0042379 | MF | GO:0042379 | chemokine receptor binding | 9/709 | 74/18496 | 0.002026482 | 0.026701459 | 0.022753123 | CCL18/CCL5/CXCL9/CXCL10/CXCL11/CXCL5/CXCL13/CCL20/CCL25 | 9 |
| GO:0030323 | BP | GO:0030323 | respiratory tube development | 16/695 | 192/18870 | 0.002035033 | 0.03891255 | 0.033153869 | SIM2/VEGFA/SOX11/FGF1/TCF21/PROX1/LOX/TP73/GPC3/STRA6/TBX5/WNT7B/FOXJ1/SPDEF/GRHL2/FGF10 | 16 |
| GO:0008237 | MF | GO:0008237 | metallopeptidase activity | 16/709 | 185/18496 | 0.002078045 | 0.026701459 | 0.022753123 | ADAM18/XPNPEP2/AMZ1/ADAMDEC1/PAPPA/ADAMTS14/DPEP1/MMP9/KLK7/ADAMTS20/MEP1A/CPB1/ADAMTS19/PAPPA2/MMP13/AGBL1 | 16 |
| GO:0004089 | MF | GO:0004089 | carbonate dehydratase activity | 4/709 | 15/18496 | 0.002083844 | 0.026701459 | 0.022753123 | CA9/CA8/CA10/CA1 | 4 |
| GO:0070492 | MF | GO:0070492 | oligosaccharide binding | 4/709 | 15/18496 | 0.002083844 | 0.026701459 | 0.022753123 | LGALS12/ITLN1/REG1B/REG3A | 4 |
| GO:0099507 | MF | GO:0099507 | ligand-gated monoatomic ion channel activity involved in regulation of presynaptic membrane potential | 4/709 | 15/18496 | 0.002083844 | 0.026701459 | 0.022753123 | GRIK3/GRIA4/GRIK5/GABRA2 | 4 |
| GO:0072676 | BP | GO:0072676 | lymphocyte migration | 12/695 | 124/18870 | 0.002094205 | 0.039624693 | 0.033760622 | CCL18/CCL5/TNFSF14/CXCL10/CRTAM/CXCL11/KLRK1/GATA3/CXCL13/CCL20/SAA1/CCL25 | 12 |
| GO:0000768 | BP | GO:0000768 | syncytium formation by plasma membrane fusion | 8/695 | 63/18870 | 0.002106959 | 0.039624693 | 0.033760622 | TREM2/NOS1/NPHS1/TNFSF14/CXCL9/CXCL10/DCSTAMP/MYOD1 | 8 |
| GO:0140253 | BP | GO:0140253 | cell-cell fusion | 8/695 | 63/18870 | 0.002106959 | 0.039624693 | 0.033760622 | TREM2/NOS1/NPHS1/TNFSF14/CXCL9/CXCL10/DCSTAMP/MYOD1 | 8 |
| GO:2000179 | BP | GO:2000179 | positive regulation of neural precursor cell proliferation | 8/695 | 63/18870 | 0.002106959 | 0.039624693 | 0.033760622 | VEGFA/EGF/PROX1/HAPLN1/RASSF10/NR2E1/LHX2/LHX1 | 8 |
| GO:0050817 | BP | GO:0050817 | coagulation | 18/695 | 229/18870 | 0.00211932 | 0.03969382 | 0.033819518 | VWF/HRG/KNG1/TREML1/SEMG2/F2/PROZ/F11/FGG/CYP4F2/TSPAN8/SEMG1/SAA1/HS3ST5/FGA/PLG/SLC4A1/FGB | 18 |
| GO:0033280 | BP | GO:0033280 | response to vitamin D | 6/695 | 37/18870 | 0.002141643 | 0.039948206 | 0.034036258 | STC2/SFRP1/CASR/CXCL10/CYP27B1/BMP7 | 6 |
| GO:0070663 | BP | GO:0070663 | regulation of leukocyte proliferation | 20/695 | 267/18870 | 0.002190021 | 0.040684528 | 0.034663611 | ENPP3/CD70/SOX11/TNFRSF4/IDO1/FCGR3A/IL20RB/LILRB4/TNFSF9/CCL5/TNFRSF9/HLA-G/CRTAM/HHLA2/EPO/PLA2G2D/CRP/FOXJ1/VTCN1/FGF10 | 20 |
| GO:0045619 | BP | GO:0045619 | regulation of lymphocyte differentiation | 17/695 | 212/18870 | 0.002255993 | 0.040987325 | 0.034921597 | PIK3R6/LILRB4/TNFSF9/SFRP1/HLA-G/CD2/ZNF683/BATF/CRTAM/IFNG/LAG3/CD27/RAG2/GATA3/FOXJ1/PGLYRP2/ACTL6B | 17 |
| GO:0048839 | BP | GO:0048839 | inner ear development | 16/695 | 194/18870 | 0.00226079 | 0.040987325 | 0.034921597 | ESRRB/CALB1/DLX5/MYO3A/PROX1/EYA4/HES5/GATA3/CTHRC1/MYO3B/GBX2/FGF10/PCDH15/LHFPL5/HMX2/ESRP1 | 16 |
| GO:0030280 | MF | GO:0030280 | structural constituent of skin epidermis | 6/709 | 36/18496 | 0.002263961 | 0.028375028 | 0.024179222 | KRT81/KRT36/KRT72/KRT6A/KRT78/KRT6B | 6 |
| GO:0051960 | BP | GO:0051960 | regulation of nervous system development | 30/695 | 466/18870 | 0.002282827 | 0.040987325 | 0.034921597 | TREM2/VEGFA/SEMA5B/SOX11/ITGAX/PROX1/LGI4/CST7/DLX1/HAPLN1/IFNG/CDH4/TP73/ZNF488/HELT/HES5/ITPKA/F2/DLX2/RASSF10/SLITRK5/NKX6-2/NR2E1/GBX2/CBLN2/MT3/LHX2/L1CAM/POU4F1/BMP7 | 30 |
| GO:0051139 | MF | GO:0051139 | metal cation:proton antiporter activity | 5/709 | 25/18496 | 0.002289519 | 0.028375028 | 0.024179222 | SLC9A3/SLC9A4/SLC30A2/SLC30A8/SLC9A2 | 5 |
| GO:0008544 | BP | GO:0008544 | epidermis development | 26/695 | 385/18870 | 0.002290348 | 0.040987325 | 0.034921597 | PTHLH/RBP2/ALOX15B/CYP27B1/HES5/KRT81/KLK7/KRT36/SULT2B1/KRT32/KRT72/LHX2/GRHL2/FGF10/KRT25/OVOL2/LHFPL5/KRT6A/HOXB13/ESRP1/KLK5/KRT78/REG3A/KRT6B/SPRR3/LCE2D | 26 |
| GO:0031045 | CC | GO:0031045 | dense core granule | 6/743 | 37/19886 | 0.002303253 | 0.031613272 | 0.027656991 | CRHBP/SCG2/P2RX2/SYT7/SST/CALCA | 6 |
| GO:0032421 | CC | GO:0032421 | stereocilium bundle | 8/743 | 63/19886 | 0.002305769 | 0.031613272 | 0.027656991 | CALB1/IDO1/MYO3A/CLIC5/TRPA1/MYO3B/PCDH15/LHFPL5 | 8 |
| GO:0002438 | BP | GO:0002438 | acute inflammatory response to antigenic stimulus | 5/695 | 26/18870 | 0.002308737 | 0.040987325 | 0.034921597 | FCGR3A/IL20RB/FCGR1A/C3/PLA2G2D | 5 |
| GO:0060740 | BP | GO:0060740 | prostate gland epithelium morphogenesis | 5/695 | 26/18870 | 0.002308737 | 0.040987325 | 0.034921597 | SFRP1/HOXD13/FGF10/HOXB13/BMP7 | 5 |
| GO:1901623 | BP | GO:1901623 | regulation of lymphocyte chemotaxis | 5/695 | 26/18870 | 0.002308737 | 0.040987325 | 0.034921597 | CCL5/TNFSF14/CXCL10/KLRK1/CXCL13 | 5 |
| GO:0002864 | BP | GO:0002864 | regulation of acute inflammatory response to antigenic stimulus | 4/695 | 16/18870 | 0.002330198 | 0.040987325 | 0.034921597 | IL20RB/FCGR1A/C3/PLA2G2D | 4 |
| GO:0003096 | BP | GO:0003096 | renal sodium ion transport | 4/695 | 16/18870 | 0.002330198 | 0.040987325 | 0.034921597 | WNK4/UMOD/SLC12A3/CLCNKB | 4 |
| GO:0010819 | BP | GO:0010819 | regulation of T cell chemotaxis | 4/695 | 16/18870 | 0.002330198 | 0.040987325 | 0.034921597 | CCL5/TNFSF14/CXCL10/CXCL13 | 4 |
| GO:0030201 | BP | GO:0030201 | heparan sulfate proteoglycan metabolic process | 4/695 | 16/18870 | 0.002330198 | 0.040987325 | 0.034921597 | HS6ST2/HS3ST2/HS3ST6/HS3ST5 | 4 |
| GO:0048712 | BP | GO:0048712 | negative regulation of astrocyte differentiation | 4/695 | 16/18870 | 0.002330198 | 0.040987325 | 0.034921597 | TREM2/HES5/F2/NR2E1 | 4 |
| GO:2000696 | BP | GO:2000696 | regulation of epithelial cell differentiation involved in kidney development | 4/695 | 16/18870 | 0.002330198 | 0.040987325 | 0.034921597 | WNT9B/MMP9/GATA3/LHX1 | 4 |
| GO:1904036 | BP | GO:1904036 | negative regulation of epithelial cell apoptotic process | 8/695 | 64/18870 | 0.002331883 | 0.040987325 | 0.034921597 | ANGPTL4/SCG2/GATA3/FGG/TERT/NKX2-5/FGA/FGB | 8 |
| GO:0046651 | BP | GO:0046651 | lymphocyte proliferation | 22/695 | 307/18870 | 0.002356369 | 0.04125903 | 0.035153093 | CD70/SOX11/TNFRSF4/IDO1/FCGR3A/IL20RB/LILRB4/TNFSF9/CCL5/TNFRSF9/HLA-G/TNFSF14/CRTAM/RAG2/HHLA2/EPO/PLA2G2D/CR2/FOXJ1/VTCN1/FGF10/IFNA14 | 22 |
| GO:0003018 | BP | GO:0003018 | vascular process in circulatory system | 20/695 | 269/18870 | 0.002388465 | 0.041595145 | 0.035439466 | VEGFA/MYLK3/NOS1/SLC7A8/KNG1/SLC15A2/CASR/SLC28A2/AVPR1B/HRH2/AVPR2/SLC22A8/SLC13A3/FGG/SCNN1B/CRP/UTS2R/FGA/FGB/CALCA | 20 |
| GO:2000027 | BP | GO:2000027 | regulation of animal organ morphogenesis | 12/695 | 126/18870 | 0.002396212 | 0.041595145 | 0.035439466 | VEGFA/FGF1/SFRP1/WNT9B/GPC3/GATA3/CTHRC1/TACSTD2/FGF10/LHX1/DMRT3/BMP7 | 12 |
| GO:0045104 | BP | GO:0045104 | intermediate filament cytoskeleton organization | 10/695 | 94/18870 | 0.002402871 | 0.041595145 | 0.035439466 | KRT81/KRT36/KRT32/KRT72/KRT40/KRT25/KRT6A/KRT78/KRT20/KRT6B | 10 |
| GO:1990351 | CC | GO:1990351 | transporter complex | 28/743 | 421/19886 | 0.002443974 | 0.031613272 | 0.027656991 | GABRD/GRIK3/HSPA2/TRPV6/ATP12A/CLIC5/ATP8B3/KCNN1/CHRNA1/PKD2L1/CLCNKA/OLFM3/CHRNA6/SCN7A/FXYD4/SCNN1B/SCN2A/GRIA4/SCNN1G/GRIK5/CHRND/DPP6/GABRA2/SCN10A/CHRNA4/CATSPERD/CLCNKB/CACNG2 | 28 |
| GO:0030900 | BP | GO:0030900 | forebrain development | 27/695 | 407/18870 | 0.002447152 | 0.042133558 | 0.035898199 | SLC6A3/INHBB/UNCX/DLX5/PROX1/HTR6/EOMES/DLX1/AVPR2/PCDH9/HES5/DLX2/SLITRK5/ERBB4/LHX8/SCN2A/NR2E1/MGARP/WNT7B/GBX2/LHX2/FGF10/PITX1/POU4F1/LHX1/ARX/SOX1 | 27 |
| GO:0015850 | BP | GO:0015850 | organic hydroxy compound transport | 22/695 | 308/18870 | 0.002452413 | 0.042133558 | 0.035898199 | SCARB1/TREM2/SLC16A3/APOC1/SLC6A3/WNK4/EGF/NOS1/ABAT/ABCA4/AQP2/CEL/STRA6/APOB/SLC5A11/CHRNA6/AQP9/SYT7/SLC18A3/ABCA13/CHRNA4/APOA4 | 22 |
| GO:0005892 | CC | GO:0005892 | acetylcholine-gated channel complex | 4/743 | 16/19886 | 0.002456109 | 0.031613272 | 0.027656991 | CHRNA1/CHRNA6/CHRND/CHRNA4 | 4 |
| GO:0036379 | CC | GO:0036379 | myofilament | 5/743 | 26/19886 | 0.00245881 | 0.031613272 | 0.027656991 | TNNT2/TNNI1/TNNC1/TNNT1/ACTL8 | 5 |
| GO:0046883 | BP | GO:0046883 | regulation of hormone secretion | 19/695 | 251/18870 | 0.00248135 | 0.04247104 | 0.036185737 | SOX11/INHBB/SFRP1/ABAT/CCL5/CRHBP/CASR/IFNG/TFR2/TFAP2B/F2/FGG/SYT7/SSTR5/IL11/SLC30A8/ADCY8/FGA/FGB | 19 |
| GO:0010001 | BP | GO:0010001 | glial cell differentiation | 19/695 | 252/18870 | 0.0025941 | 0.044156758 | 0.037621985 | TREM2/KCNJ10/SOX11/LGI4/EOMES/DLX1/HAPLN1/IFNG/TP73/ZNF488/HES5/F2/DLX2/MAL/CNTN1/NKX6-2/NR2E1/FGF10/SOX1 | 19 |
| GO:0045103 | BP | GO:0045103 | intermediate filament-based process | 10/695 | 95/18870 | 0.002599161 | 0.044156758 | 0.037621985 | KRT81/KRT36/KRT32/KRT72/KRT40/KRT25/KRT6A/KRT78/KRT20/KRT6B | 10 |
| GO:0004869 | MF | GO:0004869 | cysteine-type endopeptidase inhibitor activity | 6/709 | 37/18496 | 0.002616 | 0.031898321 | 0.027181526 | BIRC7/HRG/KNG1/CST7/CST9/LCN1 | 6 |
| GO:0005507 | MF | GO:0005507 | copper ion binding | 8/709 | 63/18496 | 0.002697131 | 0.032365568 | 0.027579682 | LOX/CP/ALB/HAMP/PRND/MT3/SNCB/APOA4 | 8 |
| GO:0010951 | BP | GO:0010951 | negative regulation of endopeptidase activity | 12/695 | 128/18870 | 0.002732813 | 0.045278494 | 0.038577715 | NOL3/VEGFA/HRG/KNG1/CST7/SERPINA5/DPEP1/MMP9/FABP1/SERPINA12/SERPINA9/MAGEA3 | 12 |
| GO:0007274 | BP | GO:0007274 | neuromuscular synaptic transmission | 5/695 | 27/18870 | 0.002748743 | 0.045278494 | 0.038577715 | SLC5A7/CHRNA1/RIMBP2/P2RX2/CHAT | 5 |
| GO:0010818 | BP | GO:0010818 | T cell chemotaxis | 5/695 | 27/18870 | 0.002748743 | 0.045278494 | 0.038577715 | CCL5/TNFSF14/CXCL10/CXCL11/CXCL13 | 5 |
| GO:0021895 | BP | GO:0021895 | cerebral cortex neuron differentiation | 5/695 | 27/18870 | 0.002748743 | 0.045278494 | 0.038577715 | EOMES/DLX1/DLX2/NR2E1/ARX | 5 |
| GO:0022010 | BP | GO:0022010 | central nervous system myelination | 5/695 | 27/18870 | 0.002748743 | 0.045278494 | 0.038577715 | KCNJ10/HES5/MAL/CNTN1/NKX6-2 | 5 |
| GO:0032291 | BP | GO:0032291 | axon ensheathment in central nervous system | 5/695 | 27/18870 | 0.002748743 | 0.045278494 | 0.038577715 | KCNJ10/HES5/MAL/CNTN1/NKX6-2 | 5 |
| GO:0042133 | BP | GO:0042133 | neurotransmitter metabolic process | 5/695 | 27/18870 | 0.002748743 | 0.045278494 | 0.038577715 | NOS1/ABAT/SLC5A7/PRIMA1/CHAT | 5 |
| GO:0045745 | BP | GO:0045745 | positive regulation of G protein-coupled receptor signaling pathway | 5/695 | 27/18870 | 0.002748743 | 0.045278494 | 0.038577715 | C3/F2/MRAP2/KLK6/KLK5 | 5 |
| GO:0030534 | BP | GO:0030534 | adult behavior | 13/695 | 145/18870 | 0.002754359 | 0.045278494 | 0.038577715 | KCNJ10/ABAT/CRHBP/LGI4/SPTBN2/CHL1/EN1/SLITRK5/PPP1R1B/DMRT3/CHRNA4/DMBX1/KLHL1 | 13 |
| GO:0045664 | BP | GO:0045664 | regulation of neuron differentiation | 16/695 | 198/18870 | 0.002775169 | 0.045457073 | 0.038729866 | SOX11/SFRP1/PROX1/SH3GL3/DLX1/GDF6/TP73/HES5/DLX2/EPO/NR2E1/ISL2/BRINP3/NKX2-5/ESRP1/BMP7 | 16 |
| GO:0002573 | BP | GO:0002573 | myeloid leukocyte differentiation | 18/695 | 235/18870 | 0.002804814 | 0.045668831 | 0.038910286 | TREM2/VEGFA/TNFAIP6/LILRB4/SFRP1/TMEM178A/BATF/IFNG/GPC3/UBD/MMP9/GATA3/MT1G/DCSTAMP/TF/POU4F1/FSHB/NKX2-3 | 18 |
| GO:0045666 | BP | GO:0045666 | positive regulation of neuron differentiation | 10/695 | 96/18870 | 0.002808083 | 0.045668831 | 0.038910286 | SOX11/PROX1/SH3GL3/DLX1/GDF6/DLX2/EPO/BRINP3/NKX2-5/BMP7 | 10 |
| GO:0060688 | BP | GO:0060688 | regulation of morphogenesis of a branching structure | 7/695 | 52/18870 | 0.002823266 | 0.045752926 | 0.038981936 | VEGFA/SFRP1/HOXD13/TACSTD2/FGF10/LHX1/BMP7 | 7 |
| GO:0007162 | BP | GO:0007162 | negative regulation of cell adhesion | 22/695 | 312/18870 | 0.002870383 | 0.046352114 | 0.039492449 | CDKN2A/VEGFA/IDO1/IL20RB/ANGPT2/LILRB4/HRG/PTPRO/KNG1/HLA-G/CRTAM/ADAMDEC1/LAG3/TGFBI/RAG2/TACSTD2/PLA2G2D/B4GALNT2/FOXJ1/VTCN1/CCL25/PLG | 22 |
| GO:0021953 | BP | GO:0021953 | central nervous system neuron differentiation | 15/695 | 181/18870 | 0.002919426 | 0.046569665 | 0.039677805 | PROX1/EOMES/DLX1/HES5/DLX2/LHX8/NR2E1/GBX2/ISL2/POU4F1/BRINP3/LHX1/ARX/DMRT3/SOX1 | 15 |
| GO:0045580 | BP | GO:0045580 | regulation of T cell differentiation | 15/695 | 181/18870 | 0.002919426 | 0.046569665 | 0.039677805 | PIK3R6/LILRB4/TNFSF9/HLA-G/CD2/ZNF683/BATF/CRTAM/IFNG/LAG3/CD27/RAG2/GATA3/FOXJ1/ACTL6B | 15 |
| GO:0035296 | BP | GO:0035296 | regulation of tube diameter | 13/695 | 146/18870 | 0.002924616 | 0.046569665 | 0.039677805 | NOS1/KNG1/CASR/AVPR1B/HRH2/AVPR2/FGG/SCNN1B/CRP/UTS2R/FGA/FGB/CALCA | 13 |
| GO:0097746 | BP | GO:0097746 | blood vessel diameter maintenance | 13/695 | 146/18870 | 0.002924616 | 0.046569665 | 0.039677805 | NOS1/KNG1/CASR/AVPR1B/HRH2/AVPR2/FGG/SCNN1B/CRP/UTS2R/FGA/FGB/CALCA | 13 |
| GO:0048557 | BP | GO:0048557 | embryonic digestive tract morphogenesis | 4/695 | 17/18870 | 0.002959245 | 0.046957468 | 0.040008217 | SOX11/TCF21/FGF10/OVOL2 | 4 |
| GO:0034765 | BP | GO:0034765 | regulation of monoatomic ion transmembrane transport | 29/695 | 454/18870 | 0.00299685 | 0.047389628 | 0.040376422 | TREM2/KCNJ10/WNK4/CLIC5/NOS1/KCNJ1/CHP2/CXCL9/CXCL10/KCNK10/CXCL11/IFNG/HAMP/HECW1/MMP9/F2/CLCNKA/P2RX2/EPO/SCN7A/FXYD4/KCNK17/SCN2A/DPP6/SCN10A/SLN/CLCNKB/CACNG2/KCNU1 | 29 |
| GO:0005501 | MF | GO:0005501 | retinoid binding | 6/709 | 38/18496 | 0.003007542 | 0.034484905 | 0.029385633 | RBP2/ABCA4/SERPINA5/STRA6/OPN4/UGT1A3 | 6 |
| GO:0019840 | MF | GO:0019840 | isoprenoid binding | 6/709 | 38/18496 | 0.003007542 | 0.034484905 | 0.029385633 | RBP2/ABCA4/SERPINA5/STRA6/OPN4/UGT1A3 | 6 |
| GO:0050679 | BP | GO:0050679 | positive regulation of epithelial cell proliferation | 17/695 | 218/18870 | 0.003016132 | 0.047406868 | 0.04039111 | VEGFA/SOX11/FGF1/PGF/EGF/DLX5/SFRP1/PTPRN/PROX1/EPGN/SCG2/FGF10/HMX2/NKX2-5/APELA/REG3A/FGFBP1 | 17 |
| GO:1904035 | BP | GO:1904035 | regulation of epithelial cell apoptotic process | 11/695 | 113/18870 | 0.003018687 | 0.047406868 | 0.04039111 | ANGPTL4/PLA2R1/FASLG/HLA-G/SCG2/GATA3/FGG/TERT/NKX2-5/FGA/FGB | 11 |
| GO:0009953 | BP | GO:0009953 | dorsal/ventral pattern formation | 10/695 | 97/18870 | 0.003030207 | 0.047424809 | 0.040406396 | WNT8B/SFRP1/RFX8/EN1/LMX1B/LHX2/OVOL2/LHX1/DMRT3/SOX1 | 10 |
| GO:0030414 | MF | GO:0030414 | peptidase inhibitor activity | 15/709 | 175/18496 | 0.003076297 | 0.034484905 | 0.029385633 | BIRC7/C3/HRG/GBP5/KNG1/CST7/SERPINA5/SPINK13/GPC3/CST9/ITIH1/LCN1/WFDC10B/SERPINA12/SERPINA9 | 15 |
| GO:0061134 | MF | GO:0061134 | peptidase regulator activity | 18/709 | 228/18496 | 0.00309122 | 0.034484905 | 0.029385633 | BIRC7/C3/HRG/GBP5/KNG1/CST7/SERPINA5/SPINK13/GPC3/CST9/AIM2/ITIH1/MAL/LCN1/WFDC10B/SERPINA12/PRSS22/SERPINA9 | 18 |
| GO:0008528 | MF | GO:0008528 | G protein-coupled peptide receptor activity | 12/709 | 125/18496 | 0.003101817 | 0.034484905 | 0.029385633 | KISS1R/MCHR1/HCRTR2/AVPR1B/AVPR2/PTH1R/SSTR5/NMUR2/TACR3/UTS2R/SORCS3/NPY2R | 12 |
| GO:0006949 | BP | GO:0006949 | syncytium formation | 8/695 | 67/18870 | 0.003121087 | 0.04851486 | 0.04133513 | TREM2/NOS1/NPHS1/TNFSF14/CXCL9/CXCL10/DCSTAMP/MYOD1 | 8 |
| GO:0060675 | BP | GO:0060675 | ureteric bud morphogenesis | 8/695 | 67/18870 | 0.003121087 | 0.04851486 | 0.04133513 | VEGFA/FGF1/TCF21/WNT9B/GPC3/GATA3/TACSTD2/LHX1 | 8 |
| GO:0050670 | BP | GO:0050670 | regulation of lymphocyte proliferation | 18/695 | 238/18870 | 0.003211544 | 0.04961825 | 0.042275229 | CD70/SOX11/TNFRSF4/IDO1/FCGR3A/IL20RB/LILRB4/TNFSF9/CCL5/TNFRSF9/HLA-G/CRTAM/HHLA2/EPO/PLA2G2D/FOXJ1/VTCN1/FGF10 | 18 |
| GO:0045616 | BP | GO:0045616 | regulation of keratinocyte differentiation | 6/695 | 40/18870 | 0.00322113 | 0.04961825 | 0.042275229 | ALOX15B/CYP27B1/KRT36/GRHL2/OVOL2/REG3A | 6 |
| GO:0060479 | BP | GO:0060479 | lung cell differentiation | 5/695 | 28/18870 | 0.003246358 | 0.04961825 | 0.042275229 | TP73/FOXJ1/SPDEF/GRHL2/FGF10 | 5 |
| GO:0060487 | BP | GO:0060487 | lung epithelial cell differentiation | 5/695 | 28/18870 | 0.003246358 | 0.04961825 | 0.042275229 | TP73/FOXJ1/SPDEF/GRHL2/FGF10 | 5 |
| GO:0060512 | BP | GO:0060512 | prostate gland morphogenesis | 5/695 | 28/18870 | 0.003246358 | 0.04961825 | 0.042275229 | SFRP1/HOXD13/FGF10/HOXB13/BMP7 | 5 |
| GO:0006835 | BP | GO:0006835 | dicarboxylic acid transport | 10/695 | 98/18870 | 0.003266113 | 0.049753789 | 0.04239071 | KCNJ10/ABAT/AGXT/SLC26A4/SLC13A3/GRM1/SLC13A2/FOLR3/SLC7A13/SLC22A7 | 10 |
| GO:0004222 | MF | GO:0004222 | metalloendopeptidase activity | 11/709 | 110/18496 | 0.003315207 | 0.036323133 | 0.030952043 | ADAM18/ADAMDEC1/PAPPA/ADAMTS14/MMP9/KLK7/ADAMTS20/MEP1A/ADAMTS19/PAPPA2/MMP13 | 11 |
| GO:1903522 | BP | GO:1903522 | regulation of blood circulation | 19/695 | 258/18870 | 0.003364574 | 0.051083394 | 0.043523546 | CYP2J2/NOS1/CASR/AVPR1B/HRH2/TNNT2/AVPR2/ATP2B2/FGG/HSPB7/TBX5/MYH7/TACR3/SCN10A/NKX2-5/FGA/APELA/GATA4/FGB | 19 |
| GO:0002695 | BP | GO:0002695 | negative regulation of leukocyte activation | 16/695 | 202/18870 | 0.003382971 | 0.051192648 | 0.043616631 | ENPP3/SOX11/IDO1/CD300LF/IL20RB/LILRB4/SFRP1/CST7/HLA-G/CRTAM/LAG3/RAG2/PLA2G2D/FOXJ1/PGLYRP2/VTCN1 | 16 |
| GO:0034185 | MF | GO:0034185 | apolipoprotein binding | 4/709 | 17/18496 | 0.003418364 | 0.036918331 | 0.03145923 | SCARB1/TREM2/LILRB4/PLG | 4 |
| GO:0072171 | BP | GO:0072171 | mesonephric tubule morphogenesis | 8/695 | 68/18870 | 0.003425728 | 0.051516442 | 0.043892507 | VEGFA/FGF1/TCF21/WNT9B/GPC3/GATA3/TACSTD2/LHX1 | 8 |
| GO:0042310 | BP | GO:0042310 | vasoconstriction | 9/695 | 83/18870 | 0.003426914 | 0.051516442 | 0.043892507 | CASR/AVPR1B/HRH2/AVPR2/FGG/SCNN1B/CRP/FGA/FGB | 9 |
| GO:0000795 | CC | GO:0000795 | synaptonemal complex | 6/743 | 40/19886 | 0.00345944 | 0.042074265 | 0.03680883 | HSPA2/RNF212B/TEX11/MSH4/HORMAD1/FAM9A | 6 |
| GO:0099086 | CC | GO:0099086 | synaptonemal structure | 6/743 | 40/19886 | 0.00345944 | 0.042074265 | 0.03680883 | HSPA2/RNF212B/TEX11/MSH4/HORMAD1/FAM9A | 6 |
| GO:0043583 | BP | GO:0043583 | ear development | 17/695 | 221/18870 | 0.003469436 | 0.05198467 | 0.044291442 | ESRRB/CALB1/DLX5/MYO3A/PROX1/EYA4/STRA6/HES5/GATA3/CTHRC1/MYO3B/GBX2/FGF10/PCDH15/LHFPL5/HMX2/ESRP1 | 17 |
| GO:0050768 | BP | GO:0050768 | negative regulation of neurogenesis | 13/695 | 149/18870 | 0.003487567 | 0.052085558 | 0.044377399 | TREM2/SEMA5B/SOX11/PROX1/DLX1/HES5/F2/DLX2/NKX6-2/NR2E1/MT3/LHX2/BMP7 | 13 |
| GO:0043086 | BP | GO:0043086 | negative regulation of catalytic activity | 30/695 | 480/18870 | 0.003522688 | 0.052438709 | 0.044678287 | NOL3/CDKN2A/ANGPTL4/VEGFA/APOC1/PLA2R1/USP44/LILRB4/NOS1/HRG/PTPRO/GZMA/KNG1/CST7/TRIB3/CYP27B1/IFNG/SERPINA5/KLRK1/DPEP1/MMP9/HP/FABP1/PTPRQ/PPP1R1B/MT3/SERPINA12/SERPINA9/SLN/MAGEA3 | 30 |
| GO:0010959 | BP | GO:0010959 | regulation of metal ion transport | 26/695 | 398/18870 | 0.003577448 | 0.053080959 | 0.045225491 | STC2/TREM2/WNK4/EGF/NOS1/MCHR1/CCL5/CHP2/CASR/NKAIN1/CXCL9/CXCL10/CXCL11/IFNG/HAMP/HECW1/F2/P2RX2/EPO/FXYD4/TF/CNTN1/SEMG1/DPP6/NKX2-5/SLN | 26 |
| GO:0045211 | CC | GO:0045211 | postsynaptic membrane | 20/743 | 275/19886 | 0.003625528 | 0.042114813 | 0.036844304 | GABRD/NETO2/GRIK3/DDN/SLC6A3/PTPRO/CHRNA1/ATP2B2/KCTD8/CHRNA6/ERBB4/GRM1/CNTN1/GRIA4/GRIK5/CHRND/GABRA2/SORCS3/CHRNA4/CACNG2 | 20 |
| GO:0042734 | CC | GO:0042734 | presynaptic membrane | 14/743 | 165/19886 | 0.00364995 | 0.042114813 | 0.036844304 | GRIK3/SLC6A3/SLC5A7/ATP2B2/KCTD8/NTNG1/CHRNA6/ERBB4/SYT7/CNTN1/SCN2A/GRIK5/SCN10A/ADCY8 | 14 |
| GO:0043388 | BP | GO:0043388 | positive regulation of DNA binding | 6/695 | 41/18870 | 0.003658006 | 0.053651336 | 0.045711457 | EGF/PYHIN1/IFNG/MMP9/GATA3/POU4F1 | 6 |
| GO:0045066 | BP | GO:0045066 | regulatory T cell differentiation | 6/695 | 41/18870 | 0.003658006 | 0.053651336 | 0.045711457 | USP44/LILRB4/HLA-G/IFNG/LAG3/PLA2G2D | 6 |
| GO:0050892 | BP | GO:0050892 | intestinal absorption | 6/695 | 41/18870 | 0.003658006 | 0.053651336 | 0.045711457 | SCARB1/HAMP/CEL/FABP1/MOGAT2/APOA4 | 6 |
| GO:0002923 | BP | GO:0002923 | regulation of humoral immune response mediated by circulating immunoglobulin | 4/695 | 18/18870 | 0.003695092 | 0.053651336 | 0.045711457 | TREM2/SUSD4/CR2/FOXJ1 | 4 |
| GO:0006957 | BP | GO:0006957 | complement activation, alternative pathway | 4/695 | 18/18870 | 0.003695092 | 0.053651336 | 0.045711457 | C3/SUSD4/C7/CR2 | 4 |
| GO:0051917 | BP | GO:0051917 | regulation of fibrinolysis | 4/695 | 18/18870 | 0.003695092 | 0.053651336 | 0.045711457 | HRG/F2/F11/PLG | 4 |
| GO:0015908 | BP | GO:0015908 | fatty acid transport | 11/695 | 116/18870 | 0.003698068 | 0.053651336 | 0.045711457 | FABP6/PLA2R1/FABP7/RBP2/AVPR1B/SLC22A8/CYP4F2/FABP1/PLA2G2D/NMUR2/SLC22A7 | 11 |
| GO:0032835 | BP | GO:0032835 | glomerulus development | 8/695 | 69/18870 | 0.003752919 | 0.053933465 | 0.045951834 | ANGPT2/TCF21/PTPRO/NPHS1/NPHS2/FOXJ1/LHX1/BMP7 | 8 |
| GO:0045670 | BP | GO:0045670 | regulation of osteoclast differentiation | 8/695 | 69/18870 | 0.003752919 | 0.053933465 | 0.045951834 | TREM2/TNFAIP6/LILRB4/SFRP1/TMEM178A/IFNG/POU4F1/FSHB | 8 |
| GO:0050918 | BP | GO:0050918 | positive chemotaxis | 8/695 | 69/18870 | 0.003752919 | 0.053933465 | 0.045951834 | VEGFA/PGF/ANGPT2/CCL5/CASR/CXCL10/SCG2/FGF10 | 8 |
| GO:0000041 | BP | GO:0000041 | transition metal ion transport | 10/695 | 100/18870 | 0.003781661 | 0.054006848 | 0.046014357 | HRG/CP/IFNG/TFR2/HAMP/TF/SLC30A2/MT3/SLC30A8/SLC30A10 | 10 |
| GO:0050764 | BP | GO:0050764 | regulation of phagocytosis | 10/695 | 100/18870 | 0.003781661 | 0.054006848 | 0.046014357 | SCARB1/TREM2/CD300LF/IL2RB/FCGR1A/C3/SIRPG/IFNG/SYT7/MBL2 | 10 |
| GO:0099505 | BP | GO:0099505 | regulation of presynaptic membrane potential | 5/695 | 29/18870 | 0.003805649 | 0.05416029 | 0.046145091 | GRIK3/GRIA4/GRIK5/GABRA2/SCN10A | 5 |
| GO:0071695 | BP | GO:0071695 | anatomical structure maturation | 19/695 | 261/18870 | 0.003816108 | 0.05416029 | 0.046145091 | VEGFA/PTPRN/C3/LGI4/PTH1R/SEMG2/HES5/GATA3/FGG/PAEP/C1QL1/EPO/SEMG1/VSX1/FOXJ1/TFCP2L1/HOXB13/CATSPERD/ACTL6B | 19 |
| GO:0005452 | MF | GO:0005452 | solute:inorganic anion antiporter activity | 5/709 | 28/18496 | 0.003852293 | 0.041018779 | 0.034953346 | SLC4A11/SLC22A8/SLC26A4/SLC4A9/SLC4A1 | 5 |
| GO:0002455 | BP | GO:0002455 | humoral immune response mediated by circulating immunoglobulin | 7/695 | 55/18870 | 0.003895099 | 0.055110226 | 0.046954446 | TREM2/C3/SUSD4/C7/CR2/FOXJ1/MBL2 | 7 |
| GO:0098839 | CC | GO:0098839 | postsynaptic density membrane | 10/743 | 99/19886 | 0.003896044 | 0.043830499 | 0.038345279 | NETO2/GRIK3/PTPRO/ATP2B2/ERBB4/GRM1/GRIA4/GRIK5/SORCS3/CACNG2 | 10 |
| GO:1901618 | MF | GO:1901618 | organic hydroxy compound transmembrane transporter activity | 7/709 | 53/18496 | 0.003926511 | 0.041228365 | 0.03513194 | SLC16A3/SLC6A3/ABCA4/AQP2/STRA6/SLC5A11/AQP9 | 7 |
| GO:0023061 | BP | GO:0023061 | signal release | 30/695 | 484/18870 | 0.003967766 | 0.0559651 | 0.047682807 | DOC2A/SOX11/INHBB/TRPV6/WNK4/SFRP1/PTPRN/ABAT/CCL5/CRHBP/CASR/IFNG/TFR2/TFAP2B/GATA3/FAM3B/F2/FGG/CHRNA6/SYT7/SLC18A3/SSTR5/GRIK5/IL11/SLC30A8/ADCY8/FGA/CPLX2/CHRNA4/FGB | 30 |
| GO:1903037 | BP | GO:1903037 | regulation of leukocyte cell-cell adhesion | 25/695 | 382/18870 | 0.00413478 | 0.05799908 | 0.049415777 | CD70/IDO1/IL20RB/PIK3R6/LILRB4/TNFSF9/CCL5/HLA-G/TNFSF14/CRTAM/ICOS/SIRPG/IFNG/LAG3/CD27/KLRK1/RAG2/GATA3/HHLA2/EPO/PLA2G2D/FOXJ1/VTCN1/CCL25/ACTL6B | 25 |
| GO:0006958 | BP | GO:0006958 | complement activation, classical pathway | 6/695 | 42/18870 | 0.004137352 | 0.05799908 | 0.049415777 | TREM2/C3/SUSD4/C7/CR2/MBL2 | 6 |
| GO:0002706 | BP | GO:0002706 | regulation of lymphocyte mediated immunity | 15/695 | 188/18870 | 0.004173289 | 0.058237622 | 0.049619017 | TREM2/IL20RB/PIK3R6/LILRB4/FCGR1A/C3/HLA-G/CRTAM/LAG3/KLRK1/SUSD4/GATA3/CLEC12B/CR2/FOXJ1 | 15 |
| GO:0022409 | BP | GO:0022409 | positive regulation of cell-cell adhesion | 22/695 | 322/18870 | 0.004184751 | 0.058237622 | 0.049619017 | CD70/PIK3R6/LILRB4/TNFSF9/CCL5/HLA-G/TNFSF14/ICOS/SIRPG/IFNG/CD27/KLRK1/GATA3/CXCL13/FGG/HHLA2/EPO/VTCN1/FGA/BMP7/FGB/ACTL6B | 22 |
| GO:0006937 | BP | GO:0006937 | regulation of muscle contraction | 14/695 | 170/18870 | 0.004192599 | 0.058237622 | 0.049619017 | NOS1/ABAT/TNNT2/TNNI1/TNNC1/MYBPH/TNNT1/MYH7/NMUR2/TACR3/SCN10A/NKX2-5/GATA4/CALCA | 14 |
| GO:1904724 | CC | GO:1904724 | tertiary granule lumen | 7/743 | 55/19886 | 0.00421601 | 0.046273285 | 0.040482359 | TNFAIP6/CHIT1/MMP9/HP/OLFM4/FOLR3/ORM1 | 7 |
| GO:0006816 | BP | GO:0006816 | calcium ion transport | 28/695 | 445/18870 | 0.004308237 | 0.059551688 | 0.050738614 | NOL3/STC2/TRPV6/EGF/NOS1/TRPA1/MCHR1/FASLG/CCL5/CASR/CXCL9/CXCL10/CYP27B1/TRPV5/CXCL11/ATP2B2/F2/PKD2L1/P2RX2/EPO/SCN7A/SEMG1/SCN2A/NMUR2/SCN10A/CHRNA4/SLN/CACNG2 | 28 |
| GO:0030857 | BP | GO:0030857 | negative regulation of epithelial cell differentiation | 7/695 | 56/18870 | 0.004313262 | 0.059551688 | 0.050738614 | VEGFA/IFNG/MMP9/HES5/GRHL2/FGF10/REG3A | 7 |
| GO:0033273 | BP | GO:0033273 | response to vitamin | 9/695 | 86/18870 | 0.004351083 | 0.059752768 | 0.050909937 | STC2/SFRP1/CASR/CXCL10/CYP27B1/EPO/SLC34A1/BMP7/GATA4 | 9 |
| GO:0009913 | BP | GO:0009913 | epidermal cell differentiation | 18/695 | 245/18870 | 0.004353976 | 0.059752768 | 0.050909937 | ALOX15B/CYP27B1/HES5/KRT81/KRT36/SULT2B1/KRT72/GRHL2/OVOL2/LHFPL5/KRT6A/ESRP1/KLK5/KRT78/REG3A/KRT6B/SPRR3/LCE2D | 18 |
| GO:0010039 | BP | GO:0010039 | response to iron ion | 5/695 | 30/18870 | 0.004430666 | 0.060623184 | 0.051651539 | SLC6A3/ABAT/TFR2/HAMP/TF | 5 |
| GO:0098686 | CC | GO:0098686 | hippocampal mossy fiber to CA3 synapse | 6/743 | 42/19886 | 0.004439397 | 0.047564973 | 0.041612397 | CALB1/SYT7/GRIK5/ADCY8/CACNG2/CALCA | 6 |
| GO:0042056 | MF | GO:0042056 | chemoattractant activity | 6/709 | 41/18496 | 0.004445819 | 0.046041636 | 0.039233474 | VEGFA/PGF/CCL5/CXCL10/SCG2/FGF10 | 6 |
| GO:0002548 | BP | GO:0002548 | monocyte chemotaxis | 8/695 | 71/18870 | 0.004479309 | 0.060923931 | 0.051907779 | PLA2G7/PTPRO/CCL18/CCL5/CXCL10/CCL20/CCL25/CALCA | 8 |
| GO:0030239 | BP | GO:0030239 | myofibril assembly | 8/695 | 71/18870 | 0.004479309 | 0.060923931 | 0.051907779 | MYLK3/PROX1/MYOZ2/TNNT2/TNNT1/MYH7/NKX2-5/ACTL8 | 8 |
| GO:0008037 | BP | GO:0008037 | cell recognition | 13/695 | 154/18870 | 0.004618974 | 0.062637122 | 0.053367434 | SCARB1/TREM2/FCGR1A/ADAM18/ATP8B3/CRTAM/ZP1/PAEP/CRP/ZAN/FOLR3/LBP/MBL2 | 13 |
| GO:0045663 | BP | GO:0045663 | positive regulation of myoblast differentiation | 6/695 | 43/18870 | 0.004661563 | 0.063027645 | 0.053700163 | IGFBP3/RANBP3L/TNFSF14/CXCL9/MYOD1/ACTL6B | 6 |
| GO:0010466 | BP | GO:0010466 | negative regulation of peptidase activity | 12/695 | 137/18870 | 0.004754541 | 0.063655444 | 0.054235054 | NOL3/VEGFA/HRG/KNG1/CST7/SERPINA5/DPEP1/MMP9/FABP1/SERPINA12/SERPINA9/MAGEA3 | 12 |
| GO:1901616 | BP | GO:1901616 | organic hydroxy compound catabolic process | 7/695 | 57/18870 | 0.004764478 | 0.063655444 | 0.054235054 | SCARB1/SLC6A3/CYP27B1/CYP4F2/ALDH3B2/HAO1/SULT2A1 | 7 |
| GO:0005581 | CC | GO:0005581 | collagen trimer | 9/743 | 86/19886 | 0.004779939 | 0.050022613 | 0.043762473 | COL23A1/COL5A3/LOX/COL4A6/COL26A1/CTHRC1/C1QL1/C1QL4/MBL2 | 9 |
| GO:0001653 | MF | GO:0001653 | peptide receptor activity | 12/709 | 132/18496 | 0.004828687 | 0.04933091 | 0.042036365 | KISS1R/MCHR1/HCRTR2/AVPR1B/AVPR2/PTH1R/SSTR5/NMUR2/TACR3/UTS2R/SORCS3/NPY2R | 12 |
| GO:0051961 | BP | GO:0051961 | negative regulation of nervous system development | 13/695 | 155/18870 | 0.004877236 | 0.063655444 | 0.054235054 | TREM2/SEMA5B/SOX11/PROX1/DLX1/HES5/F2/DLX2/NKX6-2/NR2E1/MT3/LHX2/BMP7 | 13 |
| GO:0030858 | BP | GO:0030858 | positive regulation of epithelial cell differentiation | 8/695 | 72/18870 | 0.004880723 | 0.063655444 | 0.054235054 | ALOX15B/CYP27B1/TP73/SULT2B1/FOXJ1/OVOL2/LHX1/BMP7 | 8 |
| GO:0042130 | BP | GO:0042130 | negative regulation of T cell proliferation | 8/695 | 72/18870 | 0.004880723 | 0.063655444 | 0.054235054 | IDO1/IL20RB/LILRB4/HLA-G/CRTAM/PLA2G2D/FOXJ1/VTCN1 | 8 |
| GO:0050766 | BP | GO:0050766 | positive regulation of phagocytosis | 8/695 | 72/18870 | 0.004880723 | 0.063655444 | 0.054235054 | TREM2/CD300LF/IL2RB/FCGR1A/C3/SIRPG/IFNG/MBL2 | 8 |
| GO:0002883 | BP | GO:0002883 | regulation of hypersensitivity | 3/695 | 10/18870 | 0.00491693 | 0.063655444 | 0.054235054 | IL20RB/FCGR1A/C3 | 3 |
| GO:0010496 | BP | GO:0010496 | intercellular transport | 3/695 | 10/18870 | 0.00491693 | 0.063655444 | 0.054235054 | CLDN16/GJA3/GJA8 | 3 |
| GO:0030002 | BP | GO:0030002 | intracellular monoatomic anion homeostasis | 3/695 | 10/18870 | 0.00491693 | 0.063655444 | 0.054235054 | WNK4/FASLG/UMOD | 3 |
| GO:0030643 | BP | GO:0030643 | intracellular phosphate ion homeostasis | 3/695 | 10/18870 | 0.00491693 | 0.063655444 | 0.054235054 | UMOD/SLC34A3/SLC34A1 | 3 |
| GO:0030644 | BP | GO:0030644 | intracellular chloride ion homeostasis | 3/695 | 10/18870 | 0.00491693 | 0.063655444 | 0.054235054 | WNK4/FASLG/UMOD | 3 |
| GO:0060525 | BP | GO:0060525 | prostate glandular acinus development | 3/695 | 10/18870 | 0.00491693 | 0.063655444 | 0.054235054 | SFRP1/HOXD13/HOXB13 | 3 |
| GO:0072205 | BP | GO:0072205 | metanephric collecting duct development | 3/695 | 10/18870 | 0.00491693 | 0.063655444 | 0.054235054 | CALB1/AQP2/WNT7B | 3 |
| GO:0099566 | BP | GO:0099566 | regulation of postsynaptic cytosolic calcium ion concentration | 3/695 | 10/18870 | 0.00491693 | 0.063655444 | 0.054235054 | CALB1/ATP2B2/GRM1 | 3 |
| GO:2000425 | BP | GO:2000425 | regulation of apoptotic cell clearance | 3/695 | 10/18870 | 0.00491693 | 0.063655444 | 0.054235054 | TREM2/CD300LF/C3 | 3 |
| GO:0061135 | MF | GO:0061135 | endopeptidase regulator activity | 15/709 | 184/18496 | 0.004917705 | 0.049570469 | 0.0422405 | BIRC7/C3/HRG/GBP5/KNG1/CST7/SERPINA5/SPINK13/CST9/AIM2/ITIH1/LCN1/WFDC10B/SERPINA12/SERPINA9 | 15 |
| GO:0007188 | BP | GO:0007188 | adenylate cyclase-modulating G protein-coupled receptor signaling pathway | 18/695 | 248/18870 | 0.004936832 | 0.063732554 | 0.054300753 | GRIK3/PTHLH/RGS1/MCHR1/CASR/CXCL9/CXCL10/CXCL11/AVPR2/PTH1R/PTGER1/MRAP2/GRM1/GPR12/MTNR1A/NPY2R/ADCY8/CALCA | 18 |
| GO:0030098 | BP | GO:0030098 | lymphocyte differentiation | 27/695 | 429/18870 | 0.004986745 | 0.064195564 | 0.054695241 | PIK3R6/USP44/LILRB4/TNFSF9/SFRP1/TNFRSF9/EOMES/AICDA/HLA-G/CD8A/CD2/ZNF683/BATF/CRTAM/IFNG/LAG3/CD27/RAG2/GATA3/PLA2G2D/CR2/FOXJ1/IL11/PGLYRP2/IFNA14/ACTL6B/NKX2-3 | 27 |
| GO:0030217 | BP | GO:0030217 | T cell differentiation | 21/695 | 307/18870 | 0.00500156 | 0.064205414 | 0.054703634 | PIK3R6/USP44/LILRB4/TNFSF9/TNFRSF9/EOMES/HLA-G/CD8A/CD2/ZNF683/BATF/CRTAM/IFNG/LAG3/CD27/RAG2/GATA3/PLA2G2D/FOXJ1/ACTL6B/NKX2-3 | 21 |
| GO:0050672 | BP | GO:0050672 | negative regulation of lymphocyte proliferation | 9/695 | 88/18870 | 0.005067811 | 0.064873658 | 0.055272984 | SOX11/IDO1/IL20RB/LILRB4/HLA-G/CRTAM/PLA2G2D/FOXJ1/VTCN1 | 9 |
| GO:0007271 | BP | GO:0007271 | synaptic transmission, cholinergic | 5/695 | 31/18870 | 0.005125427 | 0.065427941 | 0.055745239 | SLC5A7/CHRNA1/CHRNA6/SLC18A3/CHRNA4 | 5 |
| GO:0046661 | BP | GO:0046661 | male sex differentiation | 14/695 | 174/18870 | 0.005147574 | 0.065527618 | 0.055830164 | INHBB/SFRP1/TCF21/ADAM18/WNT9B/TEX11/GATA3/HSD17B3/HOXD13/FGF10/DMRT3/GATA4/FSHB/DMRT1 | 14 |
| GO:0016918 | MF | GO:0016918 | retinal binding | 4/709 | 19/18496 | 0.005238526 | 0.051432803 | 0.043827451 | RBP2/ABCA4/STRA6/OPN4 | 4 |
| GO:0042834 | MF | GO:0042834 | peptidoglycan binding | 4/709 | 19/18496 | 0.005238526 | 0.051432803 | 0.043827451 | TREM2/PGLYRP2/REG1B/REG3A | 4 |
| GO:0002707 | BP | GO:0002707 | negative regulation of lymphocyte mediated immunity | 7/695 | 58/18870 | 0.005250336 | 0.066650102 | 0.056786532 | IL20RB/LILRB4/HLA-G/SUSD4/CLEC12B/CR2/FOXJ1 | 7 |
| GO:0045661 | BP | GO:0045661 | regulation of myoblast differentiation | 8/695 | 73/18870 | 0.005309116 | 0.067023921 | 0.057105029 | IGFBP3/RANBP3L/TNFSF14/CXCL9/CXCL10/ANKRD2/MYOD1/ACTL6B | 8 |
| GO:0055002 | BP | GO:0055002 | striated muscle cell development | 8/695 | 73/18870 | 0.005309116 | 0.067023921 | 0.057105029 | MYLK3/PROX1/MYOZ2/TNNT2/TNNT1/MYH7/NKX2-5/ACTL8 | 8 |
| GO:0004866 | MF | GO:0004866 | endopeptidase inhibitor activity | 14/709 | 168/18496 | 0.005338169 | 0.051739174 | 0.044088519 | BIRC7/C3/HRG/GBP5/KNG1/CST7/SERPINA5/SPINK13/CST9/ITIH1/LCN1/WFDC10B/SERPINA12/SERPINA9 | 14 |
| GO:0042472 | BP | GO:0042472 | inner ear morphogenesis | 10/695 | 105/18870 | 0.005354592 | 0.067411805 | 0.05743551 | DLX5/MYO3A/PROX1/GATA3/CTHRC1/MYO3B/GBX2/FGF10/LHFPL5/HMX2 | 10 |
| GO:0046849 | BP | GO:0046849 | bone remodeling | 9/695 | 89/18870 | 0.005458862 | 0.06835014 | 0.05823498 | SFRP1/PTH1R/HAMP/CTHRC1/DCSTAMP/SYT7/TF/FSHB/CALCA | 9 |
| GO:0042359 | BP | GO:0042359 | vitamin D metabolic process | 4/695 | 20/18870 | 0.005518862 | 0.06835014 | 0.05823498 | CYP27B1/IFNG/UGT1A3/UGT1A4 | 4 |
| GO:0042693 | BP | GO:0042693 | muscle cell fate commitment | 4/695 | 20/18870 | 0.005518862 | 0.06835014 | 0.05823498 | TBX5/FGF10/NKX2-5/MYOD1 | 4 |
| GO:0055062 | BP | GO:0055062 | phosphate ion homeostasis | 4/695 | 20/18870 | 0.005518862 | 0.06835014 | 0.05823498 | ENPP3/UMOD/SLC34A3/SLC34A1 | 4 |
| GO:0097154 | BP | GO:0097154 | GABAergic neuron differentiation | 4/695 | 20/18870 | 0.005518862 | 0.06835014 | 0.05823498 | DLX1/HELT/DLX2/ARX | 4 |
| GO:1901317 | BP | GO:1901317 | regulation of flagellated sperm motility | 4/695 | 20/18870 | 0.005518862 | 0.06835014 | 0.05823498 | TPPP2/SEMG2/SEMG1/TACR3 | 4 |
| GO:0042098 | BP | GO:0042098 | T cell proliferation | 16/695 | 213/18870 | 0.005639669 | 0.06965753 | 0.05934889 | CD70/TNFRSF4/IDO1/IL20RB/LILRB4/TNFSF9/CCL5/TNFRSF9/HLA-G/TNFSF14/CRTAM/HHLA2/EPO/PLA2G2D/FOXJ1/VTCN1 | 16 |
| GO:0016782 | MF | GO:0016782 | transferase activity, transferring sulphur-containing groups | 8/709 | 71/18496 | 0.005675197 | 0.05430948 | 0.046278755 | HS6ST2/HS3ST2/NDST3/GAL3ST3/SULT2B1/HS3ST6/HS3ST5/SULT2A1 | 8 |
| GO:0030100 | BP | GO:0030100 | regulation of endocytosis | 20/695 | 291/18870 | 0.005762906 | 0.070987823 | 0.060482312 | SCARB1/TREM2/VEGFA/APOC1/CD300LF/IL2RB/EGF/FCGR1A/C3/SH3GL3/SIRPG/IFNG/GPC3/TFR2/SYT7/ABCA13/TF/PROM2/APELA/MBL2 | 20 |
| GO:0055001 | BP | GO:0055001 | muscle cell development | 15/695 | 195/18870 | 0.005835321 | 0.071533797 | 0.060947487 | VEGFA/MYLK3/PROX1/LOX/MYOZ2/TNNT2/TNNT1/P2RX2/TBX5/MYH7/SGCZ/NKX2-5/MYOD1/GATA4/ACTL8 | 15 |
| GO:0031641 | BP | GO:0031641 | regulation of myelination | 6/695 | 45/18870 | 0.005854188 | 0.071533797 | 0.060947487 | ITGAX/LGI4/CST7/ZNF488/HES5/NKX6-2 | 6 |
| GO:0045214 | BP | GO:0045214 | sarcomere organization | 6/695 | 45/18870 | 0.005854188 | 0.071533797 | 0.060947487 | MYLK3/PROX1/MYOZ2/TNNT2/TNNT1/MYH7 | 6 |
| GO:0007589 | BP | GO:0007589 | body fluid secretion | 9/695 | 90/18870 | 0.005872842 | 0.071570364 | 0.060978643 | VEGFA/HK2/SLC6A3/WNK4/CEL/ERBB4/SCNN1B/FGF10/SLC4A9 | 9 |
| GO:0030216 | BP | GO:0030216 | keratinocyte differentiation | 14/695 | 177/18870 | 0.005973027 | 0.072597695 | 0.061853938 | ALOX15B/CYP27B1/KRT81/KRT36/KRT72/GRHL2/OVOL2/KRT6A/KLK5/KRT78/REG3A/KRT6B/SPRR3/LCE2D | 14 |
| GO:0070372 | BP | GO:0070372 | regulation of ERK1 and ERK2 cascade | 21/695 | 313/18870 | 0.006208966 | 0.075265184 | 0.064126665 | TREM2/MTURN/FGF1/CCL18/CCL5/CASR/LMO3/FGG/CCL20/EPO/ERBB4/MT3/C1QL4/FGF10/CCL25/FGA/APELA/GATA4/FGB/SLC30A10/OR2AT4 | 21 |
| GO:0043195 | CC | GO:0043195 | terminal bouton | 6/743 | 45/19886 | 0.006273032 | 0.062770576 | 0.054915077 | GRIK3/CALB1/SCGN/SLC18A3/CPLX2/CALCA | 6 |
| GO:0030669 | CC | GO:0030669 | clathrin-coated endocytic vesicle membrane | 8/743 | 74/19886 | 0.006277058 | 0.062770576 | 0.054915077 | EGF/FCGR1A/AVPR2/EPGN/TYRP1/APOB/SLC18A3/TF | 8 |
| GO:0033555 | BP | GO:0033555 | multicellular organismal response to stress | 9/695 | 91/18870 | 0.006310612 | 0.076142877 | 0.064874468 | IDO1/NOS1/TRPA1/UMOD/P2RX2/AQP9/NR2E1/NMUR2/CALCA | 9 |
| GO:0007517 | BP | GO:0007517 | muscle organ development | 23/695 | 354/18870 | 0.006314694 | 0.076142877 | 0.064874468 | SOX11/TCF21/PROX1/LOX/NPHS1/EOMES/CXCL10/MYOZ2/CHRNA1/MSC/TNNT2/TNNI1/STRA6/TNNC1/P2RX2/MYH7/CHRND/ANKRD2/ANKRD33/PITX1/POU4F1/NKX2-5/MYOD1 | 23 |
| GO:0001658 | BP | GO:0001658 | branching involved in ureteric bud morphogenesis | 7/695 | 60/18870 | 0.006332383 | 0.076155237 | 0.064884998 | VEGFA/FGF1/TCF21/WNT9B/GPC3/TACSTD2/LHX1 | 7 |
| GO:0050673 | BP | GO:0050673 | epithelial cell proliferation | 29/695 | 480/18870 | 0.006479711 | 0.076611035 | 0.065273343 | SCARB1/VEGFA/SOX11/IGFBP3/FGF1/FABP7/PGF/EGF/DLX5/SFRP1/PTPRN/PROX1/EPGN/GPC3/SCG2/HES5/GATA3/CLEC12B/TACSTD2/FGF10/OVOL2/HMX2/NKX2-5/ARX/APELA/FSHB/REG3A/FGFBP1/NKX2-3 | 29 |
| GO:0001838 | BP | GO:0001838 | embryonic epithelial tube formation | 11/695 | 125/18870 | 0.006493071 | 0.076611035 | 0.065273343 | IRX2/SFRP1/WNT9B/IRX1/HES5/GATA3/CTHRC1/LHX2/GRHL2/OVOL2/BMP7 | 11 |
| GO:0003009 | BP | GO:0003009 | skeletal muscle contraction | 6/695 | 46/18870 | 0.006527403 | 0.076611035 | 0.065273343 | CHRNA1/TNNI1/TNNC1/TNNT1/MYH7/CHRND | 6 |
| GO:1990573 | BP | GO:1990573 | potassium ion import across plasma membrane | 6/695 | 46/18870 | 0.006527403 | 0.076611035 | 0.065273343 | KCNJ10/SLC12A1/ATP12A/KCNJ1/KCNK9/SLC12A3 | 6 |
| GO:0002775 | BP | GO:0002775 | antimicrobial peptide production | 3/695 | 11/18870 | 0.006577249 | 0.076611035 | 0.065273343 | KLK7/LGALS4/KLK5 | 3 |
| GO:0002924 | BP | GO:0002924 | negative regulation of humoral immune response mediated by circulating immunoglobulin | 3/695 | 11/18870 | 0.006577249 | 0.076611035 | 0.065273343 | SUSD4/CR2/FOXJ1 | 3 |
| GO:0015747 | BP | GO:0015747 | urate transport | 3/695 | 11/18870 | 0.006577249 | 0.076611035 | 0.065273343 | UMOD/SLC17A4/SLC17A2 | 3 |
| GO:0021892 | BP | GO:0021892 | cerebral cortex GABAergic interneuron differentiation | 3/695 | 11/18870 | 0.006577249 | 0.076611035 | 0.065273343 | DLX1/DLX2/ARX | 3 |
| GO:0043320 | BP | GO:0043320 | natural killer cell degranulation | 3/695 | 11/18870 | 0.006577249 | 0.076611035 | 0.065273343 | FCGR3A/NKG7/KLRF2 | 3 |
| GO:0060900 | BP | GO:0060900 | embryonic camera-type eye formation | 3/695 | 11/18870 | 0.006577249 | 0.076611035 | 0.065273343 | SOX11/PROX1/STRA6 | 3 |
| GO:0071918 | BP | GO:0071918 | urea transmembrane transport | 3/695 | 11/18870 | 0.006577249 | 0.076611035 | 0.065273343 | SLC14A2/UMOD/AQP9 | 3 |
| GO:0035809 | BP | GO:0035809 | regulation of urine volume | 4/695 | 21/18870 | 0.006621742 | 0.076611035 | 0.065273343 | TRPV5/AVPR2/UMOD/SLC4A1 | 4 |
| GO:0052695 | BP | GO:0052695 | cellular glucuronidation | 4/695 | 21/18870 | 0.006621742 | 0.076611035 | 0.065273343 | UGT1A3/UGT1A10/UGT2A1/UGT1A4 | 4 |
| GO:0097501 | BP | GO:0097501 | stress response to metal ion | 4/695 | 21/18870 | 0.006621742 | 0.076611035 | 0.065273343 | MT1G/MT1H/MT3/SLC30A10 | 4 |
| GO:1901739 | BP | GO:1901739 | regulation of myoblast fusion | 4/695 | 21/18870 | 0.006621742 | 0.076611035 | 0.065273343 | TNFSF14/CXCL9/CXCL10/MYOD1 | 4 |
| GO:0032102 | BP | GO:0032102 | negative regulation of response to external stimulus | 29/695 | 481/18870 | 0.006662561 | 0.076812307 | 0.065444829 | TREM2/SEMA5B/TNFAIP6/ENPP3/SLC6A3/IL20RB/ANGPT2/CLDN19/HRG/PTPRO/KNG1/CST7/HLA-G/AURKB/KLRK1/SUSD4/HAMP/GATA3/F2/F11/CXCL13/CLEC12B/FGG/TSPAN8/SAA1/FGA/PLG/FGB/REG3A | 29 |
| GO:0015012 | BP | GO:0015012 | heparan sulfate proteoglycan biosynthetic process | 5/695 | 33/18870 | 0.006739986 | 0.076812307 | 0.065444829 | HS6ST2/HS3ST2/NDST3/HS3ST6/HS3ST5 | 5 |
| GO:0034368 | BP | GO:0034368 | protein-lipid complex remodeling | 5/695 | 33/18870 | 0.006739986 | 0.076812307 | 0.065444829 | SCARB1/APOC1/PLA2G7/APOB/APOA4 | 5 |
| GO:0034369 | BP | GO:0034369 | plasma lipoprotein particle remodeling | 5/695 | 33/18870 | 0.006739986 | 0.076812307 | 0.065444829 | SCARB1/APOC1/PLA2G7/APOB/APOA4 | 5 |
| GO:0048566 | BP | GO:0048566 | embryonic digestive tract development | 5/695 | 33/18870 | 0.006739986 | 0.076812307 | 0.065444829 | SOX11/TCF21/STRA6/FGF10/OVOL2 | 5 |
| GO:0050891 | BP | GO:0050891 | multicellular organismal-level water homeostasis | 5/695 | 33/18870 | 0.006739986 | 0.076812307 | 0.065444829 | AQP2/UMOD/CYP4F2/SCNN1B/SCNN1G | 5 |
| GO:0031526 | CC | GO:0031526 | brush border membrane | 7/743 | 60/19886 | 0.0068383 | 0.066896417 | 0.058524585 | SLC28A2/SLC26A4/SLC17A4/ITLN1/SLC9A3/SLC34A3/SLC34A1 | 7 |
| GO:0070851 | MF | GO:0070851 | growth factor receptor binding | 12/709 | 138/18496 | 0.006854437 | 0.064774433 | 0.055196259 | VEGFA/ESM1/FGF1/CD300LF/PGF/EGF/EPGN/GATA3/ERBB4/IL11/FGF10/FAM83B | 12 |
| GO:0019755 | BP | GO:0019755 | one-carbon compound transport | 7/695 | 61/18870 | 0.00693179 | 0.078411589 | 0.066807433 | SLC4A11/SLC14A2/UMOD/SLC26A4/AQP9/SLC4A9/SLC4A1 | 7 |
| GO:0034113 | BP | GO:0034113 | heterotypic cell-cell adhesion | 7/695 | 61/18870 | 0.00693179 | 0.078411589 | 0.066807433 | ITGAX/ITGAD/CD2/FGG/FGA/BMP7/FGB | 7 |
| GO:0055008 | BP | GO:0055008 | cardiac muscle tissue morphogenesis | 7/695 | 61/18870 | 0.00693179 | 0.078411589 | 0.066807433 | PROX1/TNNT2/TNNI1/TNNC1/MYH7/POU4F1/NKX2-5 | 7 |
| GO:0002520 | BP | GO:0002520 | immune system development | 15/695 | 199/18870 | 0.007002129 | 0.079011683 | 0.067318718 | IDO1/LILRB4/TCF21/AICDA/HLA-G/BATF/ICOS/RAG2/PDCD1/GATA3/FOXJ1/FGF10/BARX1/NKX2-5/NKX2-3 | 15 |
| GO:0031528 | CC | GO:0031528 | microvillus membrane | 5/743 | 33/19886 | 0.007155818 | 0.068513151 | 0.059938991 | CA9/SLC7A8/DPEP1/PROM2/CEACAM20 | 5 |
| GO:0005267 | MF | GO:0005267 | potassium channel activity | 11/709 | 122/18496 | 0.007240435 | 0.066931499 | 0.05703436 | GRIK3/KCNJ10/KCNJ1/KCNK9/KCNN1/KCNK10/PKD2L1/FXYD4/KCNK17/GRIK5/KCNU1 | 11 |
| GO:0048806 | BP | GO:0048806 | genitalia development | 6/695 | 47/18870 | 0.007255071 | 0.081664224 | 0.069578709 | WNT9B/STRA6/HSD17B3/HOXD13/FGF10/LHX1 | 6 |
| GO:0019864 | MF | GO:0019864 | IgG binding | 3/709 | 11/18496 | 0.007348299 | 0.066931499 | 0.05703436 | FCGR3A/FCGR1A/UMOD | 3 |
| GO:0042166 | MF | GO:0042166 | acetylcholine binding | 3/709 | 11/18496 | 0.007348299 | 0.066931499 | 0.05703436 | CHRNA1/CHRND/CHRNA4 | 3 |
| GO:0014013 | BP | GO:0014013 | regulation of gliogenesis | 10/695 | 110/18870 | 0.007398319 | 0.083072033 | 0.070778175 | TREM2/SOX11/DLX1/TP73/ZNF488/HES5/F2/DLX2/NKX6-2/NR2E1 | 10 |
| GO:0045334 | CC | GO:0045334 | clathrin-coated endocytic vesicle | 9/743 | 92/19886 | 0.007419534 | 0.069558127 | 0.060853192 | EGF/CPNE6/FCGR1A/AVPR2/EPGN/TYRP1/APOB/SLC18A3/TF | 9 |
| GO:0050921 | BP | GO:0050921 | positive regulation of chemotaxis | 12/695 | 145/18870 | 0.007422081 | 0.083134587 | 0.070831472 | TREM2/VEGFA/PGF/PLA2G7/CCL5/CASR/TNFSF14/CXCL10/SCG2/CXCL13/FGF10/LBP | 12 |
| GO:0002696 | BP | GO:0002696 | positive regulation of leukocyte activation | 24/695 | 380/18870 | 0.007508963 | 0.083902102 | 0.071485402 | TREM2/CD70/TNFRSF4/FCGR3A/PIK3R6/LILRB4/TNFSF9/CCL5/HLA-G/CD2/TNFSF14/ICOS/SIRPG/IFNG/CD27/KLRK1/HAMP/GATA3/HHLA2/EPO/VTCN1/FGF10/LBP/ACTL6B | 24 |
| GO:0042129 | BP | GO:0042129 | regulation of T cell proliferation | 14/695 | 182/18870 | 0.007581752 | 0.084508801 | 0.072002316 | CD70/IDO1/IL20RB/LILRB4/TNFSF9/CCL5/TNFRSF9/HLA-G/CRTAM/HHLA2/EPO/PLA2G2D/FOXJ1/VTCN1 | 14 |
| GO:0046475 | BP | GO:0046475 | glycerophospholipid catabolic process | 5/695 | 34/18870 | 0.007667506 | 0.084843833 | 0.072287767 | SCARB1/APOC1/ENPP6/PLA2G7/PLA2G4D | 5 |
| GO:0048265 | BP | GO:0048265 | response to pain | 5/695 | 34/18870 | 0.007667506 | 0.084843833 | 0.072287767 | TRPA1/P2RX2/AQP9/NMUR2/CALCA | 5 |
| GO:0048665 | BP | GO:0048665 | neuron fate specification | 5/695 | 34/18870 | 0.007667506 | 0.084843833 | 0.072287767 | ISL2/POU4F1/DMRT3/ESRP1/SOX1 | 5 |
| GO:0048704 | BP | GO:0048704 | embryonic skeletal system morphogenesis | 9/695 | 94/18870 | 0.007775406 | 0.085741265 | 0.073052387 | SOX11/WNT9B/TBX15/DLX2/HOXB9/ALX1/GRHL2/LHX1/BMP7 | 9 |
| GO:0042060 | BP | GO:0042060 | wound healing | 26/695 | 423/18870 | 0.007786132 | 0.085741265 | 0.073052387 | SCARB1/VEGFA/FGF1/VWF/CLDN19/HRG/KNG1/TREML1/F2/PROZ/F11/FGG/CYP4F2/SYT7/TSPAN8/EPB41L4B/SAA1/FGF10/KRT6A/FGA/PLG/SLC4A1/GATA4/FGB/REG3A/SPRR3 | 26 |
| GO:0006883 | BP | GO:0006883 | intracellular sodium ion homeostasis | 4/695 | 22/18870 | 0.007861264 | 0.085848194 | 0.073143492 | ATP12A/UMOD/SCNN1B/SCNN1G | 4 |
| GO:0072234 | BP | GO:0072234 | metanephric nephron tubule development | 4/695 | 22/18870 | 0.007861264 | 0.085848194 | 0.073143492 | CALB1/UMOD/HES5/WNT7B | 4 |
| GO:0072311 | BP | GO:0072311 | glomerular epithelial cell differentiation | 4/695 | 22/18870 | 0.007861264 | 0.085848194 | 0.073143492 | PTPRO/NPHS1/NPHS2/FOXJ1 | 4 |
| GO:1905954 | BP | GO:1905954 | positive regulation of lipid localization | 10/695 | 111/18870 | 0.007870983 | 0.085848194 | 0.073143492 | HILPDA/SCARB1/TREM2/PLA2R1/C3/FASLG/AVPR1B/APOB/CYP4F2/ABCA13 | 10 |
| GO:1905952 | BP | GO:1905952 | regulation of lipid localization | 14/695 | 183/18870 | 0.007941353 | 0.086409489 | 0.07362172 | HILPDA/SCARB1/TREM2/APOC1/PLA2R1/EGF/C3/FASLG/AVPR1B/APOB/CYP4F2/ABCA13/CRP/APOA4 | 14 |
| GO:0042445 | BP | GO:0042445 | hormone metabolic process | 17/695 | 241/18870 | 0.008141662 | 0.088378609 | 0.07529943 | STC2/PLA2G7/CYP27B1/IYD/DIO1/CEL/GATA3/ADH1C/HSD17B3/UGT1A3/SCNN1B/MEP1A/KLK6/SLC30A8/RDH8/FSHB/SULT2A1 | 17 |
| GO:0071715 | BP | GO:0071715 | icosanoid transport | 7/695 | 63/18870 | 0.008255435 | 0.089401271 | 0.076170748 | PLA2R1/AVPR1B/SLC22A8/CYP4F2/PLA2G2D/NMUR2/SLC22A7 | 7 |
| GO:0031406 | MF | GO:0031406 | carboxylic acid binding | 15/709 | 195/18496 | 0.008287522 | 0.074587699 | 0.06355844 | EGLN3/FABP6/SIGLEC8/ST8SIA4/APOC1/FABP7/RBP2/NOS1/CASR/ALB/SERPINA5/AGXT/UGT1A3/FABP1/FOLR3 | 15 |
| GO:0015791 | BP | GO:0015791 | polyol transmembrane transport | 3/695 | 12/18870 | 0.008532066 | 0.090678002 | 0.077258535 | AQP2/SLC5A11/AQP9 | 3 |
| GO:0015840 | BP | GO:0015840 | urea transport | 3/695 | 12/18870 | 0.008532066 | 0.090678002 | 0.077258535 | SLC14A2/UMOD/AQP9 | 3 |
| GO:0031630 | BP | GO:0031630 | regulation of synaptic vesicle fusion to presynaptic active zone membrane | 3/695 | 12/18870 | 0.008532066 | 0.090678002 | 0.077258535 | DOC2A/GRIK5/CPLX2 | 3 |
| GO:0044341 | BP | GO:0044341 | sodium-dependent phosphate transport | 3/695 | 12/18870 | 0.008532066 | 0.090678002 | 0.077258535 | SLC17A4/SLC34A3/SLC34A1 | 3 |
| GO:0051549 | BP | GO:0051549 | positive regulation of keratinocyte migration | 3/695 | 12/18870 | 0.008532066 | 0.090678002 | 0.077258535 | MMP9/EPB41L4B/FGF10 | 3 |
| GO:0061303 | BP | GO:0061303 | cornea development in camera-type eye | 3/695 | 12/18870 | 0.008532066 | 0.090678002 | 0.077258535 | SOX11/WNT9B/KERA | 3 |
| GO:0072310 | BP | GO:0072310 | glomerular epithelial cell development | 3/695 | 12/18870 | 0.008532066 | 0.090678002 | 0.077258535 | NPHS1/NPHS2/FOXJ1 | 3 |
| GO:1901632 | BP | GO:1901632 | regulation of synaptic vesicle membrane organization | 3/695 | 12/18870 | 0.008532066 | 0.090678002 | 0.077258535 | DOC2A/GRIK5/CPLX2 | 3 |
| GO:0015078 | MF | GO:0015078 | proton transmembrane transporter activity | 12/709 | 142/18496 | 0.008540713 | 0.075962102 | 0.064729611 | SLC16A3/SLC4A11/ATP12A/SLC15A2/SLC47A2/SLC9A3/SLC9A4/SLC18A3/SLC36A2/SLC30A2/SLC30A8/SLC9A2 | 12 |
| GO:0034367 | BP | GO:0034367 | protein-containing complex remodeling | 5/695 | 35/18870 | 0.008680189 | 0.091416955 | 0.07788813 | SCARB1/APOC1/PLA2G7/APOB/APOA4 | 5 |
| GO:0070633 | BP | GO:0070633 | transepithelial transport | 5/695 | 35/18870 | 0.008680189 | 0.091416955 | 0.07788813 | CLDN19/CLCNKA/SLC9A4/SCNN1B/CLCNKB | 5 |
| GO:2000406 | BP | GO:2000406 | positive regulation of T cell migration | 5/695 | 35/18870 | 0.008680189 | 0.091416955 | 0.07788813 | CCL5/TNFSF14/CXCL10/CXCL13/CCL20 | 5 |
| GO:0060078 | BP | GO:0060078 | regulation of postsynaptic membrane potential | 12/695 | 148/18870 | 0.00868161 | 0.091416955 | 0.07788813 | GABRD/GRIK3/ABAT/CHRNA1/P2RX2/CHRNA6/GRM1/GRIA4/GRIK5/CHRND/GABRA2/CHRNA4 | 12 |
| GO:0042627 | CC | GO:0042627 | chylomicron | 3/743 | 12/19886 | 0.008876796 | 0.081521598 | 0.07131948 | APOC1/APOB/APOA4 | 3 |
| GO:0007520 | BP | GO:0007520 | myoblast fusion | 6/695 | 49/18870 | 0.008883212 | 0.09289766 | 0.079149705 | NOS1/NPHS1/TNFSF14/CXCL9/CXCL10/MYOD1 | 6 |
| GO:0042220 | BP | GO:0042220 | response to cocaine | 6/695 | 49/18870 | 0.008883212 | 0.09289766 | 0.079149705 | SLC6A3/ABAT/CRHBP/EN1/PPP1R1B/TACR3 | 6 |
| GO:0042269 | BP | GO:0042269 | regulation of natural killer cell mediated cytotoxicity | 6/695 | 49/18870 | 0.008883212 | 0.09289766 | 0.079149705 | PIK3R6/HLA-G/CRTAM/LAG3/KLRK1/CLEC12B | 6 |
| GO:0019229 | BP | GO:0019229 | regulation of vasoconstriction | 7/695 | 64/18870 | 0.008982888 | 0.09372557 | 0.079855093 | CASR/AVPR1B/HRH2/AVPR2/FGG/FGA/FGB | 7 |
| GO:0030666 | CC | GO:0030666 | endocytic vesicle membrane | 15/743 | 202/19886 | 0.009068606 | 0.081562676 | 0.071355417 | SCARB1/EGF/FCGR1A/SLC15A2/HLA-G/AVPR2/EPGN/TYRP1/APOB/SYT7/SLC18A3/TF/GRIA4/WNT7B/CACNG2 | 15 |
| GO:0097553 | BP | GO:0097553 | calcium ion transmembrane import into cytosol | 15/695 | 205/18870 | 0.009095283 | 0.094682104 | 0.080670069 | NOL3/TRPV6/NOS1/TRPA1/FASLG/CXCL9/CXCL10/TRPV5/CXCL11/F2/P2RX2/EPO/SCN7A/SCN2A/SCN10A | 15 |
| GO:0034704 | CC | GO:0034704 | calcium channel complex | 8/743 | 79/19886 | 0.00924377 | 0.081562676 | 0.071355417 | HSPA2/TRPV6/PKD2L1/SCN7A/SCN2A/SCN10A/CATSPERD/CACNG2 | 8 |
| GO:0060065 | BP | GO:0060065 | uterus development | 4/695 | 23/18870 | 0.009243882 | 0.09601032 | 0.081801722 | WNT9B/STRA6/GATA3/LHX1 | 4 |
| GO:0018958 | BP | GO:0018958 | phenol-containing compound metabolic process | 10/695 | 114/18870 | 0.009429324 | 0.09771431 | 0.083253538 | SLC6A3/ABAT/IYD/DIO1/GATA3/TYRP1/DAO/TACR3/SNCB/SULT2A1 | 10 |
| GO:0008217 | BP | GO:0008217 | regulation of blood pressure | 14/695 | 187/18870 | 0.009517063 | 0.098400407 | 0.083838099 | WNK4/NOS1/ABAT/PTPRO/AVPR1B/AVPR2/UMOD/P2RX2/CYP4F2/SCNN1B/SCNN1G/TACR3/UTS2R/CALCA | 14 |
| GO:0140829 | MF | GO:0140829 | bicarbonate:monoatomic anion antiporter activity | 3/709 | 12/18496 | 0.009521616 | 0.082739562 | 0.07050489 | SLC26A4/SLC4A9/SLC4A1 | 3 |
| GO:0140900 | MF | GO:0140900 | chloride:bicarbonate antiporter activity | 3/709 | 12/18496 | 0.009521616 | 0.082739562 | 0.07050489 | SLC26A4/SLC4A9/SLC4A1 | 3 |
| GO:0005504 | MF | GO:0005504 | fatty acid binding | 6/709 | 48/18496 | 0.009686362 | 0.083214654 | 0.070909731 | FABP6/APOC1/FABP7/RBP2/ALB/FABP1 | 6 |
| GO:0046503 | BP | GO:0046503 | glycerolipid catabolic process | 7/695 | 65/18870 | 0.00975623 | 0.100073177 | 0.085263315 | SCARB1/APOC1/ENPP6/PLA2G7/APOB/PLA2G4D/APOA4 | 7 |
| GO:2000117 | BP | GO:2000117 | negative regulation of cysteine-type endopeptidase activity | 7/695 | 65/18870 | 0.00975623 | 0.100073177 | 0.085263315 | NOL3/VEGFA/CST7/DPEP1/MMP9/FABP1/MAGEA3 | 7 |
| GO:0007617 | BP | GO:0007617 | mating behavior | 5/695 | 36/18870 | 0.009781654 | 0.100073177 | 0.085263315 | ABAT/EDDM3A/SEMG1/PPP1R1B/MTNR1A | 5 |
| GO:1905332 | BP | GO:1905332 | positive regulation of morphogenesis of an epithelium | 5/695 | 36/18870 | 0.009781654 | 0.100073177 | 0.085263315 | VEGFA/ITGAX/EGF/GATA3/LHX1 | 5 |
| GO:0030850 | BP | GO:0030850 | prostate gland development | 6/695 | 50/18870 | 0.009788339 | 0.100073177 | 0.085263315 | SFRP1/ALOX15B/HOXD13/FGF10/HOXB13/BMP7 | 6 |
| GO:0051250 | BP | GO:0051250 | negative regulation of lymphocyte activation | 13/695 | 169/18870 | 0.009865957 | 0.100641566 | 0.085747588 | SOX11/IDO1/IL20RB/LILRB4/SFRP1/HLA-G/CRTAM/LAG3/RAG2/PLA2G2D/FOXJ1/PGLYRP2/VTCN1 | 13 |
| GO:0071674 | BP | GO:0071674 | mononuclear cell migration | 15/695 | 207/18870 | 0.009893711 | 0.100699913 | 0.0857973 | PLA2G7/PTPRO/CCL18/CCL5/TNFSF14/CXCL10/CRTAM/CXCL11/KLRK1/GATA3/CXCL13/CCL20/SAA1/CCL25/CALCA | 15 |
| GO:0050708 | BP | GO:0050708 | regulation of protein secretion | 18/695 | 266/18870 | 0.009921787 | 0.10076126 | 0.085849568 | TREM2/INHBB/SFRP1/ABAT/CCL5/CASR/IFNG/TFAP2B/F2/FGG/SYT7/SSTR5/SAA1/SLC30A8/ADCY8/FGA/FGB/KRT20 | 18 |
| GO:0032496 | BP | GO:0032496 | response to lipopolysaccharide | 22/695 | 348/18870 | 0.010084964 | 0.102191325 | 0.087067997 | SCARB1/TREM2/IDO1/CD68/NOS1/FASLG/GBP5/AICDA/CXCL9/CXCL10/CYP27B1/CXCL11/UMOD/TNIP3/KLRK1/MMP9/PTGER1/CXCL5/CXCL13/EPO/FGF10/LBP | 22 |
| GO:0035592 | BP | GO:0035592 | establishment of protein localization to extracellular region | 23/695 | 369/18870 | 0.010143323 | 0.102377647 | 0.087226745 | TREM2/INHBB/SFRP1/PTPRN/ABAT/CCL5/CASR/IFNG/CTAGE9/SCG2/TFAP2B/AFM/FAM3B/F2/FGG/SYT7/SSTR5/SAA1/SLC30A8/ADCY8/FGA/FGB/KRT20 | 23 |
| GO:0050767 | BP | GO:0050767 | regulation of neurogenesis | 24/695 | 390/18870 | 0.01015452 | 0.102377647 | 0.087226745 | TREM2/VEGFA/SEMA5B/SOX11/PROX1/DLX1/HAPLN1/IFNG/CDH4/TP73/ZNF488/HELT/HES5/ITPKA/F2/DLX2/RASSF10/NKX6-2/NR2E1/MT3/LHX2/L1CAM/POU4F1/BMP7 | 24 |
| GO:0061041 | BP | GO:0061041 | regulation of wound healing | 11/695 | 133/18870 | 0.010170558 | 0.102377647 | 0.087226745 | CLDN19/HRG/KNG1/F2/F11/FGG/TSPAN8/FGA/PLG/FGB/REG3A | 11 |
| GO:0019724 | BP | GO:0019724 | B cell mediated immunity | 15/695 | 208/18870 | 0.010313145 | 0.103584775 | 0.088255231 | TREM2/CD70/FCGR3A/IL2RB/FCGR1A/C3/AICDA/HLA-G/BATF/CD27/SUSD4/C7/CR2/FOXJ1/MBL2 | 15 |
| GO:0050909 | BP | GO:0050909 | sensory perception of taste | 7/695 | 66/18870 | 0.010577045 | 0.104709928 | 0.089213871 | RTP2/PKD2L1/P2RX2/SCNN1B/RTP5/SCNN1G/LCN1 | 7 |
| GO:0022848 | MF | GO:0022848 | acetylcholine-gated monoatomic cation-selective channel activity | 4/709 | 23/18496 | 0.010602582 | 0.089655181 | 0.076397899 | CHRNA1/CHRNA6/CHRND/CHRNA4 | 4 |
| GO:0033293 | MF | GO:0033293 | monocarboxylic acid binding | 8/709 | 79/18496 | 0.010673236 | 0.089655181 | 0.076397899 | FABP6/APOC1/FABP7/RBP2/ALB/SERPINA5/UGT1A3/FABP1 | 8 |
| GO:0010043 | BP | GO:0010043 | response to zinc ion | 6/695 | 51/18870 | 0.010757218 | 0.104709928 | 0.089213871 | MT1G/MT1H/SLC30A2/MT3/SLC30A8/SLC30A10 | 6 |
| GO:0035036 | BP | GO:0035036 | sperm-egg recognition | 6/695 | 51/18870 | 0.010757218 | 0.104709928 | 0.089213871 | ADAM18/ATP8B3/ZP1/PAEP/ZAN/FOLR3 | 6 |
| GO:0030101 | BP | GO:0030101 | natural killer cell activation | 9/695 | 99/18870 | 0.01077483 | 0.104709928 | 0.089213871 | FCGR3A/NKG7/CD2/ZNF683/KLRK1/GATA3/PGLYRP2/KLRF2/IFNA14 | 9 |
| GO:0002922 | BP | GO:0002922 | positive regulation of humoral immune response | 4/695 | 24/18870 | 0.010775597 | 0.104709928 | 0.089213871 | TREM2/C3/KLK7/KLK5 | 4 |
| GO:0021871 | BP | GO:0021871 | forebrain regionalization | 4/695 | 24/18870 | 0.010775597 | 0.104709928 | 0.089213871 | EOMES/WNT7B/LHX2/LHX1 | 4 |
| GO:0030539 | BP | GO:0030539 | male genitalia development | 4/695 | 24/18870 | 0.010775597 | 0.104709928 | 0.089213871 | WNT9B/HSD17B3/HOXD13/FGF10 | 4 |
| GO:0034114 | BP | GO:0034114 | regulation of heterotypic cell-cell adhesion | 4/695 | 24/18870 | 0.010775597 | 0.104709928 | 0.089213871 | FGG/FGA/BMP7/FGB | 4 |
| GO:0060571 | BP | GO:0060571 | morphogenesis of an epithelial fold | 4/695 | 24/18870 | 0.010775597 | 0.104709928 | 0.089213871 | HOXD13/FGF10/OVOL2/BMP7 | 4 |
| GO:2001026 | BP | GO:2001026 | regulation of endothelial cell chemotaxis | 4/695 | 24/18870 | 0.010775597 | 0.104709928 | 0.089213871 | VEGFA/FGF1/HRG/CXCL13 | 4 |
| GO:0002664 | BP | GO:0002664 | regulation of T cell tolerance induction | 3/695 | 13/18870 | 0.010791767 | 0.104709928 | 0.089213871 | IDO1/LILRB4/HLA-G | 3 |
| GO:0007512 | BP | GO:0007512 | adult heart development | 3/695 | 13/18870 | 0.010791767 | 0.104709928 | 0.089213871 | MYH7/NKX2-5/APELA | 3 |
| GO:0009415 | BP | GO:0009415 | response to water | 3/695 | 13/18870 | 0.010791767 | 0.104709928 | 0.089213871 | AQP2/UMOD/PKD2L1 | 3 |
| GO:0032490 | BP | GO:0032490 | detection of molecule of bacterial origin | 3/695 | 13/18870 | 0.010791767 | 0.104709928 | 0.089213871 | SCARB1/TREM2/LBP | 3 |
| GO:0051918 | BP | GO:0051918 | negative regulation of fibrinolysis | 3/695 | 13/18870 | 0.010791767 | 0.104709928 | 0.089213871 | HRG/F2/PLG | 3 |
| GO:0070234 | BP | GO:0070234 | positive regulation of T cell apoptotic process | 3/695 | 13/18870 | 0.010791767 | 0.104709928 | 0.089213871 | IDO1/CCL5/PDCD1 | 3 |
| GO:0005200 | MF | GO:0005200 | structural constituent of cytoskeleton | 10/709 | 112/18496 | 0.010882555 | 0.090408917 | 0.07704018 | TUBA3D/SPTBN2/TUBA3E/EPB41L4B/TUBA3C/TUBAL3/KRT6A/KRT20/ACTL6B/KRT6B | 10 |
| GO:0060205 | CC | GO:0060205 | cytoplasmic vesicle lumen | 21/743 | 325/19886 | 0.010895021 | 0.093760777 | 0.082026972 | VEGFA/EGF/VWF/RNASET2/C3/HRG/FASLG/KNG1/CTSW/ALB/CHIT1/HP/FGG/OLFM4/TF/FOLR3/ORM2/FGA/ORM1/PLG/FGB | 21 |
| GO:0031076 | BP | GO:0031076 | embryonic camera-type eye development | 5/695 | 37/18870 | 0.010975406 | 0.106041451 | 0.090348342 | SOX11/PROX1/STRA6/FGF10/BMP7 | 5 |
| GO:0070269 | BP | GO:0070269 | pyroptosis | 5/695 | 37/18870 | 0.010975406 | 0.106041451 | 0.090348342 | TREM2/GZMA/GBP5/AIM2/GSDMC | 5 |
| GO:0044194 | CC | GO:0044194 | cytolytic granule | 3/743 | 13/19886 | 0.011223298 | 0.093760777 | 0.082026972 | NKG7/GNLY/GZMH | 3 |
| GO:0031983 | CC | GO:0031983 | vesicle lumen | 21/743 | 326/19886 | 0.011251293 | 0.093760777 | 0.082026972 | VEGFA/EGF/VWF/RNASET2/C3/HRG/FASLG/KNG1/CTSW/ALB/CHIT1/HP/FGG/OLFM4/TF/FOLR3/ORM2/FGA/ORM1/PLG/FGB | 21 |
| GO:0001895 | BP | GO:0001895 | retina homeostasis | 8/695 | 83/18870 | 0.011330778 | 0.10901401 | 0.092880992 | ESRRB/ABCA4/SLC28A2/ALB/CDHR1/TF/LCN1/PCDH15 | 8 |
| GO:0031424 | BP | GO:0031424 | keratinization | 8/695 | 83/18870 | 0.011330778 | 0.10901401 | 0.092880992 | KRT81/KRT72/KRT6A/KLK5/KRT78/KRT6B/SPRR3/LCE2D | 8 |
| GO:0048705 | BP | GO:0048705 | skeletal system morphogenesis | 16/695 | 230/18870 | 0.011390006 | 0.109211615 | 0.093049353 | SOX11/UNCX/DLX5/SFRP1/WNT9B/TBX15/DLX2/HOXB9/FREM1/ALX1/PAPPA2/GRHL2/MMP13/LHX1/IFITM5/BMP7 | 16 |
| GO:0006066 | BP | GO:0006066 | alcohol metabolic process | 23/695 | 373/18870 | 0.011431987 | 0.109211615 | 0.093049353 | SCARB1/APOC1/FGF1/AVPR1B/CYP27B1/PTH1R/CEL/ITPKA/ADH1C/SPTSSB/UGT1A3/APOB/SULT2B1/SLC34A1/MOGAT2/SCNN1B/ALDH3B2/SERPINA12/RDH8/UGT1A4/HAO1/SULT2A1/APOA4 | 23 |
| GO:0001655 | BP | GO:0001655 | urogenital system development | 7/695 | 67/18870 | 0.011446907 | 0.109211615 | 0.093049353 | SFRP1/ALOX15B/HOXD13/FGF10/HOXB13/LHX1/BMP7 | 7 |
| GO:0006956 | BP | GO:0006956 | complement activation | 7/695 | 67/18870 | 0.011446907 | 0.109211615 | 0.093049353 | TREM2/C3/SUSD4/CD5L/C7/CR2/MBL2 | 7 |
| GO:0015101 | MF | GO:0015101 | organic cation transmembrane transporter activity | 5/709 | 36/18496 | 0.011494974 | 0.094458699 | 0.080491122 | SLC6A3/SLC7A8/SLC5A7/SLC47A2/SLC18A3 | 5 |
| GO:0006968 | BP | GO:0006968 | cellular defense response | 6/695 | 52/18870 | 0.011792081 | 0.111890704 | 0.095331962 | GNLY/HLA-G/CXCL9/UMOD/CD5L/LBP | 6 |
| GO:0043154 | BP | GO:0043154 | negative regulation of cysteine-type endopeptidase activity involved in apoptotic process | 6/695 | 52/18870 | 0.011792081 | 0.111890704 | 0.095331962 | NOL3/VEGFA/DPEP1/MMP9/FABP1/MAGEA3 | 6 |
| GO:0071466 | BP | GO:0071466 | cellular response to xenobiotic stimulus | 14/695 | 192/18870 | 0.011821377 | 0.111890704 | 0.095331962 | GSTM3/CYP2J2/WNK4/NOS1/CRHBP/SLC28A2/DPEP1/AIM2/UGT1A3/CYP4F2/CYP2B6/SLC22A7/UGT1A10/SULT2A1 | 14 |
| GO:0007599 | BP | GO:0007599 | hemostasis | 16/695 | 231/18870 | 0.011835421 | 0.111890704 | 0.095331962 | VWF/HRG/KNG1/TREML1/AVPR2/F2/PROZ/F11/FGG/CYP4F2/TSPAN8/SAA1/FGA/PLG/SLC4A1/FGB | 16 |
| GO:0007204 | BP | GO:0007204 | positive regulation of cytosolic calcium ion concentration | 13/695 | 173/18870 | 0.011850131 | 0.111890704 | 0.095331962 | NOS1/MCHR1/KNG1/AVPR1B/CXCR3/PTGER1/CXCL13/P2RX2/EPO/SAA1/NMUR2/ADCY8/CALCA | 13 |
| GO:0050870 | BP | GO:0050870 | positive regulation of T cell activation | 17/695 | 251/18870 | 0.01189445 | 0.111905444 | 0.095344521 | CD70/PIK3R6/LILRB4/TNFSF9/CCL5/HLA-G/TNFSF14/ICOS/SIRPG/IFNG/CD27/KLRK1/GATA3/HHLA2/EPO/VTCN1/ACTL6B | 17 |
| GO:0034219 | BP | GO:0034219 | carbohydrate transmembrane transport | 11/695 | 136/18870 | 0.011900666 | 0.111905444 | 0.095344521 | HK2/C3/TRIB3/SLC5A2/AQP2/GPC3/ITLN1/SLC2A12/TERT/SLC5A11/AQP9 | 11 |
| GO:1903706 | BP | GO:1903706 | regulation of hemopoiesis | 25/695 | 417/18870 | 0.011984023 | 0.112457875 | 0.095815197 | TREM2/TNFAIP6/MTURN/PIK3R6/LILRB4/TNFSF9/SFRP1/LOX/TMEM178A/HLA-G/CD2/ZNF683/BATF/CRTAM/IFNG/LAG3/CD27/RAG2/GATA3/DCSTAMP/FOXJ1/PGLYRP2/POU4F1/FSHB/ACTL6B | 25 |
| GO:0008035 | MF | GO:0008035 | high-density lipoprotein particle binding | 3/709 | 13/18496 | 0.012029984 | 0.096751787 | 0.082445132 | SCARB1/TREM2/APOL5 | 3 |
| GO:0015166 | MF | GO:0015166 | polyol transmembrane transporter activity | 3/709 | 13/18496 | 0.012029984 | 0.096751787 | 0.082445132 | AQP2/SLC5A11/AQP9 | 3 |
| GO:0050867 | BP | GO:0050867 | positive regulation of cell activation | 24/695 | 396/18870 | 0.012074907 | 0.113078536 | 0.096344007 | TREM2/CD70/TNFRSF4/FCGR3A/PIK3R6/LILRB4/TNFSF9/CCL5/HLA-G/CD2/TNFSF14/ICOS/SIRPG/IFNG/CD27/KLRK1/HAMP/GATA3/HHLA2/EPO/VTCN1/FGF10/LBP/ACTL6B | 24 |
| GO:0007339 | BP | GO:0007339 | binding of sperm to zona pellucida | 5/695 | 38/18870 | 0.012264817 | 0.114388194 | 0.097459848 | ADAM18/ATP8B3/ZP1/PAEP/ZAN | 5 |
| GO:0048536 | BP | GO:0048536 | spleen development | 5/695 | 38/18870 | 0.012264817 | 0.114388194 | 0.097459848 | TCF21/FGF10/BARX1/NKX2-5/NKX2-3 | 5 |
| GO:0072010 | BP | GO:0072010 | glomerular epithelium development | 4/695 | 25/18870 | 0.012461945 | 0.115989995 | 0.098824598 | PTPRO/NPHS1/NPHS2/FOXJ1 | 4 |
| GO:0007411 | BP | GO:0007411 | axon guidance | 16/695 | 233/18870 | 0.01276726 | 0.118349652 | 0.100835048 | VEGFA/SEMA5B/DLX5/PTPRO/CDH4/UNC5A/GATA3/CHL1/CNTN1/GBX2/LHX2/L1CAM/ISL2/LHX1/ARX/BMP7 | 16 |
| GO:0097485 | BP | GO:0097485 | neuron projection guidance | 16/695 | 233/18870 | 0.01276726 | 0.118349652 | 0.100835048 | VEGFA/SEMA5B/DLX5/PTPRO/CDH4/UNC5A/GATA3/CHL1/CNTN1/GBX2/LHX2/L1CAM/ISL2/LHX1/ARX/BMP7 | 16 |
| GO:0071692 | BP | GO:0071692 | protein localization to extracellular region | 23/695 | 377/18870 | 0.012849257 | 0.118589607 | 0.101039492 | TREM2/INHBB/SFRP1/PTPRN/ABAT/CCL5/CASR/IFNG/CTAGE9/SCG2/TFAP2B/AFM/FAM3B/F2/FGG/SYT7/SSTR5/SAA1/SLC30A8/ADCY8/FGA/FGB/KRT20 | 23 |
| GO:0048754 | BP | GO:0048754 | branching morphogenesis of an epithelial tube | 12/695 | 156/18870 | 0.012861696 | 0.118589607 | 0.101039492 | VEGFA/FGF1/EGF/TCF21/WNT9B/CASR/GPC3/TACSTD2/GBX2/FGF10/LHX1/BMP7 | 12 |
| GO:0005109 | MF | GO:0005109 | frizzled binding | 5/709 | 37/18496 | 0.012882314 | 0.101448221 | 0.086447105 | WNT8B/SFRP1/WNT9B/CTHRC1/WNT7B | 5 |
| GO:0005231 | MF | GO:0005231 | excitatory extracellular ligand-gated monoatomic ion channel activity | 5/709 | 37/18496 | 0.012882314 | 0.101448221 | 0.086447105 | CHRNA1/P2RX2/CHRNA6/CHRND/CHRNA4 | 5 |
| GO:0002715 | BP | GO:0002715 | regulation of natural killer cell mediated immunity | 6/695 | 53/18870 | 0.012895115 | 0.118589607 | 0.101039492 | PIK3R6/HLA-G/CRTAM/LAG3/KLRK1/CLEC12B | 6 |
| GO:0031214 | BP | GO:0031214 | biomineral tissue development | 13/695 | 175/18870 | 0.012951416 | 0.118589607 | 0.101039492 | PTHLH/LOX/CYP27B1/PTH1R/GPC3/IBSP/NELL1/DMP1/MMP13/IFITM5/BMP7/AMELY/KLK4 | 13 |
| GO:0002028 | BP | GO:0002028 | regulation of sodium ion transport | 8/695 | 85/18870 | 0.012974793 | 0.118589607 | 0.101039492 | WNK4/NOS1/CHP2/NKAIN1/HECW1/FXYD4/CNTN1/NKX2-5 | 8 |
| GO:0034103 | BP | GO:0034103 | regulation of tissue remodeling | 8/695 | 85/18870 | 0.012974793 | 0.118589607 | 0.101039492 | SFRP1/HRG/HAMP/DCSTAMP/SYT7/TF/FSHB/CALCA | 8 |
| GO:0051101 | BP | GO:0051101 | regulation of DNA binding | 8/695 | 85/18870 | 0.012974793 | 0.118589607 | 0.101039492 | SOX11/EGF/GZMA/PYHIN1/IFNG/MMP9/GATA3/POU4F1 | 8 |
| GO:0098992 | CC | GO:0098992 | neuronal dense core vesicle | 4/743 | 25/19886 | 0.01308501 | 0.107059175 | 0.093661126 | SCG2/P2RX2/SST/CALCA | 4 |
| GO:0002064 | BP | GO:0002064 | epithelial cell development | 15/695 | 214/18870 | 0.013131967 | 0.119786609 | 0.102059349 | VEGFA/GSTM3/VSIG1/PROX1/NPHS1/SLC9A4/NPHS2/WNT7B/FOXJ1/SPDEF/GRHL2/TFCP2L1/RAB25/HOXB13/DMRT1 | 15 |
| GO:0005254 | MF | GO:0005254 | chloride channel activity | 8/709 | 82/18496 | 0.013204909 | 0.102916612 | 0.087698366 | GABRD/CLIC5/ANO4/CLCNKA/NMUR2/GABRA2/CLCNKB/BSND | 8 |
| GO:0002704 | BP | GO:0002704 | negative regulation of leukocyte mediated immunity | 7/695 | 69/18870 | 0.013339969 | 0.120226275 | 0.10243395 | IL20RB/LILRB4/HLA-G/SUSD4/CLEC12B/CR2/FOXJ1 | 7 |
| GO:0030049 | BP | GO:0030049 | muscle filament sliding | 3/695 | 14/18870 | 0.013364321 | 0.120226275 | 0.10243395 | TNNT2/TNNC1/MYH7 | 3 |
| GO:0042136 | BP | GO:0042136 | neurotransmitter biosynthetic process | 3/695 | 14/18870 | 0.013364321 | 0.120226275 | 0.10243395 | NOS1/SLC5A7/CHAT | 3 |
| GO:0045605 | BP | GO:0045605 | negative regulation of epidermal cell differentiation | 3/695 | 14/18870 | 0.013364321 | 0.120226275 | 0.10243395 | HES5/GRHL2/REG3A | 3 |
| GO:0045683 | BP | GO:0045683 | negative regulation of epidermis development | 3/695 | 14/18870 | 0.013364321 | 0.120226275 | 0.10243395 | HES5/GRHL2/REG3A | 3 |
| GO:0051547 | BP | GO:0051547 | regulation of keratinocyte migration | 3/695 | 14/18870 | 0.013364321 | 0.120226275 | 0.10243395 | MMP9/EPB41L4B/FGF10 | 3 |
| GO:0098883 | BP | GO:0098883 | synapse pruning | 3/695 | 14/18870 | 0.013364321 | 0.120226275 | 0.10243395 | TREM2/C3/C1QL1 | 3 |
| GO:0010634 | BP | GO:0010634 | positive regulation of epithelial cell migration | 13/695 | 176/18870 | 0.013530798 | 0.121484768 | 0.103506197 | SCARB1/VEGFA/FGF1/EGF/PROX1/IFNG/MMP9/GATA3/EPB41L4B/FGF10/RAB25/PLG/FGFBP1 | 13 |
| GO:0070229 | BP | GO:0070229 | negative regulation of lymphocyte apoptotic process | 5/695 | 39/18870 | 0.013653122 | 0.122342681 | 0.104237148 | IDO1/CCL5/AURKB/CD27/PDCD1 | 5 |
| GO:0043177 | MF | GO:0043177 | organic acid binding | 15/709 | 207/18496 | 0.013830386 | 0.106691551 | 0.090915106 | EGLN3/FABP6/SIGLEC8/ST8SIA4/APOC1/FABP7/RBP2/NOS1/CASR/ALB/SERPINA5/AGXT/UGT1A3/FABP1/FOLR3 | 15 |
| GO:0034703 | CC | GO:0034703 | cation channel complex | 14/743 | 193/19886 | 0.013844633 | 0.107791877 | 0.094302133 | GRIK3/HSPA2/TRPV6/KCNN1/PKD2L1/SCN7A/SCNN1B/SCN2A/SCNN1G/GRIK5/DPP6/SCN10A/CATSPERD/CACNG2 | 14 |
| GO:0001518 | CC | GO:0001518 | voltage-gated sodium channel complex | 3/743 | 14/19886 | 0.013893175 | 0.107791877 | 0.094302133 | SCN7A/SCN2A/SCN10A | 3 |
| GO:1990023 | CC | GO:1990023 | mitotic spindle midzone | 3/743 | 14/19886 | 0.013893175 | 0.107791877 | 0.094302133 | OR2A4/KIF18B/AURKB | 3 |
| GO:0043588 | BP | GO:0043588 | skin development | 20/695 | 317/18870 | 0.014001481 | 0.125218721 | 0.106687562 | COL5A3/ALOX15B/CYP27B1/TFAP2B/KRT81/SLITRK5/KRT36/KRT72/LHX2/GRHL2/FGF10/KRT25/OVOL2/KRT6A/KLK5/KRT78/REG3A/KRT6B/SPRR3/LCE2D | 20 |
| GO:0010883 | BP | GO:0010883 | regulation of lipid storage | 6/695 | 54/18870 | 0.014068453 | 0.12557193 | 0.106988499 | HILPDA/SCARB1/TREM2/C3/APOB/CRP | 6 |
| GO:0098754 | BP | GO:0098754 | detoxification | 12/695 | 158/18870 | 0.014114217 | 0.125734834 | 0.107127295 | GSTM3/SLC15A2/ALB/SLC47A2/MT1G/HP/FABP1/MT1H/MT3/BMP7/SLC30A10/APOA4 | 12 |
| GO:0006063 | BP | GO:0006063 | uronic acid metabolic process | 4/695 | 26/18870 | 0.014307983 | 0.125745154 | 0.107136088 | UGT1A3/UGT1A10/UGT2A1/UGT1A4 | 4 |
| GO:0019585 | BP | GO:0019585 | glucuronate metabolic process | 4/695 | 26/18870 | 0.014307983 | 0.125745154 | 0.107136088 | UGT1A3/UGT1A10/UGT2A1/UGT1A4 | 4 |
| GO:0048596 | BP | GO:0048596 | embryonic camera-type eye morphogenesis | 4/695 | 26/18870 | 0.014307983 | 0.125745154 | 0.107136088 | SOX11/PROX1/STRA6/BMP7 | 4 |
| GO:0070193 | BP | GO:0070193 | synaptonemal complex organization | 4/695 | 26/18870 | 0.014307983 | 0.125745154 | 0.107136088 | HSPA2/TEX15/TEX11/HORMAD1 | 4 |
| GO:0071280 | BP | GO:0071280 | cellular response to copper ion | 4/695 | 26/18870 | 0.014307983 | 0.125745154 | 0.107136088 | AQP2/MT1G/MT1H/MT3 | 4 |
| GO:0097186 | BP | GO:0097186 | amelogenesis | 4/695 | 26/18870 | 0.014307983 | 0.125745154 | 0.107136088 | DMP1/AMELY/KLK5/KLK4 | 4 |
| GO:0098581 | BP | GO:0098581 | detection of external biotic stimulus | 4/695 | 26/18870 | 0.014307983 | 0.125745154 | 0.107136088 | SCARB1/TREM2/PGLYRP2/LBP | 4 |
| GO:0050922 | BP | GO:0050922 | negative regulation of chemotaxis | 7/695 | 70/18870 | 0.014366216 | 0.126014602 | 0.10736566 | SEMA5B/TNFAIP6/ANGPT2/HRG/PTPRO/KLRK1/CXCL13 | 7 |
| GO:0070555 | BP | GO:0070555 | response to interleukin-1 | 11/695 | 140/18870 | 0.014545762 | 0.127345084 | 0.108499244 | MYLK3/INHBB/SFRP1/CCL18/CCL5/FGG/CCL20/EPO/SLC30A8/CCL25/FGB | 11 |
| GO:0021782 | BP | GO:0021782 | glial cell development | 10/695 | 122/18870 | 0.014738587 | 0.128632457 | 0.109596097 | TREM2/KCNJ10/SOX11/LGI4/IFNG/ZNF488/HES5/MAL/CNTN1/NKX6-2 | 10 |
| GO:0060538 | BP | GO:0060538 | skeletal muscle organ development | 13/695 | 178/18870 | 0.014749104 | 0.128632457 | 0.109596097 | SOX11/TCF21/NPHS1/EOMES/MYOZ2/CHRNA1/MSC/STRA6/P2RX2/CHRND/ANKRD33/PITX1/MYOD1 | 13 |
| GO:0071277 | BP | GO:0071277 | cellular response to calcium ion | 8/695 | 87/18870 | 0.014786916 | 0.12871658 | 0.109667771 | INHBB/CPNE6/CRHBP/CHP2/DPEP1/ITPKA/SYT7/ADCY8 | 8 |
| GO:0015386 | MF | GO:0015386 | potassium:proton antiporter activity | 3/709 | 14/18496 | 0.014881186 | 0.113638145 | 0.096834509 | SLC9A3/SLC9A4/SLC9A2 | 3 |
| GO:0072341 | MF | GO:0072341 | modified amino acid binding | 8/709 | 84/18496 | 0.015120156 | 0.114308377 | 0.097405634 | SCARB1/TREM2/CD300LF/CPNE6/DPEP1/SYT7/GSDMC/FOLR3 | 8 |
| GO:0003352 | BP | GO:0003352 | regulation of cilium movement | 5/695 | 40/18870 | 0.015143406 | 0.131070772 | 0.111673566 | DNAH11/TPPP2/SEMG2/SEMG1/TACR3 | 5 |
| GO:0009069 | BP | GO:0009069 | serine family amino acid metabolic process | 5/695 | 40/18870 | 0.015143406 | 0.131070772 | 0.111673566 | SDS/AGXT/DAO/BAAT/HAO1 | 5 |
| GO:0009595 | BP | GO:0009595 | detection of biotic stimulus | 5/695 | 40/18870 | 0.015143406 | 0.131070772 | 0.111673566 | SCARB1/TREM2/CRTAM/PGLYRP2/LBP | 5 |
| GO:0046887 | BP | GO:0046887 | positive regulation of hormone secretion | 11/695 | 141/18870 | 0.015271311 | 0.131799913 | 0.112294801 | SOX11/INHBB/ABAT/CASR/TFR2/F2/FGG/SLC30A8/ADCY8/FGA/FGB | 11 |
| GO:0007140 | BP | GO:0007140 | male meiotic nuclear division | 6/695 | 55/18870 | 0.015314169 | 0.131799913 | 0.112294801 | HSPA2/TEX15/TEX11/RBM46/DNMT3L/DMRTC2 | 6 |
| GO:0014888 | BP | GO:0014888 | striated muscle adaptation | 6/695 | 55/18870 | 0.015314169 | 0.131799913 | 0.112294801 | MYOZ2/TNNI1/TNNC1/TNNT1/MYH7/MYOD1 | 6 |
| GO:0071772 | BP | GO:0071772 | response to BMP | 13/695 | 179/18870 | 0.015388856 | 0.131945724 | 0.112419034 | TNFAIP6/SOX11/DLX5/SFRP1/SOST/DLX1/GDF6/GPC3/TFAP2B/HES5/GATA3/BMP7/GATA4 | 13 |
| GO:0071773 | BP | GO:0071773 | cellular response to BMP stimulus | 13/695 | 179/18870 | 0.015388856 | 0.131945724 | 0.112419034 | TNFAIP6/SOX11/DLX5/SFRP1/SOST/DLX1/GDF6/GPC3/TFAP2B/HES5/GATA3/BMP7/GATA4 | 13 |
| GO:0046545 | BP | GO:0046545 | development of primary female sexual characteristics | 9/695 | 105/18870 | 0.015432327 | 0.132070664 | 0.112525484 | VEGFA/TNFAIP6/INHBB/SFRP1/PTPRN/MSH4/DACH2/LHX8/FSHB | 9 |
| GO:0010811 | BP | GO:0010811 | positive regulation of cell-substrate adhesion | 10/695 | 123/18870 | 0.015532988 | 0.132189486 | 0.112626721 | VEGFA/SFRP1/HRG/COL26A1/FGG/OLFM4/DMP1/CCL25/FGA/FGB | 10 |
| GO:0030282 | BP | GO:0030282 | bone mineralization | 10/695 | 123/18870 | 0.015532988 | 0.132189486 | 0.112626721 | PTHLH/LOX/CYP27B1/PTH1R/GPC3/IBSP/NELL1/MMP13/IFITM5/BMP7 | 10 |
| GO:0071774 | BP | GO:0071774 | response to fibroblast growth factor | 10/695 | 123/18870 | 0.015532988 | 0.132189486 | 0.112626721 | FGF1/SFRP1/CCL5/CASR/FLRT1/GATA3/CXCL13/FGF10/LHX1/FGFBP1 | 10 |
| GO:0030073 | BP | GO:0030073 | insulin secretion | 14/695 | 199/18870 | 0.015750724 | 0.133545102 | 0.113781718 | INHBB/SFRP1/PTPRN/ABAT/CCL5/CASR/IFNG/TFAP2B/FAM3B/F2/SYT7/SSTR5/SLC30A8/ADCY8 | 14 |
| GO:1902075 | BP | GO:1902075 | cellular response to salt | 14/695 | 199/18870 | 0.015750724 | 0.133545102 | 0.113781718 | KCNJ10/INHBB/CPNE6/CRHBP/CHP2/AQP2/DPEP1/ITPKA/LY6H/SYT7/SLC13A2/PPP1R1B/CHRND/ADCY8 | 14 |
| GO:0009071 | BP | GO:0009071 | serine family amino acid catabolic process | 3/695 | 15/18870 | 0.016255489 | 0.135340934 | 0.115311785 | SDS/AGXT/DAO | 3 |
| GO:0010820 | BP | GO:0010820 | positive regulation of T cell chemotaxis | 3/695 | 15/18870 | 0.016255489 | 0.135340934 | 0.115311785 | CCL5/TNFSF14/CXCL13 | 3 |
| GO:0033189 | BP | GO:0033189 | response to vitamin A | 3/695 | 15/18870 | 0.016255489 | 0.135340934 | 0.115311785 | EPO/SLC34A1/GATA4 | 3 |
| GO:0034310 | BP | GO:0034310 | primary alcohol catabolic process | 3/695 | 15/18870 | 0.016255489 | 0.135340934 | 0.115311785 | ALDH3B2/HAO1/SULT2A1 | 3 |
| GO:0060413 | BP | GO:0060413 | atrial septum morphogenesis | 3/695 | 15/18870 | 0.016255489 | 0.135340934 | 0.115311785 | TBX5/NKX2-5/GATA4 | 3 |
| GO:0071599 | BP | GO:0071599 | otic vesicle development | 3/695 | 15/18870 | 0.016255489 | 0.135340934 | 0.115311785 | PROX1/GATA3/FGF10 | 3 |
| GO:0072148 | BP | GO:0072148 | epithelial cell fate commitment | 3/695 | 15/18870 | 0.016255489 | 0.135340934 | 0.115311785 | PROX1/SPDEF/ARX | 3 |
| GO:0002366 | BP | GO:0002366 | leukocyte activation involved in immune response | 19/695 | 301/18870 | 0.01625639 | 0.135340934 | 0.115311785 | TREM2/ENPP3/FCGR3A/NKG7/EOMES/AICDA/ZNF683/BATF/LAT/IFNG/RAG2/GATA3/SCNN1B/PGLYRP2/KLRF2/LBP/IFNA14/CPLX2/NKX2-3 | 19 |
| GO:0010884 | BP | GO:0010884 | positive regulation of lipid storage | 4/695 | 27/18870 | 0.016318285 | 0.135340934 | 0.115311785 | HILPDA/SCARB1/C3/APOB | 4 |
| GO:0015740 | BP | GO:0015740 | C4-dicarboxylate transport | 4/695 | 27/18870 | 0.016318285 | 0.135340934 | 0.115311785 | ABAT/SLC13A3/SLC13A2/SLC7A13 | 4 |
| GO:0031639 | BP | GO:0031639 | plasminogen activation | 4/695 | 27/18870 | 0.016318285 | 0.135340934 | 0.115311785 | F11/FGG/FGA/FGB | 4 |
| GO:0071467 | BP | GO:0071467 | cellular response to pH | 4/695 | 27/18870 | 0.016318285 | 0.135340934 | 0.115311785 | PKD2L1/SCNN1B/INSRR/SCNN1G | 4 |
| GO:0005891 | CC | GO:0005891 | voltage-gated calcium channel complex | 6/743 | 55/19886 | 0.016336675 | 0.124601755 | 0.109008319 | HSPA2/SCN7A/SCN2A/SCN10A/CATSPERD/CACNG2 | 6 |
| GO:0050678 | BP | GO:0050678 | regulation of epithelial cell proliferation | 24/695 | 407/18870 | 0.016347526 | 0.135340934 | 0.115311785 | VEGFA/SOX11/FGF1/PGF/EGF/DLX5/SFRP1/PTPRN/PROX1/EPGN/GPC3/SCG2/HES5/GATA3/TACSTD2/FGF10/OVOL2/HMX2/NKX2-5/ARX/APELA/REG3A/FGFBP1/NKX2-3 | 24 |
| GO:0010810 | BP | GO:0010810 | regulation of cell-substrate adhesion | 15/695 | 220/18870 | 0.016517018 | 0.13649688 | 0.116296662 | CDKN2A/VEGFA/ANGPT2/SFRP1/HRG/PTPRO/COL26A1/FGG/TACSTD2/OLFM4/DMP1/CCL25/FGA/PLG/FGB | 15 |
| GO:0060411 | BP | GO:0060411 | cardiac septum morphogenesis | 7/695 | 72/18870 | 0.016585568 | 0.136815964 | 0.116568525 | SOX11/DNAH11/PROX1/TBX5/NKX2-5/BMP7/GATA4 | 7 |
| GO:0042417 | BP | GO:0042417 | dopamine metabolic process | 5/695 | 41/18870 | 0.016738601 | 0.137829561 | 0.11743212 | SLC6A3/ABAT/DAO/TACR3/SNCB | 5 |
| GO:0048592 | BP | GO:0048592 | eye morphogenesis | 12/695 | 162/18870 | 0.016895394 | 0.138621097 | 0.118106515 | VEGFA/CALB1/SOX11/PROX1/FASLG/TFAP2B/STRA6/OLFM3/VSX1/LHX1/BMP7/SOX1 | 12 |
| GO:0120254 | BP | GO:0120254 | olefinic compound metabolic process | 12/695 | 162/18870 | 0.016895394 | 0.138621097 | 0.118106515 | CYP2J2/ABCA4/ALOX15B/ELOVL2/CEL/ADH1C/HSD17B3/CYP4F2/SCNN1B/CYP2B6/RDH8/FSHB | 12 |
| GO:0002698 | BP | GO:0002698 | negative regulation of immune effector process | 10/695 | 125/18870 | 0.017216674 | 0.140751697 | 0.119921808 | ENPP3/IL20RB/LILRB4/HLA-G/SUSD4/CLEC12B/CR2/PRG2/FOXJ1/PGLYRP2 | 10 |
| GO:0006805 | BP | GO:0006805 | xenobiotic metabolic process | 10/695 | 125/18870 | 0.017216674 | 0.140751697 | 0.119921808 | GSTM3/CYP2J2/NOS1/SLC28A2/UGT1A3/CYP4F2/CYP2B6/SLC22A7/UGT1A10/SULT2A1 | 10 |
| GO:1904019 | BP | GO:1904019 | epithelial cell apoptotic process | 11/695 | 144/18870 | 0.017611439 | 0.143721923 | 0.122452469 | ANGPTL4/PLA2R1/FASLG/HLA-G/SCG2/GATA3/FGG/TERT/NKX2-5/FGA/FGB | 11 |
| GO:0008328 | CC | GO:0008328 | ionotropic glutamate receptor complex | 5/743 | 41/19886 | 0.017709519 | 0.132821395 | 0.116199302 | GRIK3/OLFM3/GRIA4/GRIK5/CACNG2 | 5 |
| GO:0045665 | BP | GO:0045665 | negative regulation of neuron differentiation | 7/695 | 73/18870 | 0.017781555 | 0.144851526 | 0.123414901 | DLX1/TP73/HES5/DLX2/NR2E1/ISL2/BMP7 | 7 |
| GO:0006584 | BP | GO:0006584 | catecholamine metabolic process | 6/695 | 57/18870 | 0.018030725 | 0.145242291 | 0.123747837 | SLC6A3/ABAT/GATA3/DAO/TACR3/SNCB | 6 |
| GO:0009712 | BP | GO:0009712 | catechol-containing compound metabolic process | 6/695 | 57/18870 | 0.018030725 | 0.145242291 | 0.123747837 | SLC6A3/ABAT/GATA3/DAO/TACR3/SNCB | 6 |
| GO:0010181 | MF | GO:0010181 | FMN binding | 3/709 | 15/18496 | 0.018080521 | 0.135335385 | 0.115323386 | NOS1/IYD/HAO1 | 3 |
| GO:0002688 | BP | GO:0002688 | regulation of leukocyte chemotaxis | 10/695 | 126/18870 | 0.01810734 | 0.145242291 | 0.123747837 | VEGFA/TNFAIP6/PGF/PLA2G7/CCL5/TNFSF14/CXCL10/KLRK1/CXCL13/LBP | 10 |
| GO:0042471 | BP | GO:0042471 | ear morphogenesis | 10/695 | 126/18870 | 0.01810734 | 0.145242291 | 0.123747837 | DLX5/MYO3A/PROX1/GATA3/CTHRC1/MYO3B/GBX2/FGF10/LHFPL5/HMX2 | 10 |
| GO:0019827 | BP | GO:0019827 | stem cell population maintenance | 13/695 | 183/18870 | 0.018161475 | 0.145242291 | 0.123747837 | ESRRB/POU5F1/SFRP1/WNT9B/PROX1/EOMES/HES5/NR2E1/FGF10/TUBA3C/BMP7/ACTL6B/NANOS2 | 13 |
| GO:0009306 | BP | GO:0009306 | protein secretion | 22/695 | 368/18870 | 0.018172103 | 0.145242291 | 0.123747837 | TREM2/INHBB/SFRP1/PTPRN/ABAT/CCL5/CASR/IFNG/CTAGE9/SCG2/TFAP2B/FAM3B/F2/FGG/SYT7/SSTR5/SAA1/SLC30A8/ADCY8/FGA/FGB/KRT20 | 22 |
| GO:0003279 | BP | GO:0003279 | cardiac septum development | 9/695 | 108/18870 | 0.018249518 | 0.145242291 | 0.123747837 | SOX11/DNAH11/PROX1/STRA6/GATA3/TBX5/NKX2-5/BMP7/GATA4 | 9 |
| GO:0001819 | BP | GO:0001819 | positive regulation of cytokine production | 28/695 | 499/18870 | 0.018400079 | 0.145242291 | 0.123747837 | HILPDA/TREM2/IDO1/FCGR3A/PLA2R1/IL20RB/C3/ALOX15B/GBP5/HLA-G/CD2/BATF/CRTAM/IFNG/KLRK1/AIM2/GATA3/HHLA2/PAEP/PRG2/SAA1/VTCN1/ORM2/KLRF2/LBP/ORM1/CEACAM20/GATA4 | 28 |
| GO:0002263 | BP | GO:0002263 | cell activation involved in immune response | 19/695 | 305/18870 | 0.018403943 | 0.145242291 | 0.123747837 | TREM2/ENPP3/FCGR3A/NKG7/EOMES/AICDA/ZNF683/BATF/LAT/IFNG/RAG2/GATA3/SCNN1B/PGLYRP2/KLRF2/LBP/IFNA14/CPLX2/NKX2-3 | 19 |
| GO:0009268 | BP | GO:0009268 | response to pH | 5/695 | 42/18870 | 0.018441472 | 0.145242291 | 0.123747837 | PKD2L1/SCNN1B/INSRR/SCNN1G/SST | 5 |
| GO:0032941 | BP | GO:0032941 | secretion by tissue | 5/695 | 42/18870 | 0.018441472 | 0.145242291 | 0.123747837 | WNK4/CEL/SCNN1B/FGF10/SLC4A9 | 5 |
| GO:0045124 | BP | GO:0045124 | regulation of bone resorption | 5/695 | 42/18870 | 0.018441472 | 0.145242291 | 0.123747837 | HAMP/DCSTAMP/TF/FSHB/CALCA | 5 |
| GO:0071542 | BP | GO:0071542 | dopaminergic neuron differentiation | 5/695 | 42/18870 | 0.018441472 | 0.145242291 | 0.123747837 | VEGFA/SFRP1/WNT9B/EN1/LMX1B | 5 |
| GO:2000403 | BP | GO:2000403 | positive regulation of lymphocyte migration | 5/695 | 42/18870 | 0.018441472 | 0.145242291 | 0.123747837 | CCL5/TNFSF14/CXCL10/CXCL13/CCL20 | 5 |
| GO:0042552 | BP | GO:0042552 | myelination | 11/695 | 145/18870 | 0.018447879 | 0.145242291 | 0.123747837 | KCNJ10/ITGAX/LGI4/CST7/ZNF488/HES5/KLK6/MAL/CNTN1/SCN2A/NKX6-2 | 11 |
| GO:0006829 | BP | GO:0006829 | zinc ion transport | 4/695 | 28/18870 | 0.018496939 | 0.145242291 | 0.123747837 | SLC30A2/MT3/SLC30A8/SLC30A10 | 4 |
| GO:0032703 | BP | GO:0032703 | negative regulation of interleukin-2 production | 4/695 | 28/18870 | 0.018496939 | 0.145242291 | 0.123747837 | IL20RB/LILRB4/LAG3/GATA3 | 4 |
| GO:0045606 | BP | GO:0045606 | positive regulation of epidermal cell differentiation | 4/695 | 28/18870 | 0.018496939 | 0.145242291 | 0.123747837 | ALOX15B/CYP27B1/SULT2B1/OVOL2 | 4 |
| GO:0048245 | BP | GO:0048245 | eosinophil chemotaxis | 4/695 | 28/18870 | 0.018496939 | 0.145242291 | 0.123747837 | CCL18/CCL5/SCG2/CCL25 | 4 |
| GO:0051873 | BP | GO:0051873 | killing by host of symbiont cells | 4/695 | 28/18870 | 0.018496939 | 0.145242291 | 0.123747837 | HRG/F2/SCNN1B/MBL2 | 4 |
| GO:2000108 | BP | GO:2000108 | positive regulation of leukocyte apoptotic process | 4/695 | 28/18870 | 0.018496939 | 0.145242291 | 0.123747837 | CDKN2A/IDO1/CCL5/PDCD1 | 4 |
| GO:0008066 | MF | GO:0008066 | glutamate receptor activity | 4/709 | 27/18496 | 0.018629927 | 0.138080632 | 0.117662694 | GRIK3/GRM1/GRIA4/GRIK5 | 4 |
| GO:0003007 | BP | GO:0003007 | heart morphogenesis | 17/695 | 264/18870 | 0.018676425 | 0.145936369 | 0.124339198 | VEGFA/SOX11/DNAH11/PROX1/TNNT2/TNNI1/GATA3/TNNC1/TBX5/MYH7/GRHL2/OVOL2/NPY2R/POU4F1/NKX2-5/BMP7/GATA4 | 17 |
| GO:0014706 | BP | GO:0014706 | striated muscle tissue development | 17/695 | 264/18870 | 0.018676425 | 0.145936369 | 0.124339198 | VEGFA/MYLK3/PROX1/LOX/MSC/TNNT2/TP73/TNNI1/TNNC1/TBX5/ERBB4/MYH7/SGCZ/POU4F1/NKX2-5/BMP7/GATA4 | 17 |
| GO:0002237 | BP | GO:0002237 | response to molecule of bacterial origin | 22/695 | 369/18870 | 0.018681133 | 0.145936369 | 0.124339198 | SCARB1/TREM2/IDO1/CD68/NOS1/FASLG/GBP5/AICDA/CXCL9/CXCL10/CYP27B1/CXCL11/UMOD/TNIP3/KLRK1/MMP9/PTGER1/CXCL5/CXCL13/EPO/FGF10/LBP | 22 |
| GO:0034774 | CC | GO:0034774 | secretory granule lumen | 20/743 | 322/19886 | 0.018794442 | 0.138647522 | 0.121296312 | VEGFA/EGF/VWF/RNASET2/C3/HRG/KNG1/CTSW/ALB/CHIT1/HP/FGG/OLFM4/TF/FOLR3/ORM2/FGA/ORM1/PLG/FGB | 20 |
| GO:0048771 | BP | GO:0048771 | tissue remodeling | 13/695 | 184/18870 | 0.018910087 | 0.146097551 | 0.124476527 | SFRP1/HRG/PTH1R/UMOD/HAMP/CTHRC1/DCSTAMP/SYT7/TF/FGF10/PLG/FSHB/CALCA | 13 |
| GO:0008202 | BP | GO:0008202 | steroid metabolic process | 20/695 | 327/18870 | 0.018957133 | 0.146097551 | 0.124476527 | SCARB1/APOC1/FGF1/PROX1/CYP27B1/IFNG/HSD17B3/UGT1A3/APOB/SULT2B1/SCNN1B/CYP2B6/BAAT/UGT2A1/SERPINA12/RDH8/UGT1A4/FSHB/SULT2A1/APOA4 | 20 |
| GO:0003281 | BP | GO:0003281 | ventricular septum development | 7/695 | 74/18870 | 0.01903695 | 0.146097551 | 0.124476527 | SOX11/PROX1/STRA6/GATA3/TBX5/NKX2-5/GATA4 | 7 |
| GO:0060415 | BP | GO:0060415 | muscle tissue morphogenesis | 7/695 | 74/18870 | 0.01903695 | 0.146097551 | 0.124476527 | PROX1/TNNT2/TNNI1/TNNC1/MYH7/POU4F1/NKX2-5 | 7 |
| GO:0007596 | BP | GO:0007596 | blood coagulation | 15/695 | 224/18870 | 0.019121502 | 0.146097551 | 0.124476527 | VWF/HRG/KNG1/TREML1/F2/PROZ/F11/FGG/CYP4F2/TSPAN8/SAA1/FGA/PLG/SLC4A1/FGB | 15 |
| GO:0050796 | BP | GO:0050796 | regulation of insulin secretion | 12/695 | 165/18870 | 0.019238094 | 0.146097551 | 0.124476527 | INHBB/SFRP1/ABAT/CCL5/CASR/IFNG/TFAP2B/F2/SYT7/SSTR5/SLC30A8/ADCY8 | 12 |
| GO:0090277 | BP | GO:0090277 | positive regulation of peptide hormone secretion | 9/695 | 109/18870 | 0.019266666 | 0.146097551 | 0.124476527 | ABAT/CASR/TFR2/F2/FGG/SLC30A8/ADCY8/FGA/FGB | 9 |
| GO:0055123 | BP | GO:0055123 | digestive system development | 11/695 | 146/18870 | 0.01931346 | 0.146097551 | 0.124476527 | SOX11/SFRP1/TCF21/STRA6/HOXD13/SPDEF/FGF10/OVOL2/BARX1/GATA4/NKX2-3 | 11 |
| GO:0002713 | BP | GO:0002713 | negative regulation of B cell mediated immunity | 3/695 | 16/18870 | 0.019469017 | 0.146097551 | 0.124476527 | SUSD4/CR2/FOXJ1 | 3 |
| GO:0002890 | BP | GO:0002890 | negative regulation of immunoglobulin mediated immune response | 3/695 | 16/18870 | 0.019469017 | 0.146097551 | 0.124476527 | SUSD4/CR2/FOXJ1 | 3 |
| GO:0010273 | BP | GO:0010273 | detoxification of copper ion | 3/695 | 16/18870 | 0.019469017 | 0.146097551 | 0.124476527 | MT1G/MT1H/MT3 | 3 |
| GO:0021534 | BP | GO:0021534 | cell proliferation in hindbrain | 3/695 | 16/18870 | 0.019469017 | 0.146097551 | 0.124476527 | EGF/GBX2/LHX1 | 3 |
| GO:0021924 | BP | GO:0021924 | cell proliferation in external granule layer | 3/695 | 16/18870 | 0.019469017 | 0.146097551 | 0.124476527 | EGF/GBX2/LHX1 | 3 |
| GO:0021930 | BP | GO:0021930 | cerebellar granule cell precursor proliferation | 3/695 | 16/18870 | 0.019469017 | 0.146097551 | 0.124476527 | EGF/GBX2/LHX1 | 3 |
| GO:0033275 | BP | GO:0033275 | actin-myosin filament sliding | 3/695 | 16/18870 | 0.019469017 | 0.146097551 | 0.124476527 | TNNT2/TNNC1/MYH7 | 3 |
| GO:0034375 | BP | GO:0034375 | high-density lipoprotein particle remodeling | 3/695 | 16/18870 | 0.019469017 | 0.146097551 | 0.124476527 | SCARB1/APOC1/APOA4 | 3 |
| GO:0034638 | BP | GO:0034638 | phosphatidylcholine catabolic process | 3/695 | 16/18870 | 0.019469017 | 0.146097551 | 0.124476527 | SCARB1/APOC1/PLA2G7 | 3 |
| GO:0043584 | BP | GO:0043584 | nose development | 3/695 | 16/18870 | 0.019469017 | 0.146097551 | 0.124476527 | DLX5/PROX1/STRA6 | 3 |
| GO:0051004 | BP | GO:0051004 | regulation of lipoprotein lipase activity | 3/695 | 16/18870 | 0.019469017 | 0.146097551 | 0.124476527 | ANGPTL4/APOC1/APOA4 | 3 |
| GO:0060841 | BP | GO:0060841 | venous blood vessel development | 3/695 | 16/18870 | 0.019469017 | 0.146097551 | 0.124476527 | VEGFA/PROX1/NKX2-5 | 3 |
| GO:0070230 | BP | GO:0070230 | positive regulation of lymphocyte apoptotic process | 3/695 | 16/18870 | 0.019469017 | 0.146097551 | 0.124476527 | IDO1/CCL5/PDCD1 | 3 |
| GO:0072378 | BP | GO:0072378 | blood coagulation, fibrin clot formation | 3/695 | 16/18870 | 0.019469017 | 0.146097551 | 0.124476527 | FGG/FGA/FGB | 3 |
| GO:1901741 | BP | GO:1901741 | positive regulation of myoblast fusion | 3/695 | 16/18870 | 0.019469017 | 0.146097551 | 0.124476527 | TNFSF14/CXCL9/MYOD1 | 3 |
| GO:1990169 | BP | GO:1990169 | stress response to copper ion | 3/695 | 16/18870 | 0.019469017 | 0.146097551 | 0.124476527 | MT1G/MT1H/MT3 | 3 |
| GO:0045766 | BP | GO:0045766 | positive regulation of angiogenesis | 13/695 | 185/18870 | 0.019681699 | 0.147210086 | 0.125424418 | ANGPTL4/VEGFA/HK2/FGF1/ITGAX/PGF/ANGPT2/PIK3R6/C3/TERT/NR2E1/APELA/GATA4 | 13 |
| GO:1904018 | BP | GO:1904018 | positive regulation of vasculature development | 13/695 | 185/18870 | 0.019681699 | 0.147210086 | 0.125424418 | ANGPTL4/VEGFA/HK2/FGF1/ITGAX/PGF/ANGPT2/PIK3R6/C3/TERT/NR2E1/APELA/GATA4 | 13 |
| GO:0022408 | BP | GO:0022408 | negative regulation of cell-cell adhesion | 14/695 | 205/18870 | 0.019855051 | 0.148264027 | 0.126322385 | VEGFA/IDO1/IL20RB/LILRB4/HLA-G/CRTAM/LAG3/RAG2/PLA2G2D/B4GALNT2/FOXJ1/VTCN1/CCL25/PLG | 14 |
| GO:0048706 | BP | GO:0048706 | embryonic skeletal system development | 10/695 | 128/18870 | 0.019989726 | 0.149026179 | 0.126971746 | SOX11/WNT9B/DLX1/TBX15/DLX2/HOXB9/ALX1/GRHL2/LHX1/BMP7 | 10 |
| GO:0007519 | BP | GO:0007519 | skeletal muscle tissue development | 12/695 | 166/18870 | 0.020070459 | 0.149384362 | 0.127276921 | SOX11/TCF21/NPHS1/EOMES/MYOZ2/CHRNA1/MSC/P2RX2/CHRND/ANKRD33/PITX1/MYOD1 | 12 |
| GO:0007272 | BP | GO:0007272 | ensheathment of neurons | 11/695 | 147/18870 | 0.020208741 | 0.14992524 | 0.127737755 | KCNJ10/ITGAX/LGI4/CST7/ZNF488/HES5/KLK6/MAL/CNTN1/SCN2A/NKX6-2 | 11 |
| GO:0008366 | BP | GO:0008366 | axon ensheathment | 11/695 | 147/18870 | 0.020208741 | 0.14992524 | 0.127737755 | KCNJ10/ITGAX/LGI4/CST7/ZNF488/HES5/KLK6/MAL/CNTN1/SCN2A/NKX6-2 | 11 |
| GO:0019098 | BP | GO:0019098 | reproductive behavior | 5/695 | 43/18870 | 0.020254618 | 0.150022052 | 0.127820239 | ABAT/EDDM3A/SEMG1/PPP1R1B/MTNR1A | 5 |
| GO:0001708 | BP | GO:0001708 | cell fate specification | 9/695 | 110/18870 | 0.020324241 | 0.150051344 | 0.127845197 | POU5F1/EOMES/TBX15/TBX5/ISL2/POU4F1/DMRT3/ESRP1/SOX1 | 9 |
| GO:0045445 | BP | GO:0045445 | myoblast differentiation | 9/695 | 110/18870 | 0.020324241 | 0.150051344 | 0.127845197 | IGFBP3/RANBP3L/TNFSF14/CXCL9/CXCL10/ANKRD2/PITX1/MYOD1/ACTL6B | 9 |
| GO:0006936 | BP | GO:0006936 | muscle contraction | 21/695 | 351/18870 | 0.020513966 | 0.15109022 | 0.128730329 | NOS1/ABAT/CHRNA1/TNNT2/TNNI1/TNNC1/MYBPH/TNNT1/P2RX2/SCN7A/MYH7/SCNN1B/CHRND/NMUR2/MYH13/ANKRD2/TACR3/SCN10A/NKX2-5/GATA4/CALCA | 21 |
| GO:0051251 | BP | GO:0051251 | positive regulation of lymphocyte activation | 20/695 | 330/18870 | 0.020682448 | 0.15109022 | 0.128730329 | CD70/TNFRSF4/FCGR3A/PIK3R6/LILRB4/TNFSF9/CCL5/HLA-G/TNFSF14/ICOS/SIRPG/IFNG/CD27/KLRK1/GATA3/HHLA2/EPO/VTCN1/FGF10/ACTL6B | 20 |
| GO:0001773 | BP | GO:0001773 | myeloid dendritic cell activation | 4/695 | 29/18870 | 0.020847546 | 0.15109022 | 0.128730329 | CD2/BATF/UBD/DCSTAMP | 4 |
| GO:0003209 | BP | GO:0003209 | cardiac atrium morphogenesis | 4/695 | 29/18870 | 0.020847546 | 0.15109022 | 0.128730329 | PROX1/TBX5/NKX2-5/GATA4 | 4 |
| GO:0003401 | BP | GO:0003401 | axis elongation | 4/695 | 29/18870 | 0.020847546 | 0.15109022 | 0.128730329 | FGF1/SFRP1/HOXD13/FGF10 | 4 |
| GO:0010447 | BP | GO:0010447 | response to acidic pH | 4/695 | 29/18870 | 0.020847546 | 0.15109022 | 0.128730329 | PKD2L1/SCNN1B/SCNN1G/SST | 4 |
| GO:0032770 | BP | GO:0032770 | positive regulation of monooxygenase activity | 4/695 | 29/18870 | 0.020847546 | 0.15109022 | 0.128730329 | SCARB1/CYP27B1/IFNG/TERT | 4 |
| GO:0042403 | BP | GO:0042403 | thyroid hormone metabolic process | 4/695 | 29/18870 | 0.020847546 | 0.15109022 | 0.128730329 | IYD/DIO1/GATA3/SULT2A1 | 4 |
| GO:0051354 | BP | GO:0051354 | negative regulation of oxidoreductase activity | 4/695 | 29/18870 | 0.020847546 | 0.15109022 | 0.128730329 | GZMA/CYP27B1/HP/MT3 | 4 |
| GO:0060295 | BP | GO:0060295 | regulation of cilium movement involved in cell motility | 4/695 | 29/18870 | 0.020847546 | 0.15109022 | 0.128730329 | TPPP2/SEMG2/SEMG1/TACR3 | 4 |
| GO:1902019 | BP | GO:1902019 | regulation of cilium-dependent cell motility | 4/695 | 29/18870 | 0.020847546 | 0.15109022 | 0.128730329 | TPPP2/SEMG2/SEMG1/TACR3 | 4 |
| GO:0030509 | BP | GO:0030509 | BMP signaling pathway | 12/695 | 167/18870 | 0.020929344 | 0.15109022 | 0.128730329 | TNFAIP6/SOX11/DLX5/SFRP1/SOST/DLX1/GDF6/GPC3/TFAP2B/HES5/BMP7/GATA4 | 12 |
| GO:0007127 | BP | GO:0007127 | meiosis I | 10/695 | 129/18870 | 0.020982787 | 0.15109022 | 0.128730329 | HSPA2/RNF212B/TEX15/TEX11/MSH4/FMN2/HORMAD1/DNMT3L/DMRT1/DMRTC2 | 10 |
| GO:0048738 | BP | GO:0048738 | cardiac muscle tissue development | 16/695 | 247/18870 | 0.020995015 | 0.15109022 | 0.128730329 | VEGFA/MYLK3/PROX1/MSC/TNNT2/TP73/TNNI1/TNNC1/TBX5/ERBB4/MYH7/SGCZ/POU4F1/NKX2-5/BMP7/GATA4 | 16 |
| GO:0002823 | BP | GO:0002823 | negative regulation of adaptive immune response based on somatic recombination of immune receptors built from immunoglobulin superfamily domains | 6/695 | 59/18870 | 0.021060059 | 0.15109022 | 0.128730329 | IL20RB/LILRB4/HLA-G/SUSD4/CR2/FOXJ1 | 6 |
| GO:0006826 | BP | GO:0006826 | iron ion transport | 6/695 | 59/18870 | 0.021060059 | 0.15109022 | 0.128730329 | HRG/CP/IFNG/TFR2/HAMP/TF | 6 |
| GO:0046686 | BP | GO:0046686 | response to cadmium ion | 6/695 | 59/18870 | 0.021060059 | 0.15109022 | 0.128730329 | MMP9/MT1G/TERT/SLC34A1/MT1H/MT3 | 6 |
| GO:0050881 | BP | GO:0050881 | musculoskeletal movement | 6/695 | 59/18870 | 0.021060059 | 0.15109022 | 0.128730329 | CHRNA1/TNNI1/TNNC1/TNNT1/MYH7/CHRND | 6 |
| GO:0098727 | BP | GO:0098727 | maintenance of cell number | 13/695 | 187/18870 | 0.02129554 | 0.152478557 | 0.129913205 | ESRRB/POU5F1/SFRP1/WNT9B/PROX1/EOMES/HES5/NR2E1/FGF10/TUBA3C/BMP7/ACTL6B/NANOS2 | 13 |
| GO:0043279 | BP | GO:0043279 | response to alkaloid | 8/695 | 93/18870 | 0.021320306 | 0.152478557 | 0.129913205 | SLC6A3/ABAT/CRHBP/EN1/SLC34A1/PPP1R1B/TACR3/ADCY8 | 8 |
| GO:0002793 | BP | GO:0002793 | positive regulation of peptide secretion | 9/695 | 111/18870 | 0.021423058 | 0.152497469 | 0.129929319 | ABAT/CASR/TFR2/F2/FGG/SLC30A8/ADCY8/FGA/FGB | 9 |
| GO:0071347 | BP | GO:0071347 | cellular response to interleukin-1 | 9/695 | 111/18870 | 0.021423058 | 0.152497469 | 0.129929319 | MYLK3/INHBB/SFRP1/CCL18/CCL5/FGG/CCL20/CCL25/FGB | 9 |
| GO:1902106 | BP | GO:1902106 | negative regulation of leukocyte differentiation | 9/695 | 111/18870 | 0.021423058 | 0.152497469 | 0.129929319 | TNFAIP6/LILRB4/SFRP1/TMEM178A/HLA-G/LAG3/RAG2/FOXJ1/PGLYRP2 | 9 |
| GO:0043028 | MF | GO:0043028 | cysteine-type endopeptidase regulator activity involved in apoptotic process | 5/709 | 42/18496 | 0.021516799 | 0.152832394 | 0.130233117 | NOL3/BIRC7/TNFSF14/CD27/DPEP1 | 5 |
| GO:0008392 | MF | GO:0008392 | arachidonic acid epoxygenase activity | 3/709 | 16/18496 | 0.02163104 | 0.152832394 | 0.130233117 | CYP2J2/CYP4F2/CYP2B6 | 3 |
| GO:0015378 | MF | GO:0015378 | sodium:chloride symporter activity | 3/709 | 16/18496 | 0.02163104 | 0.152832394 | 0.130233117 | SLC6A3/SLC12A1/SLC12A3 | 3 |
| GO:0015385 | MF | GO:0015385 | sodium:proton antiporter activity | 3/709 | 16/18496 | 0.02163104 | 0.152832394 | 0.130233117 | SLC9A3/SLC9A4/SLC9A2 | 3 |
| GO:0022841 | MF | GO:0022841 | potassium ion leak channel activity | 3/709 | 16/18496 | 0.02163104 | 0.152832394 | 0.130233117 | KCNK9/KCNK10/KCNK17 | 3 |
| GO:0050731 | BP | GO:0050731 | positive regulation of peptidyl-tyrosine phosphorylation | 12/695 | 168/18870 | 0.021815214 | 0.15504748 | 0.132101953 | TREM2/VEGFA/EGF/FCGR1A/CCL5/EPGN/IFNG/HES5/EPO/ERBB4/IL11/FGF10 | 12 |
| GO:0044282 | BP | GO:0044282 | small molecule catabolic process | 22/695 | 375/18870 | 0.021973433 | 0.155687731 | 0.132647453 | SCARB1/SLC16A3/HK2/ENO2/SDS/IDO1/ALDH6A1/NOS1/ABAT/UPP2/AICDA/CYP27B1/HPD/AGXT/DPEP1/ALDOB/CYP4F2/FABP1/DAO/ALDH3B2/HAO1/SULT2A1 | 22 |
| GO:0090132 | BP | GO:0090132 | epithelium migration | 22/695 | 375/18870 | 0.021973433 | 0.155687731 | 0.132647453 | SCARB1/VEGFA/FGF1/EGF/ANGPT2/PROX1/HRG/IFNG/SCG2/S100A2/MMP9/GATA3/CXCL13/TACSTD2/EPB41L4B/NR2E1/GRHL2/FGF10/RAB25/APELA/PLG/FGFBP1 | 22 |
| GO:0046006 | BP | GO:0046006 | regulation of activated T cell proliferation | 5/695 | 44/18870 | 0.022180462 | 0.156911315 | 0.133689959 | LILRB4/TNFSF9/CRTAM/HHLA2/EPO | 5 |
| GO:0005244 | MF | GO:0005244 | voltage-gated monoatomic ion channel activity | 14/709 | 200/18496 | 0.022233143 | 0.1531321 | 0.130488506 | KCNJ10/CLIC5/KCNJ1/KCNK9/KCNN1/KCNK10/CLCNKA/SCN7A/KCNK17/SCN2A/SCN10A/CLCNKB/CACNG2/KCNU1 | 14 |
| GO:0022832 | MF | GO:0022832 | voltage-gated channel activity | 14/709 | 200/18496 | 0.022233143 | 0.1531321 | 0.130488506 | KCNJ10/CLIC5/KCNJ1/KCNK9/KCNN1/KCNK10/CLCNKA/SCN7A/KCNK17/SCN2A/SCN10A/CLCNKB/CACNG2/KCNU1 | 14 |
| GO:0008083 | MF | GO:0008083 | growth factor activity | 12/709 | 162/18496 | 0.022281126 | 0.1531321 | 0.130488506 | VEGFA/INHBB/FGF1/PGF/EGF/GDF6/EPGN/F2/INHBE/IL11/FGF10/BMP7 | 12 |
| GO:0007566 | BP | GO:0007566 | embryo implantation | 6/695 | 60/18870 | 0.022696468 | 0.158345169 | 0.134911616 | STC2/VEGFA/MMP9/EPO/APELA/CALCA | 6 |
| GO:0045620 | BP | GO:0045620 | negative regulation of lymphocyte differentiation | 6/695 | 60/18870 | 0.022696468 | 0.158345169 | 0.134911616 | LILRB4/SFRP1/LAG3/RAG2/FOXJ1/PGLYRP2 | 6 |
| GO:0050879 | BP | GO:0050879 | multicellular organismal movement | 6/695 | 60/18870 | 0.022696468 | 0.158345169 | 0.134911616 | CHRNA1/TNNI1/TNNC1/TNNT1/MYH7/CHRND | 6 |
| GO:0070228 | BP | GO:0070228 | regulation of lymphocyte apoptotic process | 6/695 | 60/18870 | 0.022696468 | 0.158345169 | 0.134911616 | IDO1/BIRC7/CCL5/AURKB/CD27/PDCD1 | 6 |
| GO:0002921 | BP | GO:0002921 | negative regulation of humoral immune response | 3/695 | 17/18870 | 0.023006825 | 0.158345169 | 0.134911616 | SUSD4/CR2/FOXJ1 | 3 |
| GO:0003159 | BP | GO:0003159 | morphogenesis of an endothelium | 3/695 | 17/18870 | 0.023006825 | 0.158345169 | 0.134911616 | FGF1/ITGAX/CXCL10 | 3 |
| GO:0006544 | BP | GO:0006544 | glycine metabolic process | 3/695 | 17/18870 | 0.023006825 | 0.158345169 | 0.134911616 | AGXT/BAAT/HAO1 | 3 |
| GO:0007379 | BP | GO:0007379 | segment specification | 3/695 | 17/18870 | 0.023006825 | 0.158345169 | 0.134911616 | IRX2/IRX1/HES5 | 3 |
| GO:0008228 | BP | GO:0008228 | opsonization | 3/695 | 17/18870 | 0.023006825 | 0.158345169 | 0.134911616 | CRP/LBP/MBL2 | 3 |
| GO:0030322 | BP | GO:0030322 | stabilization of membrane potential | 3/695 | 17/18870 | 0.023006825 | 0.158345169 | 0.134911616 | KCNK9/KCNK10/KCNK17 | 3 |
| GO:0031643 | BP | GO:0031643 | positive regulation of myelination | 3/695 | 17/18870 | 0.023006825 | 0.158345169 | 0.134911616 | ITGAX/CST7/ZNF488 | 3 |
| GO:0044320 | BP | GO:0044320 | cellular response to leptin stimulus | 3/695 | 17/18870 | 0.023006825 | 0.158345169 | 0.134911616 | INHBB/MT3/FGB | 3 |
| GO:0051447 | BP | GO:0051447 | negative regulation of meiotic cell cycle | 3/695 | 17/18870 | 0.023006825 | 0.158345169 | 0.134911616 | HORMAD1/DMRT1/NANOS2 | 3 |
| GO:0060192 | BP | GO:0060192 | negative regulation of lipase activity | 3/695 | 17/18870 | 0.023006825 | 0.158345169 | 0.134911616 | ANGPTL4/APOC1/PLA2R1 | 3 |
| GO:0061154 | BP | GO:0061154 | endothelial tube morphogenesis | 3/695 | 17/18870 | 0.023006825 | 0.158345169 | 0.134911616 | FGF1/ITGAX/CXCL10 | 3 |
| GO:0071468 | BP | GO:0071468 | cellular response to acidic pH | 3/695 | 17/18870 | 0.023006825 | 0.158345169 | 0.134911616 | PKD2L1/SCNN1B/SCNN1G | 3 |
| GO:0072224 | BP | GO:0072224 | metanephric glomerulus development | 3/695 | 17/18870 | 0.023006825 | 0.158345169 | 0.134911616 | TCF21/NPHS2/LHX1 | 3 |
| GO:0090399 | BP | GO:0090399 | replicative senescence | 3/695 | 17/18870 | 0.023006825 | 0.158345169 | 0.134911616 | CDKN2A/PLA2R1/TERT | 3 |
| GO:1902652 | BP | GO:1902652 | secondary alcohol metabolic process | 11/695 | 150/18870 | 0.023078303 | 0.158598262 | 0.135127254 | SCARB1/APOC1/FGF1/CYP27B1/UGT1A3/APOB/SULT2B1/SERPINA12/UGT1A4/SULT2A1/APOA4 | 11 |
| GO:0048732 | BP | GO:0048732 | gland development | 25/695 | 443/18870 | 0.023441669 | 0.160853494 | 0.137048733 | VEGFA/HK2/SLC6A3/INHBB/FGF1/EGF/SFRP1/TCF21/PROX1/ALOX15B/STRA6/GATA3/HOXB9/HOXD13/ERBB4/BAAT/WNT7B/FGF10/TFCP2L1/PITX1/UGT1A10/HOXB13/NKX2-5/BMP7/NKX2-3 | 25 |
| GO:0031589 | BP | GO:0031589 | cell-substrate adhesion | 21/695 | 356/18870 | 0.02352846 | 0.161206992 | 0.137349917 | CDKN2A/VEGFA/COL5A3/ITGAX/ITGAD/ANGPT2/VWF/SFRP1/HRG/PTPRO/COL26A1/FGG/TACSTD2/FREM1/OLFM4/DMP1/L1CAM/CCL25/FGA/PLG/FGB | 21 |
| GO:0015144 | MF | GO:0015144 | carbohydrate transmembrane transporter activity | 5/709 | 43/18496 | 0.023604355 | 0.160285865 | 0.136584446 | SLC5A2/AQP2/SLC2A12/SLC5A11/AQP9 | 5 |
| GO:0001968 | MF | GO:0001968 | fibronectin binding | 4/709 | 29/18496 | 0.023746054 | 0.160285865 | 0.136584446 | VEGFA/TNFAIP6/IGFBP3/LILRB4 | 4 |
| GO:0032355 | BP | GO:0032355 | response to estradiol | 9/695 | 113/18870 | 0.023747601 | 0.162464873 | 0.138421644 | WNT8B/SFRP1/CRHBP/SLC34A1/TACR3/FGF10/POU4F1/BMP7/MYOD1 | 9 |
| GO:0001674 | CC | GO:0001674 | female germ cell nucleus | 3/743 | 17/19886 | 0.023888896 | 0.173387146 | 0.151688403 | KPNA7/LHX8/DMRT1 | 3 |
| GO:1901617 | BP | GO:1901617 | organic hydroxy compound biosynthetic process | 16/695 | 251/18870 | 0.023959811 | 0.163671654 | 0.139449832 | SLC6A3/FGF1/PROX1/ALOX15B/AVPR1B/CYP27B1/PTH1R/IFNG/ITPKA/GATA3/TYRP1/HSD17B3/SPTSSB/APOB/DAO/BAAT | 16 |
| GO:0090287 | BP | GO:0090287 | regulation of cellular response to growth factor stimulus | 21/695 | 357/18870 | 0.024170205 | 0.16486244 | 0.140464394 | VEGFA/TNFAIP6/SOX11/FGF1/HTRA4/SFRP1/HRG/SOST/LOX/PRDM16/DLX1/RASL11B/GPC3/TFAP2B/HES5/GATA3/CXCL13/MT3/FGF10/GATA4/FGFBP1 | 21 |
| GO:0048701 | BP | GO:0048701 | embryonic cranial skeleton morphogenesis | 5/695 | 45/18870 | 0.024221246 | 0.164964372 | 0.140551241 | WNT9B/TBX15/DLX2/GRHL2/LHX1 | 5 |
| GO:0050730 | BP | GO:0050730 | regulation of peptidyl-tyrosine phosphorylation | 15/695 | 231/18870 | 0.024416589 | 0.166047342 | 0.141473942 | TREM2/VEGFA/EGF/LILRB4/SFRP1/FCGR1A/HRG/CCL5/EPGN/IFNG/HES5/EPO/ERBB4/IL11/FGF10 | 15 |
| GO:0004857 | MF | GO:0004857 | enzyme inhibitor activity | 23/709 | 386/18496 | 0.024935885 | 0.166827691 | 0.142158934 | CDKN2A/ANGPTL4/APOC1/BIRC7/LILRB4/C3/HRG/GBP5/KNG1/CST7/TRIB3/SERPINA5/SPINK13/GPC3/CST9/ITIH1/PPP1R1B/LCN1/WFDC10B/SERPINA12/SNCB/SERPINA9/SLN | 23 |
| GO:0090130 | BP | GO:0090130 | tissue migration | 22/695 | 380/18870 | 0.025045725 | 0.170072756 | 0.144903633 | SCARB1/VEGFA/FGF1/EGF/ANGPT2/PROX1/HRG/IFNG/SCG2/S100A2/MMP9/GATA3/CXCL13/TACSTD2/EPB41L4B/NR2E1/GRHL2/FGF10/RAB25/APELA/PLG/FGFBP1 | 22 |
| GO:1903038 | BP | GO:1903038 | negative regulation of leukocyte cell-cell adhesion | 11/695 | 152/18870 | 0.025149852 | 0.170526447 | 0.145290182 | IDO1/IL20RB/LILRB4/HLA-G/CRTAM/LAG3/RAG2/PLA2G2D/FOXJ1/VTCN1/CCL25 | 11 |
| GO:0002690 | BP | GO:0002690 | positive regulation of leukocyte chemotaxis | 8/695 | 96/18870 | 0.025255931 | 0.170892905 | 0.145602408 | VEGFA/PGF/PLA2G7/CCL5/TNFSF14/CXCL10/CXCL13/LBP | 8 |
| GO:0048593 | BP | GO:0048593 | camera-type eye morphogenesis | 10/695 | 133/18870 | 0.025316082 | 0.170892905 | 0.145602408 | VEGFA/CALB1/SOX11/PROX1/TFAP2B/STRA6/VSX1/LHX1/BMP7/SOX1 | 10 |
| GO:0050868 | BP | GO:0050868 | negative regulation of T cell activation | 10/695 | 133/18870 | 0.025316082 | 0.170892905 | 0.145602408 | IDO1/IL20RB/LILRB4/HLA-G/CRTAM/LAG3/RAG2/PLA2G2D/FOXJ1/VTCN1 | 10 |
| GO:0030169 | MF | GO:0030169 | low-density lipoprotein particle binding | 3/709 | 17/18496 | 0.025533756 | 0.167856692 | 0.143035778 | SCARB1/TREM2/CRP | 3 |
| GO:0042165 | MF | GO:0042165 | neurotransmitter binding | 3/709 | 17/18496 | 0.025533756 | 0.167856692 | 0.143035778 | CHRNA1/CHRND/CHRNA4 | 3 |
| GO:0010632 | BP | GO:0010632 | regulation of epithelial cell migration | 18/695 | 295/18870 | 0.025621225 | 0.172697637 | 0.147140058 | SCARB1/VEGFA/FGF1/EGF/ANGPT2/PROX1/HRG/IFNG/MMP9/GATA3/CXCL13/TACSTD2/EPB41L4B/NR2E1/FGF10/RAB25/PLG/FGFBP1 | 18 |
| GO:0005516 | MF | GO:0005516 | calmodulin binding | 14/709 | 204/18496 | 0.025810871 | 0.168215677 | 0.14334168 | PNCK/TRPV6/MYO3A/NOS1/RGS1/KCNN1/TRPV5/ATP2B2/ITPKA/SYT7/MYH7/SCN2A/MYH13/ADCY8 | 14 |
| GO:0031646 | BP | GO:0031646 | positive regulation of nervous system process | 4/695 | 31/18870 | 0.026076597 | 0.174480302 | 0.148658905 | ITGAX/ABAT/CST7/ZNF488 | 4 |
| GO:0035767 | BP | GO:0035767 | endothelial cell chemotaxis | 4/695 | 31/18870 | 0.026076597 | 0.174480302 | 0.148658905 | VEGFA/FGF1/HRG/CXCL13 | 4 |
| GO:0048240 | BP | GO:0048240 | sperm capacitation | 4/695 | 31/18870 | 0.026076597 | 0.174480302 | 0.148658905 | SEMG2/PAEP/SEMG1/CATSPERD | 4 |
| GO:0050482 | BP | GO:0050482 | arachidonic acid secretion | 4/695 | 31/18870 | 0.026076597 | 0.174480302 | 0.148658905 | PLA2R1/AVPR1B/PLA2G2D/NMUR2 | 4 |
| GO:1903963 | BP | GO:1903963 | arachidonate transport | 4/695 | 31/18870 | 0.026076597 | 0.174480302 | 0.148658905 | PLA2R1/AVPR1B/PLA2G2D/NMUR2 | 4 |
| GO:0022617 | BP | GO:0022617 | extracellular matrix disassembly | 6/695 | 62/18870 | 0.026220963 | 0.174879727 | 0.14899922 | MMP9/KLK7/MMP13/PLG/KLK5/KLK4 | 6 |
| GO:0044344 | BP | GO:0044344 | cellular response to fibroblast growth factor stimulus | 9/695 | 115/18870 | 0.026246512 | 0.174879727 | 0.14899922 | FGF1/SFRP1/CCL5/FLRT1/GATA3/CXCL13/FGF10/LHX1/FGFBP1 | 9 |
| GO:0021536 | BP | GO:0021536 | diencephalon development | 7/695 | 79/18870 | 0.026251093 | 0.174879727 | 0.14899922 | SLC6A3/INHBB/GBX2/FGF10/PITX1/POU4F1/ARX | 7 |
| GO:0032309 | BP | GO:0032309 | icosanoid secretion | 5/695 | 46/18870 | 0.026379032 | 0.175221183 | 0.149290143 | PLA2R1/AVPR1B/CYP4F2/PLA2G2D/NMUR2 | 5 |
| GO:0097028 | BP | GO:0097028 | dendritic cell differentiation | 5/695 | 46/18870 | 0.026379032 | 0.175221183 | 0.149290143 | TREM2/HLA-G/BATF/UBD/DCSTAMP | 5 |
| GO:1903039 | BP | GO:1903039 | positive regulation of leukocyte cell-cell adhesion | 17/695 | 275/18870 | 0.026460274 | 0.175467086 | 0.149499655 | CD70/PIK3R6/LILRB4/TNFSF9/CCL5/HLA-G/TNFSF14/ICOS/SIRPG/IFNG/CD27/KLRK1/GATA3/HHLA2/EPO/VTCN1/ACTL6B | 17 |
| GO:0048565 | BP | GO:0048565 | digestive tract development | 10/695 | 134/18870 | 0.026492842 | 0.175467086 | 0.149499655 | SOX11/SFRP1/TCF21/STRA6/HOXD13/SPDEF/FGF10/OVOL2/GATA4/NKX2-3 | 10 |
| GO:0015106 | MF | GO:0015106 | bicarbonate transmembrane transporter activity | 4/709 | 30/18496 | 0.026592404 | 0.170371673 | 0.145178869 | SLC4A11/SLC26A4/SLC4A9/SLC4A1 | 4 |
| GO:0031210 | MF | GO:0031210 | phosphatidylcholine binding | 4/709 | 30/18496 | 0.026592404 | 0.170371673 | 0.145178869 | APOC1/SERPINA5/GPR12/APOA4 | 4 |
| GO:0010002 | BP | GO:0010002 | cardioblast differentiation | 3/695 | 18/18870 | 0.026869172 | 0.175933255 | 0.149896835 | EOMES/TBX5/NKX2-5 | 3 |
| GO:0010522 | BP | GO:0010522 | regulation of calcium ion transport into cytosol | 3/695 | 18/18870 | 0.026869172 | 0.175933255 | 0.149896835 | NOS1/P2RX2/EPO | 3 |
| GO:0019373 | BP | GO:0019373 | epoxygenase P450 pathway | 3/695 | 18/18870 | 0.026869172 | 0.175933255 | 0.149896835 | CYP2J2/CYP4F2/CYP2B6 | 3 |
| GO:0035864 | BP | GO:0035864 | response to potassium ion | 3/695 | 18/18870 | 0.026869172 | 0.175933255 | 0.149896835 | KCNJ10/CRHBP/SLC34A1 | 3 |
| GO:0045780 | BP | GO:0045780 | positive regulation of bone resorption | 3/695 | 18/18870 | 0.026869172 | 0.175933255 | 0.149896835 | DCSTAMP/TF/FSHB | 3 |
| GO:0046851 | BP | GO:0046851 | negative regulation of bone remodeling | 3/695 | 18/18870 | 0.026869172 | 0.175933255 | 0.149896835 | SFRP1/HAMP/CALCA | 3 |
| GO:1904044 | BP | GO:1904044 | response to aldosterone | 3/695 | 18/18870 | 0.026869172 | 0.175933255 | 0.149896835 | SCNN1B/SLC12A3/SCNN1G | 3 |
| GO:0022412 | BP | GO:0022412 | cellular process involved in reproduction in multicellular organism | 26/695 | 471/18870 | 0.026871206 | 0.175933255 | 0.149896835 | TNFAIP6/HSPA2/INHBB/ANGPT2/SFRP1/STRA8/TEX15/SERPINA5/TEX11/SEMG2/PDILT/RBM46/PAEP/FMN2/SEMG1/HORMAD1/SPATA16/FOLR3/DNMT3L/CATSPERD/FAM9A/FSHB/DMRT1/GALNTL5/NANOS2/DMRTC2 | 26 |
| GO:0021537 | BP | GO:0021537 | telencephalon development | 17/695 | 276/18870 | 0.027273042 | 0.178308729 | 0.151920762 | UNCX/DLX5/PROX1/HTR6/EOMES/DLX1/AVPR2/HES5/DLX2/SLITRK5/ERBB4/SCN2A/NR2E1/MGARP/LHX2/LHX1/ARX | 17 |
| GO:0043270 | BP | GO:0043270 | positive regulation of monoatomic ion transport | 14/695 | 214/18870 | 0.027460728 | 0.179279322 | 0.152747717 | TREM2/NOS1/MCHR1/CCL5/CHP2/CASR/CXCL9/CXCL10/CXCL11/IFNG/F2/P2RX2/CNTN1/NKX2-5 | 14 |
| GO:0140013 | BP | GO:0140013 | meiotic nuclear division | 13/695 | 194/18870 | 0.027727164 | 0.180760539 | 0.154009728 | HSPA2/RNF212B/STRA8/TEX15/TEX11/RBM46/MSH4/FMN2/HORMAD1/DNMT3L/DMRT1/NANOS2/DMRTC2 | 13 |
| GO:0098878 | CC | GO:0098878 | neurotransmitter receptor complex | 5/743 | 46/19886 | 0.027849562 | 0.194585164 | 0.17023357 | GRIK3/OLFM3/GRIA4/GRIK5/CACNG2 | 5 |
| GO:0035580 | CC | GO:0035580 | specific granule lumen | 6/743 | 62/19886 | 0.027886106 | 0.194585164 | 0.17023357 | CHIT1/HP/OLFM4/FOLR3/ORM2/ORM1 | 6 |
| GO:0002437 | BP | GO:0002437 | inflammatory response to antigenic stimulus | 7/695 | 80/18870 | 0.027889972 | 0.181047117 | 0.154253895 | TREM2/FCGR3A/IL20RB/CD68/FCGR1A/C3/PLA2G2D | 7 |
| GO:0003407 | BP | GO:0003407 | neural retina development | 7/695 | 80/18870 | 0.027889972 | 0.181047117 | 0.154253895 | CALB1/DLX1/ATP2B2/TFAP2B/DLX2/VSX1/LHX1 | 7 |
| GO:0007193 | BP | GO:0007193 | adenylate cyclase-inhibiting G protein-coupled receptor signaling pathway | 7/695 | 80/18870 | 0.027889972 | 0.181047117 | 0.154253895 | GRIK3/RGS1/MCHR1/CASR/GRM1/MTNR1A/NPY2R | 7 |
| GO:0098982 | CC | GO:0098982 | GABA-ergic synapse | 7/743 | 79/19886 | 0.028106746 | 0.194585164 | 0.17023357 | GABRD/CALB1/PTPRO/ATP2B2/ERBB4/GABRA2/SST | 7 |
| GO:0051784 | BP | GO:0051784 | negative regulation of nuclear division | 6/695 | 63/18870 | 0.028112071 | 0.182230019 | 0.155261739 | USP44/AURKB/HORMAD1/BMP7/DMRT1/NANOS2 | 6 |
| GO:0050798 | BP | GO:0050798 | activated T cell proliferation | 5/695 | 47/18870 | 0.028655694 | 0.185490821 | 0.158039974 | LILRB4/TNFSF9/CRTAM/HHLA2/EPO | 5 |
| GO:1904659 | BP | GO:1904659 | glucose transmembrane transport | 9/695 | 117/18870 | 0.028925746 | 0.185898711 | 0.1583875 | HK2/C3/TRIB3/SLC5A2/GPC3/ITLN1/SLC2A12/TERT/SLC5A11 | 9 |
| GO:0010765 | BP | GO:0010765 | positive regulation of sodium ion transport | 4/695 | 32/18870 | 0.028959839 | 0.185898711 | 0.1583875 | NOS1/CHP2/CNTN1/NKX2-5 | 4 |
| GO:0045684 | BP | GO:0045684 | positive regulation of epidermis development | 4/695 | 32/18870 | 0.028959839 | 0.185898711 | 0.1583875 | ALOX15B/CYP27B1/SULT2B1/OVOL2 | 4 |
| GO:0048710 | BP | GO:0048710 | regulation of astrocyte differentiation | 4/695 | 32/18870 | 0.028959839 | 0.185898711 | 0.1583875 | TREM2/HES5/F2/NR2E1 | 4 |
| GO:0072677 | BP | GO:0072677 | eosinophil migration | 4/695 | 32/18870 | 0.028959839 | 0.185898711 | 0.1583875 | CCL18/CCL5/SCG2/CCL25 | 4 |
| GO:0061982 | BP | GO:0061982 | meiosis I cell cycle process | 10/695 | 136/18870 | 0.028962775 | 0.185898711 | 0.1583875 | HSPA2/RNF212B/TEX15/TEX11/MSH4/FMN2/HORMAD1/DNMT3L/DMRT1/DMRTC2 | 10 |
| GO:0001667 | BP | GO:0001667 | ameboidal-type cell migration | 27/695 | 497/18870 | 0.029123882 | 0.186670603 | 0.159045159 | SCARB1/VEGFA/SEMA5B/FGF1/EGF/ANGPT2/PROX1/HRG/IFNG/GPC3/SCG2/S100A2/MMP9/GATA3/CXCL13/TACSTD2/ERBB4/EPB41L4B/NR2E1/GBX2/FGF10/OVOL2/RAB25/BMP7/APELA/PLG/FGFBP1 | 27 |
| GO:0005253 | MF | GO:0005253 | monoatomic anion channel activity | 8/709 | 95/18496 | 0.029371517 | 0.178727091 | 0.152298774 | GABRD/CLIC5/ANO4/CLCNKA/NMUR2/GABRA2/CLCNKB/BSND | 8 |
| GO:0008344 | BP | GO:0008344 | adult locomotory behavior | 7/695 | 81/18870 | 0.029596859 | 0.189171535 | 0.161175978 | KCNJ10/LGI4/CHL1/EN1/DMRT3/DMBX1/KLHL1 | 7 |
| GO:0048644 | BP | GO:0048644 | muscle organ morphogenesis | 7/695 | 81/18870 | 0.029596859 | 0.189171535 | 0.161175978 | PROX1/TNNT2/TNNI1/TNNC1/MYH7/POU4F1/NKX2-5 | 7 |
| GO:0005164 | MF | GO:0005164 | tumor necrosis factor receptor binding | 4/709 | 31/18496 | 0.029634272 | 0.178727091 | 0.152298774 | CD70/TNFSF9/FASLG/TNFSF14 | 4 |
| GO:0005310 | MF | GO:0005310 | dicarboxylic acid transmembrane transporter activity | 4/709 | 31/18496 | 0.029634272 | 0.178727091 | 0.152298774 | SLC26A4/SLC13A3/SLC13A2/SLC22A7 | 4 |
| GO:0071813 | MF | GO:0071813 | lipoprotein particle binding | 4/709 | 31/18496 | 0.029634272 | 0.178727091 | 0.152298774 | SCARB1/TREM2/APOL5/CRP | 4 |
| GO:0071814 | MF | GO:0071814 | protein-lipid complex binding | 4/709 | 31/18496 | 0.029634272 | 0.178727091 | 0.152298774 | SCARB1/TREM2/APOL5/CRP | 4 |
| GO:2001237 | BP | GO:2001237 | negative regulation of extrinsic apoptotic signaling pathway | 8/695 | 99/18870 | 0.029672395 | 0.189389446 | 0.16136164 | NOL3/EYA4/SCG2/FGG/TERT/FGF10/FGA/FGB | 8 |
| GO:0008391 | MF | GO:0008391 | arachidonic acid monooxygenase activity | 3/709 | 18/18496 | 0.029787848 | 0.178727091 | 0.152298774 | CYP2J2/CYP4F2/CYP2B6 | 3 |
| GO:0015373 | MF | GO:0015373 | monoatomic anion:sodium symporter activity | 3/709 | 18/18496 | 0.029787848 | 0.178727091 | 0.152298774 | SLC6A3/SLC12A1/SLC12A3 | 3 |
| GO:0030297 | MF | GO:0030297 | transmembrane receptor protein tyrosine kinase activator activity | 3/709 | 18/18496 | 0.029787848 | 0.178727091 | 0.152298774 | VEGFA/EGF/EPGN | 3 |
| GO:0009636 | BP | GO:0009636 | response to toxic substance | 16/695 | 258/18870 | 0.029891859 | 0.190524121 | 0.162328394 | GSTM3/INHBB/CCL5/SLC15A2/ALB/SLC47A2/SLC22A8/MT1G/HP/FABP1/MT1H/TTPA/MT3/BMP7/SLC30A10/APOA4 | 16 |
| GO:0002712 | BP | GO:0002712 | regulation of B cell mediated immunity | 6/695 | 64/18870 | 0.030090952 | 0.190993958 | 0.1627287 | TREM2/FCGR1A/C3/SUSD4/CR2/FOXJ1 | 6 |
| GO:0002820 | BP | GO:0002820 | negative regulation of adaptive immune response | 6/695 | 64/18870 | 0.030090952 | 0.190993958 | 0.1627287 | IL20RB/LILRB4/HLA-G/SUSD4/CR2/FOXJ1 | 6 |
| GO:0002889 | BP | GO:0002889 | regulation of immunoglobulin mediated immune response | 6/695 | 64/18870 | 0.030090952 | 0.190993958 | 0.1627287 | TREM2/FCGR1A/C3/SUSD4/CR2/FOXJ1 | 6 |
| GO:1903707 | BP | GO:1903707 | negative regulation of hemopoiesis | 9/695 | 118/18870 | 0.030334766 | 0.191525647 | 0.163181704 | TNFAIP6/LILRB4/SFRP1/TMEM178A/HLA-G/LAG3/RAG2/FOXJ1/PGLYRP2 | 9 |
| GO:0034764 | BP | GO:0034764 | positive regulation of transmembrane transport | 14/695 | 217/18870 | 0.030419395 | 0.191525647 | 0.163181704 | TREM2/NOS1/C3/CHP2/CXCL9/CXCL10/CXCL11/IFNG/GPC3/F2/ITLN1/TERT/P2RX2/SLC34A1 | 14 |
| GO:0150034 | CC | GO:0150034 | distal axon | 17/743 | 276/19886 | 0.030729071 | 0.209516392 | 0.183296212 | GRIK3/CALB1/SLC6A3/PTPRN/PTPRO/CRHBP/CASR/CDH8/SCGN/PCDH9/SLC18A3/L1CAM/STMN2/SNCB/CPLX2/FRMD7/CALCA | 17 |
| GO:0045137 | BP | GO:0045137 | development of primary sexual characteristics | 15/695 | 238/18870 | 0.030739242 | 0.191525647 | 0.163181704 | VEGFA/TNFAIP6/INHBB/SFRP1/TCF21/PTPRN/ADAM18/TEX11/GATA3/MSH4/DACH2/LHX8/GATA4/FSHB/DMRT1 | 15 |
| GO:0071356 | BP | GO:0071356 | cellular response to tumor necrosis factor | 15/695 | 238/18870 | 0.030739242 | 0.191525647 | 0.163181704 | CD70/TNFRSF4/BIRC7/SFRP1/CCL18/CCL5/CRHBP/UMOD/AIM2/GATA3/DCSTAMP/CCL20/CCL25/MYOD1/CALCA | 15 |
| GO:0034612 | BP | GO:0034612 | response to tumor necrosis factor | 16/695 | 259/18870 | 0.030820754 | 0.191525647 | 0.163181704 | CD70/TNFRSF4/BIRC7/SFRP1/CCL18/CCL5/CRHBP/UMOD/UBD/AIM2/GATA3/DCSTAMP/CCL20/CCL25/MYOD1/CALCA | 16 |
| GO:0030902 | BP | GO:0030902 | hindbrain development | 11/695 | 157/18870 | 0.030913919 | 0.191525647 | 0.163181704 | EGF/ABAT/PROX1/SPTBN2/EN1/CNTN1/GBX2/POU4F1/LHX1/BMP7/KLHL1 | 11 |
| GO:0002861 | BP | GO:0002861 | regulation of inflammatory response to antigenic stimulus | 5/695 | 48/18870 | 0.031052918 | 0.191525647 | 0.163181704 | TREM2/IL20RB/FCGR1A/C3/PLA2G2D | 5 |
| GO:0031295 | BP | GO:0031295 | T cell costimulation | 5/695 | 48/18870 | 0.031052918 | 0.191525647 | 0.163181704 | LILRB4/TNFSF14/ICOS/KLRK1/HHLA2 | 5 |
| GO:0042551 | BP | GO:0042551 | neuron maturation | 5/695 | 48/18870 | 0.031052918 | 0.191525647 | 0.163181704 | C3/LGI4/C1QL1/VSX1/ACTL6B | 5 |
| GO:0002281 | BP | GO:0002281 | macrophage activation involved in immune response | 3/695 | 19/18870 | 0.031054815 | 0.191525647 | 0.163181704 | TREM2/IFNG/LBP | 3 |
| GO:0002544 | BP | GO:0002544 | chronic inflammatory response | 3/695 | 19/18870 | 0.031054815 | 0.191525647 | 0.163181704 | IDO1/CCL5/CXCL13 | 3 |
| GO:0007620 | BP | GO:0007620 | copulation | 3/695 | 19/18870 | 0.031054815 | 0.191525647 | 0.163181704 | ABAT/EDDM3A/SEMG1 | 3 |
| GO:0010885 | BP | GO:0010885 | regulation of cholesterol storage | 3/695 | 19/18870 | 0.031054815 | 0.191525647 | 0.163181704 | SCARB1/TREM2/APOB | 3 |
| GO:0021513 | BP | GO:0021513 | spinal cord dorsal/ventral patterning | 3/695 | 19/18870 | 0.031054815 | 0.191525647 | 0.163181704 | RFX8/DMRT3/SOX1 | 3 |
| GO:0030540 | BP | GO:0030540 | female genitalia development | 3/695 | 19/18870 | 0.031054815 | 0.191525647 | 0.163181704 | STRA6/FGF10/LHX1 | 3 |
| GO:0032305 | BP | GO:0032305 | positive regulation of icosanoid secretion | 3/695 | 19/18870 | 0.031054815 | 0.191525647 | 0.163181704 | PLA2R1/AVPR1B/CYP4F2 | 3 |
| GO:0042053 | BP | GO:0042053 | regulation of dopamine metabolic process | 3/695 | 19/18870 | 0.031054815 | 0.191525647 | 0.163181704 | SLC6A3/ABAT/TACR3 | 3 |
| GO:0045618 | BP | GO:0045618 | positive regulation of keratinocyte differentiation | 3/695 | 19/18870 | 0.031054815 | 0.191525647 | 0.163181704 | ALOX15B/CYP27B1/OVOL2 | 3 |
| GO:0055003 | BP | GO:0055003 | cardiac myofibril assembly | 3/695 | 19/18870 | 0.031054815 | 0.191525647 | 0.163181704 | MYLK3/PROX1/NKX2-5 | 3 |
| GO:0060602 | BP | GO:0060602 | branch elongation of an epithelium | 3/695 | 19/18870 | 0.031054815 | 0.191525647 | 0.163181704 | FGF1/HOXD13/FGF10 | 3 |
| GO:0072376 | BP | GO:0072376 | protein activation cascade | 3/695 | 19/18870 | 0.031054815 | 0.191525647 | 0.163181704 | FGG/FGA/FGB | 3 |
| GO:0001786 | MF | GO:0001786 | phosphatidylserine binding | 6/709 | 62/18496 | 0.031059187 | 0.184887759 | 0.157548466 | SCARB1/TREM2/CD300LF/CPNE6/SYT7/GSDMC | 6 |
| GO:2000177 | BP | GO:2000177 | regulation of neural precursor cell proliferation | 8/695 | 100/18870 | 0.031255519 | 0.192503669 | 0.164014988 | VEGFA/EGF/PROX1/HAPLN1/RASSF10/NR2E1/LHX2/LHX1 | 8 |
| GO:0051341 | BP | GO:0051341 | regulation of oxidoreductase activity | 7/695 | 82/18870 | 0.031372797 | 0.192706561 | 0.164187854 | SCARB1/GZMA/CYP27B1/IFNG/HP/TERT/MT3 | 7 |
| GO:0070227 | BP | GO:0070227 | lymphocyte apoptotic process | 7/695 | 82/18870 | 0.031372797 | 0.192706561 | 0.164187854 | IDO1/BIRC7/FASLG/CCL5/AURKB/CD27/PDCD1 | 7 |
| GO:0030665 | CC | GO:0030665 | clathrin-coated vesicle membrane | 10/743 | 136/19886 | 0.031537698 | 0.209522137 | 0.183301238 | EGF/FCGR1A/AVPR2/EPGN/TYRP1/APOB/SLC18A3/TF/EPN3/ADCY8 | 10 |
| GO:0000794 | CC | GO:0000794 | condensed nuclear chromosome | 7/743 | 81/19886 | 0.031661123 | 0.209522137 | 0.183301238 | HSPA2/RNF212B/TEX11/MSH4/HORMAD1/DNMT3L/FAM9A | 7 |
| GO:0048306 | MF | GO:0048306 | calcium-dependent protein binding | 7/709 | 79/18496 | 0.03166423 | 0.187016858 | 0.159362736 | NOS1/S100A2/TNNC1/STMN2/S100G/CPLX2/MBL2 | 7 |
| GO:0040020 | BP | GO:0040020 | regulation of meiotic nuclear division | 4/695 | 33/18870 | 0.032024646 | 0.1956586 | 0.16670302 | STRA8/HORMAD1/DMRT1/NANOS2 | 4 |
| GO:0045671 | BP | GO:0045671 | negative regulation of osteoclast differentiation | 4/695 | 33/18870 | 0.032024646 | 0.1956586 | 0.16670302 | TNFAIP6/LILRB4/SFRP1/TMEM178A | 4 |
| GO:0090279 | BP | GO:0090279 | regulation of calcium ion import | 4/695 | 33/18870 | 0.032024646 | 0.1956586 | 0.16670302 | EGF/CASR/SEMG1/SLN | 4 |
| GO:1990806 | BP | GO:1990806 | ligand-gated ion channel signaling pathway | 4/695 | 33/18870 | 0.032024646 | 0.1956586 | 0.16670302 | GRIK3/F2/GRIA4/GRIK5 | 4 |
| GO:0030166 | BP | GO:0030166 | proteoglycan biosynthetic process | 6/695 | 65/18870 | 0.03215889 | 0.195693909 | 0.166733103 | HS6ST2/HS3ST2/NDST3/GAL3ST3/HS3ST6/HS3ST5 | 6 |
| GO:0045143 | BP | GO:0045143 | homologous chromosome segregation | 6/695 | 65/18870 | 0.03215889 | 0.195693909 | 0.166733103 | RNF212B/TEX15/TEX11/MSH4/FMN2/HORMAD1 | 6 |
| GO:0045453 | BP | GO:0045453 | bone resorption | 6/695 | 65/18870 | 0.03215889 | 0.195693909 | 0.166733103 | PTH1R/HAMP/DCSTAMP/TF/FSHB/CALCA | 6 |
| GO:0008585 | BP | GO:0008585 | female gonad development | 8/695 | 101/18870 | 0.032895498 | 0.199910143 | 0.170325376 | VEGFA/TNFAIP6/INHBB/SFRP1/PTPRN/MSH4/LHX8/FSHB | 8 |
| GO:0008645 | BP | GO:0008645 | hexose transmembrane transport | 9/695 | 120/18870 | 0.033295001 | 0.202069263 | 0.172164966 | HK2/C3/TRIB3/SLC5A2/GPC3/ITLN1/SLC2A12/TERT/SLC5A11 | 9 |
| GO:0008643 | BP | GO:0008643 | carbohydrate transport | 11/695 | 159/18870 | 0.033464043 | 0.20255719 | 0.172580685 | HK2/C3/TRIB3/SLC5A2/AQP2/GPC3/ITLN1/SLC2A12/TERT/SLC5A11/AQP9 | 11 |
| GO:0060041 | BP | GO:0060041 | retina development in camera-type eye | 11/695 | 159/18870 | 0.033464043 | 0.20255719 | 0.172580685 | CALB1/PROX1/DLX1/ATP2B2/TFAP2B/OPN4/DLX2/NR2E1/VSX1/LHX2/LHX1 | 11 |
| GO:0005543 | MF | GO:0005543 | phospholipid binding | 27/709 | 484/18496 | 0.033564744 | 0.19258874 | 0.164110705 | SCARB1/TREM2/DOC2A/APOC1/CD300LF/CPNE6/PLA2G7/KCNJ1/RUFY4/SERPINA5/AMPH/DPEP1/RAG2/SPTBN2/SLC9A3/APOB/PLA2G4D/CLVS2/SYT7/FABP1/EPN3/PLA2G2D/PIK3C2G/GPR12/TTPA/GSDMC/APOA4 | 27 |
| GO:2000404 | BP | GO:2000404 | regulation of T cell migration | 5/695 | 49/18870 | 0.033572203 | 0.20294308 | 0.172909468 | CCL5/TNFSF14/CXCL10/CXCL13/CCL20 | 5 |
| GO:0052689 | MF | GO:0052689 | carboxylic ester hydrolase activity | 11/709 | 153/18496 | 0.03367279 | 0.19258874 | 0.164110705 | TNFAIP6/CES4A/PLA2G7/CEL/ACOT12/NDST3/LIPH/PLA2G4D/PLA2G2D/BAAT/CA1 | 11 |
| GO:0051146 | BP | GO:0051146 | striated muscle cell differentiation | 18/695 | 305/18870 | 0.034029359 | 0.205434835 | 0.175032467 | VEGFA/MYLK3/NOS1/PROX1/NPHS1/TNFSF14/CXCL9/CXCL10/MYOZ2/TNNT2/TNNT1/P2RX2/TBX5/MYH7/NKX2-5/MYOD1/GATA4/ACTL8 | 18 |
| GO:0042446 | BP | GO:0042446 | hormone biosynthetic process | 6/695 | 66/18870 | 0.034317074 | 0.206625861 | 0.176047232 | STC2/CYP27B1/DIO1/HSD17B3/RDH8/FSHB | 6 |
| GO:1905953 | BP | GO:1905953 | negative regulation of lipid localization | 6/695 | 66/18870 | 0.034317074 | 0.206625861 | 0.176047232 | TREM2/APOC1/PLA2R1/EGF/CYP4F2/CRP | 6 |
| GO:0004970 | MF | GO:0004970 | ionotropic glutamate receptor activity | 3/709 | 19/18496 | 0.034390846 | 0.19258874 | 0.164110705 | GRIK3/GRIA4/GRIK5 | 3 |
| GO:0005372 | MF | GO:0005372 | water transmembrane transporter activity | 3/709 | 19/18496 | 0.034390846 | 0.19258874 | 0.164110705 | SLC4A11/AQP2/AQP9 | 3 |
| GO:0022821 | MF | GO:0022821 | solute:potassium antiporter activity | 3/709 | 19/18496 | 0.034390846 | 0.19258874 | 0.164110705 | SLC9A3/SLC9A4/SLC9A2 | 3 |
| GO:0022840 | MF | GO:0022840 | leak channel activity | 3/709 | 19/18496 | 0.034390846 | 0.19258874 | 0.164110705 | KCNK9/KCNK10/KCNK17 | 3 |
| GO:0022842 | MF | GO:0022842 | narrow pore channel activity | 3/709 | 19/18496 | 0.034390846 | 0.19258874 | 0.164110705 | KCNK9/KCNK10/KCNK17 | 3 |
| GO:0021510 | BP | GO:0021510 | spinal cord development | 8/695 | 102/18870 | 0.034593105 | 0.208013805 | 0.177229775 | SOX11/UNCX/PROX1/RFX8/ISL2/LHX1/DMRT3/SOX1 | 8 |
| GO:0030510 | BP | GO:0030510 | regulation of BMP signaling pathway | 9/695 | 121/18870 | 0.03484751 | 0.209268228 | 0.178298555 | TNFAIP6/SOX11/SFRP1/SOST/DLX1/GPC3/TFAP2B/HES5/GATA4 | 9 |
| GO:0002717 | BP | GO:0002717 | positive regulation of natural killer cell mediated immunity | 4/695 | 34/18870 | 0.035272265 | 0.209967053 | 0.17889396 | HLA-G/CRTAM/LAG3/KLRK1 | 4 |
| GO:0071353 | BP | GO:0071353 | cellular response to interleukin-4 | 4/695 | 34/18870 | 0.035272265 | 0.209967053 | 0.17889396 | CD300LF/RUFY4/GATA3/DCSTAMP | 4 |
| GO:0090183 | BP | GO:0090183 | regulation of kidney development | 4/695 | 34/18870 | 0.035272265 | 0.209967053 | 0.17889396 | VEGFA/GATA3/TACSTD2/LHX1 | 4 |
| GO:0003215 | BP | GO:0003215 | cardiac right ventricle morphogenesis | 3/695 | 20/18870 | 0.03556116 | 0.209967053 | 0.17889396 | GATA3/NKX2-5/GATA4 | 3 |
| GO:0021516 | BP | GO:0021516 | dorsal spinal cord development | 3/695 | 20/18870 | 0.03556116 | 0.209967053 | 0.17889396 | UNCX/PROX1/LHX1 | 3 |
| GO:0032303 | BP | GO:0032303 | regulation of icosanoid secretion | 3/695 | 20/18870 | 0.03556116 | 0.209967053 | 0.17889396 | PLA2R1/AVPR1B/CYP4F2 | 3 |
| GO:0042069 | BP | GO:0042069 | regulation of catecholamine metabolic process | 3/695 | 20/18870 | 0.03556116 | 0.209967053 | 0.17889396 | SLC6A3/ABAT/TACR3 | 3 |
| GO:0043011 | BP | GO:0043011 | myeloid dendritic cell differentiation | 3/695 | 20/18870 | 0.03556116 | 0.209967053 | 0.17889396 | BATF/UBD/DCSTAMP | 3 |
| GO:0051546 | BP | GO:0051546 | keratinocyte migration | 3/695 | 20/18870 | 0.03556116 | 0.209967053 | 0.17889396 | MMP9/EPB41L4B/FGF10 | 3 |
| GO:0060749 | BP | GO:0060749 | mammary gland alveolus development | 3/695 | 20/18870 | 0.03556116 | 0.209967053 | 0.17889396 | VEGFA/EGF/ERBB4 | 3 |
| GO:0061377 | BP | GO:0061377 | mammary gland lobule development | 3/695 | 20/18870 | 0.03556116 | 0.209967053 | 0.17889396 | VEGFA/EGF/ERBB4 | 3 |
| GO:0140131 | BP | GO:0140131 | positive regulation of lymphocyte chemotaxis | 3/695 | 20/18870 | 0.03556116 | 0.209967053 | 0.17889396 | CCL5/TNFSF14/CXCL13 | 3 |
| GO:1900221 | BP | GO:1900221 | regulation of amyloid-beta clearance | 3/695 | 20/18870 | 0.03556116 | 0.209967053 | 0.17889396 | TREM2/IFNG/TTPA | 3 |
| GO:0060047 | BP | GO:0060047 | heart contraction | 15/695 | 243/18870 | 0.035939836 | 0.211929095 | 0.180565639 | CYP2J2/NOS1/TNNT2/ATP2B2/TNNI1/TNNC1/HSPB7/TBX5/MYH7/TACR3/SGCZ/SCN10A/NKX2-5/APELA/GATA4 | 15 |
| GO:0031294 | BP | GO:0031294 | lymphocyte costimulation | 5/695 | 50/18870 | 0.036214855 | 0.21272736 | 0.181245769 | LILRB4/TNFSF14/ICOS/KLRK1/HHLA2 | 5 |
| GO:0071827 | BP | GO:0071827 | plasma lipoprotein particle organization | 5/695 | 50/18870 | 0.036214855 | 0.21272736 | 0.181245769 | SCARB1/APOC1/PLA2G7/APOB/APOA4 | 5 |
| GO:1902041 | BP | GO:1902041 | regulation of extrinsic apoptotic signaling pathway via death domain receptors | 5/695 | 50/18870 | 0.036214855 | 0.21272736 | 0.181245769 | SFRP1/FGG/MAL/FGA/FGB | 5 |
| GO:0030296 | MF | GO:0030296 | protein tyrosine kinase activator activity | 4/709 | 33/18496 | 0.036311285 | 0.201848027 | 0.172000825 | VEGFA/EGF/CCL5/EPGN | 4 |
| GO:0006885 | BP | GO:0006885 | regulation of pH | 8/695 | 103/18870 | 0.03634908 | 0.212968328 | 0.181451075 | ATP12A/FASLG/SLC26A4/SLC9A3/SLC9A4/SLC9A2/SLC4A9/SLC4A1 | 8 |
| GO:0015914 | BP | GO:0015914 | phospholipid transport | 8/695 | 103/18870 | 0.03634908 | 0.212968328 | 0.181451075 | SCARB1/APOC1/ATP8B3/ABCA4/FASLG/ANO4/SLC4A1/APOA4 | 8 |
| GO:0034361 | CC | GO:0034361 | very-low-density lipoprotein particle | 3/743 | 20/19886 | 0.036881394 | 0.237094677 | 0.20742318 | APOC1/APOB/APOA4 | 3 |
| GO:0034385 | CC | GO:0034385 | triglyceride-rich plasma lipoprotein particle | 3/743 | 20/19886 | 0.036881394 | 0.237094677 | 0.20742318 | APOC1/APOB/APOA4 | 3 |
| GO:0060560 | BP | GO:0060560 | developmental growth involved in morphogenesis | 15/695 | 244/18870 | 0.037051982 | 0.216808651 | 0.184723068 | VEGFA/SEMA5B/FGF1/CPNE6/SFRP1/FLRT1/CDH4/HOXD13/PAK6/WNT7B/MT3/LHX2/FGF10/L1CAM/LHX1 | 15 |
| GO:0021700 | BP | GO:0021700 | developmental maturation | 19/695 | 330/18870 | 0.037173164 | 0.21723959 | 0.185090233 | VEGFA/PTPRN/C3/LGI4/PTH1R/SEMG2/HES5/GATA3/FGG/PAEP/C1QL1/EPO/SEMG1/VSX1/FOXJ1/TFCP2L1/HOXB13/CATSPERD/ACTL6B | 19 |
| GO:0015749 | BP | GO:0015749 | monosaccharide transmembrane transport | 9/695 | 123/18870 | 0.038100378 | 0.222373855 | 0.189464676 | HK2/C3/TRIB3/SLC5A2/GPC3/ITLN1/SLC2A12/TERT/SLC5A11 | 9 |
| GO:0021549 | BP | GO:0021549 | cerebellum development | 8/695 | 104/18870 | 0.038164127 | 0.222461809 | 0.189539614 | ABAT/PROX1/SPTBN2/EN1/CNTN1/GBX2/LHX1/KLHL1 | 8 |
| GO:0030139 | CC | GO:0030139 | endocytic vesicle | 20/743 | 348/19886 | 0.038293756 | 0.242706906 | 0.212333059 | SCARB1/EGF/CPNE6/FCGR1A/GNLY/SLC15A2/HLA-G/AVPR2/EPGN/SCGB3A2/TYRP1/HP/APOB/SYT7/SLC18A3/TF/GRIA4/SAA1/WNT7B/CACNG2 | 20 |
| GO:0052548 | BP | GO:0052548 | regulation of endopeptidase activity | 17/695 | 288/18870 | 0.038538872 | 0.224360059 | 0.191156941 | EGLN3/NOL3/VEGFA/HRG/FASLG/KNG1/CST7/SERPINA5/DPEP1/SEMG2/MMP9/AIM2/FABP1/SEMG1/SERPINA12/SERPINA9/MAGEA3 | 17 |
| GO:0015701 | BP | GO:0015701 | bicarbonate transport | 4/695 | 35/18870 | 0.0387035 | 0.224460656 | 0.191242651 | SLC4A11/SLC26A4/SLC4A9/SLC4A1 | 4 |
| GO:0048048 | BP | GO:0048048 | embryonic eye morphogenesis | 4/695 | 35/18870 | 0.0387035 | 0.224460656 | 0.191242651 | SOX11/PROX1/STRA6/BMP7 | 4 |
| GO:0098810 | BP | GO:0098810 | neurotransmitter reuptake | 4/695 | 35/18870 | 0.0387035 | 0.224460656 | 0.191242651 | KCNJ10/SLC6A3/NOS1/SLC18A3 | 4 |
| GO:0032813 | MF | GO:0032813 | tumor necrosis factor receptor superfamily binding | 5/709 | 49/18496 | 0.038850338 | 0.214385805 | 0.182684646 | NOL3/CD70/TNFSF9/FASLG/TNFSF14 | 5 |
| GO:0034341 | BP | GO:0034341 | response to type II interferon | 10/695 | 143/18870 | 0.038889471 | 0.224776272 | 0.191511558 | CCL18/FASLG/CCL5/GBP5/CYP27B1/IFNG/UBD/CCL20/SLC30A8/CCL25 | 10 |
| GO:0051047 | BP | GO:0051047 | positive regulation of secretion | 18/695 | 310/18870 | 0.038926343 | 0.224776272 | 0.191511558 | TREM2/SOX11/INHBB/PLA2R1/ABAT/CASR/AVPR1B/IFNG/TFR2/F2/FGG/CYP4F2/SYT7/SLC18A3/SLC30A8/ADCY8/FGA/FGB | 18 |
| GO:0035094 | BP | GO:0035094 | response to nicotine | 5/695 | 51/18870 | 0.038981989 | 0.224776272 | 0.191511558 | SLC6A3/ABAT/CHRNA1/CHRNA6/CHRNA4 | 5 |
| GO:0046622 | BP | GO:0046622 | positive regulation of organ growth | 5/695 | 51/18870 | 0.038981989 | 0.224776272 | 0.191511558 | PROX1/RAG2/TBX5/ERBB4/ARX | 5 |
| GO:0050905 | BP | GO:0050905 | neuromuscular process | 11/695 | 163/18870 | 0.039003848 | 0.224776272 | 0.191511558 | SLC6A3/CHRNA1/TNNI1/STRA6/TNNC1/TNNT1/MYH7/NKX6-2/CHRND/PCDH15/POU4F1 | 11 |
| GO:2000243 | BP | GO:2000243 | positive regulation of reproductive process | 7/695 | 86/18870 | 0.039185999 | 0.225541583 | 0.192163611 | VEGFA/INHBB/STRA8/RBM46/TACR3/APELA/DMRT1 | 7 |
| GO:0042288 | MF | GO:0042288 | MHC class I protein binding | 3/709 | 20/18496 | 0.039338803 | 0.215508224 | 0.183641093 | CD8A/CD8B/KLRK1 | 3 |
| GO:0048863 | BP | GO:0048863 | stem cell differentiation | 15/695 | 246/18870 | 0.039350395 | 0.226202897 | 0.192727056 | SEMA5B/ESRRB/SLC4A11/SFRP1/EOMES/BATF/HES5/TACSTD2/TBX5/ERBB4/GBX2/DNMT3L/OVOL2/NKX2-5/BMP7 | 15 |
| GO:0002761 | BP | GO:0002761 | regulation of myeloid leukocyte differentiation | 9/695 | 124/18870 | 0.039801894 | 0.228510875 | 0.194693476 | TREM2/TNFAIP6/LILRB4/SFRP1/TMEM178A/IFNG/DCSTAMP/POU4F1/FSHB | 9 |
| GO:0015020 | MF | GO:0015020 | glucuronosyltransferase activity | 4/709 | 34/18496 | 0.039948482 | 0.21727376 | 0.18514556 | UGT1A3/UGT1A10/UGT2A1/UGT1A4 | 4 |
| GO:0006067 | BP | GO:0006067 | ethanol metabolic process | 3/695 | 21/18870 | 0.040384396 | 0.228978523 | 0.195091916 | ADH1C/ALDH3B2/SULT2A1 | 3 |
| GO:0021511 | BP | GO:0021511 | spinal cord patterning | 3/695 | 21/18870 | 0.040384396 | 0.228978523 | 0.195091916 | RFX8/DMRT3/SOX1 | 3 |
| GO:0034104 | BP | GO:0034104 | negative regulation of tissue remodeling | 3/695 | 21/18870 | 0.040384396 | 0.228978523 | 0.195091916 | SFRP1/HAMP/CALCA | 3 |
| GO:0043651 | BP | GO:0043651 | linoleic acid metabolic process | 3/695 | 21/18870 | 0.040384396 | 0.228978523 | 0.195091916 | CYP2J2/ALOX15B/ELOVL2 | 3 |
| GO:0044321 | BP | GO:0044321 | response to leptin | 3/695 | 21/18870 | 0.040384396 | 0.228978523 | 0.195091916 | INHBB/MT3/FGB | 3 |
| GO:0061318 | BP | GO:0061318 | renal filtration cell differentiation | 3/695 | 21/18870 | 0.040384396 | 0.228978523 | 0.195091916 | PTPRO/NPHS1/NPHS2 | 3 |
| GO:0072112 | BP | GO:0072112 | podocyte differentiation | 3/695 | 21/18870 | 0.040384396 | 0.228978523 | 0.195091916 | PTPRO/NPHS1/NPHS2 | 3 |
| GO:0090026 | BP | GO:0090026 | positive regulation of monocyte chemotaxis | 3/695 | 21/18870 | 0.040384396 | 0.228978523 | 0.195091916 | PLA2G7/CCL5/CXCL10 | 3 |
| GO:0090189 | BP | GO:0090189 | regulation of branching involved in ureteric bud morphogenesis | 3/695 | 21/18870 | 0.040384396 | 0.228978523 | 0.195091916 | VEGFA/TACSTD2/LHX1 | 3 |
| GO:2000738 | BP | GO:2000738 | positive regulation of stem cell differentiation | 3/695 | 21/18870 | 0.040384396 | 0.228978523 | 0.195091916 | TACSTD2/TBX5/NKX2-5 | 3 |
| GO:0016235 | CC | GO:0016235 | aggresome | 4/743 | 35/19886 | 0.04047123 | 0.2497412 | 0.218487038 | KLHL14/PRDM16/UBD/HSPB7 | 4 |
| GO:0045926 | BP | GO:0045926 | negative regulation of growth | 15/695 | 247/18870 | 0.040537165 | 0.229029176 | 0.195135074 | STC2/CDKN2A/SEMA5B/SFRP1/HRG/ALOX15B/CYP27B1/TP73/GPC3/MT1G/DCSTAMP/TBX5/MT1H/TMPRSS4/MT3 | 15 |
| GO:0090257 | BP | GO:0090257 | regulation of muscle system process | 15/695 | 247/18870 | 0.040537165 | 0.229029176 | 0.195135074 | NOS1/ABAT/TNNT2/TNNI1/TNNC1/MYBPH/TNNT1/MYH7/NMUR2/TACR3/SCN10A/NKX2-5/SLN/GATA4/CALCA | 15 |
| GO:0016064 | BP | GO:0016064 | immunoglobulin mediated immune response | 13/695 | 205/18870 | 0.040543677 | 0.229029176 | 0.195135074 | TREM2/FCGR3A/IL2RB/FCGR1A/C3/AICDA/BATF/CD27/SUSD4/C7/CR2/FOXJ1/MBL2 | 13 |
| GO:0052547 | BP | GO:0052547 | regulation of peptidase activity | 18/695 | 312/18870 | 0.041022278 | 0.231446677 | 0.197194808 | EGLN3/NOL3/VEGFA/HRG/FASLG/KNG1/CST7/SERPINA5/DPEP1/SEMG2/MMP9/AIM2/FABP1/SEMG1/SERPINA12/PRSS22/SERPINA9/MAGEA3 | 18 |
| GO:0005782 | CC | GO:0005782 | peroxisomal matrix | 5/743 | 51/19886 | 0.041068553 | 0.2497412 | 0.218487038 | AGXT/FABP1/DAO/BAAT/HAO1 | 5 |
| GO:0031907 | CC | GO:0031907 | microbody lumen | 5/743 | 51/19886 | 0.041068553 | 0.2497412 | 0.218487038 | AGXT/FABP1/DAO/BAAT/HAO1 | 5 |
| GO:0035019 | BP | GO:0035019 | somatic stem cell population maintenance | 6/695 | 69/18870 | 0.041343554 | 0.232398575 | 0.198005834 | POU5F1/SFRP1/WNT9B/NR2E1/FGF10/BMP7 | 6 |
| GO:0043627 | BP | GO:0043627 | response to estrogen | 6/695 | 69/18870 | 0.041343554 | 0.232398575 | 0.198005834 | GSTM3/SFRP1/CRHBP/CYP27B1/GATA3/EPO | 6 |
| GO:2000401 | BP | GO:2000401 | regulation of lymphocyte migration | 6/695 | 69/18870 | 0.041343554 | 0.232398575 | 0.198005834 | CCL5/TNFSF14/CXCL10/KLRK1/CXCL13/CCL20 | 6 |
| GO:0048020 | MF | GO:0048020 | CCR chemokine receptor binding | 5/709 | 50/18496 | 0.041859892 | 0.226043418 | 0.192618451 | CCL18/CCL5/CXCL13/CCL20/CCL25 | 5 |
| GO:0032426 | CC | GO:0032426 | stereocilium tip | 3/743 | 21/19886 | 0.041867529 | 0.251205176 | 0.219767803 | MYO3A/MYO3B/LHFPL5 | 3 |
| GO:0045332 | BP | GO:0045332 | phospholipid translocation | 5/695 | 52/18870 | 0.041874533 | 0.234805662 | 0.200056695 | ATP8B3/ABCA4/FASLG/ANO4/SLC4A1 | 5 |
| GO:0051180 | BP | GO:0051180 | vitamin transport | 5/695 | 52/18870 | 0.041874533 | 0.234805662 | 0.200056695 | SCARB1/STRA6/AFM/TTPA/FOLR3 | 5 |
| GO:0006641 | BP | GO:0006641 | triglyceride metabolic process | 8/695 | 106/18870 | 0.041974075 | 0.235075396 | 0.200286512 | SCARB1/APOC1/C3/APOB/MOGAT2/SERPINA12/NKX2-3/APOA4 | 8 |
| GO:0045589 | BP | GO:0045589 | regulation of regulatory T cell differentiation | 4/695 | 36/18870 | 0.042318731 | 0.236137487 | 0.201191423 | LILRB4/HLA-G/IFNG/LAG3 | 4 |
| GO:0046164 | BP | GO:0046164 | alcohol catabolic process | 4/695 | 36/18870 | 0.042318731 | 0.236137487 | 0.201191423 | SCARB1/ALDH3B2/HAO1/SULT2A1 | 4 |
| GO:0070670 | BP | GO:0070670 | response to interleukin-4 | 4/695 | 36/18870 | 0.042318731 | 0.236137487 | 0.201191423 | CD300LF/RUFY4/GATA3/DCSTAMP | 4 |
| GO:0006941 | BP | GO:0006941 | striated muscle contraction | 12/695 | 186/18870 | 0.042876303 | 0.238956956 | 0.203593638 | NOS1/CHRNA1/TNNT2/TNNI1/TNNC1/MYBPH/TNNT1/MYH7/CHRND/SCN10A/NKX2-5/GATA4 | 12 |
| GO:0002703 | BP | GO:0002703 | regulation of leukocyte mediated immunity | 15/695 | 249/18870 | 0.042987057 | 0.239282401 | 0.20387092 | TREM2/IL20RB/PIK3R6/LILRB4/FCGR1A/C3/HLA-G/CRTAM/LAG3/KLRK1/SUSD4/GATA3/CLEC12B/CR2/FOXJ1 | 15 |
| GO:0051216 | BP | GO:0051216 | cartilage development | 13/695 | 207/18870 | 0.043259862 | 0.240507994 | 0.204915137 | PTHLH/UNCX/GDF6/PTH1R/TGFBI/HES5/GATA3/DLX2/WNT7B/EPYC/PITX1/MMP13/BMP7 | 13 |
| GO:0005154 | MF | GO:0005154 | epidermal growth factor receptor binding | 4/709 | 35/18496 | 0.043785333 | 0.234763914 | 0.20004945 | EGF/EPGN/ERBB4/FAM83B | 4 |
| GO:0035107 | BP | GO:0035107 | appendage morphogenesis | 10/695 | 146/18870 | 0.043785356 | 0.242544338 | 0.206650122 | DLX5/GPC3/TFAP2B/HOXD13/EN1/TBX5/GRHL2/FGF10/PITX1/BMP7 | 10 |
| GO:0035108 | BP | GO:0035108 | limb morphogenesis | 10/695 | 146/18870 | 0.043785356 | 0.242544338 | 0.206650122 | DLX5/GPC3/TFAP2B/HOXD13/EN1/TBX5/GRHL2/FGF10/PITX1/BMP7 | 10 |
| GO:0051783 | BP | GO:0051783 | regulation of nuclear division | 10/695 | 146/18870 | 0.043785356 | 0.242544338 | 0.206650122 | CDCA2/EGF/USP44/AURKB/STRA8/EPGN/HORMAD1/BMP7/DMRT1/NANOS2 | 10 |
| GO:0007281 | BP | GO:0007281 | germ cell development | 20/695 | 359/18870 | 0.044203526 | 0.244564303 | 0.208371152 | TNFAIP6/HSPA2/INHBB/ANGPT2/STRA8/SEMG2/PDILT/RBM46/PAEP/FMN2/SEMG1/HORMAD1/SPATA16/DNMT3L/CATSPERD/FAM9A/DMRT1/GALNTL5/NANOS2/DMRTC2 | 20 |
| GO:0097191 | BP | GO:0097191 | extrinsic apoptotic signaling pathway | 14/695 | 229/18870 | 0.044599904 | 0.245312142 | 0.209008318 | NOL3/CD70/SFRP1/FASLG/EYA4/IFNG/CD27/SCG2/FGG/TERT/MAL/FGF10/FGA/FGB | 14 |
| GO:0005326 | MF | GO:0005326 | neurotransmitter transmembrane transporter activity | 3/709 | 21/18496 | 0.044626454 | 0.237588729 | 0.202456561 | SLC6A3/SLC28A2/SLC18A3 | 3 |
| GO:0007129 | BP | GO:0007129 | homologous chromosome pairing at meiosis | 5/695 | 53/18870 | 0.044893222 | 0.245312142 | 0.209008318 | RNF212B/TEX15/TEX11/MSH4/HORMAD1 | 5 |
| GO:0007157 | BP | GO:0007157 | heterophilic cell-cell adhesion via plasma membrane cell adhesion molecules | 5/695 | 53/18870 | 0.044893222 | 0.245312142 | 0.209008318 | CRTAM/UMOD/CDH4/CADM3/REG3A | 5 |
| GO:0048260 | BP | GO:0048260 | positive regulation of receptor-mediated endocytosis | 5/695 | 53/18870 | 0.044893222 | 0.245312142 | 0.209008318 | VEGFA/EGF/C3/TF/APELA | 5 |
| GO:0071825 | BP | GO:0071825 | protein-lipid complex organization | 5/695 | 53/18870 | 0.044893222 | 0.245312142 | 0.209008318 | SCARB1/APOC1/PLA2G7/APOB/APOA4 | 5 |
| GO:0048639 | BP | GO:0048639 | positive regulation of developmental growth | 11/695 | 167/18870 | 0.045152205 | 0.245312142 | 0.209008318 | VEGFA/SLC6A3/CPNE6/PROX1/CDH4/RAG2/TBX5/ERBB4/L1CAM/ARX/MYOD1 | 11 |
| GO:0042063 | BP | GO:0042063 | gliogenesis | 19/695 | 338/18870 | 0.045436956 | 0.245312142 | 0.209008318 | TREM2/KCNJ10/SOX11/LGI4/EOMES/DLX1/HAPLN1/IFNG/TP73/ZNF488/HES5/F2/DLX2/MAL/CNTN1/NKX6-2/NR2E1/FGF10/SOX1 | 19 |
| GO:0002089 | BP | GO:0002089 | lens morphogenesis in camera-type eye | 3/695 | 22/18870 | 0.045519627 | 0.245312142 | 0.209008318 | SOX11/PROX1/SOX1 | 3 |
| GO:0003228 | BP | GO:0003228 | atrial cardiac muscle tissue development | 3/695 | 22/18870 | 0.045519627 | 0.245312142 | 0.209008318 | PROX1/TBX5/NKX2-5 | 3 |
| GO:0007530 | BP | GO:0007530 | sex determination | 3/695 | 22/18870 | 0.045519627 | 0.245312142 | 0.209008318 | TCF21/INSRR/DMRT1 | 3 |
| GO:0009713 | BP | GO:0009713 | catechol-containing compound biosynthetic process | 3/695 | 22/18870 | 0.045519627 | 0.245312142 | 0.209008318 | SLC6A3/GATA3/DAO | 3 |
| GO:0042423 | BP | GO:0042423 | catecholamine biosynthetic process | 3/695 | 22/18870 | 0.045519627 | 0.245312142 | 0.209008318 | SLC6A3/GATA3/DAO | 3 |
| GO:0044241 | BP | GO:0044241 | lipid digestion | 3/695 | 22/18870 | 0.045519627 | 0.245312142 | 0.209008318 | CEL/ARX/APOA4 | 3 |
| GO:0048485 | BP | GO:0048485 | sympathetic nervous system development | 3/695 | 22/18870 | 0.045519627 | 0.245312142 | 0.209008318 | SOX11/TFAP2B/GATA3 | 3 |
| GO:0051923 | BP | GO:0051923 | sulfation | 3/695 | 22/18870 | 0.045519627 | 0.245312142 | 0.209008318 | SULT2B1/HS3ST5/SULT2A1 | 3 |
| GO:0060713 | BP | GO:0060713 | labyrinthine layer morphogenesis | 3/695 | 22/18870 | 0.045519627 | 0.245312142 | 0.209008318 | WNT7B/GRHL2/BMP7 | 3 |
| GO:0061687 | BP | GO:0061687 | detoxification of inorganic compound | 3/695 | 22/18870 | 0.045519627 | 0.245312142 | 0.209008318 | MT1G/MT1H/MT3 | 3 |
| GO:0071305 | BP | GO:0071305 | cellular response to vitamin D | 3/695 | 22/18870 | 0.045519627 | 0.245312142 | 0.209008318 | SFRP1/CASR/CYP27B1 | 3 |
| GO:0072111 | BP | GO:0072111 | cell proliferation involved in kidney development | 3/695 | 22/18870 | 0.045519627 | 0.245312142 | 0.209008318 | GPC3/GATA3/BMP7 | 3 |
| GO:0098856 | BP | GO:0098856 | intestinal lipid absorption | 3/695 | 22/18870 | 0.045519627 | 0.245312142 | 0.209008318 | SCARB1/CEL/APOA4 | 3 |
| GO:1903589 | BP | GO:1903589 | positive regulation of blood vessel endothelial cell proliferation involved in sprouting angiogenesis | 3/695 | 22/18870 | 0.045519627 | 0.245312142 | 0.209008318 | VEGFA/APELA/FGFBP1 | 3 |
| GO:2000679 | BP | GO:2000679 | positive regulation of transcription regulatory region DNA binding | 3/695 | 22/18870 | 0.045519627 | 0.245312142 | 0.209008318 | IFNG/GATA3/POU4F1 | 3 |
| GO:0005518 | MF | GO:0005518 | collagen binding | 6/709 | 68/18496 | 0.045760948 | 0.241925012 | 0.206151639 | COL5A3/VWF/LOX/TGFBI/MMP9/MMP13 | 6 |
| GO:0015844 | BP | GO:0015844 | monoamine transport | 7/695 | 89/18870 | 0.045813857 | 0.245926376 | 0.209531651 | SLC6A3/NOS1/ABAT/CHRNA6/SYT7/SLC18A3/CHRNA4 | 7 |
| GO:0002685 | BP | GO:0002685 | regulation of leukocyte migration | 14/695 | 230/18870 | 0.045961033 | 0.245926376 | 0.209531651 | TREM2/VEGFA/TNFAIP6/PGF/PLA2G7/CCL5/TNFSF14/CXCL10/CXCR3/KLRK1/CXCL13/CCL20/CCL25/LBP | 14 |
| GO:0003161 | BP | GO:0003161 | cardiac conduction system development | 4/695 | 37/18870 | 0.046117922 | 0.245926376 | 0.209531651 | MSC/TBX5/NKX2-5/GATA4 | 4 |
| GO:0003230 | BP | GO:0003230 | cardiac atrium development | 4/695 | 37/18870 | 0.046117922 | 0.245926376 | 0.209531651 | PROX1/TBX5/NKX2-5/GATA4 | 4 |
| GO:0014059 | BP | GO:0014059 | regulation of dopamine secretion | 4/695 | 37/18870 | 0.046117922 | 0.245926376 | 0.209531651 | ABAT/CHRNA6/SYT7/CHRNA4 | 4 |
| GO:0044058 | BP | GO:0044058 | regulation of digestive system process | 4/695 | 37/18870 | 0.046117922 | 0.245926376 | 0.209531651 | WNK4/HAMP/FGF10/APOA4 | 4 |
| GO:0046688 | BP | GO:0046688 | response to copper ion | 4/695 | 37/18870 | 0.046117922 | 0.245926376 | 0.209531651 | AQP2/MT1G/MT1H/MT3 | 4 |
| GO:0071276 | BP | GO:0071276 | cellular response to cadmium ion | 4/695 | 37/18870 | 0.046117922 | 0.245926376 | 0.209531651 | MMP9/MT1G/MT1H/MT3 | 4 |
| GO:0071312 | BP | GO:0071312 | cellular response to alkaloid | 4/695 | 37/18870 | 0.046117922 | 0.245926376 | 0.209531651 | CRHBP/SLC34A1/PPP1R1B/ADCY8 | 4 |
| GO:0050890 | BP | GO:0050890 | cognition | 18/695 | 317/18870 | 0.046617794 | 0.248302237 | 0.211555908 | TREM2/NPTX2/CALB1/DNAH11/CRHBP/JAKMIP1/KCNK10/STRA6/CHL1/EN1/C1QL1/LHX8/SCN2A/PPP1R1B/PAK6/SORCS3/ADCY8/CHRNA4 | 18 |
| GO:0001227 | MF | GO:0001227 | DNA-binding transcription repressor activity, RNA polymerase II-specific | 19/709 | 326/18496 | 0.046783261 | 0.24561212 | 0.209293536 | IRX2/POU5F1/TCF21/PROX1/PRDM16/IRX1/MSC/TBX15/HELT/HES5/GATA3/EN1/NKX6-2/NR2E1/OVOL2/POU4F1/HOXB13/ARX/DMBX1 | 19 |
| GO:0010927 | BP | GO:0010927 | cellular component assembly involved in morphogenesis | 9/695 | 128/18870 | 0.047120856 | 0.250398038 | 0.21334155 | MYLK3/PROX1/MYOZ2/TNNT2/TNNT1/MYH7/CNTN1/NKX2-5/ACTL8 | 9 |
| GO:0030301 | BP | GO:0030301 | cholesterol transport | 9/695 | 128/18870 | 0.047120856 | 0.250398038 | 0.21334155 | SCARB1/TREM2/APOC1/EGF/CEL/APOB/SYT7/ABCA13/APOA4 | 9 |
| GO:0003012 | BP | GO:0003012 | muscle system process | 24/695 | 452/18870 | 0.047321239 | 0.251170803 | 0.213999954 | NOS1/ABAT/MYOZ2/CHRNA1/TNNT2/TNNI1/TNNC1/MYBPH/TNNT1/P2RX2/SCN7A/MYH7/SCNN1B/CHRND/NMUR2/MYH13/ANKRD2/TACR3/SCN10A/NKX2-5/SLN/MYOD1/GATA4/CALCA | 24 |
| GO:0046890 | BP | GO:0046890 | regulation of lipid biosynthetic process | 12/695 | 189/18870 | 0.047421112 | 0.251408913 | 0.214202826 | SCARB1/APOC1/FGF1/C3/PROX1/TRIB3/CYP27B1/IFNG/APOB/SERPINA12/FSHB/APOA4 | 12 |
| GO:0030099 | BP | GO:0030099 | myeloid cell differentiation | 23/695 | 430/18870 | 0.048026955 | 0.253506273 | 0.215989797 | TREM2/VEGFA/TNFAIP6/MTURN/LILRB4/SFRP1/LOX/TMEM178A/BATF/IFNG/GPC3/UBD/MMP9/GATA3/MT1G/DCSTAMP/EPO/TF/IL11/POU4F1/SLC4A1/FSHB/NKX2-3 | 23 |
| GO:0009395 | BP | GO:0009395 | phospholipid catabolic process | 5/695 | 54/18870 | 0.048038607 | 0.253506273 | 0.215989797 | SCARB1/APOC1/ENPP6/PLA2G7/PLA2G4D | 5 |
| GO:0060425 | BP | GO:0060425 | lung morphogenesis | 5/695 | 54/18870 | 0.048038607 | 0.253506273 | 0.215989797 | SOX11/TCF21/WNT7B/GRHL2/FGF10 | 5 |
| GO:0098703 | BP | GO:0098703 | calcium ion import across plasma membrane | 5/695 | 54/18870 | 0.048038607 | 0.253506273 | 0.215989797 | TRPV6/TRPV5/SCN7A/SCN2A/SCN10A | 5 |
| GO:0006766 | BP | GO:0006766 | vitamin metabolic process | 8/695 | 109/18870 | 0.048147558 | 0.253564188 | 0.216039141 | NNMT/RBP2/CYP27B1/IFNG/UGT1A3/CYP4F2/TTPA/UGT1A4 | 8 |
| GO:0090092 | BP | GO:0090092 | regulation of transmembrane receptor protein serine/threonine kinase signaling pathway | 17/695 | 297/18870 | 0.048970246 | 0.253564188 | 0.216039141 | TNFAIP6/SOX11/HTRA4/SFRP1/SOST/LOX/PRDM16/DLX1/RASL11B/GDF6/GPC3/TFAP2B/HES5/FGF10/BMP7/GATA4/DMRT1 | 17 |
| GO:0002687 | BP | GO:0002687 | positive regulation of leukocyte migration | 10/695 | 149/18870 | 0.049084001 | 0.253564188 | 0.216039141 | TREM2/VEGFA/PGF/PLA2G7/CCL5/TNFSF14/CXCL10/CXCL13/CCL20/LBP | 10 |
| GO:0009988 | BP | GO:0009988 | cell-cell recognition | 6/695 | 72/18870 | 0.049215712 | 0.253564188 | 0.216039141 | ADAM18/ATP8B3/ZP1/PAEP/ZAN/FOLR3 | 6 |
| GO:1904888 | BP | GO:1904888 | cranial skeletal system development | 6/695 | 72/18870 | 0.049215712 | 0.253564188 | 0.216039141 | WNT9B/TBX15/DLX2/FREM1/GRHL2/LHX1 | 6 |
| GO:0016234 | CC | GO:0016234 | inclusion body | 6/743 | 71/19886 | 0.049258815 | 0.291664038 | 0.255163393 | KLHL14/PRDM16/UBD/HSPB7/MT3/SNCB | 6 |
