# Supplementary material for: Identification and validation of prognostic genes associated with clear cell renal cell carcinoma: based on public whole transcriptome sequencing datasets
Source: Front Oncol. 2026 Jul 8;16:1857894. doi: 10.3389/fonc.2026.1857894 (PMC13388226; doi:10.3389/fonc.2026.1857894)
Supplement: Supplementary Table 3 — Results of KEGG Enrichment Analysis for Differentially Expressed mRNAs. [file Table3.docx]

Table S3. Results of KEGG Enrichment Analysis for Differentially Expressed mRNAs.

| category | subcategory | ID | Description | GeneRatio | BgRatio | pvalue | p.adjust | qvalue | geneID | Count | richFactor |  |
| --- | --- | --- | --- | --- | --- | --- | --- | --- | --- | --- | --- | --- |
| Environmental Information Processing | Signaling molecules and interaction | hsa04080 | Neuroactive ligand-receptor interaction | 38/346 | 368/8842 | 3.31E-08 | 9.96E-06 | 9.30E-06 | GABRD/GRIK3/KISS1R/C3/HTR6/MCHR1/GZMA/HCRTR2/KNG1/AVPR1B/CHRNA1/HRH2/AVPR2/PTH1R/PTGER1/F2/PMCH/P2RX2/PAQR9/CHRNA6/GRM1/GRIA4/SSTR5/GRIK5/CHRND/NMUR2/GABRA2/TACR3/UTS2R/MTNR1A/NPY2R/SST/APELA/PLG/CHRNA4/FSHB/PATE1/CALCA | 38 | 0.103261 |  |
| Organismal Systems | Immune system | hsa04610 | Complement and coagulation cascades | 14/346 | 88/8842 | 7.04E-06 | 0.001059 | 0.000989 | ITGAX/VWF/C3/KNG1/SERPINA5/F2/F11/FGG/C7/CR2/FGA/PLG/FGB/MBL2 | 14 | 0.159091 |  |
| Environmental Information Processing | Signaling molecules and interaction | hsa04060 | Cytokine-cytokine receptor interaction | 27/346 | 298/8842 | 3.93E-05 | 0.003939 | 0.003678 | CD70/INHBB/TNFRSF4/IL2RB/IL20RB/TNFSF9/CCL18/FASLG/CCL5/TNFRSF9/TNFSF14/CXCL9/CXCL10/CXCL11/GDF6/CXCR3/IFNG/CD27/CXCL5/CXCL13/CCL20/EPO/INHBE/IL11/CCL25/BMP7/IFNA14 | 27 | 0.090604 |  |
| Environmental Information Processing | Signaling molecules and interaction | hsa04061 | Viral protein interaction with cytokine and cytokine receptor | 13/346 | 100/8842 | 0.000131 | 0.009894 | 0.009238 | IL2RB/IL20RB/CCL18/CCL5/TNFSF14/CXCL9/CXCL10/CXCL11/CXCR3/CXCL5/CXCL13/CCL20/CCL25 | 13 | 0.13 |  |
| Organismal Systems | Digestive system | hsa04976 | Bile secretion | 12/346 | 90/8842 | 0.000186 | 0.011212 | 0.010469 | SCARB1/SLC22A8/SLC9A3/UGT1A3/AQP9/BAAT/SLC22A7/UGT1A10/ADCY8/UGT2A1/UGT1A4/SULT2A1 | 12 | 0.133333 |  |
| Environmental Information Processing | Signaling molecules and interaction | hsa04514 | Cell adhesion molecules | 16/346 | 157/8842 | 0.000415 | 0.020798 | 0.01942 | CLDN16/CLDN19/HLA-G/CD8A/CD2/ICOS/CD8B/CDH4/PDCD1/NTNG1/SLITRK5/CNTN1/CADM3/VTCN1/L1CAM/CLDN8 | 16 | 0.101911 |  |
| Organismal Systems | Endocrine system | hsa04928 | Parathyroid hormone synthesis | secretion and action | 13/346 | 115/8842 | 0.000533 | 0.020915 | 0.019529 | PTHLH/WNK4/SOST/CASR/CYP27B1/TRPV5/PTH1R/GATA3/SLC34A3/SLC34A1/SLC12A3/MMP13/ADCY8 | 13 | 0.113043 |
| Organismal Systems | Digestive system | hsa04978 | Mineral absorption | 9/346 | 61/8842 | 0.000556 | 0.020915 | 0.019529 | TRPV6/ATP2B2/MT1G/SLC9A3/SLC34A3/SLC34A1/TF/MT1H/S100G | 9 | 0.147541 |  |
| Organismal Systems | Digestive system | hsa04975 | Fat digestion and absorption | 7/346 | 43/8842 | 0.001262 | 0.042214 | 0.039416 | SCARB1/CEL/APOB/FABP1/MOGAT2/PLA2G2D/APOA4 | 7 | 0.162791 |  |
| Human Diseases | Infectious disease: bacterial | hsa05150 | Staphylococcus aureus infection | 11/346 | 100/8842 | 0.00176 | 0.052988 | 0.049476 | FCGR3A/FCGR1A/C3/FGG/KRT36/KRT32/KRT40/KRT25/PLG/KRT20/MBL2 | 11 | 0.11 |  |
| Environmental Information Processing | Signal transduction | hsa04020 | Calcium signaling pathway | 20/346 | 254/8842 | 0.002263 | 0.061921 | 0.057817 | VEGFA/MYLK3/FGF1/EGF/NOS1/HTR6/AVPR1B/HRH2/CALML3/ATP2B2/PTGER1/ITPKA/TNNC1/P2RX2/ERBB4/GRM1/TACR3/FGF10/ADCY8/SLN | 20 | 0.07874 |  |
| Organismal Systems | Digestive system | hsa04971 | Gastric acid secretion | 9/346 | 76/8842 | 0.002732 | 0.068519 | 0.063978 | MYLK3/KCNJ10/KCNJ1/KCNK10/HRH2/CALML3/SLC9A4/SST/ADCY8 | 9 | 0.118421 |  |
| Metabolism | Xenobiotics biodegradation and metabolism | hsa00980 | Metabolism of xenobiotics by cytochrome P450 | 9/346 | 79/8842 | 0.003562 | 0.082479 | 0.077013 | GSTM3/ADH1C/UGT1A3/CYP2B6/ALDH3B2/UGT1A10/UGT2A1/UGT1A4/SULT2A1 | 9 | 0.113924 |  |
| Metabolism | Xenobiotics biodegradation and metabolism | hsa00982 | Drug metabolism - cytochrome P450 | 8/346 | 73/8842 | 0.007424 | 0.157951 | 0.147483 | GSTM3/ADH1C/UGT1A3/CYP2B6/ALDH3B2/UGT1A10/UGT2A1/UGT1A4 | 8 | 0.109589 |  |
| Organismal Systems | Digestive system | hsa04974 | Protein digestion and absorption | 10/346 | 105/8842 | 0.007871 | 0.157951 | 0.147483 | COL23A1/COL5A3/SLC7A8/XPNPEP2/COL4A6/COL26A1/SLC9A3/MEP1A/SLC36A2/CPB1 | 10 | 0.095238 |  |
| Environmental Information Processing | Signal transduction | hsa04066 | HIF-1 signaling pathway | 10/346 | 109/8842 | 0.010146 | 0.183547 | 0.171384 | EGLN3/VEGFA/HK2/ENO2/EGF/ANGPT2/IFNG/ALDOB/EPO/TF | 10 | 0.091743 |  |
| Cellular Processes | Cell motility | hsa04814 | Motor proteins | 15/346 | 197/8842 | 0.010366 | 0.183547 | 0.171384 | DNAH11/MYO3A/TUBA3D/KIF18B/TNNT2/TNNI1/TUBA3E/TNNC1/TNNT1/MYO3B/MYH7/MYH13/TUBA3C/TUBAL3/CAPZA3 | 15 | 0.076142 |  |
| Metabolism | Glycan biosynthesis and metabolism | hsa00534 | Glycosaminoglycan biosynthesis - heparan sulfate / heparin | 4/346 | 24/8842 | 0.013167 | 0.220181 | 0.205589 | HS6ST2/HS3ST2/NDST3/HS3ST5 | 4 | 0.166667 |  |
| Human Diseases | Immune disease | hsa05340 | Primary immunodeficiency | 5/346 | 38/8842 | 0.01548 | 0.245243 | 0.228991 | AICDA/CD8A/ICOS/CD8B/RAG2 | 5 | 0.131579 |  |
| Metabolism | Metabolism of cofactors and vitamins | hsa00830 | Retinol metabolism | 7/346 | 68/8842 | 0.016541 | 0.248949 | 0.232451 | ADH1C/UGT1A3/CYP2B6/UGT1A10/UGT2A1/RDH8/UGT1A4 | 7 | 0.102941 |  |
| Organismal Systems | Digestive system | hsa04977 | Vitamin digestion and absorption | 4/346 | 26/8842 | 0.017426 | 0.249775 | 0.233222 | SCARB1/RBP2/APOB/APOA4 | 4 | 0.153846 |  |
| Organismal Systems | Sensory system | hsa04742 | Taste transduction | 8/346 | 86/8842 | 0.018962 | 0.252246 | 0.235529 | PKD2L1/P2RX2/GRM1/SCNN1B/SCN2A/SCNN1G/GABRA2/ADCY8 | 8 | 0.093023 |  |
| NA | NA | hsa04820 | Cytoskeleton in muscle cells | 16/346 | 232/8842 | 0.019762 | 0.252246 | 0.235529 | COL5A3/ENO2/MYOZ2/COL4A6/TNNT2/TNNI1/SPTBN2/TNNC1/MYBPH/TNNT1/MYH7/FMN2/MYH13/ANKRD2/SGCZ/CAPZA3 | 16 | 0.068966 |  |
| Cellular Processes | Transport and catabolism | hsa04145 | Phagosome | 12/346 | 157/8842 | 0.020113 | 0.252246 | 0.235529 | SCARB1/FCGR3A/PLA2R1/FCGR1A/NOS1/TUBA3D/C3/HLA-G/TUBA3E/TUBA3C/TUBAL3/MBL2 | 12 | 0.076433 |  |
| Human Diseases | Substance dependence | hsa05033 | Nicotine addiction | 5/346 | 41/8842 | 0.021023 | 0.253112 | 0.236338 | GABRD/CHRNA6/GRIA4/GABRA2/CHRNA4 | 5 | 0.121951 |  |
| Metabolism | Metabolism of other amino acids | hsa00430 | Taurine and hypotaurine metabolism | 3/346 | 16/8842 | 0.022771 | 0.263612 | 0.246143 | GADL1/GGT6/BAAT | 3 | 0.1875 |  |
| Metabolism | Energy metabolism | hsa00910 | Nitrogen metabolism | 3/346 | 17/8842 | 0.026866 | 0.299507 | 0.279658 | CA9/CA8/CA1 | 3 | 0.176471 |  |
| Metabolism | Carbohydrate metabolism | hsa00053 | Ascorbate and aldarate metabolism | 4/346 | 30/8842 | 0.028283 | 0.304039 | 0.28389 | UGT1A3/UGT1A10/UGT2A1/UGT1A4 | 4 | 0.133333 |  |
| Metabolism | Lipid metabolism | hsa00590 | Arachidonic acid metabolism | 6/346 | 61/8842 | 0.031322 | 0.315875 | 0.294942 | CYP2J2/ALOX15B/PLA2G4D/CYP4F2/CYP2B6/PLA2G2D | 6 | 0.098361 |  |
| Metabolism | Metabolism of other amino acids | hsa00410 | beta-Alanine metabolism | 4/346 | 31/8842 | 0.031503 | 0.315875 | 0.294942 | ALDH6A1/ABAT/GADL1/ALDH3B2 | 4 | 0.129032 |  |
| Metabolism | Metabolism of cofactors and vitamins | hsa00860 | Porphyrin metabolism | 5/346 | 46/8842 | 0.032859 | 0.315875 | 0.294942 | CP/UGT1A3/UGT1A10/UGT2A1/UGT1A4 | 5 | 0.108696 |  |
| Metabolism | Lipid metabolism | hsa00140 | Steroid hormone biosynthesis | 6/346 | 62/8842 | 0.033581 | 0.315875 | 0.294942 | HSD17B3/UGT1A3/SULT2B1/UGT1A10/UGT2A1/UGT1A4 | 6 | 0.096774 |  |
| Organismal Systems | Endocrine system | hsa04913 | Ovarian steroidogenesis | 5/346 | 51/8842 | 0.048166 | 0.424058 | 0.395956 | SCARB1/CYP2J2/PLA2G4D/ADCY8/FSHB | 5 | 0.098039 |  |
| Organismal Systems | Digestive system | hsa04979 | Cholesterol metabolism | 5/346 | 51/8842 | 0.048166 | 0.424058 | 0.395956 | SCARB1/ANGPTL4/APOC1/APOB/APOA4 | 5 | 0.098039 |  |
| Metabolism | Carbohydrate metabolism | hsa00040 | Pentose and glucuronate interconversions | 4/346 | 36/8842 | 0.050718 | 0.424058 | 0.395956 | UGT1A3/UGT1A10/UGT2A1/UGT1A4 | 4 | 0.111111 |  |
| Metabolism | Amino acid metabolism | hsa00350 | Tyrosine metabolism | 4/346 | 36/8842 | 0.050718 | 0.424058 | 0.395956 | HPD/TYRP1/ADH1C/ALDH3B2 | 4 | 0.111111 |  |
| Human Diseases | Infectious disease: parasitic | hsa05143 | African trypanosomiasis | 4/346 | 37/8842 | 0.055184 | 0.444738 | 0.415265 | IDO1/FASLG/KNG1/IFNG | 4 | 0.108108 |  |
| Organismal Systems | Immune system | hsa04611 | Platelet activation | 9/346 | 125/8842 | 0.056146 | 0.444738 | 0.415265 | MYLK3/PIK3R6/VWF/F2/FGG/PLA2G4D/ADCY8/FGA/FGB | 9 | 0.072 |  |
| Human Diseases | Cancer: overview | hsa05204 | Chemical carcinogenesis - DNA adducts | 6/346 | 71/8842 | 0.05868 | 0.445869 | 0.416321 | GSTM3/UGT1A3/UGT1A10/UGT2A1/UGT1A4/SULT2A1 | 6 | 0.084507 |  |
| Organismal Systems | Excretory system | hsa04960 | Aldosterone-regulated sodium reabsorption | 4/346 | 38/8842 | 0.059855 | 0.445869 | 0.416321 | KCNJ1/FXYD4/SCNN1B/SCNN1G | 4 | 0.105263 |  |
| Environmental Information Processing | Signal transduction | hsa04350 | TGF-beta signaling pathway | 8/346 | 108/8842 | 0.060733 | 0.445869 | 0.416321 | INHBB/GDF6/IFNG/TFR2/HAMP/INHBE/TF/BMP7 | 8 | 0.074074 |  |
| Cellular Processes | Cellular community - eukaryotes | hsa04540 | Gap junction | 7/346 | 92/8842 | 0.068296 | 0.489454 | 0.457018 | EGF/TUBA3D/TUBA3E/GRM1/TUBA3C/ADCY8/TUBAL3 | 7 | 0.076087 |  |
| Human Diseases | Cancer: specific types | hsa05219 | Bladder cancer | 4/346 | 41/8842 | 0.075078 | 0.519712 | 0.48527 | CDKN2A/VEGFA/EGF/MMP9 | 4 | 0.097561 |  |
| Organismal Systems | Immune system | hsa04062 | Chemokine signaling pathway | 12/346 | 193/8842 | 0.075971 | 0.519712 | 0.48527 | PIK3R6/CCL18/CCL5/CXCL9/CXCL10/CXCL11/CXCR3/CXCL5/CXCL13/CCL20/ADCY8/CCL25 | 12 | 0.062176 |  |
| Organismal Systems | Immune system | hsa04657 | IL-17 signaling pathway | 7/346 | 95/8842 | 0.078197 | 0.523054 | 0.488391 | CXCL10/IFNG/MMP9/CXCL5/CCL20/MAPK4/MMP13 | 7 | 0.073684 |  |
| Human Diseases | Endocrine and metabolic disease | hsa04940 | Type I diabetes mellitus | 4/346 | 43/8842 | 0.086208 | 0.557074 | 0.520157 | PTPRN/FASLG/HLA-G/IFNG | 4 | 0.093023 |  |
| Metabolism | Lipid metabolism | hsa01040 | Biosynthesis of unsaturated fatty acids | 3/346 | 27/8842 | 0.086985 | 0.557074 | 0.520157 | SCD/ELOVL2/BAAT | 3 | 0.111111 |  |
| Organismal Systems | Immune system | hsa04640 | Hematopoietic cell lineage | 7/346 | 99/8842 | 0.092633 | 0.580889 | 0.542393 | FCGR1A/CD8A/CD2/CD8B/EPO/CR2/IL11 | 7 | 0.070707 |  |
| Organismal Systems | Endocrine system | hsa04915 | Estrogen signaling pathway | 9/346 | 139/8842 | 0.094653 | 0.581439 | 0.542907 | CALML3/MMP9/KRT36/GRM1/KRT32/KRT40/KRT25/ADCY8/KRT20 | 9 | 0.064748 |  |
| Metabolism | Xenobiotics biodegradation and metabolism | hsa00983 | Drug metabolism - other enzymes | 6/346 | 81/8842 | 0.096905 | 0.583371 | 0.544711 | GSTM3/UPP2/UGT1A3/UGT1A10/UGT2A1/UGT1A4 | 6 | 0.074074 |  |
| Metabolism | Lipid metabolism | hsa00591 | Linoleic acid metabolism | 3/346 | 30/8842 | 0.111017 | 0.644877 | 0.602141 | CYP2J2/PLA2G4D/PLA2G2D | 3 | 0.1 |  |
| Cellular Processes | Cellular community - eukaryotes | hsa04550 | Signaling pathways regulating pluripotency of stem cells | 9/346 | 144/8842 | 0.111407 | 0.644877 | 0.602141 | ESRRB/INHBB/DUSP9/WNT8B/DLX5/POU5F1/WNT9B/INHBE/WNT7B | 9 | 0.0625 |  |
| Environmental Information Processing | Signal transduction | hsa04022 | cGMP-PKG signaling pathway | 10/346 | 166/8842 | 0.1159 | 0.651501 | 0.608326 | MYLK3/PIK3R6/KNG1/CALML3/ATP2B2/MYH7/ADCY8/IRS4/GATA4/KCNU1 | 10 | 0.060241 |  |
| Human Diseases | Cardiovascular disease | hsa05414 | Dilated cardiomyopathy | 7/346 | 105/8842 | 0.116881 | 0.651501 | 0.608326 | NOS1/TNNT2/TNNC1/MYH7/SGCZ/ADCY8/CACNG2 | 7 | 0.066667 |  |
| Metabolism | Carbohydrate metabolism | hsa00010 | Glycolysis / Gluconeogenesis | 5/346 | 67/8842 | 0.120928 | 0.661807 | 0.617949 | HK2/ENO2/ADH1C/ALDOB/ALDH3B2 | 5 | 0.074627 |  |
| Metabolism | Amino acid metabolism | hsa00360 | Phenylalanine metabolism | 2/346 | 16/8842 | 0.127849 | 0.68719 | 0.641649 | HPD/ALDH3B2 | 2 | 0.125 |  |
| Metabolism | Lipid metabolism | hsa00565 | Ether lipid metabolism | 4/346 | 50/8842 | 0.130852 | 0.690989 | 0.645197 | ENPP6/PLA2G7/PLA2G4D/PLA2G2D | 4 | 0.08 |  |
| Human Diseases | Cancer: overview | hsa05207 | Chemical carcinogenesis - receptor activation | 12/346 | 215/8842 | 0.137028 | 0.71113 | 0.664003 | VEGFA/GSTM3/EGF/UGT1A3/KPNA7/CYP2B6/FGF10/UGT1A10/ADCY8/UGT2A1/UGT1A4/CHRNA4 | 12 | 0.055814 |  |
| Environmental Information Processing | Signal transduction | hsa04014 | Ras signaling pathway | 13/346 | 238/8842 | 0.14088 | 0.718729 | 0.671099 | VEGFA/FGF1/PGF/EGF/ANGPT2/FASLG/LAT/CALML3/KSR2/PLA2G4D/PAK6/PLA2G2D/FGF10 | 13 | 0.054622 |  |
| Metabolism | Amino acid metabolism | hsa00290 | Valine | leucine and isoleucine biosynthesis | 1/346 | 4/8842 | 0.147599 | 0.737673 | 0.688787 | SDS | 1 | 0.25 |
| Organismal Systems | Circulatory system | hsa04261 | Adrenergic signaling in cardiomyocytes | 9/346 | 154/8842 | 0.149495 | 0.737673 | 0.688787 | PIK3R6/TNNT2/CALML3/ATP2B2/TNNC1/SCN7A/MYH7/ADCY8/CACNG2 | 9 | 0.058442 |  |
| Organismal Systems | Excretory system | hsa04961 | Endocrine and other factor-regulated calcium reabsorption | 4/346 | 53/8842 | 0.152404 | 0.737713 | 0.688825 | CALB1/TRPV5/PTH1R/ATP2B2 | 4 | 0.075472 |  |
| Organismal Systems | Circulatory system | hsa04270 | Vascular smooth muscle contraction | 8/346 | 134/8842 | 0.154405 | 0.737713 | 0.688825 | MYLK3/AVPR1B/CALML3/PLA2G4D/PLA2G2D/ADCY8/KCNU1/CALCA | 8 | 0.059701 |  |
| Human Diseases | Immune disease | hsa05323 | Rheumatoid arthritis | 6/346 | 94/8842 | 0.162004 | 0.746841 | 0.697347 | VEGFA/CCL5/IFNG/CXCL5/CCL20/IL11 | 6 | 0.06383 |  |
| Organismal Systems | Nervous system | hsa04725 | Cholinergic synapse | 7/346 | 115/8842 | 0.163783 | 0.746841 | 0.697347 | PIK3R6/SLC5A7/CHRNA6/SLC18A3/ADCY8/CHAT/CHRNA4 | 7 | 0.06087 |  |
| Human Diseases | Infectious disease: bacterial | hsa05152 | Tuberculosis | 10/346 | 180/8842 | 0.167788 | 0.746841 | 0.697347 | ITGAX/FCGR3A/PLA2R1/FCGR1A/C3/CYP27B1/IFNG/CALML3/LBP/IFNA14 | 10 | 0.055556 |  |
| Metabolism | Global and overview maps | hsa01200 | Carbon metabolism | 7/346 | 116/8842 | 0.168885 | 0.746841 | 0.697347 | HK2/ENO2/SDS/ALDH6A1/AGXT/ALDOB/HAO1 | 7 | 0.060345 |  |
| Human Diseases | Infectious disease: viral | hsa05160 | Hepatitis C | 9/346 | 159/8842 | 0.170696 | 0.746841 | 0.697347 | SCARB1/CLDN16/EGF/CLDN19/FASLG/CXCL10/IFNG/IFNA14/CLDN8 | 9 | 0.056604 |  |
| Metabolism | Amino acid metabolism | hsa00250 | Alanine | aspartate and glutamate metabolism | 3/346 | 37/8842 | 0.17509 | 0.746841 | 0.697347 | ABAT/AGXT/NAT8L | 3 | 0.081081 |
| Organismal Systems | Endocrine system | hsa03320 | PPAR signaling pathway | 5/346 | 76/8842 | 0.176146 | 0.746841 | 0.697347 | ANGPTL4/FABP6/FABP7/SCD/FABP1 | 5 | 0.065789 |  |
| Metabolism | Biosynthesis of other secondary metabolites | hsa00524 | Neomycin | kanamycin and gentamicin biosynthesis | 1/346 | 5/8842 | 0.18097 | 0.746841 | 0.697347 | HK2 | 1 | 0.2 |
| Metabolism | Lipid metabolism | hsa00100 | Steroid biosynthesis | 2/346 | 20/8842 | 0.183242 | 0.746841 | 0.697347 | CYP27B1/CEL | 2 | 0.1 |  |
| Metabolism | Metabolism of cofactors and vitamins | hsa00760 | Nicotinate and nicotinamide metabolism | 3/346 | 38/8842 | 0.184967 | 0.746841 | 0.697347 | ENPP3/NNMT/NT5C1A | 3 | 0.078947 |  |
| Human Diseases | Immune disease | hsa05330 | Allograft rejection | 3/346 | 38/8842 | 0.184967 | 0.746841 | 0.697347 | FASLG/HLA-G/IFNG | 3 | 0.078947 |  |
| Metabolism | Nucleotide metabolism | hsa00240 | Pyrimidine metabolism | 4/346 | 58/8842 | 0.190993 | 0.746841 | 0.697347 | ENPP3/NT5C1A/UPP2/ENTPD3 | 4 | 0.068966 |  |
| Organismal Systems | Sensory system | hsa04750 | Inflammatory mediator regulation of TRP channels | 6/346 | 99/8842 | 0.191052 | 0.746841 | 0.697347 | CYP2J2/TRPA1/KNG1/CALML3/PLA2G4D/ADCY8 | 6 | 0.060606 |  |
| Human Diseases | Cardiovascular disease | hsa05410 | Hypertrophic cardiomyopathy | 6/346 | 99/8842 | 0.191052 | 0.746841 | 0.697347 | NOS1/TNNT2/TNNC1/MYH7/SGCZ/CACNG2 | 6 | 0.060606 |  |
| Metabolism | Metabolism of cofactors and vitamins | hsa00770 | Pantothenate and CoA biosynthesis | 2/346 | 21/8842 | 0.197598 | 0.755479 | 0.705413 | ENPP3/GADL1 | 2 | 0.095238 |  |
| Organismal Systems | Immune system | hsa04660 | T cell receptor signaling pathway | 7/346 | 122/8842 | 0.200893 | 0.755479 | 0.705413 | CD8A/ICOS/LAT/IFNG/CD8B/PDCD1/PAK6 | 7 | 0.057377 |  |
| Organismal Systems | Endocrine system | hsa04916 | Melanogenesis | 6/346 | 101/8842 | 0.203198 | 0.755479 | 0.705413 | WNT8B/WNT9B/CALML3/TYRP1/WNT7B/ADCY8 | 6 | 0.059406 |  |
| Organismal Systems | Immune system | hsa04612 | Antigen processing and presentation | 5/346 | 80/8842 | 0.203302 | 0.755479 | 0.705413 | HSPA2/HLA-G/CD8A/IFNG/CD8B | 5 | 0.0625 |  |
| Metabolism | Metabolism of other amino acids | hsa00470 | D-Amino acid metabolism | 1/346 | 6/8842 | 0.213038 | 0.781166 | 0.729398 | DAO | 1 | 0.166667 |  |
| Metabolism | Amino acid metabolism | hsa00260 | Glycine | serine and threonine metabolism | 3/346 | 41/8842 | 0.215405 | 0.781166 | 0.729398 | SDS/AGXT/DAO | 3 | 0.073171 |
| Cellular Processes | Cellular community - eukaryotes | hsa04530 | Tight junction | 9/346 | 170/8842 | 0.221807 | 0.790279 | 0.737907 | CLDN16/CLDN19/TUBA3D/TUBA3E/EPB41L4B/TUBA3C/TUBAL3/GATA4/CLDN8 | 9 | 0.052941 |  |
| Organismal Systems | Immune system | hsa04623 | Cytosolic DNA-sensing pathway | 5/346 | 83/8842 | 0.224528 | 0.790279 | 0.737907 | CCL5/CXCL10/AIM2/DNASE2B/IFNA14 | 5 | 0.060241 |  |
| Human Diseases | Cardiovascular disease | hsa05417 | Lipid and atherosclerosis | 11/346 | 216/8842 | 0.225794 | 0.790279 | 0.737907 | HSPA2/CYP2J2/FASLG/CCL5/CALML3/MMP9/APOB/CYP2B6/LBP/IFNA14/APOA4 | 11 | 0.050926 |  |
| Environmental Information Processing | Signal transduction | hsa04072 | Phospholipase D signaling pathway | 8/346 | 149/8842 | 0.228487 | 0.790511 | 0.738123 | EGF/PIK3R6/AVPR1B/AVPR2/F2/PLA2G4D/GRM1/ADCY8 | 8 | 0.053691 |  |
| Human Diseases | Infectious disease: viral | hsa05164 | Influenza A | 9/346 | 172/8842 | 0.231685 | 0.792468 | 0.739951 | FASLG/CCL5/CXCL10/IFNG/KPNA7/TMPRSS4/TMPRSS2/IFNA14/PLG | 9 | 0.052326 |  |
| Human Diseases | Immune disease | hsa05332 | Graft-versus-host disease | 3/346 | 44/8842 | 0.246797 | 0.834672 | 0.779358 | FASLG/HLA-G/IFNG | 3 | 0.068182 |  |
| Organismal Systems | Immune system | hsa04620 | Toll-like receptor signaling pathway | 6/346 | 109/8842 | 0.25431 | 0.850525 | 0.79416 | CCL5/CXCL9/CXCL10/CXCL11/LBP/IFNA14 | 6 | 0.055046 |  |
| Environmental Information Processing | Signaling molecules and interaction | hsa04512 | ECM-receptor interaction | 5/346 | 89/8842 | 0.268758 | 0.877863 | 0.819687 | VWF/COL4A6/IBSP/FREM1/DMP1 | 5 | 0.05618 |  |
| Metabolism | Lipid metabolism | hsa00592 | alpha-Linolenic acid metabolism | 2/346 | 26/8842 | 0.270717 | 0.877863 | 0.819687 | PLA2G4D/PLA2G2D | 2 | 0.076923 |  |
| Metabolism | Metabolism of cofactors and vitamins | hsa00740 | Riboflavin metabolism | 1/346 | 8/8842 | 0.273466 | 0.877863 | 0.819687 | ENPP3 | 1 | 0.125 |  |
| Human Diseases | Cancer: overview | hsa05235 | PD-L1 expression and PD-1 checkpoint pathway in cancer | 5/346 | 90/8842 | 0.276317 | 0.877863 | 0.819687 | EGF/BATF/LAT/IFNG/PDCD1 | 5 | 0.055556 |  |
| Organismal Systems | Immune system | hsa04650 | Natural killer cell mediated cytotoxicity | 7/346 | 135/8842 | 0.277066 | 0.877863 | 0.819687 | FCGR3A/FASLG/HLA-G/LAT/IFNG/KLRK1/IFNA14 | 7 | 0.051852 |  |
| Human Diseases | Substance dependence | hsa05031 | Amphetamine addiction | 4/346 | 69/8842 | 0.284258 | 0.882077 | 0.823622 | SLC6A3/CALML3/PPP1R1B/GRIA4 | 4 | 0.057971 |  |
| Human Diseases | Cardiovascular disease | hsa05416 | Viral myocarditis | 4/346 | 69/8842 | 0.284258 | 0.882077 | 0.823622 | NOS1/HLA-G/MYH7/SGCZ | 4 | 0.057971 |  |
| Cellular Processes | Cell motility | hsa04810 | Regulation of actin cytoskeleton | 11/346 | 230/8842 | 0.289967 | 0.890613 | 0.831592 | MYLK3/FGF1/ITGAX/ITGAD/EGF/KNG1/F2/C7/INSRR/PAK6/FGF10 | 11 | 0.047826 |  |
| Organismal Systems | Nervous system | hsa04724 | Glutamatergic synapse | 6/346 | 115/8842 | 0.294725 | 0.895277 | 0.835947 | GRIK3/PLA2G4D/GRM1/GRIA4/GRIK5/ADCY8 | 6 | 0.052174 |  |
| Organismal Systems | Excretory system | hsa04966 | Collecting duct acid secretion | 2/346 | 28/8842 | 0.300084 | 0.895277 | 0.835947 | CLCNKB/SLC4A1 | 2 | 0.071429 |  |
| Organismal Systems | Immune system | hsa04672 | Intestinal immune network for IgA production | 3/346 | 49/8842 | 0.300409 | 0.895277 | 0.835947 | AICDA/ICOS/CCL25 | 3 | 0.061224 |  |
| Organismal Systems | Sensory system | hsa04744 | Phototransduction | 2/346 | 29/8842 | 0.314702 | 0.89968 | 0.840058 | CALML3/GUCA1C | 2 | 0.068966 |  |
| Human Diseases | Cancer: specific types | hsa05218 | Melanoma | 4/346 | 73/8842 | 0.319826 | 0.89968 | 0.840058 | CDKN2A/FGF1/EGF/FGF10 | 4 | 0.054795 |  |
| Human Diseases | Infectious disease: viral | hsa05171 | Coronavirus disease - COVID-19 | 11/346 | 238/8842 | 0.328786 | 0.89968 | 0.840058 | VWF/C3/CXCL10/F2/FGG/C7/TMPRSS2/FGA/IFNA14/FGB/MBL2 | 11 | 0.046218 |  |
| Organismal Systems | Environmental adaptation | hsa04713 | Circadian entrainment | 5/346 | 97/8842 | 0.330234 | 0.89968 | 0.840058 | NOS1/CALML3/GRIA4/MTNR1A/ADCY8 | 5 | 0.051546 |  |
| Organismal Systems | Digestive system | hsa04970 | Salivary secretion | 5/346 | 97/8842 | 0.330234 | 0.89968 | 0.840058 | TRPV6/NOS1/CALML3/ATP2B2/ADCY8 | 5 | 0.051546 |  |
| Human Diseases | Endocrine and metabolic disease | hsa04936 | Alcoholic liver disease | 7/346 | 144/8842 | 0.333548 | 0.89968 | 0.840058 | SCD/C3/FASLG/ADH1C/FABP1/LBP/IFNA14 | 7 | 0.048611 |  |
| Cellular Processes | Cell growth and death | hsa04115 | p53 signaling pathway | 4/346 | 75/8842 | 0.337749 | 0.89968 | 0.840058 | CDKN2A/IGFBP3/TP73/RPRM | 4 | 0.053333 |  |
| Organismal Systems | Endocrine system | hsa04918 | Thyroid hormone synthesis | 4/346 | 75/8842 | 0.337749 | 0.89968 | 0.840058 | ALB/IYD/SLC26A4/ADCY8 | 4 | 0.053333 |  |
| Organismal Systems | Immune system | hsa04666 | Fc gamma R-mediated phagocytosis | 5/346 | 98/8842 | 0.338038 | 0.89968 | 0.840058 | FCGR3A/FCGR1A/LAT/AMPH/PLA2G4D | 5 | 0.05102 |  |
| Organismal Systems | Endocrine system | hsa04925 | Aldosterone synthesis and secretion | 5/346 | 98/8842 | 0.338038 | 0.89968 | 0.840058 | SCARB1/KCNK9/CALML3/ATP2B2/ADCY8 | 5 | 0.05102 |  |
| Organismal Systems | Endocrine system | hsa04935 | Growth hormone synthesis | secretion and action | 6/346 | 122/8842 | 0.343308 | 0.89968 | 0.840058 | IGFBP3/MRAP2/SSTR5/SST/ADCY8/IRS4 | 6 | 0.04918 |
| Environmental Information Processing | Signal transduction | hsa04151 | PI3K-Akt signaling pathway | 16/346 | 362/8842 | 0.343523 | 0.89968 | 0.840058 | VEGFA/MTCP1/FGF1/IL2RB/PGF/EGF/ANGPT2/PIK3R6/VWF/FASLG/COL4A6/IBSP/EPO/ERBB4/FGF10/IFNA14 | 16 | 0.044199 |  |
| Metabolism | Carbohydrate metabolism | hsa00630 | Glyoxylate and dicarboxylate metabolism | 2/346 | 31/8842 | 0.343714 | 0.89968 | 0.840058 | AGXT/HAO1 | 2 | 0.064516 |  |
| Human Diseases | Immune disease | hsa05320 | Autoimmune thyroid disease | 3/346 | 53/8842 | 0.343731 | 0.89968 | 0.840058 | FASLG/HLA-G/IFNA14 | 3 | 0.056604 |  |
| Metabolism | Metabolism of cofactors and vitamins | hsa00130 | Ubiquinone and other terpenoid-quinone biosynthesis | 1/346 | 11/8842 | 0.355543 | 0.908563 | 0.848353 | HPD | 1 | 0.090909 |  |
| Human Diseases | Infectious disease: parasitic | hsa05140 | Leishmaniasis | 4/346 | 77/8842 | 0.35571 | 0.908563 | 0.848353 | FCGR3A/FCGR1A/C3/IFNG | 4 | 0.051948 |  |
| Metabolism | Carbohydrate metabolism | hsa00640 | Propanoate metabolism | 2/346 | 32/8842 | 0.358075 | 0.908563 | 0.848353 | ALDH6A1/ABAT | 2 | 0.0625 |  |
| Human Diseases | Cancer: specific types | hsa05224 | Breast cancer | 7/346 | 148/8842 | 0.359199 | 0.908563 | 0.848353 | FGF1/WNT8B/EGF/WNT9B/HES5/WNT7B/FGF10 | 7 | 0.047297 |  |
| Human Diseases | Cancer: specific types | hsa05226 | Gastric cancer | 7/346 | 150/8842 | 0.372095 | 0.933339 | 0.871486 | FGF1/WNT8B/EGF/WNT9B/TERT/WNT7B/FGF10 | 7 | 0.046667 |  |
| Human Diseases | Infectious disease: parasitic | hsa05142 | Chagas disease | 5/346 | 103/8842 | 0.377211 | 0.938351 | 0.876166 | C3/FASLG/CCL5/KNG1/IFNG | 5 | 0.048544 |  |
| Genetic Information Processing | Information processing in viruses | hsa03265 | Virion - Ebolavirus | Lyssavirus and Morbillivirus | 1/346 | 12/8842 | 0.380793 | 0.939498 | 0.877237 | CHRNA1 | 1 | 0.083333 |
| Metabolism | Carbohydrate metabolism | hsa00051 | Fructose and mannose metabolism | 2/346 | 34/8842 | 0.386439 | 0.945676 | 0.883005 | HK2/ALDOB | 2 | 0.058824 |  |
| Human Diseases | Infectious disease: bacterial | hsa05130 | Pathogenic Escherichia coli infection | 9/346 | 203/8842 | 0.39925 | 0.957282 | 0.893843 | CLDN16/CLDN19/TUBA3D/FASLG/TUBA3E/F2/TUBA3C/TUBAL3/CLDN8 | 9 | 0.044335 |  |
| Organismal Systems | Endocrine system | hsa04926 | Relaxin signaling pathway | 6/346 | 130/8842 | 0.399662 | 0.957282 | 0.893843 | VEGFA/NOS1/COL4A6/MMP9/MMP13/ADCY8 | 6 | 0.046154 |  |
| Organismal Systems | Digestive system | hsa04972 | Pancreatic secretion | 5/346 | 106/8842 | 0.400723 | 0.957282 | 0.893843 | ATP2B2/CEL/PLA2G2D/CPB1/ADCY8 | 5 | 0.04717 |  |
| Cellular Processes | Transport and catabolism | hsa04146 | Peroxisome | 4/346 | 83/8842 | 0.409432 | 0.970385 | 0.906077 | AGXT/DAO/BAAT/HAO1 | 4 | 0.048193 |  |
| Environmental Information Processing | Signal transduction | hsa04390 | Hippo signaling pathway | 7/346 | 157/8842 | 0.417348 | 0.977005 | 0.912259 | FGF1/WNT8B/WNT9B/GDF6/TP73/WNT7B/BMP7 | 7 | 0.044586 |  |
| Organismal Systems | Nervous system | hsa04730 | Long-term depression | 3/346 | 60/8842 | 0.418717 | 0.977005 | 0.912259 | NOS1/PLA2G4D/GRM1 | 3 | 0.05 |  |
| Metabolism | Global and overview maps | hsa01232 | Nucleotide metabolism | 4/346 | 85/8842 | 0.427169 | 0.989061 | 0.923516 | ENPP3/NT5C1A/UPP2/ENTPD3 | 4 | 0.047059 |  |
| Cellular Processes | Cell growth and death | hsa04210 | Apoptosis | 6/346 | 136/8842 | 0.441821 | 0.999938 | 0.933671 | TUBA3D/FASLG/CTSW/TUBA3E/TUBA3C/TUBAL3 | 6 | 0.044118 |  |
| Organismal Systems | Circulatory system | hsa04260 | Cardiac muscle contraction | 4/346 | 87/8842 | 0.444769 | 0.999938 | 0.933671 | TNNT2/TNNC1/MYH7/CACNG2 | 4 | 0.045977 |  |
| Environmental Information Processing | Signal transduction | hsa04015 | Rap1 signaling pathway | 9/346 | 212/8842 | 0.449925 | 0.999938 | 0.933671 | VEGFA/FGF1/PGF/EGF/ANGPT2/LAT/CALML3/FGF10/ADCY8 | 9 | 0.042453 |  |
| Human Diseases | Cancer: specific types | hsa05217 | Basal cell carcinoma | 3/346 | 63/8842 | 0.450043 | 0.999938 | 0.933671 | WNT8B/WNT9B/WNT7B | 3 | 0.047619 |  |
| Metabolism | Glycan biosynthesis and metabolism | hsa00604 | Glycosphingolipid biosynthesis - ganglio series | 1/346 | 15/8842 | 0.450776 | 0.999938 | 0.933671 | B4GALNT1 | 1 | 0.066667 |  |
| Organismal Systems | Nervous system | hsa04727 | GABAergic synapse | 4/346 | 89/8842 | 0.462202 | 0.999938 | 0.933671 | GABRD/ABAT/GABRA2/ADCY8 | 4 | 0.044944 |  |
| Metabolism | Carbohydrate metabolism | hsa00500 | Starch and sucrose metabolism | 2/346 | 40/8842 | 0.467861 | 0.999938 | 0.933671 | ENPP3/HK2 | 2 | 0.05 |  |
| Environmental Information Processing | Signal transduction | hsa04371 | Apelin signaling pathway | 6/346 | 140/8842 | 0.469623 | 0.999938 | 0.933671 | MYLK3/PIK3R6/NOS1/CALML3/ADCY8/APELA | 6 | 0.042857 |  |
| Organismal Systems | Immune system | hsa04613 | Neutrophil extracellular trap formation | 8/346 | 192/8842 | 0.479571 | 0.999938 | 0.933671 | FCGR3A/VWF/FCGR1A/C3/FGG/AQP9/FGA/FGB | 8 | 0.041667 |  |
| Organismal Systems | Development and regeneration | hsa04380 | Osteoclast differentiation | 6/346 | 142/8842 | 0.483387 | 0.999938 | 0.933671 | TREM2/FCGR3A/LILRB4/FCGR1A/SIRPG/IFNG | 6 | 0.042254 |  |
| Environmental Information Processing | Signal transduction | hsa04630 | JAK-STAT signaling pathway | 7/346 | 168/8842 | 0.487718 | 0.999938 | 0.933671 | IL2RB/EGF/IL20RB/IFNG/EPO/IL11/IFNA14 | 7 | 0.041667 |  |
| Organismal Systems | Immune system | hsa04658 | Th1 and Th2 cell differentiation | 4/346 | 92/8842 | 0.487983 | 0.999938 | 0.933671 | IL2RB/LAT/IFNG/GATA3 | 4 | 0.043478 |  |
| Organismal Systems | Nervous system | hsa04720 | Long-term potentiation | 3/346 | 67/8842 | 0.490695 | 0.999938 | 0.933671 | CALML3/GRM1/ADCY8 | 3 | 0.044776 |  |
| Metabolism | Lipid metabolism | hsa00120 | Primary bile acid biosynthesis | 1/346 | 17/8842 | 0.492991 | 0.999938 | 0.933671 | BAAT | 1 | 0.058824 |  |
| Cellular Processes | Cell growth and death | hsa04216 | Ferroptosis | 2/346 | 42/8842 | 0.493559 | 0.999938 | 0.933671 | CP/TF | 2 | 0.047619 |  |
| Organismal Systems | Endocrine system | hsa04912 | GnRH signaling pathway | 4/346 | 93/8842 | 0.496465 | 0.999938 | 0.933671 | CALML3/PLA2G4D/ADCY8/FSHB | 4 | 0.043011 |  |
| Human Diseases | Cancer: specific types | hsa05225 | Hepatocellular carcinoma | 7/346 | 170/8842 | 0.500282 | 0.999938 | 0.933671 | CDKN2A/GSTM3/WNT8B/WNT9B/TERT/WNT7B/ACTL6B | 7 | 0.041176 |  |
| Environmental Information Processing | Signal transduction | hsa04668 | TNF signaling pathway | 5/346 | 119/8842 | 0.500581 | 0.999938 | 0.933671 | CCL5/CXCL10/MMP9/CXCL5/CCL20 | 5 | 0.042017 |  |
| Organismal Systems | Excretory system | hsa04962 | Vasopressin-regulated water reabsorption | 2/346 | 44/8842 | 0.518459 | 0.999938 | 0.933671 | AQP2/AVPR2 | 2 | 0.045455 |  |
| Human Diseases | Cancer: specific types | hsa05211 | Renal cell carcinoma | 3/346 | 70/8842 | 0.520197 | 0.999938 | 0.933671 | EGLN3/VEGFA/PAK6 | 3 | 0.042857 |  |
| Environmental Information Processing | Signal transduction | hsa04024 | cAMP signaling pathway | 9/346 | 226/8842 | 0.527267 | 0.999938 | 0.933671 | HTR6/CALML3/ATP2B2/PPP1R1B/GRIA4/SSTR5/SST/ADCY8/FSHB | 9 | 0.039823 |  |
| Human Diseases | Infectious disease: viral | hsa05163 | Human cytomegalovirus infection | 9/346 | 226/8842 | 0.527267 | 0.999938 | 0.933671 | CDKN2A/VEGFA/FASLG/CCL5/HLA-G/CALML3/PTGER1/ADCY8/IFNA14 | 9 | 0.039823 |  |
| Environmental Information Processing | Membrane transport | hsa02010 | ABC transporters | 2/346 | 45/8842 | 0.530601 | 0.999938 | 0.933671 | ABCA4/ABCA13 | 2 | 0.044444 |  |
| Organismal Systems | Nervous system | hsa04723 | Retrograde endocannabinoid signaling | 6/346 | 149/8842 | 0.530609 | 0.999938 | 0.933671 | NDUFA4L2/GABRD/GRM1/GRIA4/GABRA2/ADCY8 | 6 | 0.040268 |  |
| Human Diseases | Cancer: specific types | hsa05215 | Prostate cancer | 4/346 | 98/8842 | 0.537932 | 0.999938 | 0.933671 | EGF/MMP9/INSRR/TMPRSS2 | 4 | 0.040816 |  |
| Cellular Processes | Cellular community - eukaryotes | hsa04510 | Focal adhesion | 8/346 | 203/8842 | 0.543312 | 0.999938 | 0.933671 | VEGFA/MYLK3/PGF/EGF/VWF/COL4A6/IBSP/PAK6 | 8 | 0.039409 |  |
| Human Diseases | Infectious disease: viral | hsa05165 | Human papillomavirus infection | 13/346 | 333/8842 | 0.544673 | 0.999938 | 0.933671 | VEGFA/WNT8B/EGF/VWF/WNT9B/FASLG/HLA-G/COL4A6/IBSP/HES5/TERT/WNT7B/IFNA14 | 13 | 0.039039 |  |
| Metabolism | Carbohydrate metabolism | hsa00562 | Inositol phosphate metabolism | 3/346 | 73/8842 | 0.54875 | 0.999938 | 0.933671 | ALDH6A1/ITPKA/PIK3C2G | 3 | 0.041096 |  |
| Human Diseases | Cancer: overview | hsa05205 | Proteoglycans in cancer | 8/346 | 204/8842 | 0.548962 | 0.999938 | 0.933671 | VEGFA/WNT8B/WNT9B/FASLG/GPC3/MMP9/ERBB4/WNT7B | 8 | 0.039216 |  |
| Metabolism | Carbohydrate metabolism | hsa00620 | Pyruvate metabolism | 2/346 | 47/8842 | 0.554253 | 0.999938 | 0.933671 | ADH1C/ACOT12 | 2 | 0.042553 |  |
| Human Diseases | Endocrine and metabolic disease | hsa04930 | Type II diabetes mellitus | 2/346 | 47/8842 | 0.554253 | 0.999938 | 0.933671 | HK2/IRS4 | 2 | 0.042553 |  |
| Metabolism | Global and overview maps | hsa01240 | Biosynthesis of cofactors | 6/346 | 153/8842 | 0.556786 | 0.999938 | 0.933671 | IDO1/HPD/UGT1A3/UGT1A10/UGT2A1/UGT1A4 | 6 | 0.039216 |  |
| Organismal Systems | Endocrine system | hsa04921 | Oxytocin signaling pathway | 6/346 | 154/8842 | 0.563225 | 0.999938 | 0.933671 | MYLK3/PIK3R6/CALML3/PLA2G4D/ADCY8/CACNG2 | 6 | 0.038961 |  |
| Metabolism | Amino acid metabolism | hsa00280 | Valine | leucine and isoleucine degradation | 2/346 | 48/8842 | 0.565759 | 0.999938 | 0.933671 | ALDH6A1/ABAT | 2 | 0.041667 |
| Metabolism | Global and overview maps | hsa01230 | Biosynthesis of amino acids | 3/346 | 75/8842 | 0.567224 | 0.999938 | 0.933671 | ENO2/SDS/ALDOB | 3 | 0.04 |  |
| Human Diseases | Drug resistance: antineoplastic | hsa01524 | Platinum drug resistance | 3/346 | 75/8842 | 0.567224 | 0.999938 | 0.933671 | CDKN2A/GSTM3/FASLG | 3 | 0.04 |  |
| Human Diseases | Endocrine and metabolic disease | hsa04934 | Cushing syndrome | 6/346 | 155/8842 | 0.56962 | 0.999938 | 0.933671 | SCARB1/CDKN2A/WNT8B/WNT9B/WNT7B/ADCY8 | 6 | 0.03871 |  |
| Human Diseases | Cancer: specific types | hsa05214 | Glioma | 3/346 | 76/8842 | 0.576285 | 0.999938 | 0.933671 | CDKN2A/EGF/CALML3 | 3 | 0.039474 |  |
| Human Diseases | Substance dependence | hsa05030 | Cocaine addiction | 2/346 | 49/8842 | 0.577051 | 0.999938 | 0.933671 | SLC6A3/PPP1R1B | 2 | 0.040816 |  |
| Cellular Processes | Cell growth and death | hsa04218 | Cellular senescence | 6/346 | 157/8842 | 0.582272 | 0.999938 | 0.933671 | CDKN2A/IGFBP3/HLA-G/MYBL2/CALML3/GATA4 | 6 | 0.038217 |  |
| Metabolism | Amino acid metabolism | hsa00340 | Histidine metabolism | 1/346 | 22/8842 | 0.584907 | 0.999938 | 0.933671 | ALDH3B2 | 1 | 0.045455 |  |
| Human Diseases | Cancer: specific types | hsa05212 | Pancreatic cancer | 3/346 | 77/8842 | 0.585228 | 0.999938 | 0.933671 | CDKN2A/VEGFA/EGF | 3 | 0.038961 |  |
| Metabolism | Amino acid metabolism | hsa00330 | Arginine and proline metabolism | 2/346 | 50/8842 | 0.588126 | 0.999938 | 0.933671 | NOS1/DAO | 2 | 0.04 |  |
| Metabolism | Carbohydrate metabolism | hsa00520 | Amino sugar and nucleotide sugar metabolism | 2/346 | 50/8842 | 0.588126 | 0.999938 | 0.933671 | HK2/CHIT1 | 2 | 0.04 |  |
| Human Diseases | Infectious disease: parasitic | hsa05144 | Malaria | 2/346 | 50/8842 | 0.588126 | 0.999938 | 0.933671 | IFNG/KLRK1 | 2 | 0.04 |  |
| Human Diseases | Infectious disease: bacterial | hsa05133 | Pertussis | 3/346 | 78/8842 | 0.594049 | 0.999938 | 0.933671 | C3/CALML3/CXCL5 | 3 | 0.038462 |  |
| Environmental Information Processing | Signal transduction | hsa04068 | FoxO signaling pathway | 5/346 | 133/8842 | 0.600182 | 0.999938 | 0.933671 | EGF/FASLG/RAG2/GRM1/IRS4 | 5 | 0.037594 |  |
| Metabolism | Amino acid metabolism | hsa00220 | Arginine biosynthesis | 1/346 | 23/8842 | 0.601191 | 0.999938 | 0.933671 | NOS1 | 1 | 0.043478 |  |
| Organismal Systems | Excretory system | hsa04964 | Proximal tubule bicarbonate reclamation | 1/346 | 23/8842 | 0.601191 | 0.999938 | 0.933671 | SLC9A3 | 1 | 0.043478 |  |
| Organismal Systems | Nervous system | hsa04721 | Synaptic vesicle cycle | 3/346 | 79/8842 | 0.602748 | 0.999938 | 0.933671 | SLC6A3/SLC18A3/CPLX2 | 3 | 0.037975 |  |
| Organismal Systems | Immune system | hsa04659 | Th17 cell differentiation | 4/346 | 108/8842 | 0.6153 | 0.999938 | 0.933671 | IL2RB/LAT/IFNG/GATA3 | 4 | 0.037037 |  |
| Environmental Information Processing | Signal transduction | hsa04010 | MAPK signaling pathway | 11/346 | 300/8842 | 0.631953 | 0.999938 | 0.933671 | VEGFA/FGF1/DUSP9/PGF/EGF/ANGPT2/FASLG/PLA2G4D/ERBB4/FGF10/CACNG2 | 11 | 0.036667 |  |
| Human Diseases | Immune disease | hsa05322 | Systemic lupus erythematosus | 5/346 | 139/8842 | 0.639289 | 0.999938 | 0.933671 | FCGR3A/FCGR1A/C3/IFNG/C7 | 5 | 0.035971 |  |
| Human Diseases | Infectious disease: viral | hsa05167 | Kaposi sarcoma-associated herpesvirus infection | 7/346 | 196/8842 | 0.651519 | 0.999938 | 0.933671 | VEGFA/ANGPT2/PIK3R6/C3/HLA-G/CALML3/IFNA14 | 7 | 0.035714 |  |
| Human Diseases | Cardiovascular disease | hsa05418 | Fluid shear stress and atherosclerosis | 5/346 | 141/8842 | 0.651788 | 0.999938 | 0.933671 | VEGFA/GSTM3/IFNG/CALML3/MMP9 | 5 | 0.035461 |  |
| Metabolism | Global and overview maps | hsa01212 | Fatty acid metabolism | 2/346 | 57/8842 | 0.659621 | 0.999938 | 0.933671 | SCD/ELOVL2 | 2 | 0.035088 |  |
| Environmental Information Processing | Signal transduction | hsa04012 | ErbB signaling pathway | 3/346 | 86/8842 | 0.660141 | 0.999938 | 0.933671 | EGF/ERBB4/PAK6 | 3 | 0.034884 |  |
| Organismal Systems | Endocrine system | hsa04911 | Insulin secretion | 3/346 | 86/8842 | 0.660141 | 0.999938 | 0.933671 | KCNN1/ADCY8/KCNU1 | 3 | 0.034884 |  |
| Human Diseases | Cardiovascular disease | hsa05412 | Arrhythmogenic right ventricular cardiomyopathy | 3/346 | 86/8842 | 0.660141 | 0.999938 | 0.933671 | NOS1/SGCZ/CACNG2 | 3 | 0.034884 |  |
| Metabolism | Lipid metabolism | hsa00062 | Fatty acid elongation | 1/346 | 27/8842 | 0.660199 | 0.999938 | 0.933671 | ELOVL2 | 1 | 0.037037 |  |
| Metabolism | Carbohydrate metabolism | hsa00650 | Butanoate metabolism | 1/346 | 27/8842 | 0.660199 | 0.999938 | 0.933671 | ABAT | 1 | 0.037037 |  |
| Organismal Systems | Nervous system | hsa04726 | Serotonergic synapse | 4/346 | 115/8842 | 0.66442 | 0.999938 | 0.933671 | CYP2J2/HTR6/ALOX15B/PLA2G4D | 4 | 0.034783 |  |
| Metabolism | Metabolism of other amino acids | hsa00480 | Glutathione metabolism | 2/346 | 58/8842 | 0.668983 | 0.999938 | 0.933671 | GSTM3/GGT6 | 2 | 0.034483 |  |
| Organismal Systems | Immune system | hsa04670 | Leukocyte transendothelial migration | 4/346 | 116/8842 | 0.671078 | 0.999938 | 0.933671 | CLDN16/CLDN19/MMP9/CLDN8 | 4 | 0.034483 |  |
| Metabolism | Glycan biosynthesis and metabolism | hsa00601 | Glycosphingolipid biosynthesis - lacto and neolacto series | 1/346 | 28/8842 | 0.673537 | 0.999938 | 0.933671 | B4GALNT2 | 1 | 0.035714 |  |
| Organismal Systems | Endocrine system | hsa04923 | Regulation of lipolysis in adipocytes | 2/346 | 59/8842 | 0.678135 | 0.999938 | 0.933671 | ADCY8/IRS4 | 2 | 0.033898 |  |
| Environmental Information Processing | Signal transduction | hsa04310 | Wnt signaling pathway | 6/346 | 174/8842 | 0.681493 | 0.999938 | 0.933671 | WNT8B/SFRP1/WNT9B/SOST/WNT7B/TBL1Y | 6 | 0.034483 |  |
| Environmental Information Processing | Signal transduction | hsa04370 | VEGF signaling pathway | 2/346 | 60/8842 | 0.68708 | 0.999938 | 0.933671 | VEGFA/PLA2G4D | 2 | 0.033333 |  |
| Human Diseases | Substance dependence | hsa05032 | Morphine addiction | 3/346 | 91/8842 | 0.697328 | 0.999938 | 0.933671 | GABRD/GABRA2/ADCY8 | 3 | 0.032967 |  |
| Human Diseases | Drug resistance: antineoplastic | hsa01523 | Antifolate resistance | 1/346 | 30/8842 | 0.698666 | 0.999938 | 0.933671 | FOLR3 | 1 | 0.033333 |  |
| Organismal Systems | Aging | hsa04213 | Longevity regulating pathway - multiple species | 2/346 | 62/8842 | 0.704359 | 0.999938 | 0.933671 | ADCY8/IRS4 | 2 | 0.032258 |  |
| Metabolism | Carbohydrate metabolism | hsa00030 | Pentose phosphate pathway | 1/346 | 31/8842 | 0.710498 | 0.999938 | 0.933671 | ALDOB | 1 | 0.032258 |  |
| Human Diseases | Immune disease | hsa05310 | Asthma | 1/346 | 31/8842 | 0.710498 | 0.999938 | 0.933671 | PRG2 | 1 | 0.032258 |  |
| Metabolism | Carbohydrate metabolism | hsa00052 | Galactose metabolism | 1/346 | 32/8842 | 0.721867 | 0.999938 | 0.933671 | HK2 | 1 | 0.03125 |  |
| Cellular Processes | Cell growth and death | hsa04215 | Apoptosis - multiple species | 1/346 | 32/8842 | 0.721867 | 0.999938 | 0.933671 | BIRC7 | 1 | 0.03125 |  |
| Metabolism | Lipid metabolism | hsa00561 | Glycerolipid metabolism | 2/346 | 65/8842 | 0.728784 | 0.999938 | 0.933671 | CEL/MOGAT2 | 2 | 0.030769 |  |
| Organismal Systems | Endocrine system | hsa04927 | Cortisol synthesis and secretion | 2/346 | 65/8842 | 0.728784 | 0.999938 | 0.933671 | SCARB1/ADCY8 | 2 | 0.030769 |  |
| Organismal Systems | Endocrine system | hsa04929 | GnRH secretion | 2/346 | 65/8842 | 0.728784 | 0.999938 | 0.933671 | KISS1R/KCNN1 | 2 | 0.030769 |  |
| Human Diseases | Immune disease | hsa05321 | Inflammatory bowel disease | 2/346 | 65/8842 | 0.728784 | 0.999938 | 0.933671 | IFNG/GATA3 | 2 | 0.030769 |  |
| Organismal Systems | Development and regeneration | hsa04360 | Axon guidance | 6/346 | 184/8842 | 0.732181 | 0.999938 | 0.933671 | SEMA5B/UNC5A/NTNG1/PAK6/L1CAM/BMP7 | 6 | 0.032609 |  |
| Metabolism | Global and overview maps | hsa01210 | 2-Oxocarboxylic acid metabolism | 1/346 | 33/8842 | 0.73279 | 0.999938 | 0.933671 | AGXT | 1 | 0.030303 |  |
| Metabolism | Nucleotide metabolism | hsa00230 | Purine metabolism | 4/346 | 128/8842 | 0.743842 | 0.999938 | 0.933671 | ENPP3/NT5C1A/ENTPD3/ADCY8 | 4 | 0.03125 |  |
| Environmental Information Processing | Signal transduction | hsa04070 | Phosphatidylinositol signaling system | 3/346 | 98/8842 | 0.744102 | 0.999938 | 0.933671 | CALML3/ITPKA/PIK3C2G | 3 | 0.030612 |  |
| Human Diseases | Drug resistance: antineoplastic | hsa01522 | Endocrine resistance | 3/346 | 99/8842 | 0.750292 | 0.999938 | 0.933671 | CDKN2A/MMP9/ADCY8 | 3 | 0.030303 |  |
| Human Diseases | Cancer: overview | hsa05231 | Choline metabolism in cancer | 3/346 | 99/8842 | 0.750292 | 0.999938 | 0.933671 | EGF/SLC5A7/PLA2G4D | 3 | 0.030303 |  |
| Organismal Systems | Immune system | hsa04621 | NOD-like receptor signaling pathway | 6/346 | 189/8842 | 0.755292 | 0.999938 | 0.933671 | CCL5/GBP5/CASR/CASP5/AIM2/IFNA14 | 6 | 0.031746 |  |
| Metabolism | Lipid metabolism | hsa00564 | Glycerophospholipid metabolism | 3/346 | 100/8842 | 0.756362 | 0.999938 | 0.933671 | PLA2G4D/PLA2G2D/CHAT | 3 | 0.03 |  |
| Organismal Systems | Immune system | hsa04664 | Fc epsilon RI signaling pathway | 2/346 | 69/8842 | 0.758675 | 0.999938 | 0.933671 | LAT/PLA2G4D | 2 | 0.028986 |  |
| Metabolism | Glycan biosynthesis and metabolism | hsa00512 | Mucin type O-glycan biosynthesis | 1/346 | 36/8842 | 0.763059 | 0.999938 | 0.933671 | GALNTL5 | 1 | 0.027778 |  |
| Organismal Systems | Nervous system | hsa04728 | Dopaminergic synapse | 4/346 | 132/8842 | 0.765195 | 0.999938 | 0.933671 | SLC6A3/CALML3/PPP1R1B/GRIA4 | 4 | 0.030303 |  |
| Metabolism | Global and overview maps | hsa01250 | Biosynthesis of nucleotide sugars | 1/346 | 37/8842 | 0.772369 | 0.999938 | 0.933671 | HK2 | 1 | 0.027027 |  |
| Human Diseases | Cancer: overview | hsa05230 | Central carbon metabolism in cancer | 2/346 | 71/8842 | 0.772525 | 0.999938 | 0.933671 | SLC16A3/HK2 | 2 | 0.028169 |  |
| Organismal Systems | Immune system | hsa04622 | RIG-I-like receptor signaling pathway | 2/346 | 72/8842 | 0.779188 | 0.999938 | 0.933671 | CXCL10/IFNA14 | 2 | 0.027778 |  |
| Environmental Information Processing | Signal transduction | hsa04064 | NF-kappa B signaling pathway | 3/346 | 105/8842 | 0.784948 | 0.999938 | 0.933671 | TNFSF14/LAT/LBP | 3 | 0.028571 |  |
| Human Diseases | Cancer: specific types | hsa05223 | Non-small cell lung cancer | 2/346 | 73/8842 | 0.785679 | 0.999938 | 0.933671 | CDKN2A/EGF | 2 | 0.027397 |  |
| Human Diseases | Neurodegenerative disease | hsa05022 | Pathways of neurodegeneration - multiple diseases | 16/346 | 483/8842 | 0.791089 | 0.999938 | 0.933671 | NDUFA4L2/SLC6A3/WNT8B/DNAH11/NOS1/TUBA3D/WNT9B/FASLG/CALML3/SPTBN2/TUBA3E/GRM1/GRIA4/WNT7B/TUBA3C/TUBAL3 | 16 | 0.033126 |  |
| Human Diseases | Infectious disease: viral | hsa05162 | Measles | 4/346 | 139/8842 | 0.799197 | 0.999938 | 0.933671 | IL2RB/FASLG/TP73/IFNA14 | 4 | 0.028777 |  |
| Metabolism | Amino acid metabolism | hsa00380 | Tryptophan metabolism | 1/346 | 42/8842 | 0.813723 | 0.999938 | 0.933671 | IDO1 | 1 | 0.02381 |  |
| Metabolism | Glycan biosynthesis and metabolism | hsa00513 | Various types of N-glycan biosynthesis | 1/346 | 42/8842 | 0.813723 | 0.999938 | 0.933671 | B4GALNT4 | 1 | 0.02381 |  |
| Human Diseases | Infectious disease: parasitic | hsa05145 | Toxoplasmosis | 3/346 | 111/8842 | 0.815538 | 0.999938 | 0.933671 | BIRC7/PIK3R6/IFNG | 3 | 0.027027 |  |
| Metabolism | Lipid metabolism | hsa00071 | Fatty acid degradation | 1/346 | 43/8842 | 0.821047 | 0.999938 | 0.933671 | ADH1C | 1 | 0.023256 |  |
| Human Diseases | Drug resistance: antineoplastic | hsa01521 | EGFR tyrosine kinase inhibitor resistance | 2/346 | 80/8842 | 0.82659 | 0.999938 | 0.933671 | VEGFA/EGF | 2 | 0.025 |  |
| Genetic Information Processing | Replication and repair | hsa03410 | Base excision repair | 1/346 | 44/8842 | 0.828084 | 0.999938 | 0.933671 | NEIL3 | 1 | 0.022727 |  |
| Genetic Information Processing | Folding | sorting and degradation | hsa03050 | Proteasome | 1/346 | 46/8842 | 0.84134 | 0.999938 | 0.933671 | IFNG | 1 | 0.021739 |
| Metabolism | Glycan biosynthesis and metabolism | hsa00514 | Other types of O-glycan biosynthesis | 1/346 | 47/8842 | 0.847582 | 0.999938 | 0.933671 | GALNTL5 | 1 | 0.021277 |  |
| Organismal Systems | Nervous system | hsa04722 | Neurotrophin signaling pathway | 3/346 | 120/8842 | 0.854469 | 0.999938 | 0.933671 | FASLG/CALML3/TP73 | 3 | 0.025 |  |
| Organismal Systems | Endocrine system | hsa04919 | Thyroid hormone signaling pathway | 3/346 | 122/8842 | 0.862082 | 0.999938 | 0.933671 | DIO1/MYH7/GATA4 | 3 | 0.02459 |  |
| Human Diseases | Infectious disease: bacterial | hsa05132 | Salmonella infection | 7/346 | 251/8842 | 0.867007 | 0.999938 | 0.933671 | AHNAK2/TUBA3D/CASP5/TUBA3E/PIK3C2G/TUBA3C/TUBAL3 | 7 | 0.027888 |  |
| Cellular Processes | Transport and catabolism | hsa04144 | Endocytosis | 7/346 | 252/8842 | 0.8696 | 0.999938 | 0.933671 | IL2RB/SH3GL3/HLA-G/AMPH/EPN3/FOLR3/CAPZA3 | 7 | 0.027778 |  |
| Organismal Systems | Aging | hsa04211 | Longevity regulating pathway | 2/346 | 90/8842 | 0.872894 | 0.999938 | 0.933671 | ADCY8/IRS4 | 2 | 0.022222 |  |
| Cellular Processes | Cell growth and death | hsa04217 | Necroptosis | 4/346 | 159/8842 | 0.874887 | 0.999938 | 0.933671 | FASLG/IFNG/PLA2G4D/IFNA14 | 4 | 0.025157 |  |
| Metabolism | Amino acid metabolism | hsa00270 | Cysteine and methionine metabolism | 1/346 | 52/8842 | 0.875301 | 0.999938 | 0.933671 | SDS | 1 | 0.019231 |  |
| Organismal Systems | Digestive system | hsa04973 | Carbohydrate digestion and absorption | 1/346 | 52/8842 | 0.875301 | 0.999938 | 0.933671 | HK2 | 1 | 0.019231 |  |
| Organismal Systems | Immune system | hsa04662 | B cell receptor signaling pathway | 2/346 | 91/8842 | 0.87684 | 0.999938 | 0.933671 | LILRB4/CR2 | 2 | 0.021978 |  |
| Human Diseases | Cancer: overview | hsa05202 | Transcriptional misregulation in cancer | 5/346 | 193/8842 | 0.879439 | 0.999938 | 0.933671 | IGFBP3/IL2RB/FCGR1A/MMP9/TMPRSS2 | 5 | 0.025907 |  |
| Human Diseases | Cancer: specific types | hsa05222 | Small cell lung cancer | 2/346 | 93/8842 | 0.884394 | 0.999938 | 0.933671 | BIRC7/COL4A6 | 2 | 0.021505 |  |
| Metabolism | Lipid metabolism | hsa00600 | Sphingolipid metabolism | 1/346 | 54/8842 | 0.884925 | 0.999938 | 0.933671 | B4GALNT1 | 1 | 0.018519 |  |
| Environmental Information Processing | Signal transduction | hsa04340 | Hedgehog signaling pathway | 1/346 | 56/8842 | 0.893808 | 0.999938 | 0.933671 | HHATL | 1 | 0.017857 |  |
| Human Diseases | Infectious disease: bacterial | hsa05134 | Legionellosis | 1/346 | 56/8842 | 0.893808 | 0.999938 | 0.933671 | C3 | 1 | 0.017857 |  |
| Cellular Processes | Transport and catabolism | hsa04142 | Lysosome | 3/346 | 132/8842 | 0.895126 | 0.999938 | 0.933671 | CD68/CTSW/DNASE2B | 3 | 0.022727 |  |
| Human Diseases | Infectious disease: viral | hsa05169 | Epstein-Barr virus infection | 5/346 | 203/8842 | 0.904301 | 0.999938 | 0.933671 | HLA-G/CXCL10/ENTPD3/CR2/IFNA14 | 5 | 0.024631 |  |
| Human Diseases | Neurodegenerative disease | hsa05010 | Alzheimer disease | 11/346 | 391/8842 | 0.905122 | 0.999938 | 0.933671 | NDUFA4L2/WNT8B/NOS1/TUBA3D/WNT9B/CALML3/TUBA3E/WNT7B/TUBA3C/TUBAL3/IRS4 | 11 | 0.028133 |  |
| Human Diseases | Cancer: specific types | hsa05213 | Endometrial cancer | 1/346 | 59/8842 | 0.905868 | 0.999938 | 0.933671 | EGF | 1 | 0.016949 |  |
| Human Diseases | Endocrine and metabolic disease | hsa04933 | AGE-RAGE signaling pathway in diabetic complications | 2/346 | 101/8842 | 0.910521 | 0.999938 | 0.933671 | VEGFA/COL4A6 | 2 | 0.019802 |  |
| Organismal Systems | Endocrine system | hsa04910 | Insulin signaling pathway | 3/346 | 138/8842 | 0.911368 | 0.999938 | 0.933671 | HK2/CALML3/IRS4 | 3 | 0.021739 |  |
| Human Diseases | Infectious disease: bacterial | hsa05135 | Yersinia infection | 3/346 | 138/8842 | 0.911368 | 0.999938 | 0.933671 | CD8A/LAT/CD8B | 3 | 0.021739 |  |
| Human Diseases | Neurodegenerative disease | hsa05012 | Parkinson disease | 7/346 | 271/8842 | 0.911464 | 0.999938 | 0.933671 | NDUFA4L2/SLC6A3/TUBA3D/CALML3/TUBA3E/TUBA3C/TUBAL3 | 7 | 0.02583 |  |
| Human Diseases | Infectious disease: parasitic | hsa05146 | Amoebiasis | 2/346 | 103/8842 | 0.916131 | 0.999938 | 0.933671 | COL4A6/IFNG | 2 | 0.019417 |  |
| Environmental Information Processing | Signal transduction | hsa04330 | Notch signaling pathway | 1/346 | 62/8842 | 0.916561 | 0.999938 | 0.933671 | HES5 | 1 | 0.016129 |  |
| Metabolism | Amino acid metabolism | hsa00310 | Lysine degradation | 1/346 | 63/8842 | 0.91985 | 0.999938 | 0.933671 | PRDM16 | 1 | 0.015873 |  |
| Human Diseases | Neurodegenerative disease | hsa05020 | Prion disease | 7/346 | 278/8842 | 0.923687 | 0.999938 | 0.933671 | NDUFA4L2/TUBA3D/CCL5/TUBA3E/C7/TUBA3C/TUBAL3 | 7 | 0.02518 |  |
| Human Diseases | Infectious disease: viral | hsa05170 | Human immunodeficiency virus 1 infection | 5/346 | 213/8842 | 0.924586 | 0.999938 | 0.933671 | FASLG/HLA-G/CALML3/PAK6/IFNA14 | 5 | 0.023474 |  |
| Human Diseases | Neurodegenerative disease | hsa05017 | Spinocerebellar ataxia | 3/346 | 144/8842 | 0.925299 | 0.999938 | 0.933671 | SPTBN2/GRM1/MYOD1 | 3 | 0.020833 |  |
| Human Diseases | Neurodegenerative disease | hsa05016 | Huntington disease | 8/346 | 311/8842 | 0.925423 | 0.999938 | 0.933671 | NDUFA4L2/KCNJ10/DNAH11/TUBA3D/TUBA3E/GRIA4/TUBA3C/TUBAL3 | 8 | 0.025723 |  |
| Human Diseases | Cancer: specific types | hsa05221 | Acute myeloid leukemia | 1/346 | 68/8842 | 0.93445 | 0.999938 | 0.933671 | FCGR1A | 1 | 0.014706 |  |
| Organismal Systems | Endocrine system | hsa04924 | Renin secretion | 1/346 | 69/8842 | 0.937035 | 0.999938 | 0.933671 | CALML3 | 1 | 0.014493 |  |
| Organismal Systems | Endocrine system | hsa04920 | Adipocytokine signaling pathway | 1/346 | 70/8842 | 0.939518 | 0.999938 | 0.933671 | IRS4 | 1 | 0.014286 |  |
| Human Diseases | Infectious disease: viral | hsa05166 | Human T-cell leukemia virus 1 infection | 5/346 | 223/8842 | 0.940973 | 0.999938 | 0.933671 | CDKN2A/IL2RB/HLA-G/TERT/ADCY8 | 5 | 0.022422 |  |
| Organismal Systems | Endocrine system | hsa04917 | Prolactin signaling pathway | 1/346 | 71/8842 | 0.941904 | 0.999938 | 0.933671 | ELF5 | 1 | 0.014085 |  |
| Human Diseases | Infectious disease: bacterial | hsa05120 | Epithelial cell signaling in Helicobacter pylori infection | 1/346 | 71/8842 | 0.941904 | 0.999938 | 0.933671 | CCL5 | 1 | 0.014085 |  |
| Cellular Processes | Transport and catabolism | hsa04148 | Efferocytosis | 3/346 | 156/8842 | 0.947332 | 0.999938 | 0.933671 | ANO4/SIRPG/EPO | 3 | 0.019231 |  |
| Environmental Information Processing | Signal transduction | hsa04150 | mTOR signaling pathway | 3/346 | 158/8842 | 0.950358 | 0.999938 | 0.933671 | WNT8B/WNT9B/WNT7B | 3 | 0.018987 |  |
| Human Diseases | Cancer: specific types | hsa05220 | Chronic myeloid leukemia | 1/346 | 77/8842 | 0.954371 | 0.999938 | 0.933671 | CDKN2A | 1 | 0.012987 |  |
| Environmental Information Processing | Signal transduction | hsa04071 | Sphingolipid signaling pathway | 2/346 | 122/8842 | 0.955212 | 0.999938 | 0.933671 | KNG1/SPTSSB | 2 | 0.016393 |  |
| Environmental Information Processing | Signal transduction | hsa04152 | AMPK signaling pathway | 2/346 | 122/8842 | 0.955212 | 0.999938 | 0.933671 | SCD/IRS4 | 2 | 0.016393 |  |
| Genetic Information Processing | Folding | sorting and degradation | hsa03018 | RNA degradation | 1/346 | 78/8842 | 0.956172 | 0.999938 | 0.933671 | ENO2 | 1 | 0.012821 |
| Human Diseases | Infectious disease: viral | hsa05161 | Hepatitis B | 3/346 | 163/8842 | 0.957228 | 0.999938 | 0.933671 | FASLG/MMP9/IFNA14 | 3 | 0.018405 |  |
| Genetic Information Processing | Chromosome | hsa03083 | Polycomb repressive complex | 1/346 | 83/8842 | 0.964169 | 0.999938 | 0.933671 | AURKB | 1 | 0.012048 |  |
| Human Diseases | Cancer: specific types | hsa05210 | Colorectal cancer | 1/346 | 87/8842 | 0.969505 | 0.999938 | 0.933671 | EGF | 1 | 0.011494 |  |
| Metabolism | Energy metabolism | hsa00190 | Oxidative phosphorylation | 2/346 | 138/8842 | 0.973975 | 0.999938 | 0.933671 | NDUFA4L2/ATP12A | 2 | 0.014493 |  |
| Cellular Processes | Cell growth and death | hsa04114 | Oocyte meiosis | 2/346 | 139/8842 | 0.974853 | 0.999938 | 0.933671 | CALML3/ADCY8 | 2 | 0.014388 |  |
| Human Diseases | Cancer: overview | hsa05208 | Chemical carcinogenesis - reactive oxygen species | 4/346 | 226/8842 | 0.979355 | 0.999938 | 0.933671 | NDUFA4L2/VEGFA/GSTM3/EGF | 4 | 0.017699 |  |
| Human Diseases | Substance dependence | hsa05034 | Alcoholism | 3/346 | 188/8842 | 0.98011 | 0.999938 | 0.933671 | SLC6A3/CALML3/PPP1R1B | 3 | 0.015957 |  |
| Human Diseases | Neurodegenerative disease | hsa05014 | Amyotrophic lateral sclerosis | 8/346 | 371/8842 | 0.980217 | 0.999938 | 0.933671 | NDUFA4L2/DNAH11/NOS1/TUBA3D/TUBA3E/ERBB4/TUBA3C/TUBAL3 | 8 | 0.021563 |  |
| Organismal Systems | Environmental adaptation | hsa04714 | Thermogenesis | 4/346 | 235/8842 | 0.984109 | 0.999938 | 0.933671 | NDUFA4L2/PRDM16/ADCY8/ACTL6B | 4 | 0.017021 |  |
| Organismal Systems | Immune system | hsa04625 | C-type lectin receptor signaling pathway | 1/346 | 105/8842 | 0.985253 | 0.999938 | 0.933671 | CALML3 | 1 | 0.009524 |  |
| Organismal Systems | Endocrine system | hsa04922 | Glucagon signaling pathway | 1/346 | 107/8842 | 0.986398 | 0.999938 | 0.933671 | CALML3 | 1 | 0.009346 |  |
| Human Diseases | Endocrine and metabolic disease | hsa04932 | Non-alcoholic fatty liver disease | 2/346 | 157/8842 | 0.986532 | 0.999938 | 0.933671 | NDUFA4L2/FASLG | 2 | 0.012739 |  |
| Genetic Information Processing | Translation | hsa03013 | Nucleocytoplasmic transport | 1/346 | 108/8842 | 0.986937 | 0.999938 | 0.933671 | KPNA7 | 1 | 0.009259 |  |
| Cellular Processes | Cell growth and death | hsa04110 | Cell cycle | 2/346 | 158/8842 | 0.986996 | 0.999938 | 0.933671 | CDKN2A/AURKB | 2 | 0.012658 |  |
| Human Diseases | Endocrine and metabolic disease | hsa04931 | Insulin resistance | 1/346 | 109/8842 | 0.987454 | 0.999938 | 0.933671 | TRIB3 | 1 | 0.009174 |  |
| Human Diseases | Cancer: overview | hsa05203 | Viral carcinogenesis | 3/346 | 205/8842 | 0.988386 | 0.999938 | 0.933671 | CDKN2A/C3/HLA-G | 3 | 0.014634 |  |
| Organismal Systems | Endocrine system | hsa04914 | Progesterone-mediated oocyte maturation | 1/346 | 111/8842 | 0.988429 | 0.999938 | 0.933671 | ADCY8 | 1 | 0.009009 |  |
| Genetic Information Processing | Chromosome | hsa03082 | ATP-dependent chromatin remodeling | 1/346 | 117/8842 | 0.990922 | 0.999938 | 0.933671 | ACTL6B | 1 | 0.008547 |  |
| Genetic Information Processing | Folding | sorting and degradation | hsa04141 | Protein processing in endoplasmic reticulum | 2/346 | 170/8842 | 0.991483 | 0.999938 | 0.933671 | DNAJC5B/CRYAA | 2 | 0.011765 |
| Genetic Information Processing | Folding | sorting and degradation | hsa04120 | Ubiquitin mediated proteolysis | 1/346 | 142/8842 | 0.996704 | 0.999938 | 0.933671 | BIRC7 | 1 | 0.007042 |
| Human Diseases | Infectious disease: bacterial | hsa05131 | Shigellosis | 3/346 | 249/8842 | 0.997262 | 0.999938 | 0.933671 | HK2/C3/CCL5 | 3 | 0.012048 |  |
| Human Diseases | Cardiovascular disease | hsa05415 | Diabetic cardiomyopathy | 2/346 | 205/8842 | 0.99758 | 0.999938 | 0.933671 | NDUFA4L2/MMP9 | 2 | 0.009756 |  |
| Cellular Processes | Transport and catabolism | hsa04140 | Autophagy - animal | 1/346 | 169/8842 | 0.9989 | 0.999938 | 0.933671 | IRS4 | 1 | 0.005917 |  |
| Human Diseases | Cancer: overview | hsa05206 | MicroRNAs in cancer | 3/346 | 312/8842 | 0.999688 | 0.999938 | 0.933671 | CDKN2A/VEGFA/MMP9 | 3 | 0.009615 |  |
| Organismal Systems | Sensory system | hsa04740 | Olfactory transduction | 5/346 | 453/8842 | 0.999938 | 0.999938 | 0.933671 | OR2A4/CALML3/OR10Q1/OR9Q1/OR2AT4 | 5 | 0.011038 |  |
